# PPIH gene regulation system and its prognostic significance in hepatocellular carcinoma: a comprehensive analysis

**DOI:** 10.18632/aging.205134

**Published:** 2023-10-23

**Authors:** Jun Ye, Yilin Pang, Xunjun Yang, Chuan Zhang, Lei Shi, Zhitao Chen, Guijia Huang, Xianhe Wang, Fangyang Lu

**Affiliations:** 1 Department of Clinical Laboratory, The Second Affiliated Hospital of Guizhou Medical University, Kaili, Guizhou 556000, China; 2 School of Laboratory Medicine and Life Sciences, Wenzhou Medical University, Wenzhou, Zhejiang 325035, China; 3 Department of Laboratory Medicine, The Second Affiliated Hospital and Yuying Children’s Hospital of Wenzhou Medical University, Wenzhou, Zhejiang 325027, China; 4 Department of Pathology, The Second Affiliated Hospital of Guizhou Medical University, Kaili, Guizhou 556000, China; 5 Department of Oncology, The Second Affiliated Hospital of Guizhou Medical University, Kaili, Guizhou 556000, China

**Keywords:** PPIH, hepatocellular carcinoma, prognostic biomarker, functional system assessment, TCGA

## Abstract

Background: Peptidyl-prolyl isomerase H (PPIH) is a member of the cyclophilin protein family, which functions as a molecular chaperone and is involved in the splicing of pre-mRNA. According to reports, the malignant progression of HCC related to hepatitis B virus (HBV) is tightly associated with RNA-binding proteins. Nevertheless, there is no research on PPIH expression or its function in the occurrence and progression of HCC.

Results: We are the first to reveal that the mRNA and protein levels of *Ppih* are substantially overexpressed in HCC, as the outcomes show. A significant correlation existed between enriched expression of *Ppih* within HCC and more advanced, poorly differentiated, and TP53-mutated tumors.

Conclusion: These findings, which suggest that *Ppih* may serve as a predictive biomarker for people with HCC, serve as a starting point for further investigation into the function of *Ppih* in the progression of carcinogenesis.

Methods: Accordingly, we utilized clinical samples and bioinformatics analysis to assess *Ppih’s* mRNA, protein expression, and gene regulatory system in HCC. Additionally, Wilcoxon signed-rank testing and logistic regression were utilized to inspect the association between clinicopathological factors and *Ppih*. Clinical pathological traits linked to overall survival (OS) among HCC patients were examined via TCGA data via Cox regression and the Kaplan-Meier approach. Additionally, via TCGA data collection, gene set enrichment assessment was also conducted.

## INTRODUCTION

HCC, which accounts for 75–85% of all liver malignancies, is the third most common cancer fatality worldwide [1–3]. Furthermore, the 5-year survival rate and relapse rates for HCC are very poor [4]. The 5-year survival rate after diagnosis is approximately 50–70%, although surgical treatment may be successful in the early stages of malignancy. In recent years, novel treatment targets for HCC, especially early-phase HCC, have been found via transcriptomics, proteomics, phospho-proteomics, and proteo-genomics [2, 3]. The pathophysiology of HCC is also convoluted. Even though sorafenib and regorafenib are the only systemic treatments that have been shown to improve patients’ chances of surviving with HCC, overall anticancer responses are still few [1, 5]. Accordingly, it is essential to use multi-omics approaches to identify novel diagnostic biomarkers, targets of therapeutic interventions, and prognostic markers of HCC.

The cyclophilin domain is a highly conserved sequence commonly found within the extensive family of proteins known as cyclophilins (Cyps) [6]. These Cyps belong to the immunophilin protein subclass and are highly conserved across species, ranging from bacteria to eukaryotes. Research has demonstrated that Cyps possess both peptidyl-prolyl cis-trans isomerase (PPIase) and molecular chaperone functions [7], making them pivotal regulators of cellular physiology and disease in various inflammatory contexts [8]. The immunosuppressive drug, cyclosporin A, might attenuate the PPIase activity of Cyps [8, 9].

In humans, there exist 7 primary Cyp isoforms. Many human cancers are known to overexpress CypA [7]. Pathological processes integral to cancer onset and progression, including the synthesis of cancer-related proteins and the transmission of signals that foster cancer cell growth, apoptosis, metastasis, and drug resistance, are mediated by CypA [9]. Elevated levels of CypB expression have been identified in HCC [10], breast cancer [11], pancreatic cancer [12], gastric cancer [13], and glioblastoma [14]. Both CypA and CypB are proposed as potential therapeutic targets and biomarkers for the diagnosis of inflammatory conditions [15]. Another member of the Cyp family, hCypH, also recognized as *Ppih*, assists in protein folding by catalyzing the cis-trans isomerization of proline imidic peptide bonds in oligopeptides [16]. Beyond this, *Ppih* plays a crucial role in pre-mRNA splicing and significantly contributes to the assembly of the U4/U5/U6 tri-snRNP complex [16–18]. According to research by Junqing Li et al. [19], heightened *Ppih* expression correlates with a worse prognosis for patients diagnosed with stomach adenocarcinoma (STAD). Moreover, downregulating *Ppih* expression can potentially hinder STAD cell migration and invasion [20]. Another study by Maoshi Li et al. [20], suggests that RNA Binding Proteins (RBPs), including *Ppih*, play a pivotal role in the progression of the malignant trajectory of HBV-related HCC. Therefore, understanding whether *Ppih* is highly expressed, determining how it relates to the prognosis of patients with HCC, and researching the gene regulatory network for *Ppih* in HCC are all of considerable interest.

**Figure 1:**
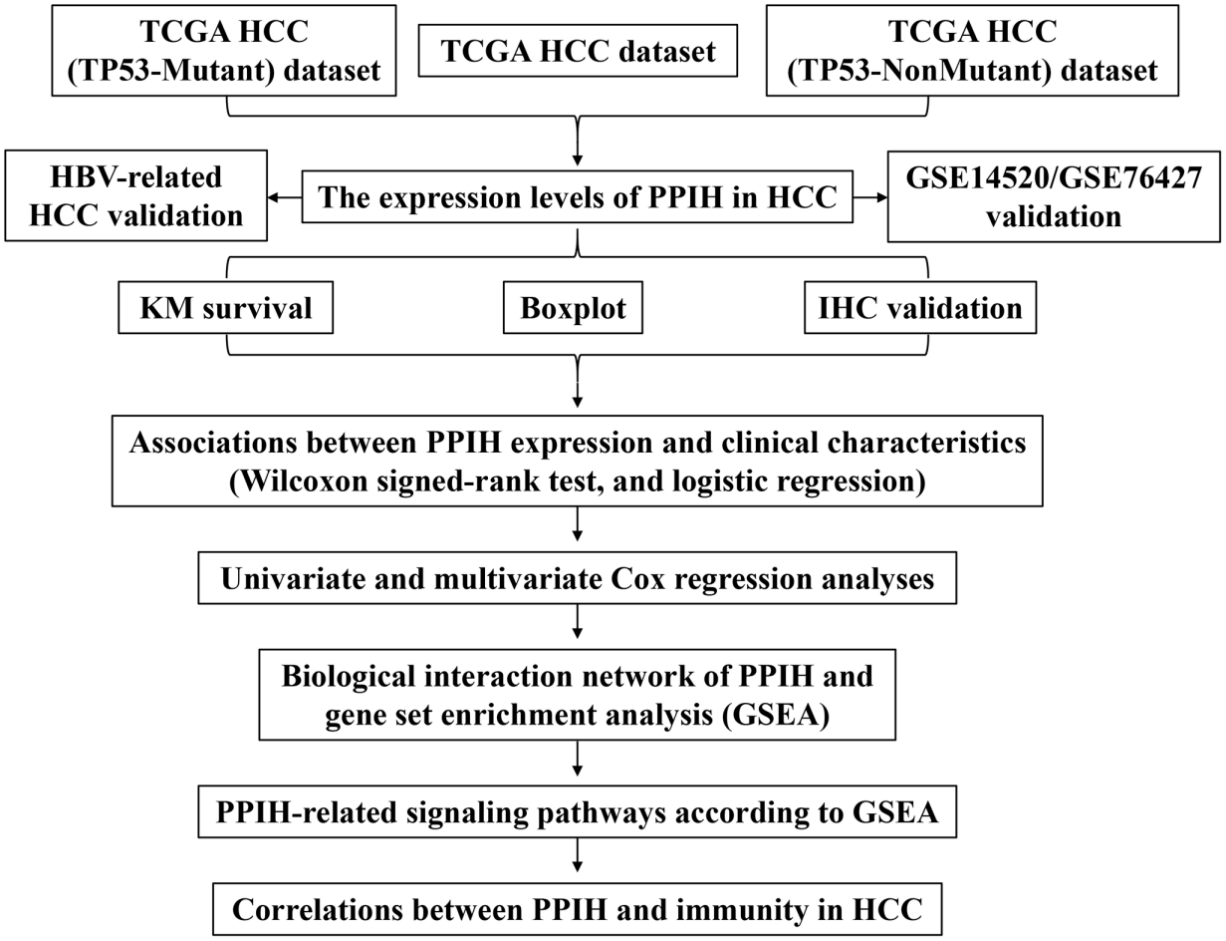
Flowchart of the whole study.

Several informatics technologies were utilized in the current research to inspect *Ppih* expression as well as its predictive value in HCC. Additionally, data from 158 HCC patients with HBV-related disease demonstrated that *Ppih’s* mRNA expression was markedly upregulated. In addition, we utilized immunohistochemistry (IHC) to validate the expression of *Ppih* in HCC tissues. We utilized TCGA, GeneMANIA, and STRING data sources to conduct GSEA and PPI systems to further examine the roles of *Ppih* in HCC. Interestingly, we explored the interaction between *Ppih* and TP53 in relation to HCC. The design and methodology of our study can be seen in the flowchart depicted in Figure 1.

Accordingly, our findings may contribute to a better understanding of *Ppih’s* function in the occurrence and progression of HCC and may point to *Ppih* as a potential novel prognostic indicator and target of therapeutic intervention for the disease.

## RESULTS

### *Ppih* is highly expressed in HCC

**Figure 2:**
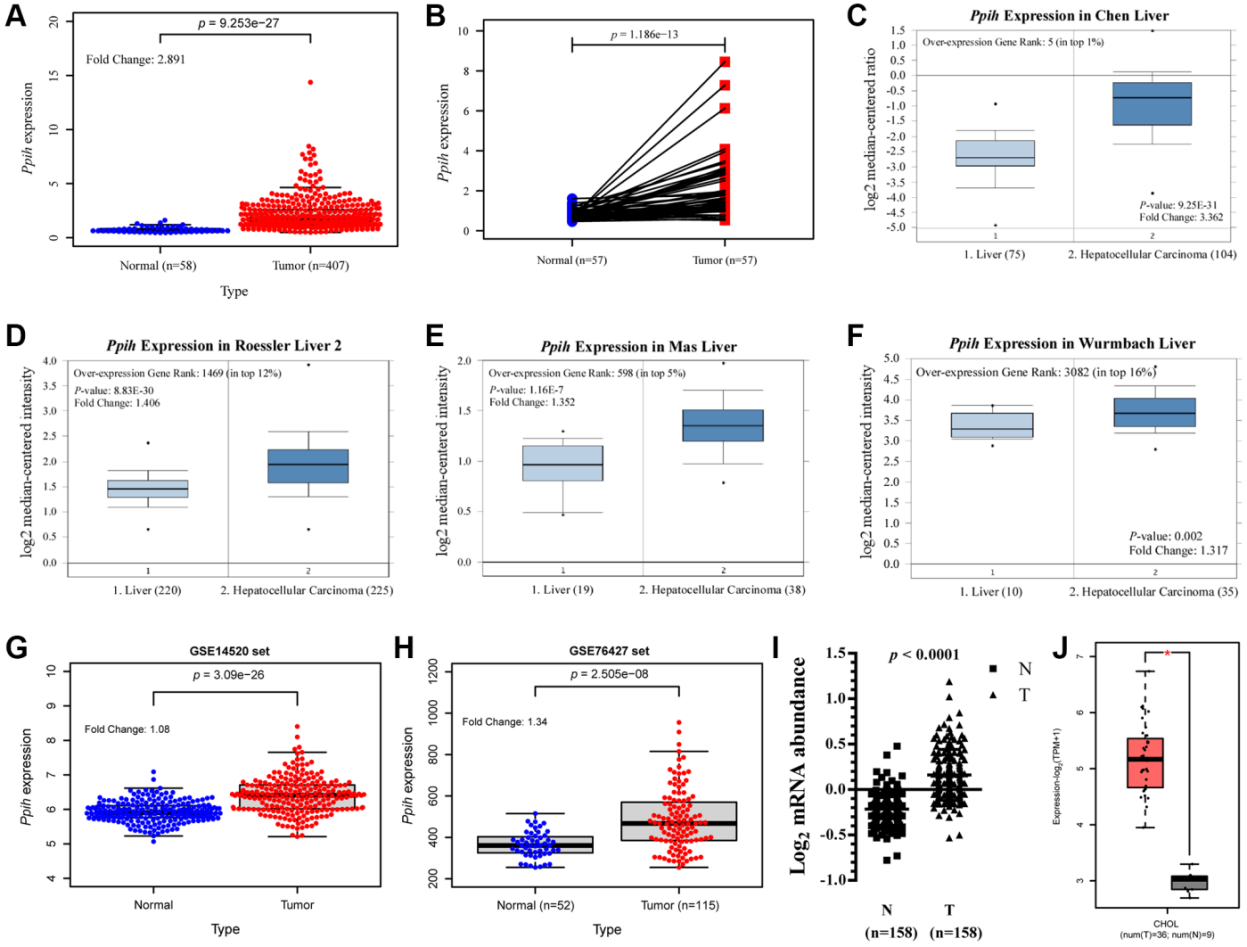
***Ppih* mRNA expression is significantly upregulated in Hepatocellular carcinoma (HCC) compared with normal tissues.** (**A**) Expression of *Ppih* in HCC tissues (TCGA) and healthy liver tissues (TCGA) by RNA-Seq. (**B**) Paired expression data of *Ppih* mRNA in HCC and adjacent normal tissues by RNA-Seq in TCGA dataset. (**C**–**F**) Boxplot showing *Ppih* mRNA expression in liver (left plot) and HCC tissue (right plot) was derived from the Oncomine database. The fold-change in *Ppih* expression in HCC was determined using the Oncomine database. The data are HCC relative to normal liver tissue. The threshold was designated using the following specific parameters: *p* value = 1E-4, fold change = 2, and gene rank 10%. (**G**, **H**) Validation of the expression level of *Ppih* in HCC and normal tissues. GSE14520 and GSE76427 were regarded as the validation set. (**I**) Expression levels of *Ppih* mRNA in HBV-related HCC and paired nontumor liver tissues were investigated using RNA-seq (*n* = 158). These results were obtained from the studies of Gao et al. [17]. (**J**) mRNA expression of *Ppih* in cholangiocarcinoma (CHOL) tissues and adjacent normal tissues from the GEPIA 2 database. ^*^p < 0.05.

The mRNA levels of *Ppih* in cancer and matched specimens in the normal state or neighboring specimens in the normal state were evaluated via various different online data sources to determine the differences in the expression of *Ppih* within HCC and normalized controls. According to boxplots and the paired differential plot (Figure 2A, 2B), HCC specimens had substantially higher mRNA expression for *Ppih* than normal liver tissues (*p* < 0.001). According to the information in the Oncomine 4.5 data sources, HCC was also substantially increased in many datasets for *Ppih’s* transcriptional expression. *Ppih* showed a 3.362-fold increase in HCC in the Chen liver dataset (Figure 2C). Additionally, Roessler, Mas, and Wurmbach discovered that mRNA expression for *Ppih* increased in HCC samples by 1.406-fold, 1.352-fold, and 1.317-fold, respectively (Figure 2D–2F). Consistent with the results in the TCGA set, the boxplots of the two validation sets (GSE14520 and GSE76427) also showed that *Ppih* expression was significantly higher in HCC tissues than in normal tissues (Figure 2G, 2H). The main contributing factor to HCC is chronic hepatitis B and C virus infection [21]. Accordingly, via the RNA sequencing expression data from the research of Gao et al. [21], we examined the expression of *Ppih* in HCC associated with the HBV. According to Figure 2I, HBV-related HCC had substantially higher *Ppih* mRNA levels than matched noncancer liver tissues (*n* = 158) (*p* < 0.0001). Moreover, a multivariate analysis of HCC patients with HBV-related HCC revealed that mRNA expression for *Ppih* was associated with a history of liver cirrhosis (*p* < 0.05) (Supplementary Table 1). Additionally, in the GEPIA2 data sources, customers with cholangiocarcinoma (CHOL) also had enriched *Ppih* mRNA levels (*p* < 0.05). (Figure 2J).

### IHC verification of *Ppih* overexpression in HCC

**Figure 3:**
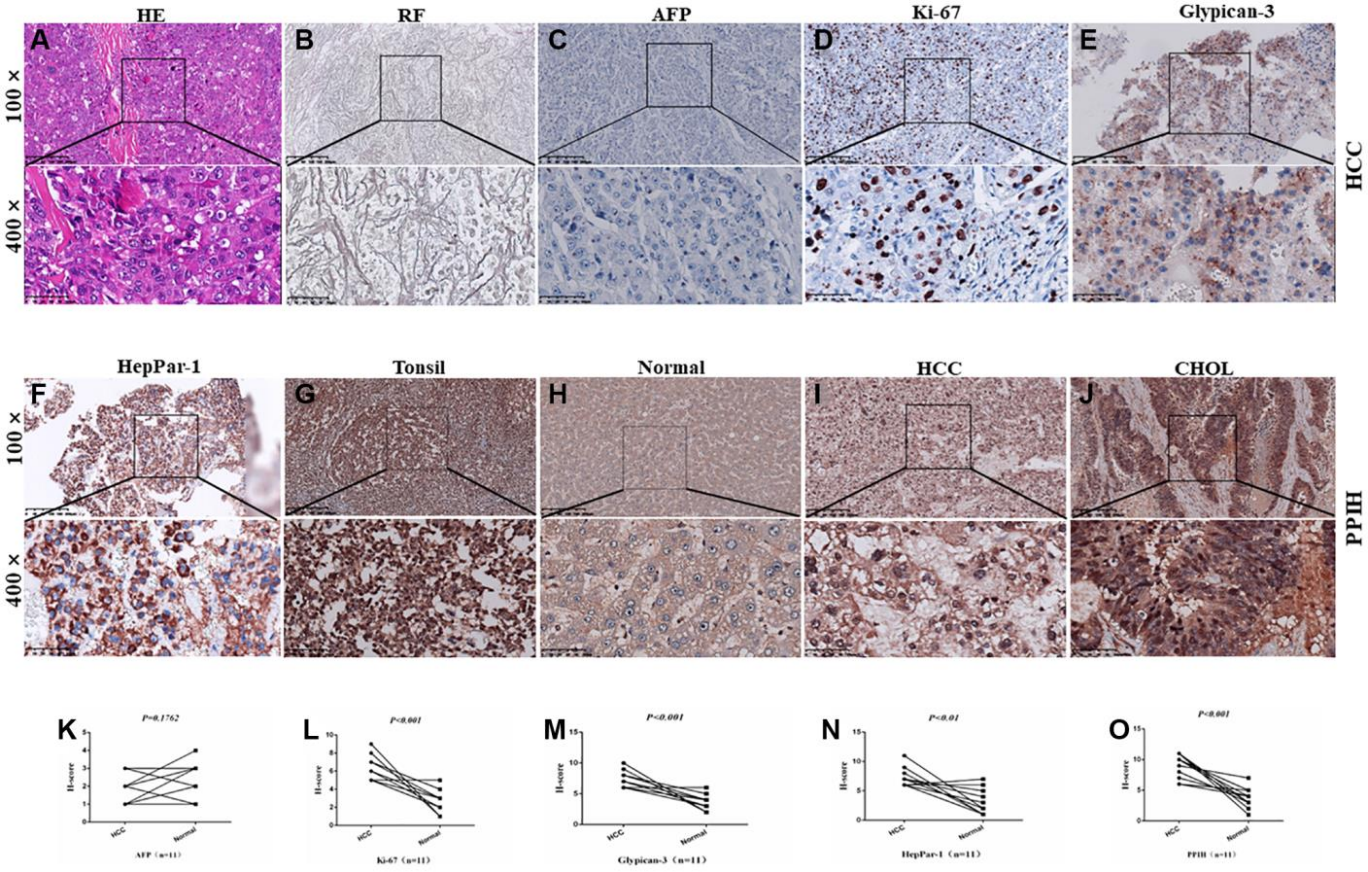
**Diagnosis and IHC identification of HCC.** (**A**) HE staining image of HCC. (**B**) Reticular fiber (RF) staining picture of HCC. (**C**–**F**) IHC for AFP, Ki-67, Glypican-3, and HepPar-1 in HCC. (**G**–**J**) Representative IHC images of PPIH in tonsil (positive control), HCC, CHOL (cholangiocarcinoma) specimens and matched adjacent normal tissues (**H**). Regions in squares are magnified 4×; in bottom panels. (**K**–**O**) Relative AFP (**K**) Ki-67 (**L**) Glypican-3 (**M**) HepPar-1 (**N**) PPIH (**O**) expression scores in HCC and paired normal tissues.

To fully establish whether the obtained tissues were HCC, as depicted in Figure 3A–3F, we first combined conventional H&E staining with reticular fiber staining and IHC markers (AFP, Ki-67, Glypican3, and HepPar-1). Then, IHC staining was performed on HCC tissues that had been embedded in paraffin. On paraffin-embedded slides, protein analysis consistently showed that PPIH, Ki-67, Glypican3, and HepPar-1 were significantly overexpressed in HCC tissues compared to nearby specimens under typical conditions (Figure 3G–3I and 3K–3O). Interestingly, CHOL tissues also showed substantially overexpression of PPIH (Figure 3J).

### Clinicopathological factors associated with mRNA expression for *Ppih* among patients with HCC

**Figure 4:**
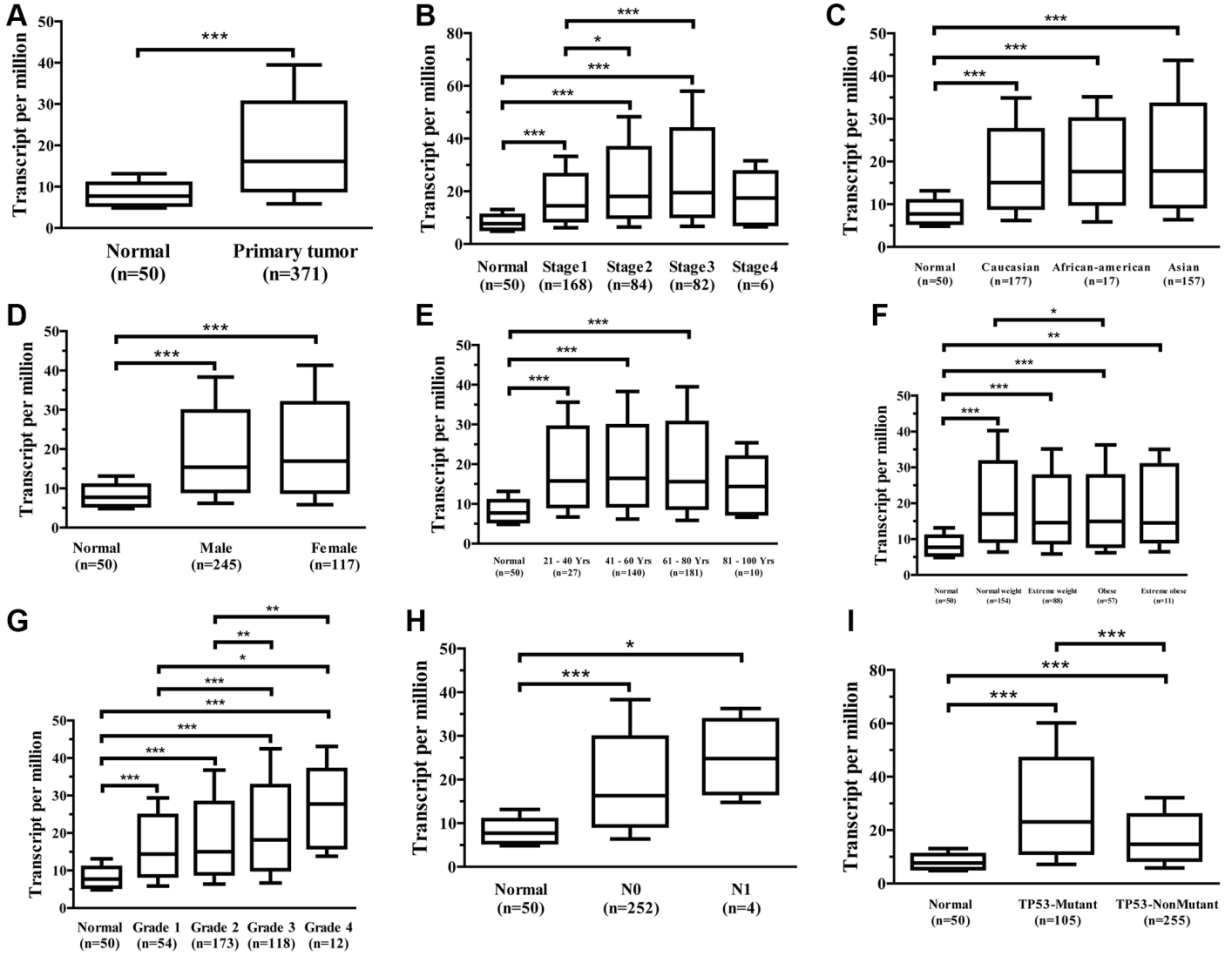
**Association between *Ppih* mRNA expression and clinical characteristics of HCC patients (UALCAN database).** (**A**) Boxplot showing *Ppih* mRNA expression in HCC tissues and adjacent normal liver tissues. (**B**–**I**) Boxplots showing the relative expression of *Ppih* mRNA for the patient characteristics of individual cancer stages (**B**), race (**C**), sex (**D**), age (**E**), weight (**F**), tumor grade (**G**), nodal metastasis status (**H**), and TP53 mutation status (**I**). Data are shown as the mean ± SE. ^*^p < 0.05; ^**^p < 0.01; ^***^p < 0.001.

**Figure 5:**
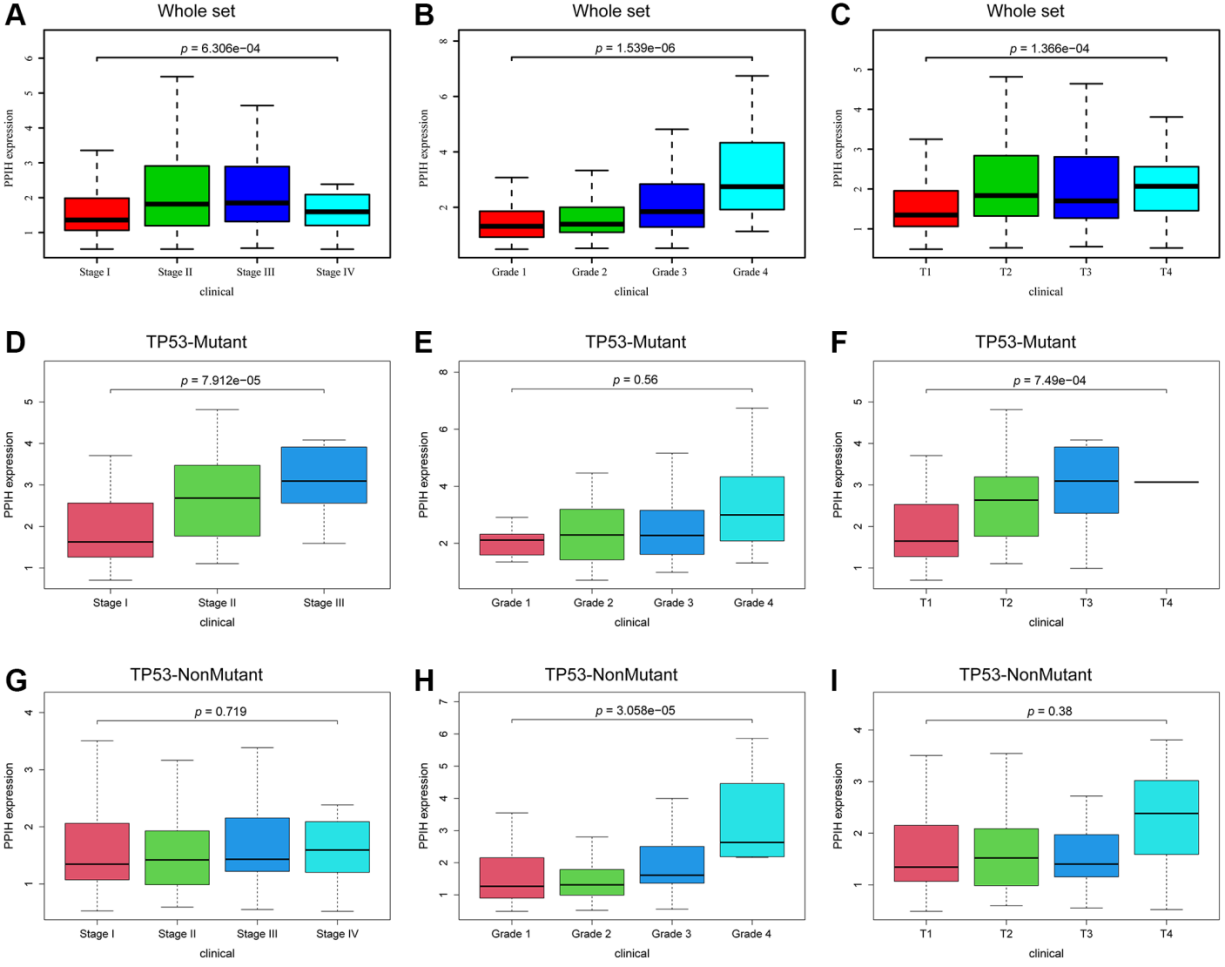
**Association with *Ppih* expression and clinicopathologic characteristics of HCC patients in the TCGA database.** (**A**–**C**) Box plot evaluating *Ppih* expression of HCC patients according to different clinical characteristics, including (**A**) Clinical stage, (**B**) Grade, and (**C**) T stage. (**D**–**I**) The link between *PPIH* mRNA expression and clinicopathologic features in HCC patients with or without mutated TP53. Whole set based on TCGA database, including HCC patients with and without TP53 mutations.

The UALCAN data sources were utilized to further subgroup evaluate the association between mRNA expression for *Ppih* and clinicopathological characteristics of HCC patients. The findings demonstrated a strong correlation between the *Ppih* mRNA expression as well as every client’s specific cancer phase, race, sex, age, weight, cancer grade, nodal metastatic status, and TP53 mutation status (Figure 4A–4I). In instance, mRNA expression for *Ppih* among patients with HCC was substantially related to more advanced (Figure 4B) and less-differentiated tumors (Figure 4G), with greater mRNA expression for *Ppih* levels. Additionally, the greatest mRNA expression levels were observed in stage 3 and/or grade 4 individuals. Stage 4’s limited sample size may be the cause of stage 3’s increased mRNA expression for *Ppih* when compared to stage 4. (Figure 4B). Additionally, mRNA expression for *Ppih* was substantially increased among patients with HCC with TP53 mutations and was favorably correlated with TP53 mutant status (Figure 4I). Additionally, groups identified by the cancer pathological stage (*p* < 0.001, Figure 5A), tumor differentiation (Grade) (*p* < 0.001, Figure 5B), and T stage (*p* < 0.001, Figure 5C) differed in the amount of *Ppih* expression. The correlations between *Ppih* expression and seven clinical parameters (age, sex, grade, stage, T stage, M stage, and N stage) among patients with HCC from the TCGA dataset were further evaluated via logistic regression analysis. The expression level of *Ppih* in HCC steadily rose as the clinical stage, grade, and T stage advanced, as seen in Table 1.

**Table 1:** *Ppih* expression^a^ associated with clinical pathological characteristics of HCC patients (whole set) (logistic regression).

**Clinical characteristics**	**Total (*N*)**	**Odds ratio in PPIH expression**	***p*-Value**
Stage (I vs. II)	261	2.56 (1.51–4.38)	**0.0006**
Stage (I vs. III)	260	2.39 (1.41–4.09)	**0.0014**
Stage (I vs. IV)	180	2.39 (0.39–18.48)	0.3477
Grade (G1 vs. G2)	234	1.23 (0.66–2.33)	0.5193
Grade (G1 vs. G3)	178	3.33 (1.73–6.57)	**0.0004**
Grade (G1 vs. G4)	67	8.75 (2.05–60.75)	**0.0085**
T (T1 vs. T2)	279	2.86 (1.72–4.84)	**6.55e-05**
T (T1 vs. T3)	265	2.20 (1.29–3.77)	**0.0040**
T (T1 vs. T4)	197	3.65 (1.14–13.90)	**0.0367**

^a^Categorical dependent variable, greater or less than the median expression level. The bold values indicate the significant difference between the two groups of data, and the statistical significance was set at *p* < 0.05.

### The effect of TP53 mutation status on the mRNA expression level of PPIH and its correlation with clinicopathological parameters in HCC

**Figure 6:**
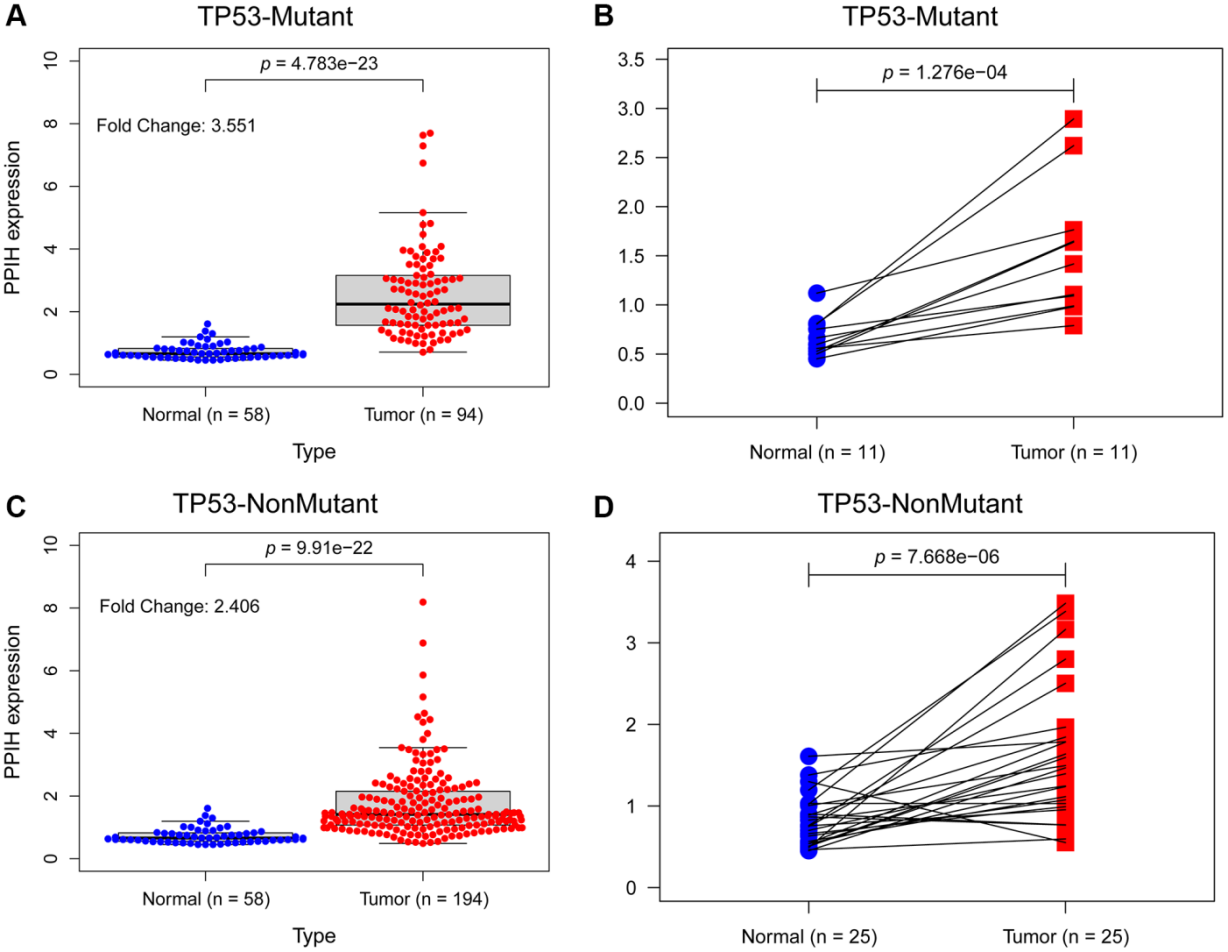
**The effect of TP53 mutation status on the expression level of *Ppih* in HCC.** (**A**) *Ppih* expression in normal and TP53 mutated HCC tissues. (**B**) *Ppih* expression in paired normal and TP53 mutated HCC tissues. (**C**) Expression level of *Ppih* in TP53 nonmutated HCC and normal tissues. (**D**) *Ppih* expression level in HCC and matched normal liver tissues.

To further determine if elevated expression of PPIH mRNA correlates with TP53 mutation status, we sourced somatic gene mutations for American HCC samples (*n* = 374) from the TCGA portal (https://portal.gdc.cancer.gov/). Of these, 91 HCC samples exhibited TP53 mutations, 189 samples did not present any TP53 mutations, and all these samples were accompanied by comprehensive clinical data. Figure 6A–6I illustrates that, in comparison to normal liver tissues, PPIH mRNA was markedly overexpressed in both TP53 mutated (Fold change: 3.551, *p* < 0.001) and non-mutated (Fold change: 2.406, *p* < 0.001) HCC cases. Notably, the heightened expression of PPIH mRNA was more pronounced in TP53 mutated HCC (See Figure 6A, 6C). Furthermore, the expression level of PPIH mRNA demonstrated a positive correlation with the cancer pathological stage (*p* < 0.001, Figure 5D), tumor differentiation grade (Figure 5E), and T stage (*p* < 0.001, Figure 5F) among HCC patients carrying TP53 mutations. However, no significant relationship was discerned between PPIH mRNA expression and these clinical characteristics for HCC patients without TP53 mutations (See Figure 5G–5I). Using logistic regression to delve deeper into the relationship between PPIH mRNA and clinical attributes of HCC patients (both with and without TP53 mutations), we arrived at consistent findings. As the cancer pathological stage and T stage advanced, there was a progressive increase in the expression level of PPIH mRNA in HCC, as delineated in Tables 2 and 3.

**Table 2:** *Ppih* expression^a^ associated with clinical pathological characteristics of HCC patients with mutated p53 (logistic regression).

**Clinical characteristics**	**Total (*N*)**	**Odds ratio in PPIH expression**	***p*-Value**
Stage (I vs. II)	64	1.29 (0.54–2.41)	**0.0160**
Stage (I vs. III)	55	8.89 (2.67–34.27)	**0.0007**
Grade (G1 vs. G2)	46	1.43 (0.21–11.72)	0.7116
Grade (G1 vs. G3)	41	1.42 (0.21–11.75)	0.7172
Grade (G1 vs. G4)	12	3.75 (0.35–53.50)	0.2858
T (T1 vs. T2)	68	2.81 (1.06–7.84)	**0.0418**
T (T1 vs. T3)	56	8.00 (2.44–30.28)	**0.0011**

^a^Categorical dependent variable, greater or less than the median expression level. The bold values indicate the significant difference between the two groups of data, and the statistical significance was set at *p* < 0.05.

**Table 3:** *Ppih* expression^a^ associated with clinical pathological characteristics in HCC patients with unmutated p53 (logistic regression).

**Clinical characteristics**	**Total (*N*)**	**Odds ratio in PPIH expression**	***p*-Value**
Stage (I vs. II)	129	1.25 (0.57–2.76)	0.5773
Stage (I vs. III)	143	1.21 (0.60–2.43)	0.5945
Stage (I vs. IV)	100	1.67 (0.26–13.09)	0.5851
Grade (G1 vs. G2)	123	0.58 (0.25–1.35)	0.2046
Grade (G1 vs. G3)	88	0.58 (1.06–6.75)	**0.0383**
Grade (G1 vs. G4)	33	0.58 (4.76e-53–NA)	0.9889
T (T1 vs. T2)	136	1.45 (0.69–3.12)	0.3290
T (T1 vs. T3)	142	1.18 (0.58–2.41)	0.6524
T (T1 vs. T4)	105	2.94 (0.60–21.25)	0.2100

^a^Categorical dependent variable, greater or less than the median expression level. The bold values indicate the significant difference between the two groups of data, and the statistical significance was set at *p* < 0.05.

### *Ppih* genetic changes and their relationships to the OS and DFS of HCC patients

**Figure 7:**
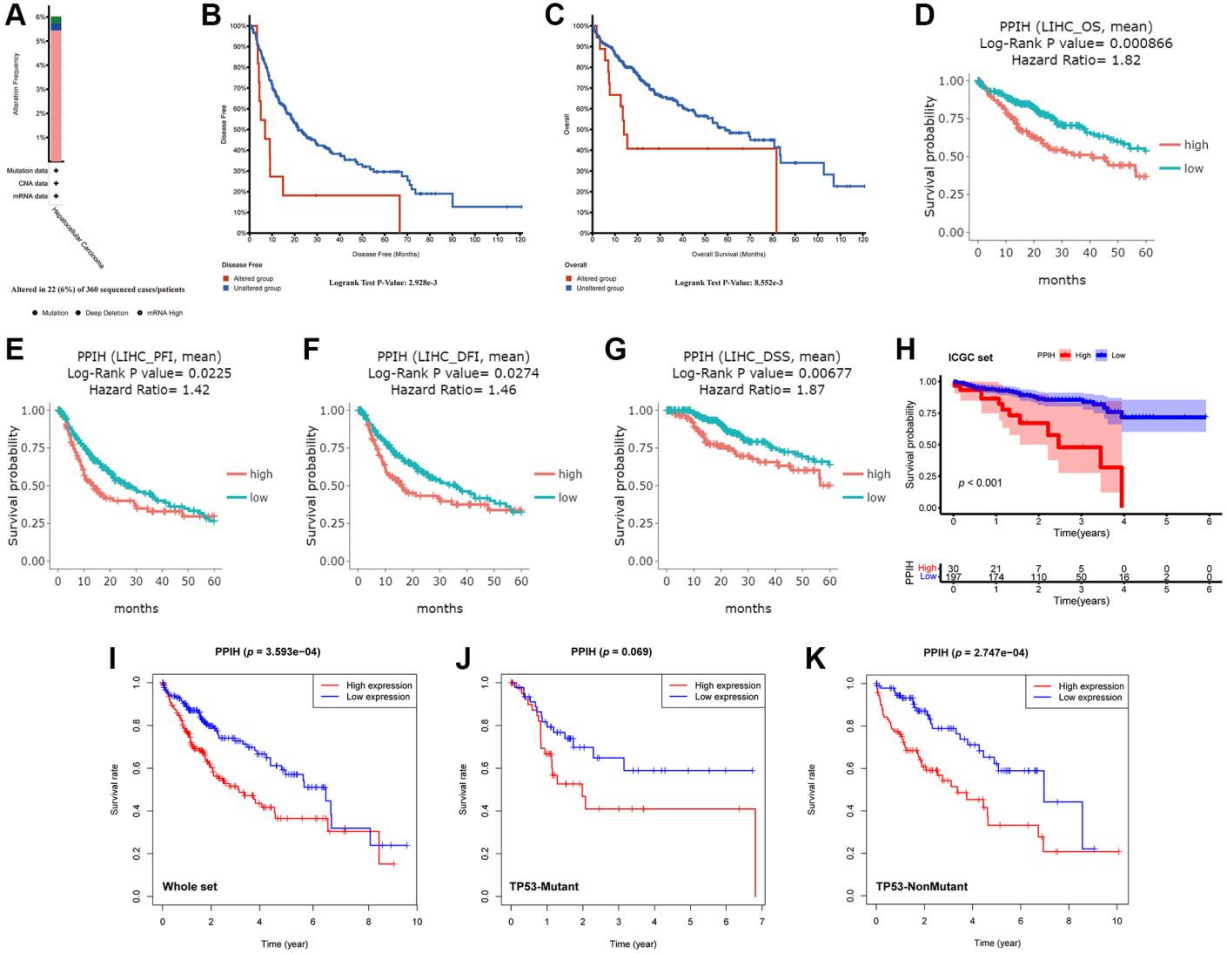
**Genetic alterations and upregulation of *Ppih* were associated with poor prognosis in HCC patients.** (**A**) The PPIH alteration frequency in HCC is presented as a bar diagram. (**B**, **C**) Genetic alterations in PPIH were associated with worse DFS (**B**) and OS (**C**) of HCC patients (cBioPortal database). (**D**–**G**) Graphs generated from the DriverDBV3 database show the prognostic values of PPIH in HCC patients. (**H**) Kaplan-Meier survival analysis of the prognostic value of PPIH in the ICGC validation set. (**I**–**K**) The relationship between *Ppih* expression and OS in HCC patients with or without mutated TP53 based on TCGA database. Abbreviations: OS: overall survival; PFI: progression free interval; DFI: disease free interval; and DSS: disease-specific survival.

The cBioPortal data sources were utilized to inspect genetic variations in *Ppih* and their relationships with OS and DFS among patients with HCC to conduct research on the reasons and importance of enriched *Ppih* expression in HCC. *Ppih* mutation rates among patients with HCC were relatively modest, as shown in Figure 7A. The mutation rate in the 360 sequenced HCC cases/patients was 6%, and 22 HCC patients had genetic changes. Additionally, we noticed that the primary result of the genetic modification in *Ppih* was mRNA upregulation (Figure 7A). Furthermore, a Kaplan-Meier plot and log-ranking testing showed that a genetic change in *Ppih* was associated with a shorter DFS (Figure 7B, *p* = 2.928e-3) and OS (Figure 7C, *p* = 8.552e-3) for HCC patients.

### *Ppih* could function as a standalone prognostic indicator for HCC patients’ OS

We assessed the predictive efficacy of mRNA expression for *Ppih* in patients with HCC via the DriverDBV3 data sources to determine whether high *Ppih* expression may serve as a prognostic signature of HCC patients. High *Ppih* expression was associated with poor OS, progression-free interval (PFI), disease-free interval, and disease-specific survival among patients with HCC, as depicted in Figure 7D–7G (HR = 1.82, log-ranking *p* = 0.0009, PFI, HR = 1.42, log-ranking *p* = 0.0225, DFI, HR = 1.46, log-ranking *p* = 0.0274, DSS, HR = 1.87, log-ranking *p* = 0.0068). To validate the predictive value of the *Ppih*, we plotted the Kaplan-Meier curve with the optimal cutoff score (cutpoint = 22.89) for patients in ICGC set. Consistent with the results in the TCGA cohort, patients in the high-expression group have shown significantly poorer OS than patients in the low-expression group (*p* < 0.001) (Figure 7H). We further investigated the impact of TP53 mutation status on the relationship between *Ppih* expression and overall survival (OS) in HCC patients. As depicted in Figure 7, regardless of the TP53 mutation status, HCC patients with elevated *Ppih* expression exhibited a reduced survival rate. However, due to the limited sample size, the Kaplan-Meier survival analysis indicated that high *Ppih* expression did not significantly correlate with a worse prognosis in HCC patients with TP53 mutations (Figure 7J). Yet, it’s noteworthy that among the HCC patients with TP53 mutations, only 5.49% (5 out of 91) of those in the high *Ppih* expression bracket survived beyond 5 years. In contrast, in HCC patients lacking TP53 mutations, 14.29% (27 out of 189) from the high *Ppih* expression group survived past the 5-year mark.

**Figure 8:**
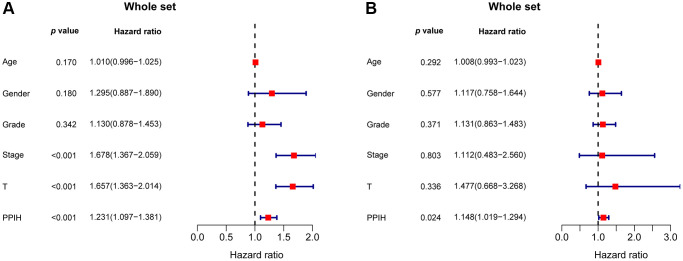
The forest plot shows the univariate (**A**) and multivariate (**B**) Cox regression analysis of the relationship between PPIH expression and OS among HCC patients.

Additionally, via data from the Kaplan-Meier plotter, we performed univariate and multivariate analyses on the clinical outcomes related to the expression of *Ppih* within different clinicopathological factors. Among patients with HCC with diverse clinicopathological factors, such as gender, race, sorafenib treatment, phase, grade, AJCC_T, alcohol intake, and hepatitis virus+/−, a univariate analysis found a significant correlation between mRNA expression for *Ppih* and OS (Table 4). mRNA expression for *Ppih* and OS among patients with HCC were proven to be substantially correlated in the multivariate analysis (Table 4). To analyze the effect of *Ppih* expression as well as other clinicopathological variables on survival, univariate and multivariate analyses via the Cox proportional hazard regression model were also performed on 349 HCC patients. According to Figure 8A, 8B, clinical stage (HR = 1.678; 95% CI, 1.367–2.059; *p* < 0.001), T stage (HR = 1.657; 95% CI, 1.363–2.014; *p* < 0.001) and *Ppih* expression levels were all substantially linked with poorer OS. In the multivariate analysis, high *Ppih* expression in particular was the only independent prognostic factor to be positively correlated with OS (HR = 1.148; 95% CI, 1.019–1.294; *p* = 0.024).

**Table 4:** Correlation of *Ppih* mRNA expression and clinical prognosis in HCC with different clinicopathological factors by Kaplan-Meier plotter.

**Clinicopathological characteristics**	**Overall survival (*n* = 364)**				
	**Univariate analysis**			**Multivariate analysis**	
	***n***	**Hazard ratio**	***p*-value**	**Hazard ratio**	***p*-value**
** All**	364			1.76 (1.24–2.49)	0.0012
**Gender:**					
Male	246	2.63 (1.68–4.1)	1e-05		
Female	118	1.42 (0.71–2.84)	0.32		
**Race:**					
White	181	1.43 (0.9–2.25)	0.12		
Black or African American	17	NA	NA		
Asian	155	2.92 (1.56–5.45)	0.00043		
**Sorafenib treatment:**					
Treated	29	4.71 (1.39–16.01)	0.0067		
**Stage:**					
1	170	1.83 (1–3.37)	0.048		
1+2	253	1.68 (1.04–2.71)	0.032		
2	83	1.98 (0.83–4.74)	0.12		
2+3	171	1.96 (1.23–3.14)	0.0043		
3	83	2.74 (1.48–5.07)	0.00085		
3+4	87	2.7 (1.49–4.88)	0.00068		
4	4	NA	NA		
**Grade:**					
1	55	2.29 (0.79–6.6)	0.12		
2	174	1.97 (1.18–3.28)	0.0079		
3	118	1.89 (1.04–3.44)	0.035		
4	12	NA	NA		
**AJCC_T:**					
1	180	1.78 (0.99–3.2)	0.049		
2	90	1.56 (0.75–3.25)	0.24		
3	78	2.75 (1.46–5.16)	0.0011		
4	13	NA	NA		
**Vascular invasion:**					
None	203	1.51 (0.9–2.55)	0.12		
Micro	90	0.68 (0.26–1.8)	0.43		
Macro	16	NA	NA		
**Alcohol consumption:**					
Yes	115	3.2 (1.64–6.21)	0.00031		
None	202	2.13 (1.12–4.05)	0.019		
**Hepatitis virus:**					
Yes	150	2.48 (1.3–4.75)	0.0044		
None	167	1.6 (1–2.56)	0.047		

Abbreviation: NA: Not available.

We conducted both univariate and multivariable Cox proportional hazard regression analyses to determine if the PPIH gene was independently linked to clinical outcome risks. For this purpose, we categorized HCC patients into TP53 mutated and non-mutated groups. The univariate Cox model showed that elevated *Ppih* expression was significantly correlated with poorer OS in TP53-mutated HCC. This was also the case for cancer pathological stage, tumor differentiation (Grade), and T stage (Supplementary Figure 1A). However, in the multivariate analysis, *Ppih* was not identified as an independent risk factor for unfavorable prognosis in HCC patients with TP53 mutations. We surmise that this might be due to the limited number of TP53-mutated HCC samples (Supplementary Figure 1B). In HCC patients without TP53 mutations, the univariate Cox model highlighted that age, tumor differentiation (Grade), cancer pathological stage, T stage, and *Ppih* expression were all strongly associated with OS (Supplementary Figure 1C). Further, the multivariate Cox model underscored that age, grade, and *Ppih* expression were all significantly tied to OS in these patients (Supplementary Figure 1D).

### *Ppih’s* biological interaction system

**Figure 9:**
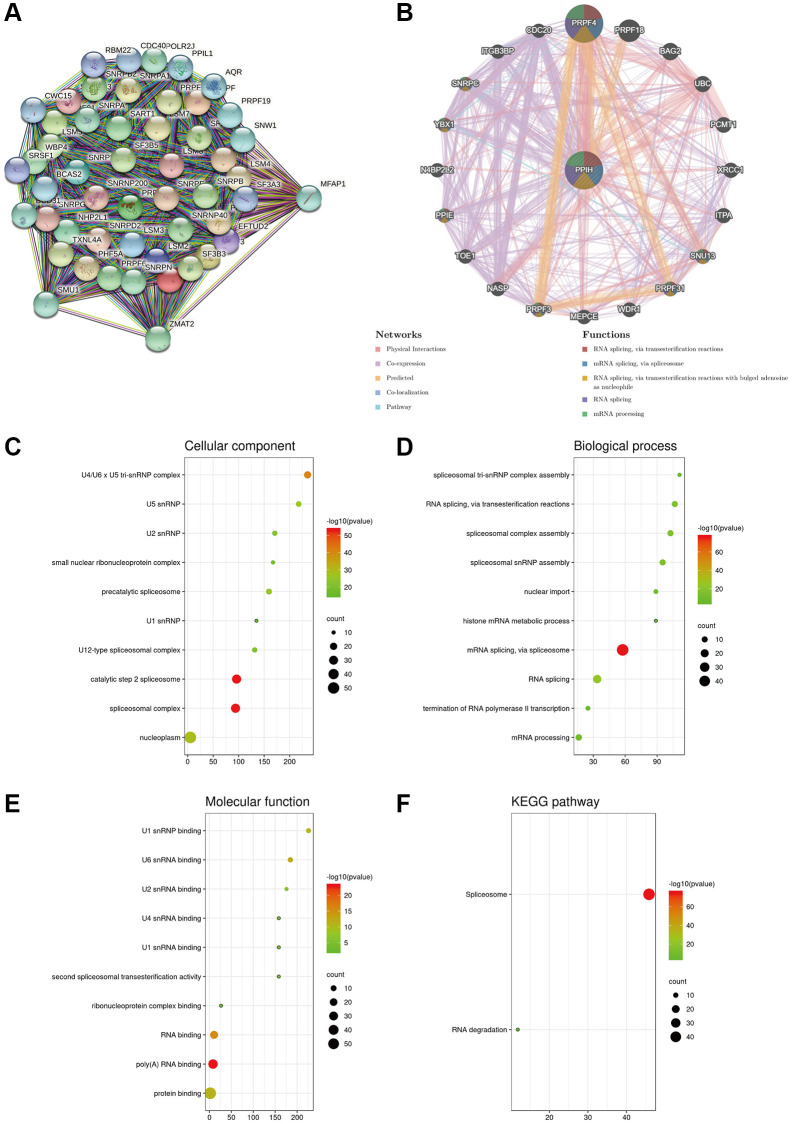
**Biological interaction network of PPIH and enrichment analysis of gene sets enriched in the target network of PPIH.** (**A**) Protein – protein interaction (PPI) network of PPIH (top 50) (STRING database). (**B**) PPI network (top 20) and functional analysis of gene sets enriched in the target network of PPIH. Colors of the network edges indicate the bioinformatics methods applied: physical interactions, coexpression, predicted, colocalization, and pathway. Colors of the network nodes indicate the biological functions of enriched gene sets (GeneMANIA database). (**C**–**E**) The GO functional enrichment analysis predicted three main functions of 65 genes enriched in the target network of PPIH, including cellular component, biological process, and molecular function. (**F**) KEGG pathway analysis (bioinformatics database).

A PPI system of *Ppih* (top 50) was created via the STRING data sources to better understand the molecular function of *Ppih* and the signaling pathways in which *Ppih* is engaged (Figure 9A). Twenty genes were also proven to be enriched in the *Ppih* system, according to the GeneMANIA assessment of the PPI system. RNA is spliced by transesterification reactions, mRNA is spliced by the spliceosome, and RNA is spliced by transesterification reactions with bulged adenosine as a nucleophile (Figure 9B). To determine the functional enrichment of these 65 interactors according to the STRING and GeneMANIA data sources, gene ontology (GO) and Kyoto Encyclopedia of Genes and Genomes (KEGG) studies were carried out via bioinformatics data sources (http://www.bioinformatics.com.cn/). The cellular component analysis revealed that these proteins were mostly found in the U4/U6 U5 tri-snRNP complex, in U5 snRNP, and in U2 snRNP, as depicted in Figure 9C–9E. The most prevalent biological processes were the construction of the spliceosomal tri-snRNP complex and RNA splicing via transesterification events. These proteins were primarily involved in the binding of the U1 snRNP and the U6 snRNA, according to an analysis of their molecular functions. The co-expressed genes were mostly engaged in the spliceosome and degradation of RNA, according to the KEGG pathway outcomes (Figure 9F).

### GSEA identification of signaling pathways connected to *Ppih*

**Figure 10:**
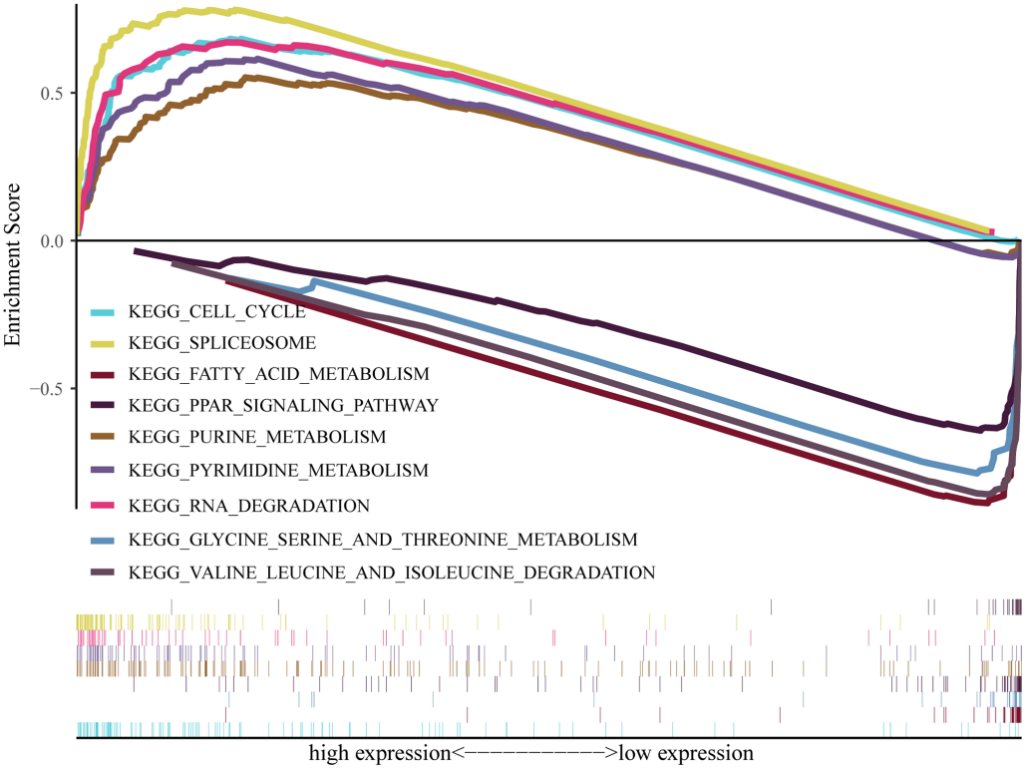
**Pathway enrichment analysis of PPIH.** Multiple-GSEA results showing pathways are differentially enriched in PPIH-related HCC. The cutoff criterion was set at *p* < 0.05 and FDR < 0.05 was considered statistically significant.

To conduct research on the role of *Ppih* and associated signaling pathways in HCC, we performed GSEA on the high and low *Ppih* expression datasets. The differentially expressed genes between high and low *Ppih* expression datasets that was used as input for GSEA in Supplementary Table 2 (logFCfilter = 1). The MSigDB collection was utilized for GSEA (c5.all.v7.5.1.symbols.gmt). With FDR and NOM score ps below 0.05 as cutoffs, we examined differentially enriched GO and KEGG pathways connected to the *Ppih* high expression phenotype (Table 5). The *Ppih* high expression phenotype was differentially enriched for pathways involved in the spliceosome, pyrimidine, purine, cell cycle, and degradation of RNA (Table 5, Figure 10). Gene sets involved in the degradation of valine, leucine, and isoleucine as well as the metabolism of fatty acids, glycine, serine, and threonine as well as the PPAR signaling pathway also showed differential enrichment in the low *Ppih* gene expression phenotype (Figure 10).

**Table 5:** Gene sets enriched in phenotype high.

**MSigDB collection**	**Gene set name**	**NES**	**NOM *p*-val**	**FDR *q*-val**
c5.all.v7.5.1.symbols.gmt	GOCC_CYTOSOLIC_RIBOSOME	2.25	0	0
	GOCC_CYTOSOLIC_LARGE_RIBOSOMAL_SUBUNIT	2.20	0	0
	GOCC_U2_TYPE_SPLICEOSOMAL_COMPLEX	2.15	0	0
	GOCC_PRECATALYTIC_SPLICEOSOME	2.13	0	0
	GOCC_CYTOSOLIC_SMALL_RIBOSOMAL_SUBUNIT	2.11	0	0
	GOBP_CYTOPLASMIC_TRANSLATION	2.10	0	0
	GOBP_RRNA_TRANSCRIPTION	2.03	0	0
	GOBP_RIBOSOMAL_LARGE_SUBUNIT_BIOGENESIS	2.01	0	0
	GOBP_RRNA_METABOLIC_PROCESS	2.00	0	0
	GOBP_RNA_SPLICING	1.98	0	0
	GOMF_STRUCTURAL_CONSTITUENT_OF_RIBOSOME	2.08	0	0
	GOMF_DNA_POLYMERASE_BINDING	2.01	0	0
	GOMF_NF_KAPPAB_BINDING	1.90	0	1.35E-05
	GOMF_THYROID_HORMONE_RECEPTOR_BINDING	1.90	0	1.46E-05
	GOMF_TELOMERASE_RNA_BINDING	1.89	0	2.33E-05
	KEGG_SPLICEOSOME	2.06	0	0.015
	KEGG_PYRIMIDINE_METABOLISM	1.99	0	0.0276
	KEGG_PURINE_METABOLISM	1.95	0	0.0302
	KEGG_CELL_CYCLE	1.90	0	0.0386
	KEGG_RNA_DEGRADATION	1.88	0	0.0354

Abbreviations: NES: normalized enrichment score; NOM: nominal; FDR: false discovery rate. Gene sets with NOM *p*-val < 0.05 and FDR *q*-val < 0.05 are considered as significant.

## DISCUSSION

A serious human cancer with an extremely low OS rate is HCC [22]. Based on yearly forecasts, the WTO predicts that more than 1 million patients will pass away from HCC by 2030 [23]. If HCC is detected early and treated well, patients may have a greater chance of surviving [24]. Only approximately 70% of HCC patients have alpha-fetoprotein (AFP) positivity, although AFP has long been utilized as a diagnostic for the early identification of HCC [25]. Additionally, owing to its limited sensitivity and significant background staining, *in situ* detection of AFP via IHC is presently unpopular (Figure 3C) [26, 27]. Accordingly, it is crucial to test novel HCC diagnostic and prognostic markers as soon as possible.

In recent years, a growing number of treatment targets and prognostic markers for HCC have been published and found thanks to advances in sequencing and omics, and the molecular process underlying HCC incidence and development has been more fully understood. For instance, Jiang et al. [3] examined the proteome profiling of early-phase HCCs tied to the HBV and discovered that a subpopulation of HCCs defined by disturbed cholesterol homeostasis and overexpression of sterol O-acyltransferase 1 (SOAT1) was associated with negative prognosis. In fact, in client-derived xenograft models, downregulating SOAT1 or treatment with the SOAT1 inhibitor avasimibe substantially lowers the size of tumors overexpressing SOAT1 and effectively inhibits HCC proliferation and migration [3].

To shed light on the novel molecular roles of *Ppih* and its link to cancer progression, we employed a range of bioinformatics techniques complemented by clinical specimen validation. Firstly, we examined the transcriptional sequencing data from more than 1000 clinical samples spread over six different geographic areas in the GEO and TCGA data repositories. *Ppih* mRNA levels are substantially higher in HCC than in healthy liver tissue, according to ethnic HCC studies [28, 29]. Furthermore, the fold change was comparable across all HCC studies (Figure 2A–2F). HCC is a heterogeneous disease with multiple etiologies. As shown in Figure 2, patients in Mas and Wurmbach datasets are Hepatitis C (Hep-C) HCCs (Figure 2E, 2F), while Roessler dataset is predominantly Hepatitis B (Hep-B) HCCs (Figure 2D), while TCGA is of mixed etiology (Figure 2A, 2B). Moreover, univariate analysis showed a significant relationship between *Ppih* mRNA expression and OS in terms of clinicopathological parameters of alcohol intake and hepatitis virus+/− in HCC patients (Table 4). Similarly, the mRNA expression of *Ppih* was significantly associated with the history of liver cirrhosis of HBV-related HCC patients (Supplementary Table 1). Moreover, more than 70% of the 11 HCC specimens analyzed by IHC (Figure 3) had a history of cirrhosis and were positive for HBV (Supplementary Table 3). Previous studies have shown that chronic Hepatitis B and C infections is the key factor driving the progression of liver cirrhosis to HCC [30, 31]. Interestingly, non-immunosuppressive cyclophilin inhibitors have the potential to treat hepatitis virus-induced liver cirrhosis and HCC by targeting cyclophilin [32]. Therefore, it is necessary to expand the specimen size in the future to explore the expression of PPIH in different etiologies (Hep-B, Hep-C, alcoholic and NAFLD). In addition, it is also an interesting topic to explore whether the high expression of PPIH can participate in the occurrence and development of viral cirrhosis to HCC by affecting the immune microenvironment and promoting malignant proliferation. Furthermore, *Ppih’s* mRNA expression rose as cancers worsened (Figures 4B, 4G, and Figure 5A–5C). The elevated expression of *Ppih* mRNA in HCC patients with TP53 mutations is notably more significant (*p* < 0.001) compared to those without the mutation (Figure 4I). Similarly, when compared to normal liver tissues, PPIH mRNA expression levels are higher in TP53-mutated HCC than in non-TP53-mutated HCC (Figure 6). This expression level has a positive correlation with the malignancy of tumors possessing TP53 mutations (Figure 5D–5F), whereas no such correlation was observed in tumors without TP53 mutations (Figure 5G–5I). Lee et al. [7] highlighted that CypA is transcriptionally regulated by two pivotal transcription factors involved in cancer development: TP53 and hypoxia-inducible factor-1. Earlier studies have demonstrated that CypA overexpression fosters resistance to both hypoxia and chemotherapeutic agents like cisplatin in HCC [33]. When combined with cisplatin, the fungal cyclic peptide cyclosporin A (CsA) or the fungal macrolide compound sanglifehrin A (SFA) can synergistically enhance apoptotic cell death in HCC cells. This enhanced effect stems from the inhibition of CypA activity by CsA and SFA. Remarkably, the combined effect of CsA or SFA with cisplatin remained evident even in TP53-defective Hep3B cells [33]. These findings suggest that wild-type TP53 may act as a transcriptional suppressor that partially modulates PPIH mRNA expression. Moreover, TP53 mutations appear to bolster PPIH mRNA overexpression, potentially accelerating the malignancy of HCC. Nonetheless, irrespective of TP53 mutation status, PPIH overexpression stands as an unfavorable prognostic indicator for HCC patients (Figure 7I–7K). Additionally, *Ppih* mRNA was also substantially overexpressed among patients with HCC who tested positive for the HBV (*n* = 158), supporting this (Figure 2I). IHC outcomes showed that the *Ppih* protein expression trend in HCC specimens was consistent with its mRNA level (Figure 3). Most importantly, clinically significant *Ppih* genetic alterations (Figure 7A–7C) or high *Ppih* expression (refer to Figure 7D–7H) were linked to poor survival among patients with HCC. These findings imply that *Ppih* may serve as a biomarker for the early detection and prediction of HCC.

Hepatocyte paraffin-1 (HepPar-1), glypican-3 and Ki-67 are useful diagnostic markers for HCC, but the expression of these 3 markers has also been reported in nonhepatocellular tumors [34, 35]. Moreover, distinction of liver metastases from HCC is also a challenging diagnostic task [36]. McKnight et al. [34] reported that arginase-1 (Arg-1) positivity was demonstrated in 37 of 44 (84.1%) cases of HCC, compared with 32 of 44 cases (72.7%) and 25 of 44 cases (56.8%) for HepPar-1 and glypican-3, respectively. Consistent with this, Timek et al. [36] demonstrated that Arg-1 is the most specific marker in differentiating a non-HCC from HCC compared with HepPar-1 and glypican-3. They all recommend to use of Arg-1 with HepPar-1 and glypican-3 as a panel in distinguishing HCC from metastatic carcinoma [34, 36]. In this study, IHC results showed that 100% (11/11), 100% (11/11), 81.8% (9/11) and 90.9% (10/11) of HCC cases were positive for PPIH, Ki-67, HepPar-1 and glypican-3, respectively (Figure 3). Therefore, it is necessary to further study whether PPIH, like Arg-1, can be used in combination with common markers such as HepPar-1 and glypican-3 to improve the specificity of HCC diagnosis and provide scientific basis for the hierarchical diagnosis and treatment of HCC.

According to Li et al. [20], 54 RNA-binding proteins, including *Ppih*, were substantially connected to the prognosis of HCC related to the HBV. This finding is consistent with the findings of the investigation. The one-, three-, and five-year overall survival of HCC customers could also be predicted more accurately via an 11-RBP model, such as *Ppih* [20]. Seven RBPs, including *Ppih*, were also discovered by Li et al. [19] to be useful as stomach cancer prognostic biomarkers. In addition, Gao et al. [37] discovered that ten genes, including *Ppih*, were identified as hub susceptibility genes for COVID-19 in lung adenocarcinoma customers, and the hub susceptibility genes were substantially linked to the infiltration of many immune cells. This finding indicates that further research is needed to ascertain whether *Ppih* affects immune cell infiltration in malignancies and promotes the progression of HCC.

*Ppih* was implicated in the control of the spliceosome and degradation of RNA, according to further analysis of the gene regulatory system in HCC (Figures 9F and 10). These outcomes are somewhat in line with those of other research [6, 16]. Pre-mRNA processing Factor 3 (PRPF3), PRPF31, and PRPF4 are the intersections of two PPI systems, as depicted in Figure 9A, 9B. High PRPF3 expression has been linked to immune infiltration and prognostication in HCC according to research by Liu et al. [38]. HCC patients with greater PRPF4 expression than those with lower expression had worse prognoses. Tam et al. [39] reported that SM08502, a novel small molecule in clinical development for the treatment of solid cancers, inhibited serine- and arginine-rich splicing factor phosphorylation and disrupted spliceosome activity. Xu et al. [40] demonstrated that many genes of the spliceosome pathway are upregulated in HCC. In conclusion, our data show the crucial role of *Ppih* in the development and spread of HCC. Nevertheless, additional investigation is required to determine how the spliceosome pathway regulates HCC deteriorates when *Ppih* is produced abnormally.

N6-methyladenosine (m6A) modification, the most abundant internal methylation of messenger RNAs (mRNAs) of most eukaryotes, is critically implicated in RNA processing [41]. In mammalian cells, m6A modification is a reversible process regulated by m6A WERs (writers, erasers and readers) [42]. Numerous studies have demonstrated that m6A modification exerts significant and comprehensive effects on diverse biological regulatory processes, including transcription splicing, RNA stability, translation efficiency, and cell fate determination [42]. Notably, several studies have pointed out that hepatocarcinogenesis is correlated with abnormal m6A modifications [41–43]. For instance, m6A demethylase FTO (fat mass and obesity-associated protein) promotes HCC tumorigenesis via mediating PKM2 demethylation [44]. GSEA revealed that the KEGG pathways associated with the spliceosome, pyrimidine metabolism, purine metabolism, RNA degradation and cell cycle were all significantly enriched in the *Ppih* high-expression phenotype (Figure 10). Moreover, TP53 may only partially inhibit PPIH mRNA expression (Figures 5–7). Therefore, we hypothesized that significant reduction of m6A modification level in *Ppih* mRNA may be also the main factor leading to the significantly high expression of *Ppih* in HCC (Figure 7A). Further studies are needed to confirm this hypothesis.

## CONCLUSIONS

In conclusion, our research found that a negative prognosis among patients with HCC is correlated with increased *Ppih* expression regardless of TP53 mutation. *Ppih* and the genes it coexpresses may help HCC develop and spread by upregulating the spliceosome pathway. These outcomes suggest that *Ppih* may serve as a target of therapeutic intervention, as well as a diagnostic and predictive biomarker in HCC. Nevertheless, additional investigation is required to validate the contribution of *Ppih’s* aberrant expression to the transition from cirrhosis to HCC.

## MATERIALS AND METHODS

### Bioinformatics analysis

Oncomine, DriverDBV3, GEPIA2, UALCAN, and cBioPortal database analysis and PPI network analysis were performed as previously described [5, 45].

### Collecting data for clinical and genetic purposes

The medical evidence for 377 HCC and the gene expression profiles for 465 instances and 407 HCC patients plus 58 nearby normal tissues (Workflow Type: HTSeq-FPKM) were retrieved from TCGA Genomics Data Commons portal website (https://portal.gdc.cancer.gov/repository).

### Evaluation of *Ppih* expression and survival

The data of original RNA-Seq gene expression that was downloaded were sorted and combined via the programming language Perl. The R software (V3.5.1; https://cran-archive.r-project.org/bin/windows/base/old/3.5.1/) and beeswarm packages were utilized to inspect the various *Ppih* expressions. Then, survival data were extracted from clinical data via the Perl programming language. The Kaplan-Meier survival curve was then produced via the survival package of the R program to analyze the prognostication-related *Ppih* scores. Additionally, univariate and multivariate Cox regression analyses were performed and shown via the coxph and ggforest commands as well as the survminer and survival packages of the R program.

### Clinical samples and IHC staining

At the Second Affiliated Hospital of Guizhou Medical University, 11 patients were diagnosed with HCC between June 15, 2017, and March 21, 2021. Detailed clinical data and the outcomes of all laboratory tests were tracked and recorded for every client. As previously stated [46], the inclusion and exclusion criteria were utilized. All participants provided written informed permission that was authorized by the ethics committees of the Second Affiliated Hospital of Guizhou Medical University. We provide detailed clinical information of these specimens in Supplementary Table 3. Minor adjustments were made to the previously published methods for IHC staining [46]. The primary antibody against PPIH (1:100), PBS as a negative control, and tonsil tissue as a positive control were all incubated on paraffin sections at 4°C overnight. Next, biotin-labeled goat anti-rabbit IgG was added, and after 30 minutes at room temperature, the streptavidin-biotin-peroxidase complex was added. IHC staining was accomplished via 3,3′-diaminobenzidine tetrahydrochloride. Hematoxylin was utilized as a counterstain, and DAB was dehydrated in ethanol, cleaned in xylene, and fixed thereafter. The same procedure we previously discussed was utilized to evaluate the IHC staining outcomes [47].

### Gene set enrichment analysis

According to the median *Ppih* expression, GSEA Version 4.1.0 was utilized to conduct research on the probable mechanism of *Ppih* and revealed statistically significant differential expression between the high and low expression groups. For every assessment, the gene set configurations were repeated 1,000 times, and *Ppih* expression levels were utilized as phenotypic labels. The substantially upregulated gene sets were examined according to the normalized enrichment score. Gene sets were then deemed to be substantially upregulated if their normalized *p*-score and false discovery rate (FDR) were both below 0.05.

### Statistical assessment

Mann-Whitney *U* testing was utilized to ascertain whether there was a difference in the expression of *Ppih* between HCC specimens and nearby specimens under typical conditions. Kruskal-Wallis testing was utilized to inspect the *Ppih* differences between various groups. Logistic regression and Wilcoxon ranking testing (two continuous independent samples) were utilized to inspect the association between *Ppih* and the clinical features of HCC. Clinically insufficient client information was intentionally left out. The effects of *Ppih* and clinical traits on OS were then compared via Cox regression analysis. R software (Edition 3.5.1) and SPSS 21.0 (IBM, Armonk, NY, USA) were utilized for all statistical analyses, and a *p* score below 0.05 was regarded as statistically significant.

### Statement of data accessibility

The STRING, GeneMANIA, LinkedOmics, DAVID, and bioinformatics data sources were utilized to conduct research on the probable regulatory mechanism of *Ppih* in the progression of HCC.

## Supplementary Materials

Supplementary Figure 1

Supplementary Tables 1 and 3

Supplementary Table 2
