# Supplementary material for: PPIH gene regulation system and its prognostic significance in hepatocellular carcinoma: a comprehensive analysis
Source: Aging (Albany NY). 2023 Oct 23;15(20):11448–70. doi: 10.18632/aging.205134 (PMC10637785; doi:10.18632/aging.205134)
Supplement: Supplementary Figure 1 [file aging-15-205134-s001.pdf]

SUPPLEMENTARY FIGURE

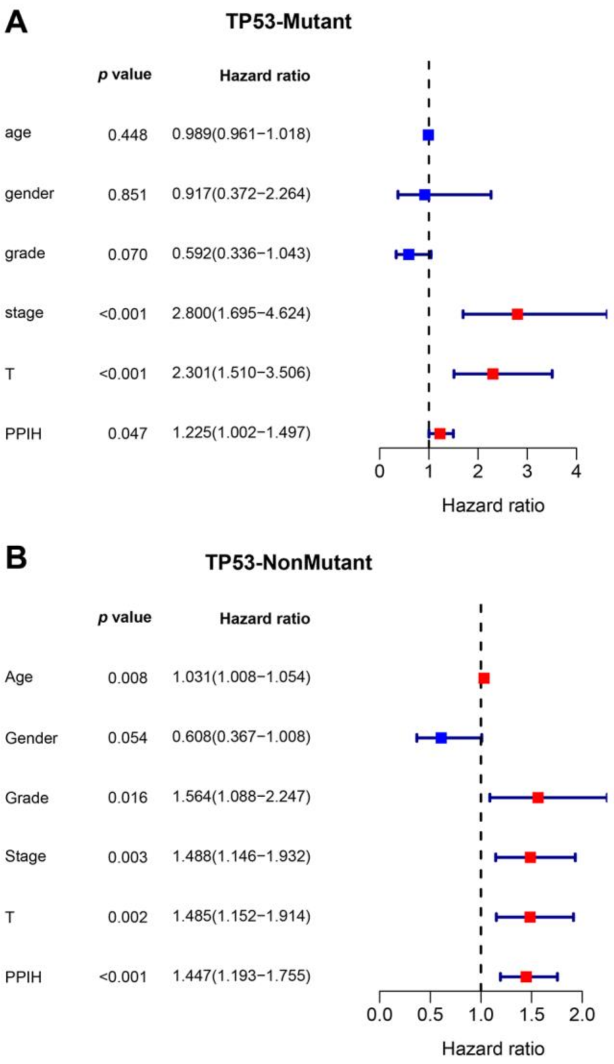

Supplementary Figure 1. Univariate (**A**, **B**) and Multivariate (**C**, **D**) Cox regression analyses of clinicopathologic variables and PPIH of HCC patients with or without TP53 mutations based on TCGA database.
