# Supplementary material for: PPIH gene regulation system and its prognostic significance in hepatocellular carcinoma: a comprehensive analysis
Source: Aging (Albany NY). 2023 Oct 23;15(20):11448–70. doi: 10.18632/aging.205134 (PMC10637785; doi:10.18632/aging.205134)
Supplement: Supplementary Tables 1 and 3 [file aging-15-205134-s002.pdf]

SUPPLEMENTARY TABLES

Supplementary Table 1. Association between *Ppih* mRNA expression and various clinicopathological factors of HBV-related HCC patients.

Variables	*Ppih* mRNA expression		*p* value	Multivariate analysis#		*p* value
	Low (*n* = 79)	High (*n* = 79)		OR	95%	
**Age, years**						
≤54	42	43	0.87	1	Reference	
>54	37	36		0.237	0.040–1.403	0.113
**Gender**						
Male	63	64	0.84	1	Reference	
Female	16	15		0.128	0.524–24.94	0.176
**Preoperative AFP (ng/ml)**						
≤200	46	41	0.48	1	Reference	
>200	33	37		3.615	0.312–1.461	0.192
**TB (μmol/L)**						
≤20	75	76	1.00	1	Reference	
>20	4	3		0.223	0.002–22.02	0.522
**ALT (U/L)**						
≤50	55	56	0.86	1	Reference	
>50	24	23		0.696	0.089–4.931	0.717
**γ-GT (U/L)**						
≤60	43	39	0.52	1	Reference	
>60	36	40		11.495	0.946–139.7	0.055
**HBcAb**						
No	5	4	1.00	1	Reference	
Yes	74	75		1.942	0.018–205.2	0.780
**Tumor size (cm)**						
≤5	39	37	0.75	1	Reference	
>5	40	42		0.290	0.034–2.462	0257
**Tumor number**						
Single	57	58	0.75	1	Reference	
Multiple	22	20		1.226	0.523–2.871	0.639
**Tumor thrombus**						
No	65	57	0.13	1	Reference	
Yes	14	22		0.288	0.038–2.210	0.231
**Tumor encapsulation**						
No	21	23	0.80	1	Reference	
Yes	55	55		2.634	0.416–16.68	0.304
**Tumor differentiation**						
High	29	34	0.45	1	Reference	
Low	49	45		2.978	0.498–17.83	0.232
**History of liver cirrhosis**						
No	19	28	0.12	1	Reference	
Yes	60	51		0.028	0.001–0.594	**0.022**

Pearson Chi-Square test was used to analyze the relationship between *Ppih* mRNA expression and clinicopathological characteristics. Bold font means statistical significance $p < 0.05$. #Logistics regression was used for multivariate analysis.

Supplementary Table 3. The clinical information of 11 patients with HCC was analyzed by IHC.

Case ID	Patient Na	Accession	Gender	Age
1	*	*	Male	61
2	*	*	Male	54
3	*	*	Male	56
4	*	*	Male	45
5	*	*	Male	44
6	*	*	Male	65
7	*	*	Male	68
8	*	*	Female	44
9	*	*	Female	48
10	*	*	Male	63
11	*	*	Male	51
**Pathologic**	**Tumor differentiation**	**Medical H**	**History of**	**Pathologic**
HCC	3	1	1	HCC
HCC	1	0	0	HCC
HCC	2	1	1	HCC
HCC	3	1	1	HCC
HCC	3	1	1	HCC
HCC	3	1	1	HCC
HCC	1	1	1	HCC
HCC	3	1	1	HCC
HCC	2	1	1	HCC
HCC	4	0	0	HCC
HCC	4	0	0	HCC
HCC	3	1	1	HCC
HCC	1	0	0	HCC
**HBsAg**	**ALT**	**AFP**	**CEA**	**AST**
1	31	4209.45	1.64	47
0	80	7200	1.48	118
1	50	29281.9	0.5	158
1	32	33242.8	0.77	37
1	49	55.9	3.87	71
1	48	12.45	1.39	111
1	90	1020	1.88	339
1	27	180.1	0.63	28
1	20	114517	1.39	50
0	23	1133	2.43	50
0	94	3348.1	2.59	111
