# Supplementary material for: PPIH gene regulation system and its prognostic significance in hepatocellular carcinoma: a comprehensive analysis
Source: Aging (Albany NY). 2023 Oct 23;15(20):11448–70. doi: 10.18632/aging.205134 (PMC10637785; doi:10.18632/aging.205134)
Supplement: Supplementary Table 2 [file aging-15-205134-s003.pdf]

Supplementary Table 2. The differentially expressed genes between high and low Ppih expression datasets in HCC that was used as input for GSEA. The cutoff criterion was set at $p < 0.05$ and logFCfilter = 1 was considered statistically significant.

gene	conMean	treatMean	logFC	pValue
TARDBP	9.758133	13.81634	4.058205	9.34E-42
PPIH	1.147248	3.060951	1.913703	1.82E-40
ARPC3	29.62318	53.67535	24.05217	1.95E-37
UBE2I	2.459423	4.833489	2.374066	2.49E-36
KTI12	1.396862	2.562219	1.165358	8.99E-35
LAMTOR5	12.21103	21.59337	9.382342	1.42E-34
COPZ1	29.89295	46.92271	17.02976	1.45E-34
DAZAP1	7.559665	11.84448	4.284816	1.85E-34
RNF220	2.847337	4.839217	1.991879	9.28E-34
ILKAP	3.517062	5.68562	2.168558	2.31E-33
ISY1	2.017659	4.067558	2.049899	4.10E-33
ZCCHC17	5.561532	9.730653	4.169122	4.94E-33
MCRS1	10.61879	18.12954	7.510747	5.49E-33
TMEM234	0.85098	1.926979	1.075999	5.98E-33
CHD4	1.682412	3.350596	1.668184	8.13E-33
RBM10	14.2353	20.70896	6.473654	8.88E-33
FKBP1A	16.00516	29.50628	13.50112	1.19E-32
LCMT1	4.544598	9.158676	4.614078	1.26E-32
RALY	11.82812	21.62357	9.795451	1.31E-32
C18orf21	4.531282	7.533637	3.002355	3.72E-32
NABP2	10.57508	18.71427	8.13919	4.41E-32
CCDC59	3.644227	5.615315	1.971088	8.93E-32
PFDN4	5.127644	10.64122	5.513579	1.22E-31
SNRPB2	12.32069	20.10575	7.78506	1.24E-31
ADPRS	11.83132	21.54189	9.710576	1.98E-31
CD2BP2	11.04341	19.59595	8.552533	2.39E-31
MRPL17	10.46323	19.05467	8.591435	2.39E-31
ZBTB8OS	3.783605	6.404546	2.620941	3.34E-31
PPIE	2.262489	3.929904	1.667415	3.95E-31
SNRPD1	5.021965	10.25957	5.237605	4.05E-31
MAGOH	15.3648	24.34781	8.983007	4.97E-31
SNRPB	56.99302	119.1508	62.15776	1.18E-30
ARFGAP1	8.348345	14.34935	6.001	1.55E-30
CCDC137	7.326834	13.99843	6.6716	2.34E-30
MAPKAPK	2.239484	4.507597	2.268112	2.36E-30
ITGB3BP	1.124292	2.306824	1.182533	3.75E-30
HSBP1	5.169516	8.631076	3.46156	3.99E-30
POLR2H	10.0577	17.60951	7.551819	5.37E-30
SF3A3	11.75019	19.10083	7.350641	6.98E-30
CERS5	2.30026	4.525853	2.225594	7.02E-30
PA2G4	16.65965	28.85685	12.19721	7.08E-30
SNRPA	12.13146	24.51602	12.38457	7.61E-30
ABCF2	1.823252	3.429736	1.606484	7.67E-30
SFPQ	15.58098	27.04535	11.46436	9.60E-30
DGUOK	10.46798	18.35641	7.888431	1.06E-29
TPD52L2	13.07633	24.57167	11.49534	1.12E-29
MFSD5	8.456393	15.03437	6.577981	1.37E-29
NACA	25.17279	41.68919	16.5164	1.49E-29
RNF25	8.341658	12.54278	4.201126	1.97E-29
STX4	9.155455	14.31426	5.158803	1.98E-29
KHDRBS1	22.3899	36.16788	13.77798	2.04E-29
SMYD5	5.84167	10.60267	4.761	2.59E-29
C14orf119	7.9118	12.03985	4.128052	2.69E-29
NUTF2	13.64458	25.18506	11.54049	3.32E-29
DYNLL1	37.78799	57.84115	20.05316	4.46E-29
NXT1	8.363308	15.0044	6.641093	5.09E-29
BTF3L4	3.324148	6.19894	2.874792	5.25E-29

HSPB11	5.374337	9.106912	3.732575	8.43E-29
RNPS1	6.545733	11.72435	5.178614	8.47E-29
ANAPC7	4.323713	7.182124	2.858411	1.03E-28
SIRT6	4.711753	8.707979	3.996227	1.07E-28
NFYC	6.096932	9.254864	3.157932	1.15E-28
GSK3A	4.190148	7.083929	2.893781	1.21E-28
CLTA	37.56131	63.24859	25.68728	1.28E-28
RBMX2	4.54221	7.58089	3.038679	1.31E-28
TPRKB	6.465541	10.05458	3.589035	1.37E-28
PSMA4	12.77477	18.61277	5.837994	1.49E-28
COMMD7	10.83024	19.7965	8.966264	1.85E-28
MRGBP	5.634734	9.578518	3.943783	2.18E-28
CFL1	56.32628	94.14068	37.8144	2.19E-28
SRSF2	19.58777	30.18869	10.60092	2.22E-28
CCDC43	2.087476	3.960534	1.873058	2.32E-28
SNRPG	14.75011	23.35787	8.607762	2.69E-28
ARPC5L	7.686377	13.32451	5.63813	2.77E-28
MED8	10.80301	16.391	5.587994	3.78E-28
ATP5MC2	48.19584	82.04505	33.84921	4.04E-28
TRAPPC3	10.41785	15.30482	4.886972	4.10E-28
PIGU	8.302622	14.33963	6.037003	4.35E-28
CENPH	1.396065	3.352272	1.956208	7.63E-28
NASP	5.924845	11.53129	5.606448	8.23E-28
NUP85	5.546277	9.269813	3.723536	8.44E-28
DNTTIP1	9.315635	14.81622	5.500582	8.95E-28
SNRPF	9.327664	17.3018	7.974141	9.78E-28
HSD17B6	237.7673	91.96777	-145.8	1.23E-27
ITPA	17.13832	31.7852	14.64688	1.51E-27
MIR1244-	2.241954	4.724358	2.482404	1.61E-27
COPS7B	3.39017	5.728106	2.337936	2.00E-27
CHMP4B	50.58066	93.34545	42.76479	2.44E-27
APOB	377.2743	200.403	-176.871	2.44E-27
AC026401	1.941319	5.373404	3.432085	2.49E-27
SMPD2	2.822412	5.05849	2.236078	2.55E-27
H2AZ1	24.36116	49.81965	25.45849	3.04E-27
RPS8	163.93	355.1493	191.2193	3.19E-27
PDRG1	8.17214	13.74656	5.57442	4.23E-27
GAR1	5.004528	8.272512	3.267984	4.30E-27
ASB6	2.487909	4.31231	1.824401	4.85E-27
BCL7C	2.533675	5.084759	2.551083	4.95E-27
RNF7	10.19714	15.40719	5.210051	6.52E-27
SMUG1	3.959251	6.216911	2.257661	7.34E-27
SAP30BP	5.087198	8.029824	2.942626	8.33E-27
CPN2	97.72367	43.80703	-53.9166	8.44E-27
RAD51	0.688883	1.993571	1.304688	1.22E-26
PYM1	11.89679	16.78505	4.888257	1.39E-26
RP9	2.478418	4.139641	1.661223	1.42E-26
PCBP2	14.25498	21.04293	6.787957	1.62E-26
CD302	3.03639	1.285505	-1.75088	1.64E-26
ACD	2.929479	5.5101	2.580621	1.70E-26
C1orf52	2.152655	3.752067	1.599413	1.90E-26
ATP6V1F	38.82581	72.84308	34.01727	1.90E-26
GNL2	6.628855	10.72385	4.094995	2.11E-26
ABAT	35.63921	15.1688	-20.4704	2.38E-26
FUS	16.30986	28.13288	11.82302	2.57E-26
PPP4C	27.71851	42.67033	14.95181	2.62E-26
EIPR1	2.720387	4.695447	1.97506	2.62E-26
BRIX1	3.181571	5.73321	2.551639	3.21E-26

DTYMK	8.08236	16.76608	8.683722	3.39E-26
CEP131	2.703976	5.507528	2.803552	3.81E-26
CLIC1	69.93899	160.3728	90.43379	3.96E-26
ARPC4	15.82899	28.85291	13.02392	4.16E-26
UTP11	5.018799	7.769822	2.751024	4.77E-26
RP9P	1.118356	2.949987	1.831631	4.81E-26
SUMO2	21.40646	32.6918	11.28534	5.39E-26
HDDC2	1.96135	4.172223	2.210873	5.78E-26
METTL1	4.564356	7.883614	3.319258	6.23E-26
RFC4	3.958682	8.30086	4.342178	6.47E-26
EIF2B5	5.459057	7.731263	2.272206	7.01E-26
MRPL47	17.15595	28.46186	11.30591	7.42E-26
RRAGC	2.393821	4.316432	1.922611	7.59E-26
EEF1E1	1.488065	3.494348	2.006283	7.75E-26
SRSF3	17.61921	26.4368	8.817588	7.76E-26
ANP32A	11.89639	17.57645	5.680063	8.60E-26
DTNBP1	2.89543	5.699693	2.804263	9.17E-26
RBX1	10.6413	16.79677	6.155466	9.44E-26
URM1	10.53555	17.28968	6.754127	9.63E-26
PPT1	13.32465	27.42638	14.10172	9.71E-26
UQCRH	56.90923	102.5848	45.67553	1.01E-25
TRAF2	6.017836	10.96799	4.950152	1.30E-25
TRNAU1A	2.101243	3.561652	1.460409	1.86E-25
KIF22	3.776996	7.214758	3.437762	1.92E-25
GLTP	7.322568	11.82982	4.507252	1.96E-25
UBE2T	3.726366	8.976489	5.250123	2.30E-25
NUP43	3.92931	6.839506	2.910196	2.39E-25
EXOSC1	4.482536	6.773967	2.291431	2.44E-25
DCTN5	1.971538	3.718698	1.74716	2.45E-25
GIT1	4.503863	9.265051	4.761188	2.48E-25
ZSWIM1	2.546586	4.241981	1.695395	2.51E-25
UBE2M	26.51313	45.33651	18.82338	2.56E-25
SRRT	13.65585	20.99908	7.343225	2.82E-25
LSM7	14.17899	27.46213	13.28314	2.93E-25
JMJD6	3.172981	5.483086	2.310105	2.95E-25
DMAP1	4.949308	8.160384	3.211076	3.02E-25
TCOF1	2.548743	4.74525	2.196507	3.09E-25
BRMS1	14.35338	24.75779	10.40441	3.25E-25
GTF3C6	28.09449	54.00165	25.90716	3.37E-25
C1orf35	3.777652	6.414381	2.63673	3.37E-25
PRPF38A	3.966635	6.889573	2.922938	3.46E-25
DMGDH	22.15515	8.125915	-14.0292	3.60E-25
ERI3	12.98055	21.25928	8.278729	4.31E-25
PQBP1	15.67826	25.68269	10.00443	4.99E-25
DPH7	2.098679	4.006713	1.908033	5.23E-25
PSMD13	20.94797	31.52786	10.57989	5.88E-25
ARL16	5.514619	10.9201	5.405486	7.16E-25
CDT1	2.786157	8.448214	5.662057	7.17E-25
RPP38	2.903433	4.352857	1.449425	7.32E-25
ZSWIM9	1.79013	2.895907	1.105778	7.75E-25
UBE2J2	5.967465	9.40919	3.441725	7.82E-25
ATG4B	5.600801	8.858471	3.257669	7.92E-25
PPM1G	21.05004	32.62376	11.57372	8.06E-25
ALYREF	18.15809	38.19999	20.0419	8.30E-25
CBX3	17.70383	28.9465	11.24267	8.44E-25
FUNDC1	5.11796	9.375671	4.257712	9.17E-25
DNTTIP2	4.903906	7.579802	2.675896	1.07E-24
MED27	2.032246	3.522836	1.490591	1.09E-24

PTTG1	4.780399	15.03348	10.25308	1.24E-24
THAP11	6.624509	10.32464	3.700135	1.29E-24
CPB2	89.11945	45.83369	-43.2858	1.32E-24
TIMM17B	12.12105	19.92182	7.800776	1.32E-24
EIF2S2	25.26967	37.80612	12.53645	1.38E-24
POLR2G	17.75127	27.27969	9.528416	1.63E-24
HRAS	9.51202	19.99197	10.47995	1.65E-24
CKS1B	8.270873	15.75117	7.480301	1.68E-24
ATIC	16.07115	28.02892	11.95777	1.68E-24
PPCDC	2.002367	3.645616	1.64325	1.69E-24
DCTN2	6.129352	9.936152	3.8068	1.77E-24
USF1	19.77446	30.71008	10.93562	1.85E-24
MIR1244-:	7.612133	18.60289	10.99075	1.87E-24
TRAIP	0.738966	2.010821	1.271855	1.90E-24
MACROH2	3.971192	6.837928	2.866736	1.91E-24
SNF8	5.50227	9.302097	3.799827	2.20E-24
ZMYND19	5.135627	8.621424	3.485797	2.20E-24
MANBAL	17.23351	26.8621	9.628583	2.40E-24
THOC6	8.03368	13.87346	5.839781	2.40E-24
COL18A1	95.01661	55.37404	-39.6426	2.68E-24
RAC1	54.20976	88.9612	34.75144	2.74E-24
HAO1	125.3717	57.7363	-67.6354	2.75E-24
CZIB	5.191457	7.568097	2.37664	2.84E-24
MITD1	2.14292	3.653985	1.511065	2.90E-24
SLC2A2	145.867	61.25748	-84.6095	3.04E-24
EHHADH	53.47175	22.35459	-31.1172	3.08E-24
EDC3	3.294824	5.002245	1.707421	3.64E-24
RPL5	125.6807	234.6078	108.9271	3.78E-24
COMMD3	3.109662	5.038785	1.929123	3.89E-24
GTF2A2	7.476035	10.55627	3.080236	4.11E-24
MAGOHB	2.11579	3.321179	1.205389	4.21E-24
CAPN10	1.351545	2.472131	1.120586	4.68E-24
CDK16	6.273307	12.28366	6.010349	4.79E-24
SERPIND1	473.9358	245.5475	-228.388	4.95E-24
ATRAID	25.60666	39.88061	14.27395	5.34E-24
PPIA	39.5861	59.45593	19.86984	5.37E-24
PPP1R14B	22.64654	47.81219	25.16565	6.05E-24
HROB	0.733503	1.843604	1.110101	6.42E-24
PRR13	12.52308	21.81709	9.294005	7.32E-24
UBE2L3	14.86399	22.82757	7.963589	7.39E-24
CHASERR	2.019697	3.844235	1.824538	7.70E-24
ANKS3	1.105785	2.508425	1.40264	7.85E-24
PPP1R7	12.44705	17.47544	5.028391	8.25E-24
RBM22	8.792309	12.47902	3.68671	8.81E-24
TBC1D10E	3.187683	5.88166	2.693977	8.94E-24
PPP2R1A	21.33241	36.78316	15.45075	8.97E-24
SNU13	25.78626	39.2718	13.48554	1.00E-23
HDAC1	16.23326	27.35885	11.12559	1.06E-23
HNRNPD	13.49117	20.95596	7.464789	1.07E-23
PKMYT1	0.699043	1.917423	1.218379	1.07E-23
RPL35A	84.82754	164.4159	79.58832	1.08E-23
ZNF207	4.016675	6.047157	2.030482	1.09E-23
UBE2D2	14.92755	20.84123	5.913685	1.11E-23
HNRNPUL	5.278174	8.422054	3.14388	1.23E-23
UBXN11	2.199308	4.94634	2.747033	1.29E-23
PDCL3	7.059619	11.33533	4.275716	1.38E-23
CWC27	3.18245	4.958769	1.77632	1.45E-23
TOE1	2.615437	4.200686	1.585249	1.47E-23

ACYP1	1.137207	2.484705	1.347499	1.68E-23
MYL6B	4.285807	9.258002	4.972195	1.74E-23
CENPB	26.30962	38.85778	12.54816	1.78E-23
NRF1	1.962059	3.09464	1.132581	1.79E-23
MASP1	10.51337	4.96117	-5.5522	1.81E-23
BNIP1	2.829475	4.547825	1.71835	1.82E-23
PPP1R14B	14.11938	32.04698	17.9276	1.85E-23
RAN	25.08434	43.48908	18.40475	1.85E-23
CENPM	2.314867	7.446904	5.132037	1.91E-23
VASP	13.61937	30.85627	17.23691	1.93E-23
PYCR2	7.370355	12.25664	4.886288	2.05E-23
STXBP2	3.190294	6.379453	3.189159	2.12E-23
MAPK3	8.208513	13.30399	5.095477	2.20E-23
CTDNEP1	5.941788	11.4984	5.556613	2.42E-23
TSEN54	6.473776	12.80538	6.3316	2.48E-23
BRAP	3.420342	4.722373	1.302031	2.51E-23
FAM50A	26.08036	48.79583	22.71547	2.66E-23
EBNA1BP2	11.77021	18.31822	6.548012	2.71E-23
RPL37	58.04707	111.2249	53.1778	2.72E-23
PLG	226.877	99.7493	-127.128	2.81E-23
MYCBP	2.689661	4.909731	2.22007	2.88E-23
UPB1	34.46261	15.04924	-19.4134	2.90E-23
DYNLT1	10.9468	17.32979	6.382992	2.93E-23
RFXANK	6.818956	12.64977	5.830814	3.07E-23
RTRAF	7.992252	11.19149	3.19924	3.27E-23
UBALD2	18.40247	41.89268	23.49021	3.42E-23
RFC2	9.970015	17.03552	7.065503	3.46E-23
CPSF3	7.257859	10.67845	3.420592	3.73E-23
PFDN1	9.719847	16.35784	6.637992	3.82E-23
CREB3	17.12886	28.78357	11.65471	4.09E-23
FARSB	4.380003	6.689593	2.30959	4.26E-23
PCLAF	0.637316	1.909379	1.272063	4.34E-23
BAX	16.73461	30.90421	14.16961	4.36E-23
CBY1	4.112902	6.898955	2.786053	4.46E-23
NAP1L4	15.03754	22.4787	7.441163	4.46E-23
CHTOP	8.923192	14.64259	5.7194	4.84E-23
PRKRIP1	4.752178	7.980539	3.228361	4.95E-23
BANF1	59.6144	88.03373	28.41934	5.07E-23
SMARCD1	6.346541	11.19387	4.847331	5.11E-23
CXXC1	6.624985	10.85403	4.229042	5.25E-23
DDX27	5.201779	8.342666	3.140887	5.43E-23
CDK5RAP1	4.646191	6.286377	1.640187	5.68E-23
DBNL	5.775947	8.696169	2.920222	5.75E-23
RBM17	6.56036	10.66881	4.108453	6.12E-23
ANP32B	44.05659	71.99385	27.93726	6.26E-23
PRPF6	36.92571	55.52307	18.59736	6.42E-23
AATF	10.16355	15.73102	5.567468	6.50E-23
CHEK1	0.895806	2.458318	1.562512	7.24E-23
TFR2	178.4968	87.0048	-91.492	8.30E-23
KMT5A	2.783625	4.627265	1.84364	8.55E-23
UXT	20.97245	33.1486	12.17615	8.61E-23
HMGN1	12.44594	21.17333	8.727388	8.66E-23
PES1	14.40232	23.57746	9.17514	8.78E-23
AAAS	6.871445	9.71568	2.844235	8.85E-23
TRIM11	1.646903	3.018026	1.371123	8.93E-23
VPS4A	4.437551	7.029834	2.592283	9.04E-23
HMGN3	26.31707	49.3454	23.02834	1.02E-22
DDX56	15.34449	21.96659	6.622105	1.03E-22

TSSC4	10.39152	16.88292	6.491403	1.03E-22
RPL24	126.0358	224.2352	98.19933	1.06E-22
RALA	8.363328	13.36569	5.002359	1.06E-22
KARS1	20.83094	31.6182	10.78725	1.09E-22
SF3B6	39.09333	54.79989	15.70656	1.12E-22
RBM8A	7.471301	10.95735	3.486048	1.22E-22
MOB2	4.667009	6.828618	2.161608	1.24E-22
EIF1AD	5.472089	7.4114	1.939311	1.26E-22
CENPT	2.35444	4.246885	1.892445	1.29E-22
LUC7L2	2.837496	3.858212	1.020716	1.33E-22
CHTF18	1.288506	3.027375	1.738869	1.35E-22
GLYATL1	20.19675	7.320406	-12.8763	1.35E-22
KIF2C	1.330608	5.0655	3.734892	1.44E-22
DDX51	2.593951	3.962875	1.368924	1.47E-22
OTC	77.2526	30.96494	-46.2877	1.48E-22
DENR	11.15886	16.6815	5.522638	1.57E-22
TXNL4A	7.521936	12.18925	4.667313	1.71E-22
AC106795	1.428052	2.681134	1.253082	1.71E-22
RHOG	17.93093	31.44171	13.51078	1.78E-22
E2F4	7.91562	13.36437	5.448754	1.87E-22
PSMA1	3.559765	6.388654	2.82889	1.87E-22
WASHC1	1.564911	3.1251	1.560189	1.88E-22
MSANTD3	2.567559	5.131943	2.564384	1.90E-22
CYP4F2	42.92031	15.79745	-27.1229	1.91E-22
RTL8A	14.52481	30.30825	15.78344	1.91E-22
MED7	2.791868	4.239591	1.447722	1.91E-22
NAP1L1	8.288104	17.33739	9.049286	2.02E-22
SNRNP40	3.97733	6.60169	2.62436	2.09E-22
LSM2	22.04767	38.13087	16.0832	2.16E-22
RPUSD1	5.236178	8.801711	3.565533	2.35E-22
CCDC12	5.858924	9.208599	3.349675	2.43E-22
RPP30	3.248843	5.064	1.815157	2.48E-22
EIF3I	13.40735	19.86139	6.454043	2.54E-22
VOPP1	4.149935	7.623165	3.47323	2.78E-22
AL031727.	0.958899	2.351294	1.392395	2.85E-22
RHNO1	3.670918	7.014431	3.343513	2.94E-22
MYL6	66.80038	112.4045	45.60411	3.01E-22
SFT2D1	3.048434	6.087563	3.039129	3.06E-22
NECAP2	6.406379	9.83976	3.433381	3.08E-22
GGCT	7.621911	14.18237	6.560455	3.09E-22
PNKP	5.089958	8.3526	3.262642	3.17E-22
DYNC2I2	11.28575	28.77859	17.49284	3.40E-22
GINS2	1.857423	4.255269	2.397847	3.46E-22
SPPL3	5.053296	8.102286	3.048991	3.50E-22
WDR70	2.459715	3.589562	1.129846	3.64E-22
SERPINA1	51.24045	28.14484	-23.0956	3.74E-22
CWC15	14.82365	20.41304	5.589389	4.02E-22
JPT1	9.242756	24.02878	14.78602	4.24E-22
IQGAP2	23.27503	12.86588	-10.4092	4.56E-22
RPS2	173.3323	348.6975	175.3652	4.67E-22
PRPF31	11.81477	21.22267	9.407904	4.80E-22
TEDC2	1.109046	2.943439	1.834393	5.02E-22
CDC45	1.329656	3.679917	2.350262	5.17E-22
IER3IP1	6.110759	8.7973	2.686541	5.34E-22
AC025449	1.677562	3.369871	1.692309	5.57E-22
OAZ1	68.14513	122.2932	54.14805	5.67E-22
TRIM65	3.333454	6.001101	2.667647	5.81E-22
NT5C	5.531924	11.96796	6.436032	6.14E-22

SAMD1	12.39516	21.32787	8.932711	6.15E-22
DDA1	5.742749	9.508716	3.765967	6.27E-22
YAE1	2.041281	3.199372	1.158092	6.76E-22
RBPJ	3.294903	5.17642	1.881517	7.10E-22
CIZ1	4.307666	7.283108	2.975442	7.74E-22
BUD23	7.34564	10.6278	3.282156	7.92E-22
GTF3C2	3.676758	6.103716	2.426958	8.15E-22
IRF3	12.41781	20.7088	8.290986	8.17E-22
FTSJ1	5.932364	9.6164	3.684036	9.01E-22
SPC25	1.20409	2.955587	1.751498	9.77E-22
SERPINB6	4.228605	7.72099	3.492385	9.82E-22
KATNA1	2.872347	4.450985	1.578637	9.86E-22
MAPRE1	15.11202	29.75136	14.63934	1.04E-21
RPL6	92.53546	159.8892	67.35372	1.04E-21
ZNF580	2.864178	6.415548	3.551369	1.06E-21
TMEM258	11.53232	19.14047	7.60815	1.09E-21
DTX2	2.108996	3.473668	1.364673	1.13E-21
COG4	8.89492	13.11463	4.219711	1.13E-21
SLCO1B1	66.53994	28.87244	-37.6675	1.16E-21
TACC3	2.425909	6.397657	3.971747	1.20E-21
NUDT1	2.971664	8.540067	5.568404	1.23E-21
FAM89B	1.260586	2.498554	1.237968	1.26E-21
EIF4E2	8.607646	12.02573	3.418081	1.27E-21
PARL	2.454984	4.722832	2.267848	1.28E-21
EWSR1	13.67794	19.57027	5.892336	1.28E-21
TRMT112	75.29616	110.8906	35.59447	1.33E-21
SYNGR2	26.69737	50.21955	23.52218	1.35E-21
TMEM147	20.46384	38.81471	18.35087	1.40E-21
SPDL1	1.130621	2.27274	1.14212	1.47E-21
NUP62	5.638362	9.953878	4.315516	1.49E-21
MIIP	6.575761	11.77849	5.202728	1.60E-21
RBMX	12.16241	19.17497	7.012551	1.62E-21
PRCC	14.37117	22.34184	7.97067	1.71E-21
LSM10	13.38983	20.02216	6.63233	1.73E-21
ALDH6A1	34.88786	15.77473	-19.1131	1.74E-21
PSME3IP1	8.715761	13.08137	4.365613	1.79E-21
NOP56	10.07183	19.97776	9.905931	1.80E-21
ETFDH	14.08087	7.53303	-6.54784	1.90E-21
SUCLG2	49.28072	31.37478	-17.9059	1.99E-21
ATF4	78.02922	118.9704	40.94117	2.00E-21
ELOVL1	18.85919	32.56933	13.71014	2.34E-21
SPATS2	2.109909	4.365059	2.25515	2.43E-21
TRMU	2.054516	3.655795	1.601278	2.43E-21
TMEM11	6.601502	10.30946	3.707957	2.48E-21
STMN1	8.006019	19.57819	11.57217	2.61E-21
SSBP1	9.433289	13.41409	3.980804	2.62E-21
METTL7A	155.8285	91.50171	-64.3268	2.69E-21
RRP1	6.338139	10.02087	3.682726	2.80E-21
ZNF133	1.302022	2.353265	1.051243	2.98E-21
ARPC2	16.19892	26.39327	10.19435	2.98E-21
B4GALT2	6.755248	10.88568	4.130437	3.01E-21
PHF19	1.333794	3.178468	1.844674	3.21E-21
AGTRAP	6.833177	14.03737	7.204191	3.34E-21
TIMM9	9.301452	13.64548	4.344026	3.50E-21
RCC2	6.836639	15.47193	8.635292	3.85E-21
DSTNP2	2.233226	4.053162	1.819936	3.87E-21
UTP6	4.687624	6.846499	2.158875	3.88E-21
CHMP1A	8.001878	12.7687	4.766819	4.01E-21

HNRNPM	22.59479	32.45858	9.863789	4.19E-21
RUVBL2	18.90432	29.2772	10.37287	4.24E-21
MXD3	1.472268	2.948252	1.475984	4.34E-21
POLR3F	2.851233	4.202175	1.350943	4.46E-21
FAF1	4.993191	7.125291	2.1321	4.57E-21
TROAP	1.202656	3.676265	2.473609	4.60E-21
RPL19	206.4628	347.1608	140.698	4.74E-21
NSUN5	6.53114	10.34373	3.81259	4.78E-21
UBE2Z	11.19849	19.44117	8.242677	4.90E-21
DPM2	13.45888	25.36756	11.90868	4.94E-21
DHTKD1	34.78813	18.59687	-16.1913	5.11E-21
TMED3	1.231825	3.831482	2.599657	5.18E-21
SUPT5H	12.36415	18.94538	6.581227	5.18E-21
SENP3	1.542291	2.587831	1.045539	5.20E-21
NDUFS5	160.5261	284.8797	124.3536	5.33E-21
CDO1	113.5794	54.88693	-58.6925	5.34E-21
AC011468	0.78813	1.887169	1.099039	5.53E-21
NAT9	5.821958	9.510656	3.688698	5.62E-21
COX19	1.509589	2.740966	1.231376	5.69E-21
MAT1A	278.5557	134.0153	-144.54	5.71E-21
CHAF1B	0.821356	2.330786	1.50943	5.73E-21
CC2D1B	3.65145	5.515609	1.86416	5.85E-21
CDCA5	2.20846	5.561767	3.353307	6.11E-21
ENTPD5	21.99947	11.00913	-10.9903	6.29E-21
PHKG2	2.609907	4.250558	1.64065	6.33E-21
NEDD8	5.659921	9.199427	3.539506	6.45E-21
MBOAT7	8.699732	14.27811	5.578375	6.47E-21
CDCA3	0.924547	2.64333	1.718783	6.52E-21
ANKRD13	1.964835	4.642609	2.677774	6.91E-21
SYF2	8.863639	12.16108	3.297438	6.97E-21
MRTO4	10.06608	16.92249	6.856411	7.07E-21
AMZ2	5.882982	9.206828	3.323845	7.14E-21
PFDN2	76.2814	116.7701	40.48868	8.18E-21
DCTPP1	9.589028	18.07075	8.481724	8.67E-21
DNAJC7	2.977737	4.258427	1.28069	9.18E-21
AC246787	1.982541	5.286979	3.304438	9.62E-21
PPP1CC	10.90151	16.11054	5.20903	1.00E-20
SLC35C2	7.241823	10.02166	2.779834	1.04E-20
SERTAD3	6.842598	12.81889	5.976295	1.05E-20
TRA2A	8.497766	13.42917	4.931402	1.07E-20
WDR75	4.213116	6.715538	2.502422	1.07E-20
MIR1244-	14.84437	35.39233	20.54796	1.08E-20
CD63	181.9261	310.1581	128.232	1.08E-20
ILF2	49.64991	78.03107	28.38116	1.11E-20
RRP36	13.48066	19.56316	6.082503	1.13E-20
STARD3NI	2.591128	4.64257	2.051442	1.14E-20
RPS27	400.19	749.3267	349.1367	1.15E-20
SCNM1	6.113807	12.07736	5.963549	1.19E-20
ZBTB17	3.811779	5.738771	1.926992	1.19E-20
BABAM1	6.687366	11.94139	5.254025	1.19E-20
SNRNP70	26.48073	42.00833	15.5276	1.24E-20
HNRNPA3	22.18177	32.08948	9.907716	1.26E-20
PSENEN	7.522007	16.2647	8.742696	1.30E-20
RBCK1	19.49574	31.95609	12.46036	1.30E-20
COA8	2.061955	3.353092	1.291137	1.31E-20
ATP6V0B	33.9614	53.67907	19.71767	1.32E-20
NAGK	2.977458	4.98243	2.004972	1.36E-20
RPL11	156.2772	261.8502	105.573	1.36E-20

HNRNPL	11.48457	16.54301	5.058431	1.37E-20
SRSF7	13.48257	19.48275	6.000177	1.39E-20
HSPBP1	16.16681	26.59434	10.42753	1.40E-20
RPF1	8.25587	12.39105	4.135181	1.41E-20
STK11IP	1.779457	3.349358	1.5699	1.48E-20
SNRPE	22.41155	37.96378	15.55222	1.50E-20
PABPC4	8.271771	13.4346	5.162825	1.53E-20
PDAP1	27.1361	39.16243	12.02633	1.54E-20
LAMTOR1	24.78941	35.04935	10.25994	1.55E-20
SEC61G	22.96659	41.78955	18.82296	1.58E-20
SSNA1	31.87807	55.04959	23.17152	1.62E-20
SSR2	25.09288	44.23167	19.13879	1.62E-20
RBM14	4.549909	6.353379	1.80347	1.65E-20
NFKBIE	6.379718	13.52462	7.1449	1.70E-20
TCF3	4.844329	9.945134	5.100805	1.73E-20
ILF3	11.44697	19.08489	7.637924	1.73E-20
BLVRA	8.673736	18.34964	9.675899	1.77E-20
EIF3F	6.422341	9.868824	3.446483	1.78E-20
R3HDM4	14.69994	25.39999	10.70005	1.80E-20
TFPT	5.813751	10.04831	4.234563	1.81E-20
NOP10	99.30835	147.6867	48.37831	1.81E-20
NDC1	3.548298	6.310868	2.762571	1.83E-20
MORN2	4.908273	8.650069	3.741796	1.92E-20
BDH1	16.73115	8.933871	-7.79728	1.97E-20
DPYS	77.28106	33.94534	-43.3357	2.00E-20
P3H1	4.942818	8.272801	3.329983	2.03E-20
RBM3	18.15771	30.34368	12.18597	2.13E-20
MRPS15	23.64526	35.82611	12.18086	2.14E-20
ZNF865	3.349331	5.409073	2.059742	2.17E-20
RHOT2	10.21879	15.32034	5.101552	2.19E-20
ACOX2	32.8092	16.41061	-16.3986	2.20E-20
ORC6	0.444978	1.458052	1.013074	2.21E-20
EMG1	3.572199	5.787663	2.215464	2.24E-20
PRR11	0.793128	2.41641	1.623282	2.42E-20
SNHG12	0.806101	2.126465	1.320364	2.50E-20
SNHG17	1.024453	2.053169	1.028716	2.52E-20
HSD17B4	37.23417	20.45459	-16.7796	2.56E-20
REXO4	8.81881	13.61072	4.791911	2.56E-20
SNRPA1	4.916516	8.400205	3.483689	2.61E-20
ZNF692	4.334973	8.531054	4.196081	2.64E-20
CCNB2	2.5172	6.525219	4.008019	3.02E-20
TAF11	9.77909	14.1376	4.35851	3.04E-20
POP5	9.186356	13.75121	4.564856	3.04E-20
SH3GL1	12.48118	19.24901	6.767828	3.11E-20
THOC7	17.4712	24.82905	7.357847	3.14E-20
H2AX	12.38702	27.2448	14.85778	3.25E-20
RABL6	2.16112	5.335924	3.174803	3.36E-20
PSMG3	9.13793	20.18848	11.05055	3.67E-20
CAT	118.8048	65.77646	-53.0283	3.70E-20
ORC1	0.809789	2.396408	1.586619	3.71E-20
EIF3B	17.62489	29.13672	11.51182	3.85E-20
ESS2	2.293373	3.349475	1.056102	3.93E-20
MTA2	10.8396	16.80945	5.969851	4.18E-20
RNF34	3.175072	5.134297	1.959225	4.38E-20
PCSK6	6.343463	2.766112	-3.57735	4.60E-20
YBX1	64.70359	105.9103	41.20674	4.66E-20
HIKESHI	4.227829	6.332394	2.104565	4.77E-20
BRAT1	7.570623	10.80973	3.239103	4.91E-20

HAUS1	3.48522	6.098796	2.613576	5.04E-20
NELFB	19.49129	29.20878	9.717483	5.05E-20
C19orf48	11.68373	25.03061	13.34688	5.23E-20
SNRPGP2	5.046834	8.138869	3.092035	5.39E-20
PPP2CA	14.65623	19.28473	4.628497	5.41E-20
PHC2	17.74777	28.83797	11.0902	5.95E-20
KDM1A	5.780426	9.541977	3.761552	6.08E-20
BMS1	3.758316	5.745829	1.987513	6.26E-20
RPL35	167.8793	340.0379	172.1586	6.26E-20
ZNF579	2.307354	4.841409	2.534055	6.33E-20
SLC52A2	4.940717	11.6658	6.725079	6.62E-20
ITGB1BP1	3.366366	5.799428	2.433063	6.69E-20
SPSB2	3.246373	6.009826	2.763453	7.13E-20
CNOT9	6.058119	9.225804	3.167685	7.36E-20
DUSP12	5.223752	8.703911	3.480159	7.37E-20
PELP1	6.64977	10.60202	3.952254	7.45E-20
WDR77	4.214087	6.628169	2.414082	7.55E-20
ZNF691	4.073543	6.315721	2.242178	7.66E-20
STK25	8.393036	12.01065	3.617619	7.69E-20
PWP1	12.3033	16.7845	4.481197	7.95E-20
KRT10	7.383711	12.59842	5.214704	7.95E-20
C6	68.25514	26.41452	-41.8406	7.98E-20
TELO2	6.769473	10.19732	3.427842	8.06E-20
RSL1D1	14.98739	22.59997	7.612582	8.14E-20
AP5Z1	3.836541	6.119855	2.283314	8.18E-20
ACSM2A	30.72508	13.52662	-17.1985	8.24E-20
EFNA4	5.447121	11.24688	5.799763	8.26E-20
ZNF205	3.356131	5.70725	2.351119	8.56E-20
YKT6	5.231759	9.6513	4.419541	9.18E-20
MED10	8.418082	13.92346	5.505375	9.20E-20
DPH2	5.514907	8.995306	3.4804	9.89E-20
ABHD12	14.5494	21.93969	7.390289	9.91E-20
FUBP1	4.849635	8.698632	3.848997	1.01E-19
RPL18	108.0607	207.9777	99.917	1.04E-19
MTF2	1.475935	2.888435	1.4125	1.04E-19
FIBP	10.8375	15.36405	4.526547	1.04E-19
SAFB2	6.843082	9.773643	2.930561	1.08E-19
DGKZ	2.7559	4.488616	1.732716	1.10E-19
WTAP	5.262791	7.813039	2.550248	1.11E-19
CSNK1D	7.388761	10.82917	3.44041	1.15E-19
SDHAF2	4.758306	8.250163	3.491857	1.16E-19
CEP89	0.996605	2.311874	1.315269	1.19E-19
IFNGR2	10.32857	19.37288	9.04431	1.22E-19
EFTUD2	6.668511	10.11044	3.441932	1.22E-19
HNRNPA2	35.36031	48.66454	13.30423	1.26E-19
NUP93	1.380801	2.695477	1.314676	1.27E-19
BLOC1S4	6.870199	10.55094	3.680745	1.30E-19
ZNF32	7.561185	12.4394	4.878215	1.30E-19
RPL39	36.41132	66.92618	30.51486	1.46E-19
PTMA	85.26785	132.7602	47.4923	1.46E-19
METTL9	2.914533	6.139185	3.224653	1.49E-19
DHX34	3.691544	6.0158	2.324256	1.49E-19
RPL23	71.88813	129.0511	57.16298	1.56E-19
DSN1	5.373824	9.216246	3.842422	1.59E-19
COPS9	10.3699	20.12023	9.750326	1.61E-19
ASF1B	3.113197	8.703512	5.590315	1.61E-19
SET	31.47378	49.10697	17.63319	1.66E-19
TRMT1	8.286961	13.86215	5.575193	1.67E-19

MRPL11	13.40665	20.02828	6.621637	1.67E-19
RPL4	142.6154	246.3812	103.7657	1.68E-19
CENPW	4.246733	12.17669	7.929955	1.69E-19
PTDSS2	3.252119	5.889683	2.637564	1.70E-19
RACK1	116.893	194.2093	77.31623	1.74E-19
BUD31	8.068735	11.79609	3.727357	1.75E-19
NR2C2AP	5.261649	9.388712	4.127063	1.81E-19
RANBP1	8.973361	15.38136	6.408001	1.82E-19
GPN1	4.807211	7.243067	2.435857	1.84E-19
LSM4	28.5231	49.06466	20.54156	1.89E-19
EMC8	3.697507	5.465491	1.767984	1.93E-19
PDZD11	18.7978	27.74995	8.952145	1.97E-19
CDC20	4.5137	18.3901	13.8764	1.99E-19
SFSWAP	2.490242	3.906222	1.41598	2.12E-19
SPINDOC	2.382757	5.604154	3.221397	2.17E-19
LIG1	3.216394	5.620733	2.404338	2.21E-19
CHCHD2	151.4358	213.2241	61.78824	2.22E-19
THAP3	2.256007	3.411937	1.15593	2.27E-19
POLR1C	2.594067	3.92281	1.328743	2.29E-19
MZT2A	6.366641	12.57822	6.211581	2.36E-19
HDAC2	2.169146	3.854047	1.6849	2.67E-19
TSEN34	9.596782	14.05863	4.461851	2.69E-19
POLE3	13.10669	19.24823	6.141539	2.70E-19
ERH	40.34805	57.54454	17.1965	2.74E-19
CDCA8	2.326045	6.435527	4.109482	2.74E-19
ATP1B3	8.638256	20.41045	11.77219	2.77E-19
ARFIP2	7.246927	10.58249	3.335567	2.81E-19
NUP107	2.485131	4.114003	1.628873	2.82E-19
HDGFL2	11.68502	17.28132	5.596301	2.98E-19
SAE1	16.01218	25.86907	9.85689	2.99E-19
WDR53	2.977182	4.229981	1.252799	3.10E-19
IQCB1	2.540561	4.404373	1.863813	3.14E-19
RPS7	62.52532	108.24	45.71469	3.14E-19
ARF3	12.75018	21.69457	8.944398	3.17E-19
ANKZF1	4.067667	6.382807	2.315141	3.21E-19
DNAJC8	18.85259	27.27451	8.421922	3.21E-19
ZNF213	0.889065	2.037394	1.148329	3.30E-19
RPS11	462.3737	847.7834	385.4098	3.36E-19
LSM5	4.499893	6.975798	2.475904	3.36E-19
THOC3	1.966972	3.031327	1.064355	3.40E-19
CKS2	19.18462	44.69098	25.50635	3.41E-19
LUC7L3	6.478439	10.5407	4.062266	3.48E-19
ERGIC3	30.10549	43.07539	12.96991	3.50E-19
CAPNS1	64.09637	105.92	41.82358	3.53E-19
ODF2	2.77665	4.83042	2.053769	3.61E-19
C2orf68	4.8355	7.043493	2.207993	3.69E-19
SLCO2B1	24.52467	10.82214	-13.7025	3.74E-19
UBL7	16.69829	24.26384	7.565542	3.82E-19
SF3B4	40.07633	67.261	27.18467	3.92E-19
AL138847.	5.198964	2.23643	-2.96253	4.02E-19
UFC1	32.84956	52.78271	19.93315	4.06E-19
PRR14	6.076233	9.131608	3.055376	4.20E-19
TRAPPC4	4.790295	8.079548	3.289253	4.20E-19
SUPT7L	4.794463	6.859958	2.065495	4.24E-19
RPLP2	250.9155	538.9437	288.0282	4.25E-19
NOL10	4.768435	6.568505	1.80007	4.35E-19
POLR3K	4.799235	7.535353	2.736118	4.43E-19
SNRPC	56.94574	88.87696	31.93122	4.44E-19

CFAP36	4.044488	8.056929	4.012441	4.47E-19
NCBP2AS2	11.77003	19.342	7.571962	4.50E-19
HEIH	1.957547	3.593273	1.635726	4.68E-19
PEDS1	3.289061	5.76654	2.47748	4.77E-19
UBE2S	3.666952	9.288918	5.621967	4.86E-19
NCBP2	7.232378	11.47299	4.240607	4.86E-19
SUGP1	4.797124	6.607598	1.810473	4.91E-19
CSNK1E	3.444789	6.811941	3.367152	5.10E-19
CIB1	53.536	85.36879	31.83279	5.12E-19
IK	19.03346	25.73814	6.704679	5.31E-19
XPO6	7.200959	10.57972	3.378766	5.33E-19
RPLP1	257.6003	472.6403	215.04	5.35E-19
AC005261	3.406177	5.765321	2.359144	5.55E-19
TTPA	30.80058	11.69607	-19.1045	5.64E-19
THUMPD2	1.323175	2.331	1.007825	5.71E-19
MYBL2	3.934715	15.87045	11.93574	5.77E-19
AP001505	2.334942	5.95771	3.622768	5.90E-19
TONSL	1.707322	3.384525	1.677202	6.00E-19
HPX	748.9304	355.4299	-393.501	6.03E-19
TRIM28	35.59453	63.4295	27.83497	6.14E-19
KRT8P12	2.023753	4.009982	1.986229	6.25E-19
AL035461.	0.498885	1.649003	1.150118	6.34E-19
SURF6	6.340414	9.312127	2.971713	6.51E-19
HNRNPU	12.50864	17.90204	5.393402	6.63E-19
PAH	121.5095	62.06446	-59.445	6.65E-19
RPL12	133.1394	243.6771	110.5377	6.65E-19
ABCA6	6.889147	2.839543	-4.0496	6.86E-19
C11orf49	1.069242	2.663847	1.594605	7.16E-19
PRMT1	10.76594	20.35084	9.584905	7.33E-19
MARCKSL	30.81513	83.65556	52.84043	7.58E-19
GFER	2.319157	3.815423	1.496266	7.60E-19
F11	18.22499	7.787269	-10.4377	7.92E-19
ANGPTL3	117.4356	59.63789	-57.7978	7.96E-19
VPS72	19.17966	32.00864	12.82899	8.28E-19
PBDC1	11.49197	17.41946	5.92749	8.43E-19
NAE1	4.882341	7.074033	2.191692	8.49E-19
UBTF	12.18801	17.10922	4.921212	8.64E-19
RAB13	5.287592	8.970686	3.683095	8.70E-19
PDCD2	3.815683	5.71033	1.894647	9.24E-19
ARFRP1	5.225639	7.819553	2.593914	9.26E-19
CMTM7	1.748959	4.539537	2.790578	9.47E-19
RBP4	3944.326	2126.658	-1817.67	9.56E-19
SMIM30	7.543501	12.75263	5.209126	9.66E-19
CYTH2	3.470474	6.73628	3.265806	9.71E-19
EIF3D	35.10436	55.32195	20.2176	9.73E-19
KLHDC4	1.64114	2.811735	1.170596	9.91E-19
RSL24D1	7.273022	10.23599	2.962968	1.00E-18
SCMH1	2.741148	5.445546	2.704398	1.04E-18
SERGEF	2.836131	4.638274	1.802144	1.04E-18
BX255925	5.289388	11.1418	5.852415	1.08E-18
TK1	13.60594	31.08184	17.47589	1.08E-18
PLCB3	3.251523	6.001516	2.749993	1.11E-18
RPA2	10.90454	16.88943	5.984889	1.12E-18
TRIM47	6.750469	17.13348	10.38301	1.15E-18
TRAPPC2L	6.1319	10.36696	4.235063	1.15E-18
BRK1	75.95962	104.1116	28.15196	1.17E-18
SF3A2	14.30791	26.87214	12.56423	1.19E-18
BIRC5	3.663737	11.21897	7.555234	1.20E-18

SNHG15	1.189391	3.003291	1.8139	1.22E-18
PYGO2	12.74875	21.70454	8.955795	1.23E-18
IFT52	3.88326	7.63553	3.75227	1.30E-18
AP4M1	1.387637	2.719003	1.331366	1.31E-18
MTHFD1L	1.927143	3.994542	2.067399	1.32E-18
OLA1	7.986319	12.14741	4.161094	1.37E-18
SNORA33	2.846056	7.359739	4.513683	1.39E-18
TBRG4	9.977301	14.04507	4.067767	1.46E-18
CDK2	3.208083	5.678415	2.470332	1.55E-18
SF3B5	86.02754	139.4613	53.43376	1.57E-18
HMG20B	10.9652	16.06822	5.103014	1.61E-18
SZRD1	11.44642	16.696	5.249578	1.63E-18
GINS1	1.496746	3.699741	2.202995	1.64E-18
PFN1	152.6558	264.2817	111.626	1.65E-18
UBXN1	26.71746	42.30768	15.59022	1.65E-18
GMPPA	10.27904	14.6162	4.337161	1.66E-18
AL355488.	0.648589	1.849052	1.200463	1.67E-18
RPIA	4.895039	7.879439	2.9844	1.69E-18
EXOSC8	3.296701	4.902285	1.605584	1.73E-18
MAD2L1	1.056681	2.523455	1.466774	1.74E-18
SART1	13.84522	18.90907	5.063846	1.75E-18
MFF	6.416581	9.713846	3.297265	1.75E-18
LYPLA2	32.8729	47.09196	14.21906	1.88E-18
C7orf26	7.00211	9.985453	2.983342	1.96E-18
CASP2	1.852194	3.575421	1.723227	1.97E-18
IVD	26.71078	17.97663	-8.73415	2.06E-18
MRPL52	10.15559	17.72582	7.570233	2.07E-18
NANS	3.873583	5.608136	1.734553	2.13E-18
EXOSC6	3.664082	5.258452	1.59437	2.17E-18
ALDH5A1	20.50837	11.21828	-9.29009	2.18E-18
ADO	2.738953	4.668897	1.929944	2.21E-18
STK32C	1.065624	2.466093	1.400469	2.29E-18
RUVBL1	3.759044	6.077884	2.31884	2.30E-18
AAGAB	6.158699	9.083782	2.925083	2.31E-18
TBC1D22B	2.280564	3.989897	1.709333	2.31E-18
RPS14	94.86427	157.7184	62.85413	2.40E-18
ZNF581	6.401045	12.27946	5.878419	2.40E-18
YARS2	3.946784	5.780685	1.833901	2.42E-18
YTHDF1	14.73827	19.78105	5.042772	2.50E-18
SMARCAL	2.984781	4.598888	1.614107	2.61E-18
RPS9	116.4073	203.8691	87.46179	2.64E-18
EXOSC7	3.527888	5.021918	1.494031	2.66E-18
SFR1	1.444884	2.459437	1.014553	2.67E-18
P2RX4	2.963622	5.138312	2.17469	2.67E-18
CCNB1	6.190997	14.98294	8.791946	2.68E-18
SAR1B	15.92217	10.23359	-5.68858	2.72E-18
AL359921.	1.29715	2.663167	1.366018	2.74E-18
SAFB	9.444982	12.84703	3.402051	2.74E-18
MCM3	16.53872	31.16866	14.62994	2.76E-18
HSPA14	3.570245	5.439863	1.869618	2.79E-18
MAN1B1	7.934637	11.74284	3.808207	2.91E-18
RPS15	83.79868	154.882	71.08329	2.91E-18
YEATS2	1.326769	2.453619	1.126849	2.92E-18
KIFC1	3.475856	8.741115	5.26526	2.94E-18
HM13	21.40379	31.00496	9.601166	2.96E-18
FOXD2-AS	1.05114	2.55724	1.5061	2.96E-18
MRPS34	42.02058	68.33765	26.31707	2.99E-18
PTBP1	24.8381	33.4907	8.652593	3.02E-18

BRD9	2.125259	3.51184	1.386581	3.02E-18
CEBPZOS	5.488953	7.971014	2.482061	3.07E-18
PSMG2	12.69346	17.00517	4.311713	3.10E-18
UIMC1	2.693821	4.069055	1.375234	3.11E-18
POMGNT1	9.465735	13.65235	4.186613	3.16E-18
B3GALT6	6.573438	9.903101	3.329663	3.17E-18
AL604028.	2.260498	3.510834	1.250337	3.21E-18
RTCA	7.198831	10.6424	3.443572	3.29E-18
FAM110A	1.749071	4.233794	2.484722	3.31E-18
OSGEP	3.672753	5.215958	1.543205	3.40E-18
MED19	4.089658	5.884768	1.79511	3.43E-18
SREK1IP1	2.01306	3.060211	1.047152	3.43E-18
HMGN4	13.44521	24.17046	10.72525	3.43E-18
TIMM29	6.01301	8.205258	2.192248	3.46E-18
RPL27A	63.84897	129.8088	65.95982	3.52E-18
RPS24	70.05096	139.1488	69.09783	3.62E-18
ATAD3B	1.458406	2.955631	1.497225	3.83E-18
SCAMP3	38.38537	59.75139	21.36602	3.88E-18
XRCC6	60.24769	84.61653	24.36884	3.92E-18
AC098583	1.183798	2.413369	1.229571	3.99E-18
ZNF48	2.117896	3.388149	1.270253	4.11E-18
RPL38	67.68538	134.2679	66.58255	4.15E-18
ATP5F1E	39.00273	67.02201	28.01928	4.15E-18
RPS3A	43.49777	80.45735	36.95958	4.20E-18
PDCD5	18.50253	31.28931	12.78678	4.45E-18
STRN4	5.931429	9.285067	3.353638	4.56E-18
CEP95	1.883498	3.363683	1.480185	4.63E-18
TOMM7	53.19282	84.156	30.96318	4.64E-18
YDJC	6.957341	12.28343	5.326092	4.68E-18
RUSC1	3.420337	6.987793	3.567456	4.72E-18
ZRSR2	2.814088	5.236461	2.422373	4.77E-18
SNRNP35	3.992206	6.102247	2.110041	4.87E-18
ALAD	48.60114	27.56272	-21.0384	4.87E-18
CTU2	2.760501	4.115872	1.355371	4.90E-18
RWDD1	5.023315	7.123105	2.09979	4.95E-18
AC145207	0.779061	1.804897	1.025836	5.03E-18
PTOV1	3.711521	5.537707	1.826187	5.21E-18
RASSF7	17.25762	37.00133	19.74371	5.29E-18
MRPL51	51.89891	74.80812	22.90921	5.36E-18
RPL27	183.809	320.7901	136.9812	5.58E-18
MED22	2.200388	4.061498	1.861109	5.63E-18
METTL2A	2.284167	3.292842	1.008676	5.67E-18
FHL3	4.977221	10.05401	5.076792	5.67E-18
SMS	17.8887	29.83204	11.94335	5.80E-18
HNRNPA1	38.90925	63.83665	24.92739	5.82E-18
GRPEL2	2.654879	3.87416	1.21928	5.84E-18
SQOR	10.37701	16.78498	6.407972	5.88E-18
BOD1	11.40427	16.32568	4.921416	5.88E-18
NCOA5	6.637022	10.71612	4.079096	6.14E-18
WDR4	2.631421	4.475828	1.844407	6.19E-18
CPSF6	4.471475	6.989782	2.518306	6.30E-18
NT5C3A	3.377994	6.369553	2.991559	6.32E-18
UNC45A	6.100603	9.139986	3.039383	6.43E-18
STX6	2.258876	4.074308	1.815433	6.71E-18
TRIP4	5.021288	7.038872	2.017584	6.76E-18
MRPL33	15.13852	22.6722	7.533676	6.83E-18
COMMD4	10.25269	15.28625	5.033553	6.90E-18
ABCC6	22.76897	13.4756	-9.29337	7.03E-18

RPS13	109.6835	178.2549	68.57143	7.32E-18
VPS29	13.59256	17.93821	4.345654	7.53E-18
PURB	3.351633	4.996622	1.644989	7.60E-18
F13B	55.45197	25.08324	-30.3687	7.82E-18
OFD1	2.722975	5.278342	2.555367	7.85E-18
ZWINT	5.345488	11.98082	6.635329	8.01E-18
RPL37A	73.57308	142.7773	69.20419	8.12E-18
NCAPH2	8.330235	13.39633	5.06609	8.14E-18
NME3	17.98289	30.66005	12.67716	8.20E-18
EXOSC2	3.236844	5.064458	1.827613	8.25E-18
SLC27A2	62.8247	29.79568	-33.029	8.30E-18
TPM3	13.05492	20.27653	7.221614	8.34E-18
TRIR	70.18425	106.7769	36.59268	8.35E-18
RFWD3	2.025779	3.703138	1.677359	8.41E-18
CANT1	9.512608	14.70919	5.196581	8.43E-18
RTF2	12.6199	17.03061	4.410705	8.53E-18
LMBR1L	2.59534	3.937102	1.341763	8.58E-18
SORD	17.44102	8.130113	-9.31091	8.67E-18
LUC7L	2.568848	4.990373	2.421526	8.82E-18
SART3	3.459957	5.024099	1.564141	8.88E-18
WRAP53	1.195912	2.348394	1.152482	9.10E-18
PANK1	6.991862	4.007059	-2.9848	9.11E-18
RPL31	77.48793	130.001	52.51311	9.42E-18
CDC37	36.18226	49.11819	12.93592	9.49E-18
ECT2	1.82271	4.836109	3.013399	9.51E-18
MIS18A	3.748762	6.637231	2.888469	9.56E-18
FBRS	6.112716	8.862121	2.749405	9.77E-18
TESK1	7.596669	11.30331	3.706639	9.96E-18
FAM136A	9.97613	14.98612	5.009992	9.99E-18
VRK1	2.09967	3.733043	1.633373	1.01E-17
EZH2	2.110972	4.268601	2.157629	1.02E-17
CCDC97	5.884756	9.422794	3.538038	1.02E-17
RTL8C	33.60982	63.51051	29.90069	1.04E-17
ZNF335	2.474642	3.620508	1.145866	1.05E-17
AL035071.	1.389796	3.217043	1.827248	1.06E-17
NAA40	1.9486	3.719564	1.770963	1.13E-17
PUS1	3.787772	6.039888	2.252116	1.13E-17
RPS27A	98.72151	177.9447	79.22319	1.14E-17
C4BPA	587.3612	253.3116	-334.05	1.14E-17
VPS16	7.148472	9.892408	2.743936	1.14E-17
ST6GALNA	2.284522	5.18914	2.904618	1.15E-17
RPS19	151.3687	333.662	182.2933	1.17E-17
SLC31A1	23.87299	15.83488	-8.03811	1.19E-17
METTL5	5.884709	8.154882	2.270173	1.19E-17
TMEM44	0.93199	2.503945	1.571955	1.20E-17
UCKL1	8.970155	13.55646	4.586304	1.23E-17
CARD19	3.04261	4.973934	1.931324	1.25E-17
DAGLB	3.102696	4.302851	1.200155	1.25E-17
POLR3C	4.232309	6.669096	2.436786	1.29E-17
RMI2	1.330684	3.394688	2.064004	1.31E-17
SLC13A5	30.13543	11.73685	-18.3986	1.31E-17
MLST8	7.060873	9.982522	2.921649	1.32E-17
NCAPH	0.708748	1.884693	1.175945	1.36E-17
CHAF1A	3.043781	5.422558	2.378776	1.37E-17
PAXX	15.09067	29.44515	14.35448	1.38E-17
MTA3	1.68109	2.777099	1.096009	1.44E-17
RRP9	11.06072	17.01567	5.954952	1.44E-17
ACBD6	3.680864	5.695749	2.014885	1.46E-17

SNX7	5.009308	9.944878	4.93557	1.52E-17
MTHFD1	16.68444	8.190143	-8.4943	1.54E-17
AC003072	0.655233	1.720094	1.064861	1.55E-17
RPL28	71.53868	143.7405	72.20179	1.55E-17
HNRNPC	33.01544	43.47916	10.46372	1.55E-17
ARRDC1	5.266524	9.301452	4.034928	1.57E-17
HJURP	1.433156	3.752534	2.319378	1.57E-17
MMUT	35.72317	20.23539	-15.4878	1.57E-17
RAE1	3.694336	6.054452	2.360115	1.63E-17
PIDD1	2.405363	4.095912	1.690549	1.64E-17
NPRL3	4.180311	6.354568	2.174257	1.71E-17
PROS1	39.71601	24.99668	-14.7193	1.72E-17
CBX1	9.285719	15.71981	6.434088	1.74E-17
MARCKS	15.59132	32.44928	16.85796	1.74E-17
ZCRB1	3.409576	4.612149	1.202573	1.79E-17
DRG1	19.20575	26.88428	7.678527	1.80E-17
TTC1	20.58378	28.22145	7.637668	1.85E-17
HNRNPH3	8.169261	12.22058	4.051316	1.87E-17
RPL41	114.0765	185.3436	71.26716	1.93E-17
DPCD	3.020175	7.328008	4.307833	1.99E-17
MED25	3.707123	5.81603	2.108907	1.99E-17
TPGS2	2.671493	4.86416	2.192667	1.99E-17
SNORD99	0.984002	3.658116	2.674114	2.04E-17
UPF3B	2.974863	4.908471	1.933609	2.06E-17
F9	69.97586	24.07142	-45.9044	2.07E-17
G6PC3	3.982168	9.174781	5.192612	2.07E-17
EIF2B4	5.016835	6.846185	1.82935	2.07E-17
INTS11	6.491279	9.313892	2.822613	2.07E-17
CNOT2	2.395792	3.59693	1.201138	2.14E-17
PRPF19	33.49181	45.80293	12.31112	2.18E-17
CCT6A	32.75795	49.50828	16.75033	2.20E-17
KNG1	381.9867	196.4486	-185.538	2.23E-17
GATM	128.9854	63.4337	-65.5517	2.28E-17
GGPS1	2.60773	4.045628	1.437898	2.31E-17
VWA8	4.912258	3.109581	-1.80268	2.33E-17
SVBP	3.704291	7.306558	3.602267	2.37E-17
TRMT6	3.267234	5.090548	1.823313	2.45E-17
RBM19	3.932089	5.432234	1.500145	2.51E-17
PLK1	1.256438	3.588876	2.332438	2.55E-17
GLRX3	6.606667	9.747606	3.140938	2.58E-17
ZFAS1	0.855808	2.384329	1.528521	2.58E-17
EHMT2	4.220825	6.741389	2.520565	2.59E-17
C1S	343.6097	202.9017	-140.708	2.62E-17
EXO1	0.815382	2.001891	1.186508	2.69E-17
POLR1H	2.175923	3.544812	1.368889	2.72E-17
RCE1	3.484124	5.616231	2.132107	2.74E-17
PRMT2	2.997506	5.896734	2.899228	2.78E-17
SLC6A1	9.147183	4.181429	-4.96575	2.80E-17
SLC38A4	83.05254	35.94302	-47.1095	2.81E-17
ELOF1	8.437803	11.97877	3.540968	2.82E-17
GOLGA3	4.912294	7.3034	2.391106	2.85E-17
MORF4L1	17.06831	24.49976	7.431448	2.87E-17
IST1	7.975243	12.1218	4.146557	2.91E-17
TEAD4	1.738703	4.968042	3.229339	3.02E-17
KDELR1	71.8272	104.6714	32.84419	3.03E-17
HAUS5	2.370095	3.951941	1.581847	3.04E-17
AOX1	179.1154	67.74576	-111.37	3.07E-17
DHX37	2.870964	4.529609	1.658646	3.09E-17

SNAPC5	5.873517	8.072693	2.199176	3.10E-17
SNHG30	2.456125	4.462261	2.006136	3.21E-17
FUNDC2	3.656452	6.584185	2.927733	3.25E-17
VPS37C	3.460536	5.480613	2.020077	3.33E-17
EIF3K	47.24569	76.09502	28.84933	3.38E-17
SCAF1	15.20014	20.93571	5.735565	3.38E-17
MPZL1	8.400876	16.5063	8.105427	3.50E-17
KXD1	5.259399	7.375271	2.115872	3.57E-17
KAT2A	12.02919	21.09625	9.067056	3.58E-17
ANXA2	23.016	52.30301	29.28701	3.60E-17
GYG1	2.135838	3.540093	1.404254	3.61E-17
AC087741	0.831646	2.105713	1.274067	3.66E-17
TBCB	9.314764	17.77579	8.461031	3.69E-17
SSU72	12.53647	17.62023	5.083764	3.71E-17
RPF2	6.247493	9.382569	3.135077	3.88E-17
ADGRE5	4.1782	10.58115	6.402947	3.91E-17
SPC24	2.8924	6.312716	3.420316	3.95E-17
SNHG1	3.931374	8.552733	4.621359	4.07E-17
B4GALT3	8.364042	12.47725	4.113209	4.07E-17
TMEM81	2.233002	3.457824	1.224822	4.09E-17
AC116533	13.17662	31.20208	18.02546	4.10E-17
POP7	27.22922	42.52118	15.29196	4.11E-17
DYNLRB1	2.334164	6.138439	3.804276	4.17E-17
PGS1	2.16598	3.37976	1.21378	4.18E-17
DRAM2	8.094117	12.72404	4.629925	4.30E-17
MRPS21	44.36272	67.99058	23.62786	4.36E-17
CPED1	2.195577	0.930822	-1.26476	4.59E-17
THAP4	13.75975	20.34454	6.584799	4.66E-17
WDR54	1.04281	3.310406	2.267596	4.67E-17
TRADD	7.755113	12.38986	4.634744	4.73E-17
HMGB2	14.38846	29.69953	15.31107	4.88E-17
UBA52	82.70082	138.8996	56.19882	4.90E-17
CCT7	44.45836	61.86267	17.40432	4.93E-17
PIH1D1	15.52762	23.14772	7.620105	4.96E-17
YWHAB	19.50458	28.3474	8.842821	4.96E-17
CBFB	3.753311	6.876007	3.122696	5.04E-17
TRAPPC1	24.51891	39.88521	15.3663	5.06E-17
P3H4	2.5633	7.484618	4.921318	5.07E-17
CNOT11	14.69391	23.23587	8.54196	5.15E-17
FEN1	10.51299	19.3193	8.806311	5.16E-17
RHOC	22.96111	41.45842	18.49731	5.20E-17
TRA2B	4.85545	6.539634	1.684184	5.33E-17
TTC27	3.97653	5.655271	1.678741	5.33E-17
GATC	3.113452	4.190928	1.077476	5.36E-17
PSMD8	36.4407	48.94065	12.49994	5.46E-17
BTF3	43.52053	62.54745	19.02691	5.48E-17
RPS6	302.2367	549.4916	247.2549	5.50E-17
ACSM5	22.61256	9.225772	-13.3868	5.68E-17
PSMB2	25.27102	35.20754	9.936514	5.75E-17
TPX2	5.817468	14.1233	8.305829	5.80E-17
SDF2	10.41717	14.16546	3.748293	5.86E-17
ZNF263	3.298797	4.606594	1.307797	5.87E-17
TSC22D4	8.779225	14.34084	5.561611	5.90E-17
IKBKE	1.226058	3.184176	1.958118	5.99E-17
YWHAH	30.24011	48.23621	17.9961	6.23E-17
PRKD2	6.069678	9.868959	3.79928	6.23E-17
ACADSB	42.92682	22.31137	-20.6155	6.23E-17
CCT2	19.07385	29.9306	10.85675	6.57E-17

CIAO3	2.621833	3.902671	1.280838	6.70E-17
SRSF6	15.26316	20.69204	5.428886	6.70E-17
NDC80	1.509103	3.743903	2.2348	6.75E-17
ACTG1	451.9252	780.9912	329.0659	6.89E-17
BAK1	5.185945	10.44167	5.255727	6.97E-17
CCDC24	1.170794	3.182713	2.011919	7.01E-17
PSMB1	56.37359	85.48065	29.10706	7.02E-17
NUF2	1.251669	3.616898	2.365229	7.04E-17
RPS21	203.0681	440.0502	236.9821	7.05E-17
LINC0026:	28.46735	16.63213	-11.8352	7.14E-17
TARBP2	4.768677	7.470405	2.701728	7.14E-17
NCAPD2	3.650347	8.129666	4.479319	7.55E-17
NRM	4.799573	12.1658	7.36623	7.74E-17
CAPZB	6.400501	9.41096	3.010459	7.94E-17
FAM98B	2.086674	3.390422	1.303748	7.97E-17
TMEM60	12.97912	18.8593	5.880172	7.97E-17
OTUB1	9.563688	14.7636	5.199916	8.06E-17
DDX23	13.12673	18.1835	5.056773	8.16E-17
RPS15A	5.836416	12.57593	6.739515	8.32E-17
PCGF1	7.503428	9.896174	2.392746	8.36E-17
RPL24P2	1.122736	2.209206	1.08647	8.36E-17
GNL1	5.952367	8.478373	2.526005	8.37E-17
AC024060	2.083773	3.916729	1.832956	8.70E-17
MELK	1.615035	3.935375	2.320339	8.74E-17
C7orf50	10.01802	16.66642	6.648396	8.76E-17
RPLP0	245.8582	413.3294	167.4711	8.80E-17
YWHAZ	32.69257	59.39615	26.70358	8.90E-17
RANBP3	5.110788	7.116738	2.00595	8.96E-17
FAM241B	2.375407	5.961162	3.585754	9.11E-17
COMMD6	13.56858	22.38747	8.818884	9.33E-17
DOK1	0.991135	2.467575	1.47644	9.49E-17
TMSB10	544.3697	2360.982	1816.612	9.51E-17
NOC4L	9.363774	14.10657	4.742796	9.52E-17
CUEDC1	2.882332	6.586433	3.704102	9.53E-17
PSMC5	23.47777	35.40839	11.93062	9.56E-17
SKA3	1.014609	2.359918	1.345309	9.71E-17
BUB1	0.94379	2.256634	1.312844	9.76E-17
CDC25C	0.944258	2.228038	1.28378	1.01E-16
DLGAP4	4.6757	7.699977	3.024277	1.02E-16
AXIN1	3.037938	5.00108	1.963142	1.03E-16
XRCC5	33.40662	46.76764	13.36103	1.04E-16
DDX49	12.96987	19.57842	6.608546	1.04E-16
GDI1	15.60363	23.50791	7.904277	1.04E-16
ACIN1	8.194955	12.13811	3.943153	1.05E-16
RPS19BP1	14.46064	22.52848	8.067838	1.10E-16
FNTA	2.693734	4.29664	1.602906	1.11E-16
RAB35	7.140885	10.65121	3.510327	1.11E-16
NCDN	2.521497	5.212119	2.690622	1.14E-16
SP140L	2.062333	3.513052	1.450719	1.14E-16
ACTB	529.4006	836.8931	307.4925	1.14E-16
CCDC58	8.413697	12.07228	3.65858	1.15E-16
PHPT1	28.84235	52.22101	23.37867	1.19E-16
DNAJB2	13.66476	19.82536	6.160606	1.20E-16
NOL7	17.45387	24.70555	7.251673	1.20E-16
COLGALT:	14.05886	22.67255	8.613699	1.22E-16
ENSA	31.7492	47.31579	15.56659	1.22E-16
TXNDC9	5.06826	7.114095	2.045835	1.23E-16
CDC25B	9.105726	19.32293	10.2172	1.26E-16

BAAT	143.2853	69.94511	-73.3402	1.27E-16
KIF11	1.1528	2.938957	1.786156	1.28E-16
MCM6	6.152801	11.8335	5.6807	1.30E-16
RAD1	2.171508	3.318719	1.147211	1.31E-16
CCT3	79.72704	118.8242	39.09717	1.33E-16
H1-10	29.11802	47.6569	18.53889	1.34E-16
SAMD10	2.529569	4.304916	1.775347	1.34E-16
HDAC3	7.039865	9.293132	2.253267	1.35E-16
GPN2	3.65457	5.076863	1.422293	1.36E-16
CCAR2	4.826223	7.928695	3.102471	1.37E-16
FLOT1	45.60604	70.03095	24.42491	1.37E-16
TPI1	121.4298	182.5862	61.15636	1.38E-16
RIT1	3.100143	5.469017	2.368874	1.38E-16
EIF2B1	10.79175	14.21829	3.426538	1.39E-16
DPY30	10.88199	14.68603	3.804038	1.42E-16
GTF2F1	18.49088	26.79451	8.303631	1.42E-16
TIMM50	7.091851	10.82077	3.728922	1.44E-16
MED15	4.111723	6.881024	2.769301	1.44E-16
RABGGTB	7.464768	11.20517	3.740399	1.44E-16
RCC1	5.611861	9.626921	4.01506	1.49E-16
TSPAN3	7.415461	13.92194	6.50648	1.53E-16
HDGF	95.53871	131.226	35.68725	1.54E-16
CSE1L	16.17366	24.60348	8.429822	1.54E-16
COPS8	6.311871	8.643591	2.331719	1.55E-16
NCL	38.08193	54.23941	16.15748	1.57E-16
ORMDL1	5.523237	7.538289	2.015051	1.59E-16
METTL26	16.72717	28.48845	11.76128	1.60E-16
FAM32A	34.3152	43.59452	9.279323	1.60E-16
PIN1	6.917471	9.616959	2.699488	1.61E-16
FRG1CP	2.906355	5.077041	2.170686	1.63E-16
MZT1	3.310986	5.397105	2.08612	1.63E-16
TMEM216	2.075583	4.005252	1.929669	1.72E-16
NUP37	2.152903	3.417061	1.264158	1.73E-16
PTBP3	5.411204	9.274569	3.863365	1.79E-16
CRTC2	12.96295	18.44624	5.483284	1.81E-16
MCM5	6.68412	11.96702	5.282898	1.83E-16
CAP1	37.81752	60.20293	22.38541	1.84E-16
UTP14A	3.676711	5.558273	1.881562	1.85E-16
NKIRAS2	6.483547	8.974489	2.490941	1.92E-16
VHL	5.034133	7.522822	2.488689	1.94E-16
RPL18A	54.46356	112.7888	58.32523	1.96E-16
STOML2	45.0769	62.95908	17.88218	1.97E-16
CENPA	1.015673	2.923009	1.907336	2.05E-16
CHCHD1	19.64179	27.53547	7.893683	2.05E-16
TRIP13	0.990242	3.37995	2.389708	2.11E-16
ARL2	4.892544	12.95976	8.06722	2.12E-16
CYB5A	53.19046	32.68393	-20.5065	2.12E-16
PI4K2B	13.57446	8.979239	-4.59522	2.14E-16
MAP7D1	5.807763	12.06574	6.257979	2.18E-16
PSRC1	1.261035	3.061139	1.800104	2.23E-16
ZNF512	2.474893	4.510442	2.035549	2.24E-16
NENF	47.61451	78.58681	30.9723	2.25E-16
CIR1	7.793886	10.77891	2.985027	2.33E-16
F2	624.4767	372.5518	-251.925	2.34E-16
TATDN2	2.589863	3.963085	1.373222	2.37E-16
CTBP1	7.625004	10.71707	3.092067	2.38E-16
ARRDC1-/	2.15916	3.376091	1.216931	2.48E-16
FRG1	8.989584	12.74301	3.753424	2.48E-16

MIEN1	13.19074	19.86159	6.670852	2.48E-16
CCHCR1	5.1412	8.398629	3.257429	2.51E-16
CNOT6	2.295792	4.002004	1.706212	2.53E-16
RPL13A	271.5499	522.7943	251.2444	2.56E-16
RARS1	13.92139	18.90414	4.982751	2.57E-16
SRI	4.562551	8.629273	4.066723	2.62E-16
KIF18B	0.920883	2.553425	1.632541	2.63E-16
MGME1	6.592548	9.746228	3.15368	2.65E-16
NAA10	3.58062	6.765166	3.184546	2.68E-16
AC120053	1.288133	2.797598	1.509465	2.70E-16
PHIP	1.34202	2.400709	1.05869	2.71E-16
PTRHD1	3.30329	5.479194	2.175904	2.82E-16
SLC9B2	3.70069	2.003716	-1.69697	2.83E-16
SURF2	9.882276	19.82031	9.93803	2.93E-16
JPT2	14.34931	25.3357	10.98639	2.94E-16
SAC3D1	6.98416	11.82268	4.838525	2.99E-16
SPPL2B	3.939318	6.427464	2.488146	3.01E-16
PPP1R35	5.432588	8.714744	3.282156	3.10E-16
ARPC1A	8.44878	15.62855	7.179772	3.11E-16
SUB1	13.38734	22.47036	9.083021	3.24E-16
TP53RK	3.517242	5.02127	1.504028	3.27E-16
SNHG3	1.520734	4.74694	3.226205	3.30E-16
VTN	2170.436	1343.717	-826.719	3.31E-16
NOSIP	8.218003	13.77443	5.556427	3.40E-16
TMEM106	15.51518	27.22794	11.71276	3.53E-16
FMO4	12.68784	6.188211	-6.49963	3.60E-16
SELENOW	10.40422	22.4305	12.02627	3.60E-16
RNF114	13.04758	17.6219	4.574318	3.70E-16
TIMM23	36.16472	46.82427	10.65956	3.71E-16
DROSHA	3.658096	5.860837	2.202741	3.78E-16
C11orf80	0.893397	2.233721	1.340324	3.88E-16
CALM2	30.95018	48.55415	17.60396	3.89E-16
ZNF576	2.436372	3.506249	1.069877	3.97E-16
DAD1	144.312	191.2123	46.90031	4.02E-16
TLCD3A	1.309953	3.941656	2.631702	4.24E-16
PMF1	7.785203	13.19621	5.411006	4.28E-16
RPL7A	262.4476	456.2726	193.825	4.33E-16
GAS2L3	0.592122	1.643429	1.051307	4.35E-16
C1orf54	2.646967	4.950249	2.303281	4.45E-16
MFSD10	5.759803	20.31023	14.55043	4.52E-16
WDR46	15.89264	23.06443	7.171791	4.66E-16
HNRNPDL	17.42309	26.20662	8.783537	4.74E-16
FOXK2	2.865388	4.410472	1.545084	4.84E-16
STX10	19.10879	28.5428	9.434016	4.90E-16
RBM38	5.358325	10.28227	4.923948	4.92E-16
DCTN3	8.664776	12.312	3.647228	5.09E-16
ABCB4	21.65034	8.986998	-12.6633	5.20E-16
TBP	3.36944	4.848311	1.478871	5.20E-16
CYP4F3	41.90732	22.42036	-19.487	5.38E-16
ANKLE2	2.067074	3.609337	1.542263	5.39E-16
KCTD2	4.461689	6.863016	2.401327	5.43E-16
OXLD1	10.57954	17.91947	7.339928	5.49E-16
ZNF239	0.403595	1.517787	1.114192	5.50E-16
FAU	121.9395	189.3728	67.43325	5.61E-16
MAST2	3.287747	5.559725	2.271978	5.62E-16
AC115223	2.926682	4.920673	1.993991	5.66E-16
HMGXB3	5.225592	7.588877	2.363285	5.69E-16
GEMIN8	3.702018	6.062874	2.360856	5.86E-16

CIAPIN1	8.607987	12.84138	4.233395	5.89E-16
RPL34	77.67252	132.4636	54.79105	5.96E-16
U2AF2	21.74174	30.6944	8.952662	5.97E-16
CCDC124	28.77663	44.45177	15.67514	5.98E-16
IGBP1	13.64347	23.19916	9.555686	6.01E-16
GYS1	3.520559	6.689581	3.169022	6.06E-16
ARF5	33.78076	48.42861	14.64786	6.07E-16
CACYBP	10.32548	16.4874	6.161928	6.08E-16
CD276	9.57746	16.50149	6.924034	6.13E-16
TMEM69	10.53523	15.87371	5.338476	6.14E-16
PIGX	2.86371	4.342946	1.479235	6.16E-16
GARS1	4.729759	7.42691	2.697152	6.19E-16
SRP14	80.80924	104.2257	23.4165	6.41E-16
TUBB	113.7602	186.333	72.57281	6.43E-16
CAPZA1	19.88506	29.63482	9.749756	6.43E-16
PTGES2	6.844744	11.22074	4.375993	6.48E-16
LSM3	10.42011	14.54228	4.122169	6.51E-16
RRP12	3.156361	5.363397	2.207036	6.59E-16
EXOSC5	15.13037	24.28624	9.155871	6.63E-16
DRAP1	38.75584	54.78579	16.02995	6.65E-16
AC048341	0.712658	2.121311	1.408653	6.76E-16
FLOT2	30.06373	45.53288	15.46915	6.81E-16
SMIM26	9.81531	15.6597	5.844385	6.83E-16
ANAPC5	7.001557	9.393638	2.392081	6.84E-16
TRIP10	3.693615	8.85656	5.162945	6.88E-16
FBXL19	1.387796	2.811363	1.423567	6.88E-16
RPS4X	221.5707	391.9959	170.4252	6.94E-16
RACGAP1	2.642486	5.545801	2.903316	6.95E-16
C16orf91	8.686835	12.49696	3.810129	6.95E-16
TNFRSF14	7.107661	11.87291	4.765253	6.96E-16
ZNF121	2.16263	3.725446	1.562816	7.02E-16
COPE	26.6704	42.60061	15.93021	7.07E-16
CDC6	1.803433	4.288842	2.485409	7.14E-16
PEF1	23.17744	30.76843	7.590988	7.19E-16
AL390728.	2.668283	6.687722	4.019439	7.40E-16
AKAP8L	3.768791	5.671605	1.902814	7.71E-16
TMEM39B	3.390082	4.854772	1.464691	7.74E-16
NARF	4.392344	7.537128	3.144784	7.83E-16
NEK2	1.905374	4.535571	2.630198	8.12E-16
PRPF4	4.762724	6.878311	2.115587	8.15E-16
TCERG1	2.588252	4.084679	1.496427	8.16E-16
DNAL4	3.505205	6.940194	3.434989	8.22E-16
PRAF2	1.683276	3.291698	1.608422	8.42E-16
DEAF1	4.333204	7.09344	2.760236	8.50E-16
INAFM1	7.940345	15.34063	7.400287	8.50E-16
AL050341.	1.743535	3.289536	1.546001	8.57E-16
RAB32	18.26369	29.69418	11.43049	8.59E-16
SLC10A1	97.11489	31.21113	-65.9038	8.62E-16
CLTB	27.76096	47.51292	19.75196	8.63E-16
MTARC2	43.7106	29.31404	-14.3966	8.72E-16
RPAP3	3.012328	4.132383	1.120055	8.98E-16
THOC5	2.640532	4.010476	1.369945	9.04E-16
CASP4	4.855248	8.210272	3.355024	9.10E-16
CSNK2B	3.416612	6.442907	3.026295	9.38E-16
ACOT12	19.13113	7.741462	-11.3897	9.76E-16
NRAV	1.034229	2.253024	1.218795	9.77E-16
RUNDC1	2.072732	3.469944	1.397212	9.86E-16
VMP1	18.57236	31.75049	13.17812	9.95E-16

ADH1B	197.7349	65.38191	-132.353	9.96E-16
PTGES3	67.75333	87.89027	20.13694	9.99E-16
C8A	110.7538	54.36301	-56.3908	1.02E-15
PKM	10.69639	63.57996	52.88357	1.03E-15
HRG	533.0796	211.9299	-321.15	1.04E-15
MRPS23	5.143114	7.731736	2.588622	1.04E-15
ZNF629	2.495207	4.302157	1.80695	1.09E-15
GNPDA1	5.809532	10.34132	4.531789	1.10E-15
GTF2F2	3.865189	5.395455	1.530266	1.11E-15
EDF1	200.4578	317.7014	117.2436	1.11E-15
FAM222B	3.45347	6.209082	2.755612	1.11E-15
IP6K2	6.379671	9.561911	3.182239	1.12E-15
RFC5	3.058925	4.900333	1.841408	1.13E-15
TRAFD1	5.154491	8.447844	3.293354	1.15E-15
ACOT9	3.215647	5.032598	1.816951	1.17E-15
HNRNPAC	11.18003	16.61924	5.439208	1.17E-15
UBE2C	6.717381	22.31174	15.59436	1.18E-15
OST4	101.4486	136.8311	35.38249	1.28E-15
H3-3B	50.48854	75.08212	24.59358	1.28E-15
WDR45	4.553844	6.328591	1.774747	1.31E-15
RNF149	7.436068	10.95168	3.515611	1.31E-15
TRAPPC2E	1.508184	2.538907	1.030724	1.34E-15
DPH5	5.090789	7.288229	2.197439	1.36E-15
C20orf27	13.07821	23.09523	10.01702	1.37E-15
RPS15AP1	1.728687	3.255893	1.527205	1.41E-15
GRK6	5.709253	8.793497	3.084244	1.47E-15
RCCD1	2.246793	3.336988	1.090195	1.50E-15
ATP13A2	2.651153	5.520505	2.869352	1.50E-15
RASSF1	4.838985	7.03266	2.193675	1.56E-15
ETV5	1.928532	4.202527	2.273995	1.57E-15
RPS26	50.36666	84.01186	33.64521	1.59E-15
CNPY4	2.079027	3.548188	1.469161	1.61E-15
MYPOP	3.432212	5.146108	1.713896	1.62E-15
SLC6A12	11.83421	4.959239	-6.87498	1.66E-15
RPL6P27	5.17795	10.98536	5.807407	1.68E-15
LYRM4	4.894104	7.432256	2.538151	1.70E-15
SAAL1	2.664103	3.909751	1.245648	1.72E-15
NPM1	33.78783	54.87078	21.08296	1.72E-15
NIF3L1	7.608773	10.57418	2.965407	1.73E-15
BABAM2	6.610586	10.21063	3.600042	1.73E-15
AL021707.	0.801567	2.256006	1.454439	1.73E-15
MRPS26	47.19882	67.24401	20.04518	1.78E-15
CPNE1	16.00649	28.29721	12.29072	1.79E-15
PYCARD	4.220816	13.83869	9.61787	1.80E-15
SEPTIN2	15.70899	29.95992	14.25093	1.81E-15
FAM220A	4.358108	6.152069	1.793961	1.81E-15
TIMM22	3.34858	5.319702	1.971123	1.85E-15
ZRANB2	6.033131	9.518112	3.484981	1.86E-15
FANCE	1.14452	2.278737	1.134217	1.88E-15
EPHX2	33.66462	18.19705	-15.4676	1.88E-15
MPHOSPH	3.190177	4.980452	1.790275	1.90E-15
NLE1	2.3064	3.925814	1.619414	1.90E-15
DDX41	15.50538	20.47348	4.968102	1.91E-15
CDC42	7.39233	10.08813	2.6958	1.95E-15
VMA21	4.804887	7.444451	2.639564	1.97E-15
FOXJ3	3.225778	4.987862	1.762084	1.98E-15
DKC1	8.44062	13.0616	4.620979	1.98E-15
AADAC	211.9186	97.17987	-114.739	2.00E-15

S100A11	62.02739	321.9397	259.9123	2.03E-15
USP39	7.731402	10.53031	2.79891	2.03E-15
ALDH8A1	32.96018	16.85525	-16.1049	2.05E-15
PIGS	2.96008	6.357575	3.397495	2.12E-15
ZDHHC12	12.45982	18.02366	5.563846	2.12E-15
TMEM9	26.05132	38.95323	12.90191	2.15E-15
ACAT1	40.94605	26.008	-14.938	2.16E-15
AC018904	1.975007	3.892748	1.917741	2.18E-15
TMEM251	2.059103	3.125802	1.066699	2.20E-15
PRKAG1	9.08535	11.87934	2.793989	2.20E-15
NSFL1C	11.29132	15.03673	3.745408	2.22E-15
PGLS	13.7639	23.25877	9.494871	2.28E-15
POLD3	1.728591	3.16529	1.4367	2.28E-15
KLC2	1.954446	3.558527	1.604081	2.30E-15
LMNA	43.80394	67.5609	23.75696	2.30E-15
NAT10	6.456955	8.976502	2.519548	2.30E-15
TMEM115	18.25784	24.02983	5.771984	2.34E-15
SHKBP1	8.606478	15.05979	6.453308	2.37E-15
GPATCH3	5.755794	8.230237	2.474443	2.41E-15
AL139287.	2.104446	3.877324	1.772878	2.43E-15
IFT22	1.198877	2.297273	1.098396	2.45E-15
AP2S1	43.82435	66.54143	22.71708	2.47E-15
NFE2L3	0.730795	2.924623	2.193829	2.50E-15
TMEM50A	19.81111	27.87107	8.059958	2.50E-15
NELFE	23.40235	37.16928	13.76693	2.50E-15
IFI27L2	2.871687	8.72794	5.856253	2.51E-15
LGALS9	3.342027	10.0695	6.72747	2.53E-15
SGSH	3.309339	4.878192	1.568853	2.53E-15
MVP	22.93913	41.84824	18.90911	2.56E-15
SERPINC1	1947.006	972.8304	-974.176	2.56E-15
ATAD3A	5.497726	8.959468	3.461742	2.57E-15
TES	4.2811	9.96743	5.686331	2.63E-15
IMPDH1	2.37318	7.694678	5.321498	2.66E-15
TYW3	2.580036	3.707755	1.127719	2.72E-15
KIF23	0.666729	1.896554	1.229825	2.73E-15
A1CF	20.35997	13.38744	-6.97253	2.77E-15
NUP188	3.011258	4.93788	1.926622	2.82E-15
PLA2G15	2.786347	4.487761	1.701414	2.85E-15
RPA3	5.908828	9.269692	3.360864	2.90E-15
SMPD4BP	2.303712	4.105319	1.801607	2.90E-15
BLOC1S3	4.055537	6.13166	2.076123	2.92E-15
AUP1	61.09328	78.73954	17.64626	2.99E-15
ING5	2.224773	3.273514	1.04874	2.99E-15
PHF21A	1.362125	2.480075	1.11795	3.00E-15
UQCC3	2.09188	3.442815	1.350935	3.07E-15
SLC25A39	44.52126	62.18228	17.66101	3.08E-15
CDKN2AIP	10.16921	17.84364	7.674431	3.15E-15
SEPTIN9	15.33899	23.72836	8.389374	3.22E-15
TTLL1	1.341743	2.392919	1.051176	3.30E-15
CPSF4	4.302258	6.827327	2.525069	3.31E-15
PLXNA3	1.047266	2.724897	1.677631	3.32E-15
KMO	3.400386	1.259285	-2.1411	3.32E-15
TUBG2	3.013071	5.179677	2.166606	3.34E-15
SSRP1	11.66644	17.426	5.759562	3.38E-15
COPRS	28.28812	40.36439	12.07627	3.44E-15
RPL32	132.2666	236.2593	103.9928	3.53E-15
PSMB7	64.72159	88.59848	23.87688	3.65E-15
SORL1	6.062949	3.166558	-2.89639	3.66E-15

MCM7	14.60416	30.80712	16.20296	3.70E-15
KNTC1	0.865639	1.971934	1.106295	3.71E-15
FDX1	17.12542	11.92561	-5.19981	3.71E-15
YWHAE	63.08056	87.9564	24.87584	3.94E-15
MUS81	3.584511	4.931426	1.346914	4.04E-15
KANSL2	3.697627	5.638717	1.94109	4.08E-15
RAB5C	13.29185	17.86101	4.56916	4.10E-15
RETSAT	45.40763	28.30912	-17.0985	4.13E-15
TMEM101	10.79184	16.13847	5.346626	4.17E-15
FASTK	21.67601	30.33259	8.656584	4.32E-15
PAK4	5.217484	9.283625	4.066141	4.36E-15
RNF24	0.830372	1.881522	1.05115	4.38E-15
NUDC	39.3864	59.75859	20.37219	4.38E-15
PSMB3	105.4045	166.9306	61.52611	4.41E-15
KCTD10	2.541625	4.173083	1.631458	4.45E-15
PRR3	2.494423	3.779531	1.285108	4.57E-15
DAXX	14.77853	20.59182	5.813286	4.57E-15
HMGA1	26.22139	70.35018	44.12879	4.61E-15
BCL10	2.139208	3.730408	1.5912	4.63E-15
ARL6IP6	1.383191	2.517211	1.13402	4.63E-15
ZBED5	1.497265	2.697473	1.200208	4.70E-15
SH3GLB2	5.025873	9.411642	4.385769	4.74E-15
RUFY1	6.370909	8.980119	2.609209	4.75E-15
MOB3A	4.355979	8.768714	4.412735	4.85E-15
PDCL	2.682402	3.898815	1.216413	5.05E-15
DDX39A	11.88002	21.55958	9.679565	5.06E-15
OIT3	6.474809	2.929453	-3.54536	5.08E-15
NFKBIL1	11.24041	17.86681	6.626402	5.09E-15
ZMYND8	2.67491	4.665717	1.990807	5.11E-15
CDCA4	1.56136	3.303604	1.742245	5.13E-15
SMARCB1	2.736482	4.255125	1.518643	5.20E-15
MPV17	3.943521	6.815612	2.872091	5.26E-15
RBBP8	2.457195	4.718233	2.261037	5.35E-15
POLR2I	2.972008	5.953238	2.981231	5.42E-15
DNM1L	4.284082	6.593902	2.30982	5.42E-15
B9D2	1.448145	3.306928	1.858783	5.49E-15
MRPL22	4.694378	6.294382	1.600004	5.54E-15
SMARCA4	4.593512	7.611998	3.018485	5.57E-15
GPD2	1.424013	2.804557	1.380544	5.67E-15
HDAC7	2.283487	6.055633	3.772145	5.68E-15
UTP18	8.821857	12.48664	3.664786	5.73E-15
SELENOP	78.47481	43.48684	-34.988	5.78E-15
LPCAT4	1.073203	3.769586	2.696383	5.84E-15
BET1L	10.44297	13.67848	3.235509	5.86E-15
BCL2L12	6.727516	11.40156	4.674042	5.89E-15
AC010326	2.148152	3.877232	1.729079	5.97E-15
CARD16	1.892557	3.772056	1.879499	5.97E-15
USP21	3.890356	6.31085	2.420494	6.02E-15
VPS45	3.971682	5.813713	1.842031	6.03E-15
CSNK2A1	1.866773	2.926762	1.059989	6.24E-15
MACIR	0.624241	2.685014	2.060773	6.28E-15
C20orf96	2.525498	5.019083	2.493585	6.31E-15
NME1	4.629494	9.739138	5.109645	6.32E-15
RPSA	81.10189	156.4467	75.34476	6.36E-15
MBD2	6.843084	9.736353	2.893268	6.41E-15
DNAJC5	10.17109	14.91496	4.743875	6.49E-15
PHETA1	5.149681	8.952982	3.803301	6.64E-15
RAB11A	12.98267	19.18473	6.202056	6.72E-15

ACAA1	25.63344	15.29298	-10.3405	6.76E-15
YPEL3	10.38426	15.73608	5.351822	6.77E-15
RPL36	110.501	215.4434	104.9424	6.79E-15
GNB1	28.85207	40.32251	11.47043	6.96E-15
LMNB1	5.233582	10.40249	5.168911	7.05E-15
GTPBP3	3.04863	4.469387	1.420758	7.09E-15
TM4SF1	23.65714	79.34807	55.69093	7.11E-15
MRPL20	17.42108	24.3462	6.925128	7.28E-15
PTP4A2	15.14945	21.2596	6.11015	7.39E-15
FAM104B	3.012897	4.10218	1.089284	7.52E-15
ARHGEF18	4.114241	7.583244	3.469003	7.54E-15
IDH3B	25.9689	34.89545	8.926552	7.68E-15
TMEM250	9.814445	14.20064	4.386195	7.70E-15
MASTL	1.538215	2.569665	1.031449	7.71E-15
BX679664	1.03081	2.051095	1.020285	7.71E-15
DPF2	2.793577	3.982746	1.189168	7.73E-15
CDKN2D	2.141718	4.271974	2.130256	8.02E-15
PRR7	1.218933	2.797489	1.578555	8.03E-15
DEF8	4.244198	6.091249	1.847051	8.12E-15
PIP5K1C	3.155343	5.360164	2.204821	8.20E-15
ACOX1	31.70951	19.61429	-12.0952	8.22E-15
ZMIZ2	7.447295	12.34569	4.898396	8.32E-15
MRPL37	33.45644	45.71304	12.2566	8.56E-15
C12orf57	8.185701	15.4254	7.239699	8.70E-15
ADRM1	22.34245	29.37858	7.036135	8.74E-15
KPTN	2.370041	3.751667	1.381627	8.76E-15
CTDSPL	5.541287	10.36533	4.824048	8.79E-15
ATP6V1E1	25.73778	35.71447	9.976687	8.79E-15
LMNB2	4.079528	10.03777	5.958247	8.88E-15
CETN2	15.14142	24.55745	9.416031	9.12E-15
LDHD	40.91275	23.29616	-17.6166	9.14E-15
KIF20A	1.935305	4.640714	2.705409	9.24E-15
PCNA	40.47405	67.0415	26.56745	9.52E-15
SDHAP3	1.881464	4.241272	2.359808	9.70E-15
CDC7	0.924534	2.037795	1.11326	9.71E-15
NDUFB3	39.52542	53.24398	13.71856	9.77E-15
YTHDF2	11.55023	14.79266	3.242425	9.81E-15
RPL23A	40.54572	73.01845	32.47273	9.83E-15
ALB	17684.25	9689.569	-7994.68	1.02E-14
NR1H2	12.73729	17.22599	4.488699	1.02E-14
RPS16	183.6174	358.0377	174.4203	1.04E-14
SKA1	1.126395	3.193856	2.067462	1.04E-14
BZW2	5.640416	9.522913	3.882498	1.05E-14
ELF4	0.840298	3.329291	2.488993	1.07E-14
RPL10	149.6925	232.5688	82.87625	1.10E-14
SERPING1	375.3035	236.3165	-138.987	1.10E-14
LRRC42	12.04519	18.85954	6.814351	1.10E-14
RNASET2	0.913018	2.291663	1.378645	1.11E-14
MAX	4.09956	5.746741	1.647181	1.13E-14
S100A10	112.1446	227.258	115.1134	1.13E-14
PRPF38B	3.769587	6.176752	2.407165	1.14E-14
UBE2N	7.485797	9.946636	2.46084	1.14E-14
CCDC174	3.825659	4.933896	1.108236	1.14E-14
GEMIN6	3.204508	4.381177	1.176668	1.16E-14
MVB12A	7.242131	11.00547	3.763341	1.16E-14
PACS1	3.9097	8.215323	4.305623	1.17E-14
TSN	13.2394	19.03343	5.794028	1.19E-14
KRI1	4.877549	7.012858	2.135309	1.23E-14

EIF2S3	37.10784	55.9192	18.81136	1.24E-14
ZCCHC24	7.101722	4.641125	-2.4606	1.25E-14
GNAI3	6.020259	8.782221	2.761962	1.25E-14
RPS3	84.17121	157.7359	73.56464	1.26E-14
KMT5C	1.608632	2.662965	1.054333	1.26E-14
GNB2	50.50902	73.44096	22.93194	1.28E-14
DOHH	5.719601	8.03019	2.310589	1.30E-14
LPCAT1	4.296477	12.27303	7.976552	1.32E-14
ELOB	103.0088	175.4056	72.39673	1.32E-14
UPP1	2.803357	5.497027	2.69367	1.33E-14
MPDZ	5.705419	2.823044	-2.88238	1.33E-14
FTSJ3	9.739364	13.61287	3.873504	1.35E-14
FMO3	130.3272	56.25075	-74.0765	1.39E-14
PML	2.567105	4.332071	1.764967	1.39E-14
RABEP2	2.92176	5.36008	2.438321	1.39E-14
USP1	5.087121	8.652781	3.56566	1.40E-14
FANCF	3.233229	4.897422	1.664193	1.41E-14
KIAA1522	10.77813	23.20187	12.42374	1.42E-14
ARL3	3.460609	5.132844	1.672235	1.46E-14
TTF1	1.86381	3.078606	1.214796	1.47E-14
SNX8	4.598416	7.328406	2.72999	1.49E-14
GRN	84.9325	131.835	46.90255	1.50E-14
SMU1	5.771156	7.686222	1.915067	1.52E-14
C11orf68	16.49673	22.21552	5.71879	1.56E-14
CMTM3	3.78687	10.55621	6.769337	1.57E-14
AGBL5	3.387946	5.412754	2.024808	1.58E-14
IMP4	15.97629	20.67119	4.694899	1.60E-14
QARS1	28.86243	39.18409	10.32166	1.60E-14
SF3B2	15.6921	20.48071	4.788611	1.62E-14
ORAI2	0.568106	2.133508	1.565402	1.62E-14
PI4KB	9.98594	15.39206	5.406115	1.63E-14
FLYWCH2	8.249697	13.83153	5.581832	1.69E-14
PCK2	116.8687	62.50037	-54.3683	1.69E-14
EVA1A	21.32552	12.72918	-8.59634	1.70E-14
ACOT2	15.93588	9.77399	-6.16189	1.70E-14
CDPF1	4.930553	7.307011	2.376458	1.74E-14
AC005912	19.8065	49.73397	29.92747	1.76E-14
VPS25	20.34608	27.39871	7.05263	1.76E-14
TEAD2	3.87704	10.28176	6.404725	1.79E-14
AC005332	3.79725	6.536838	2.739588	1.79E-14
PON3	40.48924	21.18323	-19.306	1.79E-14
RPS27AP1	4.225069	8.670838	4.445769	1.79E-14
RAD51AP1	1.219024	2.708887	1.489863	1.80E-14
BUB3	6.518898	9.11459	2.595692	1.80E-14
STX8	4.653059	6.995071	2.342013	1.82E-14
NRSN2	3.240359	9.691976	6.451618	1.82E-14
TMEM51	2.083642	8.183546	6.099904	1.83E-14
PPOX	3.642846	5.258026	1.615179	1.84E-14
SH3BGRL3	53.58782	111.4411	57.85328	1.91E-14
DUSP22	4.844236	7.381731	2.537495	1.92E-14
RPS5	106.7609	192.3794	85.61857	1.93E-14
NDUFA12	20.49386	26.89355	6.399697	1.94E-14
EXOC3	5.308591	7.523605	2.215014	1.94E-14
NSUN5P1	1.297164	2.54893	1.251766	2.00E-14
CNOT10	3.854078	5.352358	1.49828	2.04E-14
MAGEF1	12.75239	20.05751	7.305118	2.05E-14
MIR25	1.956275	4.387652	2.431377	2.07E-14
AR	8.290437	3.759313	-4.53112	2.12E-14

IQCE	1.454044	3.223849	1.769805	2.12E-14
C2orf76	2.54451	3.580922	1.036412	2.14E-14
TMEM165	3.346914	5.730298	2.383384	2.15E-14
PON1	132.7801	69.01763	-63.7624	2.15E-14
CIDECP1	1.944759	2.979051	1.034292	2.18E-14
CEP55	0.702109	2.86737	2.165261	2.21E-14
GGCX	23.27565	16.03016	-7.24549	2.22E-14
SLC10A3	4.994379	10.05723	5.062849	2.23E-14
PPIC	11.06817	17.5523	6.484136	2.27E-14
CYP2C8	144.3874	55.76843	-88.619	2.28E-14
TTC22	0.983565	2.487861	1.504297	2.28E-14
RGN	60.01761	34.31888	-25.6987	2.29E-14
CCDC167	35.53109	60.99386	25.46277	2.29E-14
MAP2K2	20.28257	30.07564	9.793069	2.33E-14
POLL	3.208372	4.534073	1.325701	2.35E-14
TAF15	18.0851	23.68295	5.59785	2.37E-14
SNRNP27	5.995004	7.915197	1.920193	2.38E-14
MLLT6	3.203079	5.8847	2.681621	2.38E-14
HECTD3	7.652847	11.00758	3.354733	2.40E-14
SOGA1	1.064339	2.09552	1.031181	2.40E-14
RABAC1	3.096935	5.709373	2.612438	2.42E-14
IFT57	1.731416	4.439925	2.708509	2.45E-14
SEPSECS	6.352241	4.391733	-1.96051	2.45E-14
MSI2	1.174426	2.283656	1.10923	2.45E-14
TTK	0.771039	1.967478	1.196439	2.49E-14
CDK2AP1	6.369688	10.94177	4.572079	2.49E-14
BUD13	5.677769	7.653773	1.976003	2.49E-14
MCM4	5.705694	10.4916	4.78591	2.50E-14
USF2	19.46513	26.47098	7.005852	2.50E-14
PLP2	11.33214	33.62627	22.29413	2.56E-14
CDK1	2.803097	6.535366	3.732268	2.60E-14
ACOT7	6.802462	11.475	4.672535	2.65E-14
PRKCD	4.824951	8.360916	3.535965	2.66E-14
EPS8L3	3.4827	12.0062	8.523498	2.74E-14
CDK7	5.612426	8.517422	2.904997	2.81E-14
TMEM87A	3.249591	5.838209	2.588618	2.82E-14
MYO1B	28.50487	18.8768	-9.62807	2.83E-14
YWHAQ	38.36342	59.25571	20.8923	2.83E-14
CYRIB	3.271533	5.913509	2.641976	2.86E-14
RCN2	2.300481	3.799218	1.498737	2.86E-14
CFHR4	22.06564	6.364199	-15.7014	2.91E-14
ZNF408	5.314204	7.342075	2.027871	2.95E-14
PPME1	3.083314	4.379929	1.296615	2.95E-14
TMEM192	4.677478	3.2597	-1.41778	2.95E-14
PINK1	4.951902	3.05013	-1.90177	2.97E-14
PLXNA1	1.620597	3.652904	2.032307	3.09E-14
SPTLC1	3.807544	6.102235	2.294691	3.11E-14
INSIG1	196.0015	81.28781	-114.714	3.14E-14
GAS5	17.35144	35.30016	17.94872	3.18E-14
LEMD2	5.898465	8.232734	2.334269	3.20E-14
ABHD11	6.117802	11.76447	5.646669	3.21E-14
AKAP8	4.814373	6.563986	1.749613	3.27E-14
SCAMP4	6.261247	9.527307	3.26606	3.28E-14
NDRG3	6.641105	12.68247	6.041367	3.29E-14
VANGL1	1.052868	2.114625	1.061757	3.35E-14
HSBP1L1	6.71226	10.57891	3.866647	3.38E-14
NFU1	10.14557	13.72663	3.581058	3.41E-14
TUSC2	10.01493	13.79991	3.784974	3.42E-14

RBM39	10.14807	14.05556	3.907495	3.46E-14
PXN	2.486486	3.808358	1.321872	3.51E-14
VPS37B	1.264861	2.854758	1.589898	3.52E-14
TRMT2A	8.140529	11.19636	3.055834	3.63E-14
COX8A	99.45131	147.4238	47.97246	3.65E-14
TSR3	23.6658	33.03356	9.367767	3.68E-14
PTPN2	1.980316	3.40053	1.420214	3.71E-14
POLD1	2.87299	4.868124	1.995134	3.72E-14
EFNA3	0.739361	1.922742	1.183381	3.76E-14
GTF3C1	4.45918	6.362275	1.903095	3.78E-14
DNAJC10	1.981458	3.903278	1.921821	3.81E-14
LRRC8B	0.677661	1.818144	1.140484	3.82E-14
SCAMP2	15.2677	19.74189	4.47419	3.85E-14
SARDH	23.12229	13.48618	-9.6361	3.89E-14
SRM	25.40583	42.75625	17.35042	3.91E-14
B4GALT7	5.808113	8.028399	2.220286	3.94E-14
HOMER3	0.990245	3.242074	2.251829	3.98E-14
MICALL2	1.939499	4.637881	2.698382	3.99E-14
BUB1B	0.753135	1.891645	1.13851	4.06E-14
CRNKL1	5.800518	7.691393	1.890875	4.14E-14
MYO9B	5.340732	7.691416	2.350684	4.19E-14
ETV6	2.452399	4.390629	1.938231	4.25E-14
KPNA2	6.541817	11.75982	5.218008	4.32E-14
SIRT7	3.032698	4.807962	1.775265	4.34E-14
KIF4A	1.649711	4.205835	2.556125	4.35E-14
COPS6	29.13357	40.56826	11.43469	4.36E-14
KHDC4	4.228252	7.856869	3.628617	4.36E-14
ATG13	9.482795	12.13548	2.652686	4.37E-14
METTL23	7.073744	10.29308	3.219341	4.42E-14
UTP4	4.002344	6.4506	2.448255	4.43E-14
RNPEPL1	11.25931	15.50863	4.249319	4.55E-14
TJAP1	4.011767	5.680689	1.668922	4.62E-14
GCA	1.660667	3.349478	1.688812	4.68E-14
RNF125	2.097645	1.017484	-1.08016	4.68E-14
GLYAT	30.30538	11.56344	-18.7419	4.88E-14
PIPOX	95.74726	55.63941	-40.1079	5.02E-14
DNAAF5	3.203948	4.821841	1.617893	5.09E-14
RPL21	55.14808	93.97055	38.82247	5.10E-14
STIP1	22.35176	34.04066	11.6889	5.10E-14
DTX3	1.243804	4.125354	2.88155	5.11E-14
PCCB	17.77089	11.66661	-6.10428	5.17E-14
UROD	19.45806	26.16639	6.708336	5.23E-14
ANXA5	51.85786	95.07938	43.22152	5.24E-14
SMPD4	4.942748	7.22775	2.285002	5.33E-14
NOP16	4.60531	7.230536	2.625226	5.46E-14
ACTR1A	14.92691	19.80642	4.879504	5.47E-14
NDUFB11	99.95112	156.2351	56.28396	5.51E-14
RPL35P1	1.048954	2.144717	1.095763	5.67E-14
UBL5	52.87405	74.67552	21.80146	5.77E-14
KRT18	147.0367	301.5647	154.5281	5.85E-14
KPNB1	11.12615	16.42109	5.294944	5.85E-14
TMUB2	8.859376	12.22051	3.361132	6.08E-14
UQCR11	5.696693	9.610261	3.913567	6.13E-14
RBM42	33.41382	51.91362	18.4998	6.17E-14
ATAT1	1.571894	2.91202	1.340126	6.18E-14
WAC-AS1	4.624661	7.336954	2.712293	6.20E-14
RPL10A	232.1099	360.4198	128.3098	6.21E-14
TMEM185	3.790814	6.055175	2.264362	6.27E-14

SUPV3L1	6.230726	8.114868	1.884143	6.35E-14
TADA3	13.91881	18.75971	4.840898	6.51E-14
NSMCE1	11.69453	17.23755	5.543016	6.53E-14
PIGBOS1	5.034651	7.2049	2.170249	6.54E-14
SNX5	7.322692	10.07401	2.75132	6.69E-14
VBP1	13.28794	18.78233	5.494387	6.79E-14
SNHG4	0.436143	1.495298	1.059155	6.81E-14
C1orf122	10.18368	15.90143	5.717752	6.87E-14
AL049840.	4.607679	7.344156	2.736477	6.88E-14
REEP4	4.815211	10.99028	6.175073	6.90E-14
DBN1	3.800695	14.76835	10.96765	6.90E-14
TMEM91	0.827449	1.893338	1.065889	7.00E-14
ZNF316	3.196439	4.495479	1.29904	7.11E-14
ERAL1	20.29665	26.91071	6.614068	7.11E-14
TMEM43	7.478924	13.03058	5.551657	7.14E-14
NUDT2	14.78772	21.81184	7.024128	7.17E-14
ZDHHC7	5.084005	10.51154	5.427539	7.17E-14
HNRNPH1	14.66229	21.84049	7.178205	7.35E-14
DTL	1.758492	3.845726	2.087234	7.43E-14
PPP1R37	3.523649	6.973185	3.449536	7.61E-14
KCTD5	5.778715	8.242488	2.463773	7.71E-14
LARP4B	3.424053	5.086397	1.662344	7.84E-14
EXOSC9	2.708056	4.130618	1.422562	7.87E-14
IGSF3	1.513155	5.139339	3.626185	7.89E-14
PRPF3	5.529378	9.412826	3.883448	7.98E-14
WASF2	9.559402	14.24952	4.690119	8.20E-14
STK17A	2.75385	5.477877	2.724027	8.35E-14
NRDC	14.8103	19.35735	4.547049	8.39E-14
GCN1	8.417934	11.83759	3.419651	8.39E-14
MAF1	32.94332	51.71704	18.77373	8.42E-14
PACS2	2.517121	3.739146	1.222025	8.54E-14
RBM6	5.283575	7.952927	2.669351	8.62E-14
TBC1D22A	6.102498	8.695683	2.593186	8.62E-14
RAVER2	0.83492	2.4229	1.58798	8.64E-14
ZNF511	2.401397	3.611311	1.209914	8.65E-14
PSMD11	10.72907	14.15082	3.421753	8.85E-14
PAF1	21.62864	29.04384	7.415195	8.88E-14
ACTR5	2.94407	4.340674	1.396604	8.88E-14
RPL26	6.131248	13.23635	7.105101	9.01E-14
NPM3	11.93237	23.4101	11.47773	9.06E-14
PUSL1	2.420956	4.168068	1.747112	9.09E-14
SLC35D1	13.52716	8.76924	-4.75792	9.11E-14
PDLIM7	3.973772	10.03279	6.059016	9.62E-14
MRPL18	28.69862	39.73086	11.03224	9.65E-14
DEDD2	7.819577	11.38228	3.562707	9.86E-14
AFM	84.37366	39.25782	-45.1158	9.90E-14
CHMP4C	4.457514	8.30717	3.849656	9.98E-14
CNIH4	6.413985	9.614672	3.200687	1.05E-13
PSMA5	16.33658	22.59333	6.256746	1.05E-13
CYP4A22	27.09869	11.42265	-15.676	1.06E-13
CCDC9	5.532705	7.619513	2.086808	1.06E-13
GRWD1	6.313536	8.266645	1.953108	1.07E-13
CENPF	1.577965	3.719843	2.141878	1.10E-13
PSMA7	82.94219	113.1935	30.25131	1.10E-13
FKBP8	81.51008	114.7185	33.20846	1.11E-13
MFSD14B	5.318875	8.333627	3.014752	1.11E-13
CYP8B1	104.6127	36.8447	-67.768	1.12E-13
FAM189B	6.216435	9.809944	3.593509	1.12E-13

HCFC1R1	15.55464	29.04828	13.49364	1.13E-13
SKP1	9.602515	12.47039	2.867877	1.13E-13
NMI	5.089942	7.457122	2.36718	1.14E-13
POLE4	9.218602	15.01421	5.795605	1.15E-13
ARMCX6	1.0844	2.696038	1.611637	1.16E-13
EIF4A3	9.205943	12.88949	3.683551	1.16E-13
RBBP7	9.951085	13.87731	3.926223	1.17E-13
SH3GLB1	7.071679	10.80905	3.737367	1.18E-13
PPP1R9B	5.894365	10.50849	4.614128	1.19E-13
GBA3	13.20589	4.870561	-8.33533	1.22E-13
ANLN	1.387232	3.649477	2.262245	1.25E-13
ERP29	61.18269	80.94937	19.76669	1.25E-13
RBM26	2.364785	3.438593	1.073807	1.26E-13
CFB	9.017671	4.732922	-4.28475	1.27E-13
BLMH	3.892676	7.83644	3.943765	1.27E-13
EFHD2	18.41757	30.80902	12.39145	1.28E-13
PSMB4	124.3914	178.2815	53.89004	1.28E-13
BHMT2	107.6876	66.59509	-41.0925	1.28E-13
CCT8	27.56196	37.55993	9.997965	1.29E-13
ZBTB45	2.9293	4.261558	1.332258	1.29E-13
EIF3G	27.49338	37.87963	10.38625	1.32E-13
DAPK3	10.02885	15.29717	5.268319	1.33E-13
ADPGK	4.048343	6.316407	2.268064	1.35E-13
CYBC1	7.196484	10.72315	3.526662	1.36E-13
CDC123	12.16645	17.329	5.162551	1.36E-13
CCNQ	7.772587	11.45965	3.687063	1.37E-13
IDH3G	15.3201	22.42711	7.107015	1.38E-13
ARMC7	5.09811	7.918333	2.820223	1.39E-13
TAF3	1.78793	2.810897	1.022967	1.41E-13
PLGLA	2.864992	0.951976	-1.91302	1.43E-13
LST1	2.217192	4.783052	2.565861	1.45E-13
DYRK2	1.537373	2.970694	1.433321	1.45E-13
SUDS3	4.619556	6.654026	2.03447	1.46E-13
CYP4A11	95.87871	45.62061	-50.2581	1.49E-13
CLASRP	5.278611	8.608433	3.329823	1.51E-13
PNMA1	4.239766	9.253846	5.014081	1.52E-13
KDM4A	2.356109	3.391629	1.035519	1.52E-13
GNPNAT1	5.961933	4.021341	-1.94059	1.52E-13
KIAA0930	7.389569	12.54404	5.154473	1.53E-13
B3GAT3	18.45663	26.79133	8.334695	1.53E-13
SC5D	15.55626	8.378688	-7.17757	1.54E-13
ORC2	2.010506	3.130177	1.119671	1.55E-13
AC008780	2.465489	1.114092	-1.3514	1.56E-13
PARN	3.344416	4.534395	1.189979	1.60E-13
XPO5	3.716173	5.398547	1.682374	1.61E-13
GTF3C5	10.86111	16.02457	5.163458	1.65E-13
KBTBD2	5.949446	7.944393	1.994947	1.66E-13
CCDC14	1.363162	2.457008	1.093847	1.67E-13
RPL13	144.7293	292.4489	147.7196	1.68E-13
SF3B3	6.321942	9.216957	2.895014	1.69E-13
CNTROB	2.755739	4.503307	1.747567	1.69E-13
JTB	7.9328	15.13681	7.204011	1.70E-13
RPS2P46	2.229717	4.255568	2.025851	1.70E-13
CD151	43.48349	75.46222	31.97873	1.72E-13
SLC25A24	0.664359	2.229002	1.564643	1.74E-13
RANGAP1	21.38753	36.50516	15.11763	1.74E-13
RPL22	13.00185	18.37581	5.373964	1.75E-13
SHMT1	35.98363	18.73661	-17.247	1.79E-13

SSR4	33.75057	53.05876	19.30819	1.81E-13
MZT2B	11.69195	23.57984	11.88789	1.83E-13
CDK10	7.819767	11.40695	3.587188	1.83E-13
SFI1	1.020157	2.036923	1.016767	1.89E-13
FAM193B	4.338972	7.33626	2.997287	1.92E-13
EHBP1L1	4.246754	7.768382	3.521628	1.94E-13
PSPC1	3.518662	5.149547	1.630886	1.94E-13
VAMP8	86.28743	133.1765	46.88902	1.97E-13
TAGLN2	158.1175	283.0874	124.9699	1.99E-13
TOMM22	28.85119	39.69931	10.84812	2.04E-13
SMG9	2.192171	4.472684	2.280513	2.05E-13
PRDM4	2.634958	4.256808	1.62185	2.08E-13
QDPR	42.29118	27.3911	-14.9001	2.15E-13
TLNRD1	2.671325	4.221977	1.550653	2.17E-13
EIF5A	62.96643	93.18973	30.2233	2.23E-13
DXO	5.127397	7.768695	2.641299	2.25E-13
RNASEH2(	4.21758	6.692747	2.475167	2.26E-13
SRC	4.332336	8.981028	4.648692	2.27E-13
FMO5	55.08259	30.40102	-24.6816	2.33E-13
ATP6AP1	23.07064	31.80911	8.738463	2.33E-13
CCDC6	4.10082	7.229761	3.128941	2.34E-13
PHF5A	14.43009	19.53965	5.109557	2.38E-13
TULP3	3.658401	6.12082	2.462419	2.40E-13
TSPO	28.46106	63.42092	34.95986	2.43E-13
LTV1	5.01914	7.237804	2.218665	2.44E-13
ASB1	1.818017	3.01411	1.196093	2.45E-13
SNAPIN	17.92473	23.98975	6.06502	2.47E-13
ITIH1	396.5226	241.2676	-155.255	2.51E-13
SLC1A5	3.797739	15.73245	11.93471	2.52E-13
MARS1	8.996329	12.64065	3.644319	2.53E-13
ZCCHC10	3.856605	4.989568	1.132964	2.55E-13
CIAO2B	54.04483	73.862	19.81717	2.55E-13
NAT14	2.530173	6.861589	4.331416	2.57E-13
SGTA	9.002802	11.73096	2.728156	2.57E-13
NIP7	3.239219	4.57832	1.339101	2.63E-13
DLG3	0.633271	2.746267	2.112997	2.65E-13
ANKS6	1.156698	2.188813	1.032115	2.65E-13
EMC9	5.733484	8.503572	2.770088	2.66E-13
CCDC102/	1.262996	2.992079	1.729083	2.69E-13
COG7	3.229728	4.443529	1.213801	2.71E-13
TMC6	1.351727	4.77192	3.420193	2.73E-13
NEDD1	1.705581	2.734129	1.028548	2.75E-13
NFKB2	12.53343	19.75134	7.217907	2.78E-13
LMAN2L	8.013004	10.93474	2.921737	2.80E-13
AMD1	3.532834	5.238323	1.705489	2.83E-13
FBXW4	8.190872	11.69809	3.507215	2.84E-13
UBAC1	8.617997	11.62829	3.01029	2.86E-13
MRPS5	6.973053	8.941956	1.968903	2.88E-13
EEF1B2	108.5842	173.6711	65.0869	2.88E-13
HCFC1	6.422148	9.19394	2.771792	2.89E-13
SOX4	5.697747	18.37994	12.6822	2.90E-13
MRPL23	11.23708	17.69856	6.461479	2.90E-13
TMA16	2.872377	4.238248	1.365871	2.93E-13
NCK2	5.095363	14.47075	9.375384	2.94E-13
C3	1468.202	995.4518	-472.75	2.95E-13
PGP	4.794968	8.939632	4.144663	2.96E-13
CD58	3.506675	8.635542	5.128867	2.99E-13
AP2A2	7.43735	9.743806	2.306456	3.03E-13

ZSCAN16	1.983533	3.356643	1.37311	3.19E-13
CYP2C9	129.0379	53.90842	-75.1295	3.21E-13
FAM214B	1.613093	2.653509	1.040417	3.21E-13
RPN2	126.7701	164.824	38.05392	3.22E-13
PDCD7	3.763229	5.077176	1.313947	3.25E-13
NBDY	2.555509	5.312353	2.756844	3.25E-13
EIF3M	8.855143	11.77949	2.924344	3.34E-13
PCYOX1	28.63088	19.71282	-8.91806	3.36E-13
RECQL5	3.079673	4.314164	1.234491	3.39E-13
PIP4K2B	7.328553	10.22797	2.899421	3.39E-13
FUT11	1.400513	2.410354	1.009841	3.41E-13
MRPL9	9.910089	14.70523	4.795144	3.46E-13
ATN1	11.09736	20.29153	9.194169	3.47E-13
TTC38	53.5329	35.73404	-17.7989	3.47E-13
FAAP20	2.613258	3.822978	1.20972	3.49E-13
PPP1CA	26.47788	35.67174	9.193866	3.51E-13
GATD1	7.738673	11.54236	3.803691	3.52E-13
SEPHS1	12.84721	17.22839	4.381175	3.53E-13
DEDD	9.306901	12.50433	3.197426	3.55E-13
SELENOF	46.67691	61.2233	14.54639	3.57E-13
CDK19	1.656726	3.383942	1.727216	3.58E-13
EIF5B	10.52994	14.14781	3.617864	3.61E-13
GTPBP4	5.331618	7.901193	2.569575	3.63E-13
NADK2	37.18549	23.31829	-13.8672	3.63E-13
SNORD10	19.67978	48.52204	28.84226	3.70E-13
PARP8	1.046449	2.058672	1.012223	3.77E-13
RNF113A	10.79111	15.20576	4.414652	3.78E-13
CAD	3.641482	6.180828	2.539345	3.89E-13
IPO13	6.099231	8.090238	1.991007	3.89E-13
XPNPEP1	4.069135	5.547059	1.477924	3.92E-13
RNF19B	4.733824	7.819924	3.0861	3.98E-13
AC132872	1.480656	3.145234	1.664578	4.02E-13
UCK2	2.712914	4.611523	1.898609	4.06E-13
STK38	9.449746	14.39217	4.942426	4.06E-13
PRPF39	2.36928	3.681583	1.312302	4.09E-13
LDB1	8.642164	14.75677	6.114608	4.09E-13
KLF15	38.93366	23.46413	-15.4695	4.10E-13
PWWP2B	4.695849	9.761881	5.066032	4.14E-13
SLC38A1	3.079707	9.804353	6.724646	4.23E-13
SPRED1	1.364787	3.086815	1.722029	4.26E-13
ERCC1	5.226798	8.438317	3.211519	4.32E-13
FHOD1	1.388276	2.884952	1.496675	4.34E-13
TM2D1	2.439541	3.520692	1.08115	4.41E-13
SLC16A2	20.40009	9.97563	-10.4245	4.41E-13
SMIM10L1	4.333147	5.941963	1.608815	4.42E-13
FBP1	84.99116	35.09067	-49.9005	4.56E-13
CD27-AS1	1.734997	2.978834	1.243837	4.57E-13
MFAP1	9.230387	12.22637	2.995982	4.57E-13
HID1	1.736489	6.046818	4.310329	4.64E-13
PRDX3	72.93288	55.81007	-17.1228	4.64E-13
YRDC	6.144627	8.130331	1.985704	4.67E-13
FAM98C	3.007934	4.085243	1.077309	4.67E-13
PAFAH1B2	7.843342	10.9826	3.139253	4.67E-13
ALKBH4	3.811057	5.088408	1.277351	4.68E-13
PPARA	7.858779	4.974652	-2.88413	4.71E-13
PTPN18	7.73818	10.44419	2.706009	4.71E-13
RPL10AP6	1.928538	3.66704	1.738502	4.73E-13
RPL13AP2	2.006503	4.91184	2.905337	4.79E-13

RGS10	3.670409	9.347411	5.677001	4.80E-13
MKRN1	11.05655	14.46655	3.410004	4.82E-13
C4B	42.25839	23.48862	-18.7698	4.86E-13
STX3	2.010854	4.256094	2.24524	4.94E-13
HARS2	5.598058	7.706404	2.108346	4.95E-13
XRCC1	6.463257	11.00681	4.543558	5.01E-13
STX16	7.209145	10.58117	3.372028	5.02E-13
MIR210HC	0.782817	2.445737	1.662921	5.07E-13
DYNC2LI1	1.83516	3.227162	1.392002	5.08E-13
CHMP2A	57.53664	83.3589	25.82226	5.09E-13
SWI5	6.614012	10.46686	3.852845	5.19E-13
CCDC93	2.245358	3.450511	1.205154	5.23E-13
FBXW9	1.904567	3.407329	1.502762	5.25E-13
HIGD2A	66.15804	97.97981	31.82177	5.28E-13
AKT1S1	10.5319	14.76487	4.232968	5.32E-13
CYB561	5.747777	11.61617	5.868395	5.36E-13
C1orf216	3.047164	4.928264	1.8811	5.41E-13
ATP13A1	7.532823	10.81106	3.278234	5.55E-13
RBIS	3.684571	5.709577	2.025006	5.55E-13
VEZF1	4.656973	7.637503	2.980531	5.56E-13
S100A16	60.93233	114.1234	53.19111	5.60E-13
NUB1	9.982582	12.8841	2.90152	5.60E-13
EIF2B3	5.852377	7.842864	1.990486	5.69E-13
MCRIP1	17.70116	31.02446	13.3233	5.70E-13
SEC61A1	73.96102	99.79191	25.83089	5.72E-13
ACSL1	105.0048	52.48711	-52.5177	5.73E-13
NIFK	5.949434	8.50771	2.558275	5.75E-13
ZNRD2	9.101142	12.6814	3.580257	5.77E-13
SMOC1	22.98922	14.2448	-8.74442	5.80E-13
SUOX	10.91225	7.80144	-3.11081	5.84E-13
CCDC61	3.891413	5.492828	1.601415	5.88E-13
AURKB	2.214866	6.866115	4.65125	6.04E-13
WSB1	4.115341	7.565787	3.450445	6.12E-13
XDH	13.25568	5.997568	-7.25811	6.12E-13
CLSTN1	8.059832	17.41991	9.360081	6.15E-13
SSB	7.299545	10.47157	3.17202	6.16E-13
FAM111A	2.818083	4.294073	1.475991	6.26E-13
LRRC1	1.525138	3.146873	1.621735	6.30E-13
MAP1S	3.651557	5.489163	1.837606	6.39E-13
MRPL43	12.07561	15.70394	3.628338	6.43E-13
COX14	22.34089	34.12155	11.78066	6.51E-13
DEPDC7	8.389427	4.809555	-3.57987	6.57E-13
ZER1	6.538644	10.01886	3.480214	6.59E-13
STMP1	9.925156	12.80907	2.88391	6.68E-13
SUGP2	5.133022	7.981904	2.848883	6.68E-13
ZNF362	4.955857	7.569321	2.613464	6.98E-13
ATG101	3.670437	4.985016	1.314579	7.00E-13
UBE2Q2	4.01634	7.233549	3.217209	7.11E-13
YARS1	6.751483	9.898022	3.146539	7.14E-13
PRKCI	2.54363	4.128942	1.585312	7.17E-13
HSF2	2.226346	3.421478	1.195132	7.21E-13
WASH8P	3.139358	5.847993	2.708635	7.58E-13
MRPL10	12.6157	18.16593	5.550232	7.70E-13
NDUFB4	42.41261	54.43333	12.02073	7.94E-13
SLC37A1	1.410071	2.733625	1.323554	7.98E-13
ZFAND2B	10.73967	15.69692	4.957245	8.17E-13
SRSF10	3.922682	5.158448	1.235766	8.18E-13
CENATAC	1.614852	3.091887	1.477035	8.21E-13

AC000089	0.660501	1.70464	1.044138	8.23E-13
DSCC1	1.23971	2.417215	1.177505	8.30E-13
CASP6	6.181313	8.939571	2.758258	8.34E-13
SNORD14	2.631467	5.665958	3.034492	8.37E-13
DLGAP5	1.210418	2.922767	1.712348	8.39E-13
IMPDH2	15.43942	26.09958	10.66016	8.48E-13
TIA1	4.736599	7.982291	3.245692	8.48E-13
SERPINF1	304.3625	191.8314	-112.531	8.56E-13
RABIF	5.010383	6.87959	1.869207	8.61E-13
HES4	2.405744	7.162865	4.75712	8.62E-13
XRCC4	1.745318	2.777695	1.032377	8.66E-13
RBM15B	6.191777	8.116053	1.924276	8.79E-13
ENO1	133.2723	238.9643	105.692	8.81E-13
YBX3	2.682814	8.190078	5.507264	8.81E-13
TCEAL9	9.611292	18.87061	9.259315	8.85E-13
ABTB1	3.860427	5.64876	1.788333	8.99E-13
LAS1L	3.235802	4.274698	1.038896	9.04E-13
TLE3	1.923033	3.281633	1.358601	9.07E-13
SFXN3	2.502673	7.691046	5.188373	9.24E-13
SNX11	4.534649	6.06468	1.530031	9.32E-13
XPA	5.878725	7.523477	1.644752	9.39E-13
RPL14	42.46639	72.21776	29.75137	9.49E-13
DMAC2	7.093514	9.74517	2.651657	9.55E-13
MCTS1	3.534429	4.705392	1.170964	9.63E-13
TMSB4X	236.5773	542.0054	305.4281	9.69E-13
CFH	190.4461	111.9326	-78.5135	9.73E-13
OTUD5	14.91748	19.10469	4.187206	9.80E-13
SMIM20	11.8014	16.72659	4.925185	9.89E-13
ATP5MF	15.19225	25.65219	10.45994	1.02E-12
TRIM52-A	3.620111	5.888881	2.26877	1.02E-12
RNF2	2.283463	3.558029	1.274567	1.03E-12
GC	1390.657	874.6445	-516.013	1.06E-12
GRB7	3.342959	10.43984	7.09688	1.07E-12
NRGN	1.827381	3.868243	2.040862	1.07E-12
COMMD8	3.926989	5.971868	2.04488	1.08E-12
PTPA	17.07923	25.40969	8.330463	1.10E-12
GTSE1	0.939419	2.334327	1.394908	1.10E-12
FKBPL	4.861934	7.130975	2.269041	1.10E-12
PPP1CB	16.87795	23.72995	6.852007	1.11E-12
DR1	2.906087	4.671233	1.765146	1.12E-12
PGRMC1	238.2643	149.4961	-88.7682	1.14E-12
TMA7	5.69121	8.942871	3.251661	1.16E-12
FARSA	19.45609	25.41473	5.95864	1.16E-12
ARPC5	13.80078	19.28397	5.483193	1.17E-12
HMGCS2	561.0774	304.001	-257.076	1.17E-12
USB1	3.872347	5.464397	1.592049	1.18E-12
GLG1	7.153315	11.45669	4.303374	1.19E-12
GGA3	3.81092	5.394083	1.583163	1.19E-12
PTRH2	4.862605	6.945352	2.082746	1.20E-12
NFATC2IP	3.158715	4.545001	1.386286	1.20E-12
MAFG-DT	0.878287	2.554632	1.676344	1.21E-12
SPRYD3	11.47382	15.5614	4.087581	1.21E-12
SMAGP	2.123387	4.307859	2.184471	1.22E-12
F12	151.9768	84.16992	-67.8068	1.22E-12
SF1	21.84146	27.55752	5.716065	1.22E-12
PPP1R8	7.816394	10.09378	2.277381	1.26E-12
ERCC3	3.261078	4.674167	1.413089	1.27E-12
PCK1	141.3284	51.77388	-89.5546	1.28E-12

SERBP1	23.43542	30.81362	7.378196	1.29E-12
DCAF16	2.468179	4.644576	2.176397	1.31E-12
PLPP2	2.161823	10.75035	8.588529	1.31E-12
ALAS1	132.7172	66.01646	-66.7007	1.33E-12
CLK2	7.917026	12.88183	4.9648	1.33E-12
SKA2	5.848885	8.6782	2.829316	1.34E-12
MRTFA	3.228404	5.335341	2.106936	1.34E-12
C15orf39	2.74247	5.468374	2.725904	1.36E-12
RPL18AP3	7.845048	16.91588	9.07083	1.36E-12
CABIN1	3.695391	5.623335	1.927945	1.38E-12
SDHAF1	11.11517	16.82614	5.710969	1.39E-12
COMMD2	3.938284	5.51368	1.575396	1.40E-12
CDKN2A	2.035437	5.411973	3.376535	1.41E-12
PLEKHB2	4.825875	9.893367	5.067492	1.45E-12
AC026412	1.695509	2.867828	1.172319	1.45E-12
ST6GAL1	69.26331	45.53468	-23.7286	1.49E-12
MIDN	11.71551	20.46837	8.752863	1.49E-12
DDOST	57.92461	79.60939	21.68478	1.50E-12
MBD3	5.289217	7.440027	2.15081	1.50E-12
CHRAC1	7.610123	10.83227	3.222143	1.52E-12
CYP39A1	6.769759	2.213609	-4.55615	1.54E-12
PHLDA2	5.173275	16.89945	11.72618	1.55E-12
ITPR2	10.19769	5.651683	-4.54601	1.55E-12
SLC39A13	6.593424	9.889933	3.296508	1.56E-12
TUBGCP2	6.916723	9.022975	2.106253	1.57E-12
GET3	33.74525	45.45806	11.71281	1.58E-12
RPL9	56.66965	103.9387	47.26905	1.60E-12
SLC25A20	37.73573	25.28083	-12.4549	1.60E-12
CCDC115	4.208308	6.537396	2.329088	1.61E-12
SPATC1L	1.804427	4.108173	2.303747	1.61E-12
ABCC1	1.341426	4.252814	2.911388	1.61E-12
PIMREG	0.318729	1.462176	1.143448	1.64E-12
RIPK2	4.580945	8.002716	3.42177	1.67E-12
DRAM1	2.606567	6.315926	3.70936	1.67E-12
CXorf38	2.453778	3.791844	1.338066	1.69E-12
GPBP1	9.278364	11.96859	2.690222	1.69E-12
VEGFB	18.39123	35.74541	17.35418	1.70E-12
GRB2	24.86086	32.33604	7.475174	1.73E-12
ZMYM4	2.998603	4.670141	1.671537	1.74E-12
MESD	7.376324	9.366122	1.989798	1.74E-12
CCT5	14.96373	23.19374	8.230007	1.75E-12
CFDP1	9.284644	13.61612	4.331472	1.76E-12
RHPN1	1.182842	4.920619	3.737777	1.76E-12
AC093673	6.037186	9.818333	3.781147	1.77E-12
CD24	31.52881	87.40157	55.87276	1.82E-12
NUP214	3.306923	4.400703	1.093781	1.84E-12
LTA4H	6.349711	8.850687	2.500975	1.84E-12
SCP2	57.07045	33.62823	-23.4422	1.84E-12
APH1B	1.065418	2.088862	1.023444	1.86E-12
COPS3	9.510485	12.88605	3.375565	1.87E-12
EEF1B2P3	1.73014	3.203907	1.473767	1.91E-12
TAZ	3.793372	5.4496	1.656228	1.92E-12
ARHGAP1	11.08884	17.66971	6.580873	1.94E-12
CCDC22	7.660314	10.15007	2.489755	1.95E-12
ALDH2	76.01168	46.79203	-29.2197	1.97E-12
LPAR2	0.944301	5.506598	4.562296	1.97E-12
DENND2D	1.538235	3.744333	2.206098	1.98E-12
PAPSS2	19.34098	11.94852	-7.39246	1.98E-12

POC1A	3.738466	6.220616	2.482151	2.00E-12
ASXL1	3.78553	5.533871	1.748341	2.00E-12
CHMP3	2.850467	5.154917	2.30445	2.04E-12
BTBD10	3.10351	4.956789	1.853278	2.13E-12
GADD45G	25.29375	44.84225	19.5485	2.17E-12
SAMD4B	7.014479	10.20629	3.191807	2.19E-12
FKBP10	4.093995	19.41548	15.32148	2.22E-12
SEC11A	15.16745	20.78093	5.613481	2.26E-12
AL031673.	1.492613	2.612166	1.119553	2.26E-12
BRD8	4.206602	6.094189	1.887587	2.35E-12
KCMF1	5.767066	7.315432	1.548366	2.36E-12
RNF123	4.858763	3.471544	-1.38722	2.38E-12
PRDX5	230.1557	313.1374	82.98171	2.41E-12
CNPPD1	19.62328	25.51596	5.892682	2.46E-12
XAB2	11.12828	14.82581	3.697534	2.46E-12
ABCC9	2.746487	1.262947	-1.48354	2.51E-12
TTC9C	4.247203	5.775442	1.528239	2.53E-12
UBAP2L	13.65627	20.08845	6.432175	2.55E-12
AC034236	12.128	24.7379	12.6099	2.63E-12
CDKN3	3.505549	7.442975	3.937426	2.64E-12
IKBIP	2.885232	4.580546	1.695314	2.65E-12
G6PD	6.028862	23.60191	17.57305	2.66E-12
SLC25A42	19.70457	11.57649	-8.12807	2.75E-12
PTGFRN	5.577454	11.40293	5.82548	2.76E-12
EIF6	45.72475	62.09869	16.37393	2.79E-12
LRRC41	5.605775	8.001855	2.39608	2.81E-12
POLR1D	4.660988	6.228845	1.567858	2.88E-12
ZNF875	2.060959	3.370087	1.309129	2.90E-12
PIK3R1	10.53247	6.425432	-4.10704	2.92E-12
SLC41A3	5.046647	6.85061	1.803963	2.93E-12
RRM2	2.537498	4.918086	2.380589	2.93E-12
CTDP1	3.702295	4.847896	1.145601	2.93E-12
GPR107	5.931668	8.895511	2.963843	2.97E-12
SLC12A9	3.717598	5.409831	1.692233	3.00E-12
POU5F1	0.42487	1.499674	1.074803	3.00E-12
CAPN15	3.395168	4.795087	1.399918	3.00E-12
TMEM223	6.986497	9.278525	2.292028	3.01E-12
U2SURP	4.238978	6.095731	1.856753	3.06E-12
UBL4A	17.20079	24.86803	7.667237	3.07E-12
AMDHD1	32.35283	14.52168	-17.8312	3.08E-12
KCNJ8	17.17123	8.326263	-8.84496	3.08E-12
UBA2	10.74083	16.49403	5.753201	3.12E-12
MLLT3	0.318827	1.393183	1.074356	3.16E-12
GMEB2	3.740499	5.022371	1.281872	3.18E-12
PIGC	5.857178	8.479007	2.621829	3.20E-12
TMED9	67.37746	89.19606	21.8186	3.21E-12
ALG3	21.10237	27.75218	6.64981	3.24E-12
ZNF787	12.06082	17.25524	5.194422	3.24E-12
BBC3	3.011027	5.310817	2.299791	3.28E-12
RAPGEF4	3.231961	1.868945	-1.36302	3.29E-12
ZNF622	18.19224	24.25826	6.06602	3.30E-12
DNMT3A	1.883502	3.182273	1.298771	3.33E-12
UBE2E3	10.51568	14.96299	4.447311	3.35E-12
NPEPPS	2.377016	4.020175	1.643159	3.37E-12
BICD2	2.70285	3.949758	1.246908	3.39E-12
SMIM7	5.395545	7.121831	1.726286	3.40E-12
ACTL6A	4.326216	6.720067	2.393852	3.41E-12
AHSA2P	2.001833	3.396498	1.394665	3.44E-12

LIMK1	2.003635	4.929526	2.925891	3.45E-12
PRR34-AS	0.839033	2.00137	1.162337	3.47E-12
ABCA8	2.981168	1.201703	-1.77947	3.50E-12
TCTN2	0.447783	1.762878	1.315096	3.52E-12
USP33	4.271528	6.278284	2.006756	3.53E-12
TTC13	3.486057	5.299332	1.813274	3.53E-12
AP2M1	41.24901	64.11502	22.86601	3.57E-12
CSNK1G2	10.70226	15.38638	4.684115	3.59E-12
PAPSS1	5.071743	9.381937	4.310195	3.62E-12
SPRING1	2.124389	5.062629	2.938239	3.66E-12
TYRO3	0.589957	2.329519	1.739562	3.78E-12
PRXL2B	4.262462	7.302035	3.039573	3.79E-12
CDK4	8.769832	14.1941	5.42427	3.82E-12
SRSF1	5.560702	7.252649	1.691947	3.83E-12
NSD3	1.466261	2.477257	1.010996	3.83E-12
NR1I2	6.721354	3.28435	-3.437	3.83E-12
TFCP2	4.484892	6.598361	2.113469	3.84E-12
SOX12	4.815858	9.239915	4.424057	3.86E-12
RPS12	738.5985	1328.337	589.7386	3.88E-12
NUMBL	0.968076	2.055898	1.087822	3.92E-12
RELB	7.269774	12.68751	5.417738	3.99E-12
RNF145	4.33744	8.357497	4.020056	4.12E-12
ABHD6	10.69951	6.789774	-3.90974	4.24E-12
APOH	3486.31	2164.921	-1321.39	4.32E-12
AL591895.	7.252788	21.09396	13.84117	4.32E-12
CTBP2	0.547099	2.027489	1.48039	4.36E-12
AK6	5.851795	7.562728	1.710933	4.43E-12
SLC25A25	21.15681	9.698129	-11.4587	4.43E-12
GPATCH4	7.50748	10.8633	3.355823	4.47E-12
ROMO1	83.56549	143.762	60.19652	4.52E-12
MKI67	1.846799	4.279006	2.432208	4.55E-12
LENG1	6.160828	8.496668	2.33584	4.61E-12
OGFOD1	3.328448	4.435467	1.107019	4.63E-12
BORCS6	2.946312	4.402376	1.456064	4.66E-12
CUEDC2	27.99582	36.93089	8.935077	4.71E-12
TUFT1	3.256709	6.152468	2.89576	4.71E-12
CCT4	27.26104	36.97739	9.716345	4.77E-12
TPM3P9	1.154434	2.324422	1.169988	4.82E-12
FRMD8	6.73903	10.25627	3.517236	4.84E-12
NELFA	3.929639	5.525228	1.595589	4.88E-12
PLEKHJ1	8.349169	13.51671	5.16754	4.89E-12
KAT2B	7.812706	4.834598	-2.97811	4.91E-12
RAD9A	2.335621	3.581773	1.246152	4.91E-12
OSBPL3	0.931049	2.186097	1.255048	4.93E-12
VDAC2	13.81066	19.47367	5.663018	4.94E-12
BCKDHB	8.318633	5.258197	-3.06044	4.96E-12
NSD2	1.559303	2.627388	1.068084	5.03E-12
TUG1	4.819151	7.489462	2.670311	5.06E-12
SS18	7.510966	10.51493	3.00396	5.07E-12
NCAPG	1.218524	2.715957	1.497433	5.13E-12
SLBP	16.51505	23.10092	6.585867	5.39E-12
DNAJB6	3.140567	4.181271	1.040704	5.42E-12
CHST14	6.149768	9.213011	3.063242	5.46E-12
POLR2D	4.654639	5.997224	1.342585	5.51E-12
AGMO	19.63437	11.34306	-8.29131	5.55E-12
PCIF1	9.236596	12.24216	3.005568	5.57E-12
APEX2	7.781367	10.63667	2.855299	5.61E-12
HSPB1	255.2869	471.6526	216.3656	5.64E-12

EMD	24.58654	33.70915	9.122602	5.74E-12
RHOA	58.99897	74.45173	15.45276	5.78E-12
TPRN	4.779833	10.20601	5.426181	5.80E-12
LRRC3	5.993775	3.129151	-2.86462	5.82E-12
TMEM184	4.921871	8.323317	3.401446	5.83E-12
CWF19L1	3.351678	4.472838	1.12116	5.83E-12
TNIP2	10.18277	13.99774	3.814977	5.83E-12
LZTS2	4.091985	7.555129	3.463144	5.88E-12
ANKRD52	3.41597	5.470103	2.054133	5.89E-12
ITPKC	8.479594	13.35015	4.870555	5.93E-12
PPP1R12C	6.836339	9.210902	2.374564	5.94E-12
TERF2IP	7.982389	10.75577	2.773377	5.94E-12
RNF41	3.310538	4.480372	1.169834	5.96E-12
ERBB3	22.22988	35.89675	13.66687	5.96E-12
ANKRD54	4.439434	6.141553	1.702119	6.05E-12
GTPBP2	6.635185	10.62756	3.992379	6.06E-12
RHBDF2	2.874746	6.377898	3.503151	6.09E-12
DYNC1LI1	3.457939	4.650684	1.192744	6.13E-12
HJV	108.2495	56.49702	-51.7525	6.15E-12
SETD1A	4.327552	5.876214	1.548662	6.19E-12
SEC61B	90.35304	119.3397	28.98662	6.20E-12
NUP155	2.415424	3.539434	1.12401	6.21E-12
LONP2	14.24866	10.09437	-4.15428	6.23E-12
PRKX	0.950471	2.508155	1.557684	6.28E-12
F7	50.90573	31.07328	-19.8324	6.31E-12
L3MBTL2	3.864828	4.941685	1.076857	6.34E-12
AAMP	31.11132	39.18593	8.074607	6.38E-12
RPS18	481.2936	767.9621	286.6685	6.42E-12
SLFN13	0.450632	3.199062	2.74843	6.44E-12
ARHGAP2	0.998643	2.081064	1.082421	6.47E-12
XPO1	3.451678	4.576625	1.124946	6.63E-12
FBLIM1	5.008081	13.5914	8.583321	6.78E-12
JOSD2	12.96763	21.64047	8.672841	6.93E-12
LAGE3	19.76503	33.02771	13.26268	6.96E-12
TOP2A	5.23487	11.78236	6.547491	7.05E-12
AC097448	3.399359	4.744712	1.345354	7.10E-12
TUBB4B	69.31233	106.3387	37.02638	7.27E-12
SUPT16H	8.506176	12.00014	3.493965	7.30E-12
CORO1C	10.28104	15.13127	4.85023	7.33E-12
HMGB1	15.321	19.85107	4.530067	7.36E-12
TUBG1	11.54517	17.81016	6.264991	7.40E-12
MMS19	5.99407	8.267453	2.273383	7.48E-12
ENPEP	6.559896	3.568467	-2.99143	7.48E-12
PLEKHO1	2.530067	6.319242	3.789175	7.54E-12
UNK	3.391639	4.779073	1.387433	7.54E-12
APOBEC3(	2.438253	8.321225	5.882972	7.56E-12
MRPL48	4.810085	6.371445	1.56136	7.63E-12
NDUFS6	86.15333	138.5536	52.40025	7.65E-12
SVIP	2.827263	4.14836	1.321097	7.66E-12
RPL3P4	21.75778	46.50313	24.74535	7.72E-12
APOBR	1.247015	2.297661	1.050646	7.73E-12
ARHGEF2	2.698946	5.508809	2.809864	7.74E-12
DENND4B	4.444763	6.675515	2.230752	7.78E-12
WDR45B	18.03389	25.51393	7.480037	7.82E-12
TGIF2	4.682897	8.116959	3.434062	7.85E-12
ELFN1	7.235152	2.277386	-4.95777	7.85E-12
METTL18	3.615873	5.248499	1.632625	7.99E-12
RPS6KA4	5.894856	8.90728	3.012424	8.00E-12

ZCCHC3	7.21004	10.79487	3.584832	8.03E-12
AC083855	0.523999	1.683165	1.159167	8.07E-12
MYDGF	75.59377	102.0031	26.40936	8.14E-12
ADAM15	11.33474	18.00307	6.668335	8.18E-12
NACC1	9.216118	13.05197	3.83585	8.25E-12
MAPK13	2.117822	6.122428	4.004606	8.28E-12
PSMB8	22.27095	35.82833	13.55738	8.32E-12
ISG20L2	6.246571	8.966523	2.719952	8.35E-12
CBX2	0.567563	1.778554	1.210991	8.39E-12
SLC18B1	3.287957	5.725884	2.437928	8.46E-12
TNPO2	5.652905	8.045434	2.392529	8.48E-12
SMOX	3.218677	7.475	4.256323	8.51E-12
PNO1	5.341437	7.113646	1.772209	8.63E-12
SLC66A1	7.233407	10.63553	3.402125	8.68E-12
ENKD1	3.20143	6.453563	3.252133	8.71E-12
SNX17	27.26972	34.10459	6.834873	8.73E-12
NOP53	39.45609	62.96786	23.51177	8.77E-12
DVL3	9.648587	13.68958	4.040992	8.82E-12
RGS19	3.471511	6.590846	3.119335	8.84E-12
ABHD17C	2.227077	4.726722	2.499645	8.90E-12
ICMT	8.502705	11.84963	3.346927	8.98E-12
MST1	37.9683	24.13688	-13.8314	9.29E-12
AC135050	7.350408	10.18347	2.833063	9.31E-12
DSTN	42.0417	66.78126	24.73956	9.41E-12
SPATA2	3.195533	4.243277	1.047743	9.42E-12
NDUFA11	1.966176	3.500998	1.534822	9.43E-12
AC099336	9.112158	21.82467	12.71251	9.44E-12
PSMC4	37.66167	50.314	12.65234	9.53E-12
SUMO1	43.7326	53.07529	9.342691	9.79E-12
KAT8	4.171319	5.476385	1.305066	9.88E-12
ENTPD2	1.17593	3.922331	2.746401	9.91E-12
UQCC2	7.463535	11.2696	3.806064	1.00E-11
CLPX	12.82172	10.07401	-2.74771	1.01E-11
CBX8	2.44855	3.743939	1.295389	1.03E-11
CIB2	0.708435	2.23842	1.529985	1.03E-11
SRSF11	7.453316	12.69589	5.242576	1.04E-11
CSTF2	3.180556	5.038871	1.858316	1.04E-11
ANXA11	15.07902	24.54148	9.462468	1.06E-11
SH2B1	5.01586	6.931896	1.916036	1.07E-11
ITPR3	0.727222	3.950226	3.223003	1.07E-11
HNRNPR	7.868418	10.5781	2.70968	1.08E-11
ZBTB12	1.194598	2.404817	1.21022	1.08E-11
SLC7A7	1.509934	3.688696	2.178761	1.09E-11
RIPOR1	3.808639	5.941558	2.132918	1.09E-11
CXXC5	25.33154	35.28211	9.950574	1.09E-11
MMD	4.566053	10.00764	5.44159	1.09E-11
XYLT2	5.37879	7.454566	2.075776	1.09E-11
TBL2	5.303844	7.007489	1.703645	1.10E-11
SRSF4	6.24304	8.090093	1.847053	1.11E-11
PYCR1	3.087594	9.135009	6.047416	1.12E-11
TMEM222	7.980131	10.39233	2.412198	1.12E-11
RBM12	4.619237	6.311574	1.692336	1.13E-11
INTS3	6.783494	10.19811	3.414612	1.13E-11
MRPL28	18.66262	28.30315	9.640525	1.14E-11
RPL15	63.56031	85.52988	21.96957	1.14E-11
BRI3BP	2.82887	4.63396	1.805089	1.14E-11
TAT	181.3973	60.53337	-120.864	1.15E-11
COASY	20.70869	27.30996	6.601266	1.15E-11

CDR2L	1.127641	5.645432	4.517791	1.16E-11
ANP32E	12.17276	18.01514	5.842386	1.18E-11
TAF12	6.098081	8.533662	2.435581	1.19E-11
SNRNP25	10.73027	15.4605	4.730225	1.20E-11
NOP58	9.678692	13.52508	3.846391	1.20E-11
PRELID3B	12.83898	18.3714	5.532422	1.22E-11
HPF1	8.38928	11.44	3.050717	1.22E-11
THYN1	10.59983	14.00824	3.408404	1.22E-11
STX5	15.46151	19.11051	3.649002	1.22E-11
PPM1M	2.718976	4.431462	1.712486	1.23E-11
TXNDC17	10.28562	15.50337	5.217748	1.24E-11
CLDN4	4.661724	23.73627	19.07454	1.25E-11
OAF	85.29118	57.0091	-28.2821	1.25E-11
RPL29	180.8823	282.4924	101.6101	1.27E-11
PHF20	2.688312	3.688465	1.000153	1.28E-11
EZR	21.01787	42.08204	21.06417	1.29E-11
RNF44	5.24157	8.787786	3.546216	1.30E-11
CHPT1	18.10601	12.54912	-5.5569	1.31E-11
USP5	20.37048	26.24643	5.875954	1.32E-11
PSMB6	54.34985	75.28756	20.93772	1.32E-11
CYP27A1	215.2137	132.4783	-82.7355	1.34E-11
TARS1	16.25887	22.89418	6.635301	1.36E-11
CES2	95.09989	50.57604	-44.5238	1.36E-11
SEC14L2	30.23411	12.25452	-17.9796	1.37E-11
PDE7A	0.772071	1.870318	1.098246	1.37E-11
PPCS	12.0341	15.26648	3.232378	1.40E-11
TRMT61A	6.5488	8.911214	2.362415	1.41E-11
MOGAT2	9.049495	3.268171	-5.78132	1.42E-11
SP110	1.819621	2.827745	1.008124	1.43E-11
AC083799	4.419471	6.331409	1.911938	1.44E-11
MTTP	34.43016	20.17401	-14.2561	1.45E-11
MYLK	3.789303	2.286405	-1.5029	1.45E-11
ACSM2B	38.36269	21.5099	-16.8528	1.45E-11
CKAP4	27.68013	47.20605	19.52592	1.46E-11
MRPS2	10.4906	14.96117	4.470574	1.46E-11
ZFC3H1	1.674977	2.772833	1.097855	1.47E-11
ATF7	1.844343	3.040623	1.19628	1.48E-11
RPL4P4	1.44031	2.536006	1.095696	1.48E-11
RPS15P4	6.882583	13.47937	6.59679	1.49E-11
MED29	15.11843	19.21983	4.101401	1.50E-11
PCNX3	6.471965	8.673027	2.201062	1.50E-11
PSMB9	9.88199	20.99503	11.11304	1.51E-11
NDUFA8	47.78697	65.50703	17.72007	1.55E-11
ACTR3	7.3719	10.82008	3.448178	1.61E-11
IQGAP1	2.952028	7.617542	4.665514	1.62E-11
COX7C	120.8195	163.6165	42.79694	1.62E-11
SNHG6	19.98304	41.39411	21.41107	1.63E-11
RAB11FIP5	2.349281	4.433417	2.084136	1.65E-11
RGS1	3.126285	9.909526	6.78324	1.67E-11
ADA	1.907586	3.294632	1.387046	1.68E-11
PNPLA6	6.47373	8.586828	2.113098	1.71E-11
FUCA2	19.19124	27.44816	8.25692	1.71E-11
PPP1R18	7.336559	14.55189	7.215327	1.72E-11
CTSC	2.528929	6.89805	4.369121	1.72E-11
TMEM65	1.740553	3.273735	1.533182	1.72E-11
EEF1B2P6	1.033972	2.55243	1.518458	1.73E-11
THAP9-AS	2.141552	3.65377	1.512218	1.73E-11
SAP30	3.121825	4.946748	1.824923	1.74E-11

ZNF266	2.440799	4.285599	1.8448	1.76E-11
NUDT21	5.990685	8.470707	2.480022	1.76E-11
ZNF888	0.526681	2.296395	1.769714	1.77E-11
SELENOM	8.175158	21.23276	13.0576	1.78E-11
POR	116.9792	73.68455	-43.2947	1.80E-11
SNHG7	3.34274	6.891399	3.548659	1.82E-11
RNU1-70I	4.796893	2.546397	-2.2505	1.83E-11
MRPS12	21.48641	33.15011	11.66369	1.84E-11
SLC35A4	20.32705	26.14364	5.816592	1.85E-11
SNAPC2	5.544367	7.617614	2.073248	1.85E-11
TMX3	2.285861	3.695741	1.40988	1.88E-11
PDE9A	1.471272	3.598492	2.12722	1.88E-11
NUP205	3.26761	4.95377	1.68616	1.88E-11
VPS9D1	3.999953	5.832681	1.832728	1.88E-11
ELAVL1	7.742333	9.790591	2.048258	1.89E-11
NACA3P	1.756995	3.050748	1.293753	1.91E-11
PPIAP22	41.07091	54.84581	13.7749	1.93E-11
MCUB	0.875874	2.294885	1.419011	1.94E-11
INO80E	4.391204	5.953683	1.562479	1.94E-11
SPHK1	2.198762	10.82212	8.623361	1.95E-11
WDR90	1.14319	2.195651	1.052461	1.96E-11
GMIP	2.119465	4.638517	2.519052	1.99E-11
SLC25A13	36.05463	24.53296	-11.5217	2.00E-11
SSBP4	6.678878	10.51179	3.832908	2.00E-11
AIF1	9.193827	19.88982	10.696	2.01E-11
AC083862	1.586818	3.272901	1.686084	2.06E-11
SYS1	5.685879	7.358616	1.672737	2.08E-11
NEMP1	2.091845	3.464805	1.37296	2.08E-11
SLC9A1	1.293385	3.200137	1.906752	2.10E-11
HIP1R	7.553931	13.58477	6.030834	2.11E-11
SS18L2	4.412582	5.966905	1.554323	2.13E-11
FOXM1	2.283431	4.970458	2.687027	2.14E-11
LAD1	12.13392	36.86031	24.72639	2.15E-11
PKLR	62.41599	26.91756	-35.4984	2.17E-11
MSMO1	104.9764	65.29178	-39.6846	2.19E-11
LAT2	1.120084	3.759571	2.639487	2.20E-11
MRPS7	16.00278	20.66086	4.658077	2.22E-11
INO80C	1.732091	3.034813	1.302722	2.23E-11
NBEAL2	1.456867	3.342205	1.885338	2.29E-11
RIC8A	10.29709	13.52564	3.228543	2.30E-11
SLC25A15	29.98143	13.93408	-16.0473	2.30E-11
COP1	11.4091	15.17386	3.764767	2.30E-11
TMEM80	2.996379	4.604903	1.608524	2.34E-11
PC	37.56451	22.42668	-15.1378	2.35E-11
POF1B	0.582767	3.585715	3.002948	2.36E-11
VPS35	5.769302	8.518673	2.749371	2.37E-11
PLCD3	0.718829	3.294522	2.575693	2.37E-11
ITM2C	17.63044	34.44883	16.81839	2.38E-11
RPL41P5	36.4339	59.28148	22.84758	2.45E-11
PLEKHG2	1.472115	3.07493	1.602814	2.46E-11
PTTG1IP	48.55305	69.11965	20.5666	2.47E-11
AC007485	2.163366	3.392452	1.229086	2.47E-11
PUDP	3.233885	5.48531	2.251426	2.50E-11
MCCC2	23.85283	18.03249	-5.82033	2.51E-11
RNF181	56.77478	79.67475	22.89998	2.52E-11
MICA	3.345986	5.246996	1.90101	2.53E-11
INPP5K	4.055165	5.667361	1.612196	2.53E-11
ENOPH1	10.71799	14.49622	3.778229	2.53E-11

FBL	36.16383	81.66347	45.49964	2.53E-11
COIL	5.413743	7.246271	1.832529	2.55E-11
AP2B1	12.76461	19.59157	6.826967	2.56E-11
RAB28	3.178316	4.496821	1.318505	2.57E-11
ZCCHC7	3.043948	4.131251	1.087302	2.57E-11
ANO9	0.392125	3.102824	2.710699	2.58E-11
ADH1A	178.4628	93.04542	-85.4174	2.58E-11
AAR2	12.50953	15.51591	3.006381	2.59E-11
APBB1	2.220483	3.771332	1.550848	2.60E-11
AP002360	2.166726	4.140272	1.973546	2.60E-11
RPL7AP50	0.740019	1.789079	1.04906	2.65E-11
NDOR1	2.793194	4.333154	1.539961	2.67E-11
MYO19	1.801348	2.80781	1.006462	2.68E-11
EPB41L4A	2.743465	4.580447	1.836983	2.68E-11
GOLGA8B	2.181655	3.713634	1.531979	2.71E-11
POMP	50.74104	66.61658	15.87554	2.73E-11
SLC25A47	83.40771	24.27044	-59.1373	2.76E-11
PAQR4	0.989006	2.440465	1.451459	2.79E-11
PBLD	21.09337	12.28851	-8.80486	2.83E-11
AL080243.	1.035583	2.18307	1.147487	2.85E-11
CYP4V2	10.02573	6.35491	-3.67082	2.85E-11
JADE2	1.912272	3.338	1.425728	2.85E-11
ARMCX1	1.148106	3.309049	2.160943	2.85E-11
CDC42SE2	6.329591	9.787096	3.457505	2.86E-11
FAM219A	3.251404	4.680262	1.428857	2.87E-11
SCX	0.625003	1.811062	1.186059	2.88E-11
BPHL	8.021889	5.274999	-2.74689	2.93E-11
RCN1	7.92583	13.87026	5.944432	2.96E-11
PIAS4	5.84957	7.769869	1.9203	2.98E-11
CTNND2	0.812849	4.226086	3.413237	3.06E-11
RERE	3.335475	5.119861	1.784386	3.07E-11
ADNP	7.02229	10.01306	2.990771	3.11E-11
FBRSL1	4.517755	6.236033	1.718279	3.12E-11
MAVS	5.971259	8.226664	2.255405	3.19E-11
RPS29	18.41081	31.17316	12.76235	3.24E-11
IFT140	0.865498	2.011019	1.145521	3.28E-11
SUV39H1	3.949848	5.644677	1.694829	3.30E-11
CCDC130	5.427384	8.561687	3.134303	3.30E-11
ZNF524	10.60125	15.76627	5.165016	3.31E-11
PLPP5	4.894719	8.523149	3.62843	3.32E-11
CDC14B	3.930363	2.310643	-1.61972	3.42E-11
E2F1	5.562532	12.55548	6.992949	3.51E-11
DDAH2	24.13166	40.9229	16.79124	3.52E-11
PDE6D	4.292498	5.620292	1.327795	3.56E-11
RPL3	315.1354	454.5145	139.3791	3.57E-11
CLIP2	2.311693	6.116284	3.804591	3.58E-11
SMIM29	8.747483	13.58516	4.83768	3.61E-11
HEXD	5.178248	7.596697	2.418449	3.63E-11
FGA	3028.844	1753.122	-1275.72	3.68E-11
PIP4P2	1.54398	3.112746	1.568766	3.86E-11
ZC3H4	3.996333	5.50842	1.512087	3.86E-11
HIBCH	7.660664	5.589765	-2.0709	3.92E-11
CAPG	10.74015	24.48383	13.74368	3.96E-11
AL662795.	0.974912	1.986003	1.011091	3.97E-11
C5	90.22486	56.40965	-33.8152	4.02E-11
TRPT1	8.181748	11.08351	2.901762	4.04E-11
PEA15	37.64162	56.08461	18.44299	4.07E-11
ZNF496	1.421651	2.708146	1.286495	4.11E-11

TIAL1	6.422113	8.052363	1.63025	4.16E-11
DNAJB9	37.82931	25.49672	-12.3326	4.17E-11
AL499602.	0.94735	3.061062	2.113712	4.18E-11
NRBP1	17.30197	23.01923	5.717255	4.21E-11
SARS1	29.22963	39.63637	10.40673	4.24E-11
TOR4A	1.459084	4.625739	3.166655	4.25E-11
RPL7	163.3143	270.3931	107.0788	4.29E-11
SIK2	12.14739	8.256685	-3.8907	4.30E-11
CIAO1	15.97017	19.48878	3.518608	4.35E-11
COBLL1	5.591161	3.287406	-2.30375	4.38E-11
TPM4	9.165799	20.98574	11.81994	4.40E-11
SMG5	24.63696	34.45744	9.820479	4.40E-11
EIF4EBP1	31.2771	60.17519	28.89809	4.41E-11
TNFSF12	4.041399	6.857559	2.816161	4.44E-11
CCAR1	6.117349	8.496313	2.378964	4.46E-11
CDK5	7.181702	9.873412	2.69171	4.50E-11
VPS26A	7.426236	9.863529	2.437293	4.52E-11
RASSF3	5.054125	8.420798	3.366673	4.53E-11
GRHPR	49.85172	32.16645	-17.6853	4.54E-11
ETV4	3.966313	11.42146	7.455149	4.54E-11
BYSL	9.814905	13.51969	3.70479	4.56E-11
MAPKAPK	26.30414	36.57913	10.27499	4.70E-11
BAD	5.325961	7.431985	2.106024	4.77E-11
SULT2A1	331.0865	163.6157	-167.471	4.78E-11
PABPC1L	3.865433	7.641912	3.776478	4.85E-11
TXLNA	13.40992	17.8787	4.468772	4.87E-11
NVL	2.199687	3.288126	1.088439	4.89E-11
ELOC	9.047933	12.77168	3.723743	4.89E-11
KNSTRN	2.800172	4.169732	1.36956	4.90E-11
PSMB5	64.56701	80.18505	15.61803	4.90E-11
USP24	3.613287	5.015155	1.401868	4.91E-11
AC008038	3.85691	6.805447	2.948537	4.92E-11
CYHR1	5.186029	7.34192	2.155892	4.93E-11
ADH4	318.8947	129.0686	-189.826	4.93E-11
LDOC1	0.62014	3.721666	3.101526	4.95E-11
ALDH7A1	10.5907	6.99639	-3.59431	5.02E-11
TSG101	15.14232	19.05104	3.908714	5.02E-11
NCKAP5L	1.732266	2.818746	1.086481	5.06E-11
AKIP1	6.017389	8.275545	2.258156	5.07E-11
KDM5C	7.135471	10.2608	3.125325	5.07E-11
TNFAIP8	0.777597	2.057405	1.279809	5.21E-11
PSMG1	8.371508	11.38869	3.017185	5.22E-11
MICAL1	1.59197	4.482129	2.890159	5.23E-11
NDUFB2	10.13353	14.72301	4.589488	5.24E-11
BTN2A1	4.007329	6.013562	2.006233	5.24E-11
RGS3	2.913157	4.810484	1.897328	5.25E-11
DSG2	4.561157	16.65957	12.09841	5.25E-11
OGFR	14.72825	19.29666	4.568407	5.30E-11
TUBA1B	9.934118	16.60158	6.667463	5.40E-11
TSPAN17	7.028303	9.924481	2.896178	5.40E-11
RPL24P4	3.605393	5.603095	1.997702	5.42E-11
ZNF768	15.74603	22.15579	6.409758	5.47E-11
TAF1C	3.156736	4.925956	1.76922	5.48E-11
LSG1	5.845517	7.710915	1.865399	5.60E-11
THBS3	2.060793	3.529281	1.468489	5.63E-11
KLHDC2	6.813147	4.95686	-1.85629	5.63E-11
RTN3	22.38815	29.56583	7.17768	5.64E-11
THAP8	3.964304	5.689711	1.725406	5.68E-11

EHD1	5.694241	8.477037	2.782796	5.76E-11
UNC119	3.486214	5.384366	1.898152	5.81E-11
ACADM	25.10387	16.19554	-8.90833	5.82E-11
WDR41	2.471591	3.516072	1.044481	5.83E-11
S100A13	2.742607	7.152952	4.410345	5.85E-11
PARP2	3.488679	4.663447	1.174768	5.94E-11
SLC44A2	6.969012	19.18567	12.21666	5.97E-11
PLEKHO2	4.625007	7.903424	3.278417	5.98E-11
FAM122B	3.628812	5.531449	1.902637	5.99E-11
PDCD2L	1.805928	3.07641	1.270483	6.17E-11
PCBP4	6.134963	9.314305	3.179342	6.21E-11
NCS1	1.276629	3.548987	2.272359	6.29E-11
BTBD2	8.443114	12.54226	4.099141	6.38E-11
UBAC2	14.02529	19.12942	5.104128	6.40E-11
QTRT1	10.59056	14.6615	4.070937	6.41E-11
CARS2	3.369973	4.683744	1.31377	6.42E-11
SERF2	21.87135	37.16008	15.28873	6.44E-11
RNU6-516	1.353637	2.445026	1.091388	6.46E-11
ZMAT5	3.127977	4.157638	1.029661	6.52E-11
LAMB1	3.549651	7.968268	4.418617	6.53E-11
RPS25	197.9193	287.1178	89.19847	6.54E-11
ADRA1B	2.201996	1.046052	-1.15594	6.55E-11
BRD7	3.80382	5.037464	1.233643	6.66E-11
CPS1	172.3506	79.87091	-92.4797	6.68E-11
GPHN	5.513231	3.172066	-2.34117	6.71E-11
KLKB1	35.33051	19.57694	-15.7536	6.80E-11
ZNF296	0.464416	1.583096	1.11868	6.84E-11
DMPK	1.22412	2.862271	1.638151	6.87E-11
GLA	3.249976	5.156958	1.906982	6.87E-11
GABARAP	16.47281	20.83546	4.362648	6.94E-11
ZNF428	6.066479	9.961224	3.894744	6.95E-11
MRPL27	15.77692	24.04845	8.271531	6.97E-11
MED24	8.605374	11.35226	2.74689	6.98E-11
RPL26L1	15.22985	19.59496	4.365111	7.03E-11
RNF115	3.543087	4.834611	1.291525	7.06E-11
PCED1A	11.78157	16.66742	4.885851	7.21E-11
UBE2R2	17.55694	21.9117	4.354756	7.24E-11
COX7A2L	14.80269	19.03031	4.227621	7.27E-11
SH2D3A	0.549337	3.498	2.948662	7.29E-11
FKBP9	7.821207	12.78715	4.965939	7.31E-11
RBMS2	0.955124	2.532116	1.576992	7.35E-11
STK39	2.654122	6.349142	3.695021	7.38E-11
LARS1	2.846513	3.884854	1.03834	7.45E-11
C12orf75	2.830134	16.79548	13.96534	7.46E-11
ABTB2	5.006293	3.433962	-1.57233	7.53E-11
PITX1	0.966807	3.217695	2.250888	7.53E-11
EIF1	135.0084	175.4752	40.46678	7.65E-11
ZNHIT2	6.374769	9.200616	2.825846	7.73E-11
SOX9	7.435411	20.09033	12.65492	7.81E-11
SDC3	5.6699	10.64153	4.971633	7.82E-11
PISD	3.714736	5.688848	1.974111	7.91E-11
STARD3	8.082341	10.88279	2.800449	7.93E-11
MTX2	13.13384	17.15178	4.017933	8.14E-11
PPP5C	5.383992	7.724252	2.34026	8.21E-11
ZNF12	2.756724	3.837143	1.08042	8.24E-11
PCGF2	1.871596	3.237864	1.366268	8.24E-11
RCN3	6.613409	11.11153	4.498124	8.30E-11
NT5C3B	12.04369	17.20006	5.156371	8.30E-11

NOC2L	9.652031	13.49815	3.846122	8.39E-11
BTBD1	7.519605	10.4662	2.946593	8.44E-11
LLGL2	9.665686	16.16187	6.496187	8.71E-11
IL15RA	4.345536	8.275469	3.929933	8.83E-11
GPLD1	11.51289	3.684937	-7.82795	8.84E-11
AOC4P	3.845533	1.748326	-2.09721	8.94E-11
AP3M2	1.117245	2.276235	1.15899	8.97E-11
UROC1	17.06385	5.634782	-11.4291	8.97E-11
COX4I1	59.32058	90.26654	30.94597	9.03E-11
GTF2H1	3.510674	4.615035	1.104361	9.03E-11
UHRF1	0.964322	2.273509	1.309187	9.12E-11
FAR1	0.729892	2.03915	1.309259	9.12E-11
RRM1	7.858836	11.89182	4.032986	9.15E-11
HCST	0.765394	2.289327	1.523933	9.23E-11
MCU	3.265924	4.768448	1.502524	9.24E-11
LHFPL2	1.347555	2.712635	1.36508	9.24E-11
KRT8	97.26051	181.4298	84.16934	9.29E-11
GLE1	6.852708	8.979271	2.126563	9.34E-11
TNFRSF12	27.69355	54.45033	26.75678	9.38E-11
INTS8	2.237684	3.347601	1.109918	9.41E-11
UNC93B1	6.928607	11.10695	4.178347	9.50E-11
ZYX	31.01811	45.44217	14.42406	9.51E-11
DDIT3	11.91311	21.49883	9.58572	9.58E-11
NF2	3.630333	5.164343	1.534009	9.62E-11
ZNF444	7.175216	10.29562	3.120407	9.67E-11
AIMP1	4.653722	6.539991	1.886268	9.73E-11
CSRNP2	2.43928	3.860607	1.421328	9.79E-11
SLC44A3	4.688863	13.79541	9.106546	9.86E-11
AURKAIP1	52.79456	75.62836	22.8338	9.89E-11
AC008610	0.655657	1.894625	1.238968	9.97E-11
IVNS1ABP	7.928379	15.61456	7.686182	1.01E-10
RDH16	60.64706	24.38415	-36.2629	1.01E-10
MBL2	29.91525	13.01761	-16.8976	1.02E-10
LGALS3BP	103.4927	197.0585	93.56581	1.02E-10
CAMSAP2	2.605622	4.531572	1.92595	1.03E-10
FANCL	2.921306	4.040684	1.119377	1.03E-10
YIPF1	17.69303	22.01237	4.319344	1.04E-10
R3HCC1L	2.618976	3.819052	1.200076	1.04E-10
AC138207	1.192147	2.377026	1.184879	1.05E-10
COX6A1	27.53281	41.75756	14.22475	1.05E-10
LSM14A	16.29663	22.678	6.381372	1.09E-10
NAA38	13.8125	22.07894	8.266435	1.09E-10
SH2D2A	0.77115	1.996154	1.225004	1.10E-10
RBM5	2.915287	3.961297	1.04601	1.11E-10
JRKL	1.506959	2.794375	1.287417	1.11E-10
IRS1	9.099047	5.614343	-3.4847	1.11E-10
TOPBP1	2.919306	4.365354	1.446048	1.13E-10
TAF1D	3.247619	5.323263	2.075644	1.13E-10
HNF4A	44.61108	32.12652	-12.4846	1.13E-10
EPHX1	1250.189	746.5687	-503.62	1.14E-10
RPL37P6	0.718122	1.989093	1.27097	1.15E-10
RPS3AP20	1.2227	3.020591	1.797891	1.16E-10
WASHC2C	4.124716	5.494207	1.369491	1.16E-10
VPS18	7.379106	9.798012	2.418905	1.17E-10
GRAMD1A	6.567058	13.28723	6.720172	1.19E-10
PHYH	96.30077	58.21604	-38.0847	1.21E-10
HEBP2	5.047953	6.978606	1.930653	1.21E-10
TYROBP	25.82702	53.04068	27.21366	1.21E-10

PLEKHM2	8.613677	12.05787	3.444191	1.22E-10
RPL13P12	9.520238	18.34206	8.821824	1.22E-10
ACAP3	2.969871	4.38617	1.4163	1.23E-10
HGD	168.3976	96.38685	-72.0107	1.23E-10
CWC22	4.515883	6.195074	1.679192	1.25E-10
OGDHL	19.21126	10.55171	-8.65955	1.27E-10
FAM117B	0.814843	2.484362	1.669518	1.27E-10
FZD6	2.428387	4.828956	2.400569	1.27E-10
RRAS	20.18684	35.23895	15.05211	1.27E-10
TMCO4	3.188884	4.28182	1.092936	1.27E-10
MDK	47.41792	121.1802	73.76231	1.28E-10
TPBG	0.466583	2.718989	2.252406	1.28E-10
PRG4	33.7856	14.47285	-19.3128	1.28E-10
PELO	4.354279	6.07247	1.718191	1.29E-10
RARG	1.056625	2.779475	1.72285	1.29E-10
NUDT7	3.1164	1.942566	-1.17383	1.29E-10
PSME3	13.07016	17.46757	4.397414	1.31E-10
RECQL4	2.867034	6.756205	3.889171	1.31E-10
SMIM14	31.86703	21.9198	-9.94723	1.32E-10
ATP5PO	7.616287	12.6943	5.078013	1.32E-10
PERP	32.13916	51.46878	19.32962	1.33E-10
ATP5MG	6.080385	8.301481	2.221097	1.33E-10
EID2	6.132837	8.462769	2.329933	1.33E-10
PIN4	4.550706	6.098228	1.547522	1.33E-10
CFL2	15.43986	10.83313	-4.60673	1.34E-10
RTKN	13.61573	18.87314	5.257408	1.34E-10
MFSD6	1.68818	4.168174	2.479994	1.34E-10
CNKSR1	0.376019	1.947306	1.571287	1.35E-10
INTS13	4.747956	6.976416	2.22846	1.35E-10
ZFP62	2.818273	4.463807	1.645534	1.37E-10
HNF4A-A:	7.981997	3.626192	-4.3558	1.37E-10
RELL1	1.97379	3.72569	1.7519	1.37E-10
BFAR	9.106611	11.59645	2.489842	1.38E-10
RIOK1	4.524549	6.505791	1.981241	1.38E-10
PRELID1	12.1769	15.72494	3.548044	1.39E-10
SDHD	29.51937	22.98288	-6.53649	1.39E-10
ATP6V0E1	87.74022	114.7095	26.96929	1.40E-10
AP000759	1.388024	2.404163	1.016139	1.40E-10
GRSF1	7.092636	9.345883	2.253247	1.41E-10
DNMT1	2.344538	3.92035	1.575812	1.43E-10
FAM160B2	2.845337	4.9344	2.089063	1.44E-10
SLC35A2	8.531799	11.63307	3.101274	1.45E-10
MAL2	14.16997	30.83866	16.66868	1.47E-10
MRPS30	5.117053	6.41071	1.293657	1.47E-10
RPA1	7.690882	10.89147	3.200588	1.47E-10
VDAC1	76.29268	99.21772	22.92504	1.48E-10
TIMMDC1	15.00305	19.00335	4.0003	1.49E-10
MOV10	10.78277	14.0526	3.26983	1.49E-10
PIGQ	3.728537	5.532756	1.804219	1.50E-10
PAFAH1B3	5.306508	18.68144	13.37493	1.50E-10
BCL7B	12.15215	15.18748	3.035332	1.50E-10
UBE2D1	3.153714	4.45515	1.301436	1.50E-10
G6PC	170.2841	64.33931	-105.945	1.52E-10
NCAPG2	1.539548	2.624033	1.084486	1.55E-10
RPL15P3	3.840842	6.141784	2.300942	1.56E-10
TFE3	10.08588	13.64131	3.555435	1.59E-10
TLCD4	16.79793	9.829789	-6.96815	1.59E-10
TMEM87B	2.546943	4.464997	1.918054	1.61E-10

CNOT3	3.90578	5.381934	1.476154	1.62E-10
RYK	2.940679	4.251695	1.311015	1.63E-10
SLC2A9	3.127622	1.59483	-1.53279	1.63E-10
ADH6	60.59351	32.06677	-28.5267	1.64E-10
ZBTB48	3.641501	4.743792	1.102291	1.64E-10
MRPL3	15.02732	19.18859	4.161279	1.68E-10
MAP3K6	1.277278	2.420091	1.142813	1.69E-10
COQ4	9.126286	11.94609	2.819805	1.70E-10
LINC0101ε	8.298715	2.950228	-5.34849	1.70E-10
IRF5	2.411568	3.618826	1.207258	1.70E-10
LARP6	0.630995	1.975842	1.344847	1.71E-10
ALDH9A1	38.4272	27.18053	-11.2467	1.71E-10
PPRC1	4.162784	6.168982	2.006198	1.74E-10
GMNN	14.24383	28.37375	14.12992	1.74E-10
CBX5	2.979735	5.126439	2.146704	1.74E-10
FNBP4	3.544354	5.314155	1.769801	1.76E-10
CENPU	2.32155	4.319409	1.99786	1.76E-10
TMEM214	19.93214	24.82566	4.893514	1.77E-10
SALL1	9.324488	6.228268	-3.09622	1.79E-10
ABR	0.955632	3.276298	2.320666	1.79E-10
ABCG8	10.89069	6.617918	-4.27277	1.79E-10
PLEKHA2	1.364287	2.700184	1.335898	1.80E-10
HDAC11	3.818751	6.164427	2.345676	1.80E-10
SRP68	10.18117	13.02331	2.842144	1.80E-10
TMEM159	0.831118	3.665573	2.834455	1.86E-10
CADM1	10.0197	6.383513	-3.63619	1.89E-10
SFXN1	13.433	10.02707	-3.40593	1.89E-10
RPS28	102.2208	173.9659	71.74516	1.90E-10
TRIM45	0.662318	2.248646	1.586328	1.90E-10
NUDT14	6.864209	11.7591	4.89489	1.91E-10
MORC2	3.063846	4.32174	1.257894	1.92E-10
FAM207A	7.563915	11.45684	3.892922	1.94E-10
PCMT1	10.55318	14.32475	3.771572	1.94E-10
ZDHHC18	5.04146	6.884643	1.843184	1.95E-10
CDC42EP1	51.81385	88.5657	36.75186	1.99E-10
NEPRO	3.285624	4.332714	1.04709	2.02E-10
DDX42	8.845165	11.70214	2.856974	2.05E-10
BCAR1	5.633357	9.345424	3.712067	2.06E-10
PHF1	10.85321	15.12192	4.268709	2.07E-10
TMEM160	8.375348	13.75553	5.38018	2.08E-10
LRP1	30.79687	21.6174	-9.17948	2.10E-10
GIPC1	15.61079	26.60468	10.99388	2.13E-10
ABCB11	13.37458	5.89418	-7.4804	2.16E-10
RUNX1	1.105354	2.14161	1.036256	2.16E-10
ALDH4A1	118.4535	77.48297	-40.9706	2.17E-10
SP1	7.059155	10.31463	3.255475	2.19E-10
SOAT1	4.490331	7.181255	2.690925	2.25E-10
MEX3D	4.272067	6.917433	2.645367	2.25E-10
STX18	4.208927	6.562787	2.35386	2.25E-10
CYBA	13.11093	40.27969	27.16877	2.29E-10
RRP7A	4.973889	7.702608	2.72872	2.31E-10
PAN3	2.144482	3.438278	1.293796	2.32E-10
ARL1	12.1789	15.59861	3.419715	2.32E-10
HMGB1P6	7.11685	10.07425	2.957402	2.33E-10
MIA2	11.29374	8.800548	-2.49319	2.34E-10
CD47	5.482061	9.23046	3.748399	2.35E-10
CD320	9.411839	15.78508	6.373238	2.36E-10
SLC27A5	55.04429	27.09598	-27.9483	2.37E-10

RPS23P8	2.216251	4.022129	1.805878	2.37E-10
TAPBP	58.47821	80.11622	21.63801	2.38E-10
NOL3	5.887104	9.816391	3.929287	2.38E-10
NFKBIB	13.38714	17.69941	4.312277	2.38E-10
MARK2	2.223226	3.674737	1.451511	2.39E-10
MOB1A	8.932977	12.77111	3.838132	2.39E-10
GIGYF1	6.786496	9.252195	2.465699	2.39E-10
POP4	6.3394	8.907629	2.568229	2.40E-10
HNRNPUL	17.63905	24.71288	7.073823	2.46E-10
PPIL1	14.31774	19.72816	5.410425	2.46E-10
CH17-340	7.052421	11.84528	4.792859	2.49E-10
RPL8	518.3063	994.9392	476.6329	2.50E-10
SRF	8.596757	11.43388	2.837126	2.54E-10
ATP5PD	33.8024	44.48444	10.68203	2.54E-10
MRPS9	5.485182	6.769605	1.284423	2.55E-10
RNY4P10	1.415053	2.44153	1.026476	2.56E-10
PECR	36.64802	24.6031	-12.0449	2.56E-10
CFHR5	81.35003	28.715	-52.635	2.56E-10
MELTF	0.696676	4.167447	3.470771	2.56E-10
SCRN1	1.327386	6.130088	4.802701	2.57E-10
OGT	7.054385	10.55054	3.49616	2.57E-10
TUSC1	3.079744	5.069829	1.990084	2.61E-10
ZBTB7A	5.520732	7.532927	2.012195	2.61E-10
TBL3	5.917631	7.651898	1.734267	2.62E-10
ZNF532	0.800496	1.996828	1.196333	2.63E-10
NRAS	11.22212	15.91828	4.696161	2.66E-10
PDE4A	1.074295	2.609114	1.53482	2.66E-10
CDK11B	5.402957	7.093087	1.690131	2.67E-10
HLA-DPB1	14.71978	33.27419	18.55441	2.68E-10
RPL21P28	1.439614	3.724247	2.284633	2.70E-10
NDUFS3	15.41983	20.10155	4.681712	2.75E-10
IRF1	4.168113	7.126708	2.958595	2.75E-10
RAB3IL1	2.039356	4.83032	2.790964	2.76E-10
FXYD5	7.926517	21.21802	13.2915	2.79E-10
HMGN2	30.60221	43.74394	13.14172	2.80E-10
TRPC4AP	25.4981	31.72048	6.222374	2.81E-10
TTC39A	0.784733	3.021522	2.236789	2.82E-10
WIPF2	4.029351	5.639865	1.610514	2.88E-10
EEF1AKMT	6.056344	9.495129	3.438785	2.89E-10
DAO	16.82846	8.58572	-8.24274	2.89E-10
MAP3K7	3.126022	4.293612	1.16759	2.90E-10
PROC	139.0593	94.45974	-44.5995	2.91E-10
RPS20	99.3967	163.2457	63.84901	2.91E-10
CHD3	2.674589	7.032059	4.35747	2.94E-10
IARS1	7.787229	11.20462	3.417394	2.97E-10
NUDT18	2.714841	4.574917	1.860076	2.99E-10
PSMD14	6.633709	8.900105	2.266396	3.00E-10
KDF1	2.069074	4.048448	1.979374	3.04E-10
ARHGAP1	2.9381	3.940965	1.002865	3.04E-10
LINC0249	5.986354	2.768399	-3.21796	3.06E-10
FAM118A	1.82393	3.486353	1.662424	3.06E-10
SNORD10	1.365132	2.604124	1.238993	3.09E-10
TOMM34	12.72022	19.85032	7.130097	3.09E-10
CHIC2	5.78612	8.22935	2.44323	3.10E-10
MAP4K5	1.868212	2.97537	1.107158	3.10E-10
EDEM2	22.6058	28.7394	6.133603	3.16E-10
MYLIP	2.117703	4.202755	2.085052	3.18E-10
TBKBP1	1.544525	2.582963	1.038438	3.18E-10

COA5	7.874888	9.785946	1.911059	3.26E-10
BTG3	5.374064	7.744767	2.370703	3.28E-10
PCNP	18.85009	24.46391	5.613814	3.32E-10
UGT2B15	156.456	84.70396	-71.752	3.34E-10
CBX6	2.527263	5.271085	2.743822	3.35E-10
SLC46A3	14.64684	7.246015	-7.40082	3.37E-10
NGDN	4.754661	5.903862	1.1492	3.38E-10
TMEM167	8.864128	11.44985	2.585722	3.40E-10
HNRNPAE	39.70371	51.02133	11.31762	3.42E-10
TMEM220	11.51944	7.231314	-4.28813	3.47E-10
HAT1	3.379308	4.513126	1.133817	3.47E-10
C1orf174	3.354472	4.408465	1.053993	3.48E-10
AKR1D1	27.84799	9.881016	-17.967	3.49E-10
NARS1	25.34276	33.66011	8.317359	3.52E-10
PFDN6	16.79042	24.0589	7.268483	3.54E-10
AC092306	1.254055	3.470119	2.216064	3.59E-10
MAPK8IP3	2.33112	4.096591	1.765471	3.60E-10
ZNF83	1.454356	3.406207	1.951851	3.63E-10
PPWD1	3.229862	4.300905	1.071043	3.72E-10
TMBIM6	245.8536	197.5549	-48.2986	3.73E-10
NELFCD	12.19123	16.40743	4.2162	3.74E-10
TSPAN14	2.888571	4.519611	1.63104	3.78E-10
HNF1B	3.171894	10.70527	7.533377	3.79E-10
DNM1	0.385158	2.063691	1.678533	3.87E-10
SPATS2L	7.649238	10.87867	3.229431	3.89E-10
PPDPF	110.0996	186.9417	76.8421	3.90E-10
ACSS1	1.5512	4.198225	2.647025	3.90E-10
PARD6B	1.605095	3.178775	1.57368	3.92E-10
RPSAP58	1.027469	2.10803	1.080561	3.94E-10
DMKN	0.756562	5.207591	4.45103	3.96E-10
STK36	1.765129	3.036356	1.271227	3.96E-10
CARMIL1	1.107199	2.445351	1.338153	4.07E-10
DDX11	1.087978	2.515937	1.427959	4.09E-10
SRSF9	7.395188	10.00671	2.61152	4.09E-10
AC115619	32.27858	19.41361	-12.865	4.11E-10
C19orf47	2.055982	3.128753	1.072771	4.11E-10
C4A	35.27187	20.04514	-15.2267	4.12E-10
NPRL2	5.444441	6.868111	1.42367	4.13E-10
GID8	13.98446	18.1512	4.166747	4.17E-10
ADAM9	1.710342	4.281613	2.571271	4.22E-10
LEPROTL1	5.24451	8.107129	2.862619	4.23E-10
MARCHF9	1.936195	3.459722	1.523528	4.24E-10
TBC1D13	7.50479	10.57759	3.0728	4.29E-10
ECM2	6.649445	3.352616	-3.29683	4.31E-10
PSME2	40.04727	55.55894	15.51167	4.31E-10
TGFBRAP1	2.328358	3.561344	1.232986	4.32E-10
RAB3IP	1.091819	2.215053	1.123233	4.33E-10
PLAGL2	1.707852	2.865872	1.158021	4.39E-10
ADGRG1	2.411152	9.756565	7.345413	4.39E-10
MCEE	9.522448	7.195713	-2.32673	4.41E-10
NXF1	8.410635	10.91696	2.506327	4.46E-10
DVL2	3.51809	5.10591	1.58782	4.48E-10
GGA1	4.743637	6.690561	1.946924	4.52E-10
UBE2Q1	20.06138	26.33465	6.273268	4.52E-10
IER5L	1.749778	3.599771	1.849993	4.52E-10
ADAP2	1.371965	2.464666	1.092701	4.57E-10
KLF16	5.069497	7.843061	2.773564	4.60E-10
HMCES	18.36929	22.82403	4.454736	4.72E-10

C6orf132	0.40621	1.923333	1.517124	4.74E-10
AC016747	2.624547	3.639803	1.015257	4.80E-10
HNRNPK	63.53558	77.18915	13.65357	4.82E-10
COA3	40.2914	55.85613	15.56474	4.83E-10
ZNF618	1.363832	2.502773	1.138941	5.04E-10
NSA2	12.12774	15.86126	3.733524	5.04E-10
HADH	10.8505	7.434886	-3.41561	5.05E-10
GOLM1	17.64362	36.22841	18.58479	5.07E-10
UGP2	47.6576	31.11014	-16.5475	5.09E-10
SERPINA6	296.8464	187.5826	-109.264	5.18E-10
PKIG	11.24042	14.97552	3.735099	5.19E-10
MAFG	3.381347	5.153748	1.7724	5.21E-10
GPKOW	14.40015	17.89787	3.497713	5.22E-10
RAP1GAP2	0.51768	1.646416	1.128735	5.41E-10
CD14	259.9032	144.2707	-115.632	5.54E-10
PSMD1	5.68392	6.928147	1.244227	5.55E-10
LYAR	4.390393	6.401819	2.011426	5.58E-10
RAB40C	4.304357	5.883155	1.578798	5.60E-10
AK3	30.44849	21.47248	-8.97601	5.61E-10
AC016596	5.304961	8.527474	3.222513	5.65E-10
ALOX5AP	1.538601	4.725373	3.186772	5.65E-10
E2F3	2.472066	3.981505	1.50944	5.68E-10
ENO2	0.712551	4.094011	3.38146	5.72E-10
GPD1	24.87972	11.58611	-13.2936	5.75E-10
TUBA4A	11.99227	19.99062	7.998355	5.75E-10
SPINT1	6.310932	35.58541	29.27448	5.77E-10
SYVN1	30.60363	23.75157	-6.85205	5.77E-10
RIDA	209.8357	123.4428	-86.393	5.86E-10
TCEAL3	2.037513	4.338765	2.301252	5.92E-10
RHBDF1	2.354959	5.395616	3.040658	5.99E-10
RPL7P9	7.522396	19.44591	11.92351	6.08E-10
MCAT	6.643306	8.901255	2.257948	6.09E-10
ATXN2L	9.709753	13.65703	3.947275	6.13E-10
PLD2	2.941166	4.758784	1.817618	6.14E-10
RAB38	0.377827	1.568222	1.190396	6.16E-10
BSG	98.86012	163.5408	64.68069	6.24E-10
CCDC74A	0.203143	1.506738	1.303595	6.26E-10
MRPS16	23.40965	28.43492	5.025267	6.30E-10
MTX1	5.552866	7.250733	1.697868	6.36E-10
CWC25	4.027069	5.076888	1.049819	6.39E-10
HS2ST1	3.701615	5.153195	1.45158	6.46E-10
WARS2	2.689303	4.263832	1.574529	6.53E-10
ATP5F1C	86.52309	117.7119	31.18883	6.56E-10
METRNL	3.642675	8.205165	4.56249	6.60E-10
NAXE	59.92044	81.0319	21.11146	6.65E-10
SEMA6A	1.041217	4.647597	3.60638	6.65E-10
SPRYD4	4.680106	3.334255	-1.34585	6.72E-10
NDUFC1	11.89831	15.81314	3.914825	6.78E-10
APRT	47.18618	71.77034	24.58417	6.79E-10
USP36	3.917259	5.273468	1.356209	6.89E-10
KDR	5.390853	3.333189	-2.05766	6.91E-10
IWS1	8.824305	11.28509	2.460787	7.07E-10
PIEZO1	5.114203	8.616498	3.502295	7.20E-10
BCAS2	19.4106	24.29677	4.886167	7.23E-10
ACOT1	7.184661	4.107361	-3.0773	7.33E-10
NIPSNAP2	3.259131	5.4759	2.21677	7.36E-10
SLC26A11	3.272054	4.539073	1.267019	7.40E-10
UBE2A	8.189181	10.92573	2.736551	7.50E-10

FLNA	16.22034	47.91617	31.69583	7.58E-10
FADS3	3.381657	5.425417	2.043761	7.60E-10
RALB	10.23878	15.21062	4.971841	7.66E-10
ARNTL2	1.189591	2.879537	1.689947	7.69E-10
GTF2H5	3.811924	5.006851	1.194927	7.78E-10
IER2	11.34525	17.59836	6.253113	7.78E-10
MADD	3.853847	5.283127	1.42928	7.94E-10
KATNB1	3.627995	4.874096	1.2461	7.95E-10
HNF1A-A!	2.878445	4.438769	1.560324	7.97E-10
CLCF1	1.896829	5.308341	3.411512	8.04E-10
CALU	13.89327	22.08829	8.195021	8.09E-10
CITED4	17.34362	53.94545	36.60183	8.17E-10
GMPS	4.848724	6.565082	1.716358	8.20E-10
SMYD3	2.480352	4.176217	1.695865	8.40E-10
PARD3	6.798357	10.23172	3.433363	8.41E-10
ARHGEF1(	2.411186	5.537081	3.125895	8.44E-10
AC132812	1.287486	2.657955	1.370469	8.46E-10
FCSK	2.986852	4.005251	1.018399	8.47E-10
AGER	1.012878	2.478729	1.465851	8.48E-10
TIPRL	12.96981	16.77551	3.8057	8.62E-10
HYAL1	45.73025	31.71203	-14.0182	8.64E-10
B3GALT4	1.231451	2.619679	1.388228	8.67E-10
EZH1	2.961089	4.065419	1.10433	8.72E-10
HSF4	2.329633	4.444013	2.114379	8.74E-10
SLC7A1	1.078095	3.056315	1.97822	8.79E-10
MYL12B	174.9844	229.836	54.85153	8.79E-10
ACKR2	2.26115	1.068743	-1.19241	8.83E-10
SEM1	9.589104	14.44325	4.854148	8.85E-10
ITGAM	1.342519	3.032229	1.689709	8.86E-10
SYNCRIP	6.911298	8.928079	2.016782	8.89E-10
FNBP1L	4.834926	8.616537	3.781611	8.94E-10
AP000355	4.96849	2.320432	-2.64806	8.98E-10
ADI1	82.31674	54.83976	-27.477	8.98E-10
RNF166	1.836383	2.862464	1.026081	9.01E-10
CDC42BP(	0.707443	2.566522	1.859078	9.16E-10
ZDHHC16	8.627377	11.2096	2.582224	9.17E-10
GTF2B	8.119633	10.11055	1.990914	9.18E-10
VPS39	5.036626	6.738535	1.701909	9.25E-10
PEBP1	922.4829	694.0565	-228.426	9.26E-10
TSNAX	4.849561	6.260325	1.410764	9.28E-10
ROGDI	2.482316	3.589697	1.107381	9.30E-10
GNE	15.08707	10.00661	-5.08046	9.31E-10
TNFRSF4	1.685962	3.107564	1.421602	9.34E-10
LZIC	5.426062	7.016562	1.5905	9.39E-10
ATP5F1EP	1.669245	3.082389	1.413144	9.50E-10
H2AZ2	15.62694	19.79094	4.163994	9.52E-10
CRYL1	38.27006	23.07494	-15.1951	9.57E-10
C8B	127.5919	79.96552	-47.6264	9.58E-10
LMO4	4.242362	7.466368	3.224006	9.63E-10
SOWAHB	6.086367	3.932103	-2.15426	9.65E-10
MTCH1	43.34282	56.08914	12.74631	9.69E-10
AC004453	2.653075	4.59725	1.944175	9.71E-10
GHR	9.907957	4.792776	-5.11518	9.74E-10
NAGPA	3.490817	4.76225	1.271433	9.74E-10
TMEM126	16.59818	20.74801	4.149828	9.76E-10
NOL11	7.958978	10.31792	2.358937	9.79E-10
ACSS3	5.918206	3.024208	-2.894	9.87E-10
ORAI1	9.29465	12.87908	3.584431	9.93E-10

AC018638	4.798483	7.438916	2.640433	9.94E-10
LRFN4	0.399202	1.865192	1.46599	9.96E-10
CTCF	3.404864	4.41828	1.013416	1.00E-09
RPS7P1	5.344302	8.891743	3.547441	1.00E-09
ENPP5	0.574289	2.025246	1.450958	1.02E-09
MGST1	89.75389	60.31332	-29.4406	1.02E-09
MAGED2	39.51391	54.51127	14.99736	1.03E-09
LINC0204:	0.50692	2.627882	2.120962	1.03E-09
HOOK2	3.066738	5.624004	2.557266	1.03E-09
GABBR1	0.417217	2.332555	1.915338	1.03E-09
NUDT16L:	13.36295	17.33803	3.975087	1.04E-09
RAP1GAP	7.090815	19.7734	12.68259	1.05E-09
CDC16	10.75218	14.27102	3.518839	1.05E-09
DDX50	7.765234	10.43398	2.668743	1.06E-09
INHBC	41.14278	23.94475	-17.198	1.07E-09
ITGB4	1.801716	15.00313	13.20141	1.07E-09
ARNT2	0.44552	2.110816	1.665296	1.08E-09
METTL17	3.425584	4.644507	1.218923	1.09E-09
R3HCC1	8.945538	11.92556	2.980019	1.11E-09
STARD7	27.74226	34.93517	7.192909	1.12E-09
MAP3K1	2.247966	4.14418	1.896214	1.13E-09
TNFAIP8L:	1.668633	3.604874	1.936241	1.13E-09
ANXA3	0.358257	3.78649	3.428233	1.14E-09
LINC0099:	0.2689	1.577712	1.308812	1.14E-09
MLLT1	9.848324	13.11401	3.265682	1.15E-09
NCOR2	5.865542	8.868571	3.003029	1.16E-09
TRAF4	8.836344	11.72879	2.892448	1.17E-09
DNPEP	11.41213	13.7528	2.340676	1.18E-09
MAGED1	24.59928	39.30617	14.70689	1.20E-09
HAX1	46.63734	59.90468	13.26734	1.21E-09
PNN	12.1214	16.54607	4.424661	1.22E-09
HLA-A	339.5205	538.109	198.5885	1.23E-09
AL390728.	2.738397	6.270513	3.532116	1.23E-09
MTM1	4.456247	3.206648	-1.2496	1.23E-09
SEL1L	18.1659	13.24392	-4.92198	1.24E-09
JUP	34.55288	55.375	20.82212	1.25E-09
MAML1	3.588451	5.122411	1.53396	1.26E-09
PLIN3	9.137719	15.47077	6.333047	1.27E-09
ZNNT1	1.38026	2.408073	1.027814	1.28E-09
TUBA1C	4.882882	8.709664	3.826782	1.30E-09
TMEM183	6.816581	8.592951	1.77637	1.30E-09
TGFA	0.834323	3.917321	3.082998	1.31E-09
AL118516.	3.0279	4.756692	1.728792	1.31E-09
PFKFB3	4.166737	15.09566	10.92892	1.31E-09
GBP2	10.81444	18.1866	7.372161	1.32E-09
AC113935	22.64325	34.36324	11.71999	1.33E-09
MDC1	3.365943	4.937131	1.571188	1.34E-09
CCND3	4.648315	7.535466	2.887151	1.36E-09
COX6B1	137.1735	238.0652	100.8917	1.37E-09
F5	74.72218	47.84831	-26.8739	1.40E-09
AC024293	8.36354	16.18194	7.818401	1.40E-09
DSC2	0.940769	1.945188	1.004419	1.40E-09
FRAT2	7.031672	10.46744	3.435764	1.41E-09
PIGT	6.63335	8.59779	1.96444	1.42E-09
PBK	2.142295	4.531698	2.389403	1.44E-09
SMTN	2.459993	4.606264	2.14627	1.44E-09
TSPAN15	4.807233	22.00072	17.19348	1.45E-09
NCEH1	1.474367	7.113771	5.639404	1.47E-09

DMAC1	6.225226	9.264845	3.039619	1.47E-09
DHX9	16.05006	21.91473	5.864669	1.47E-09
EMC10	8.57504	11.45247	2.877431	1.49E-09
BLOC1S1	4.39438	8.440905	4.046525	1.50E-09
APOBEC3(	0.80682	1.875176	1.068356	1.50E-09
FABP5	1.24175	3.428619	2.186869	1.51E-09
ZNF468	1.412744	2.557395	1.144651	1.52E-09
CARS1	3.304578	4.579973	1.275395	1.53E-09
PTAFR	1.145307	3.166269	2.020962	1.54E-09
SLC38A3	168.2871	105.6003	-62.6869	1.54E-09
AL353572.	1.612675	3.73495	2.122274	1.55E-09
ANXA2P2	2.065761	4.215213	2.149452	1.57E-09
SLC39A10	1.42813	2.509033	1.080902	1.57E-09
POLM	3.393514	4.408902	1.015388	1.58E-09
NIBAN2	10.91994	26.92619	16.00625	1.59E-09
ABHD17A	3.243717	4.938121	1.694404	1.60E-09
WASHC2A	5.36156	7.129748	1.768188	1.60E-09
RAB34	4.309686	12.63823	8.328547	1.61E-09
NCLN	22.14995	27.94291	5.792965	1.62E-09
GLS	3.259234	5.981342	2.722108	1.65E-09
SMNDC1	3.154577	4.230956	1.076379	1.65E-09
SIPA1L3	2.579192	4.548353	1.969161	1.65E-09
LINC0276(	2.126873	0.746873	-1.38	1.66E-09
LRP10	14.97455	23.47918	8.504631	1.66E-09
ENTPD6	8.555265	11.90579	3.35052	1.67E-09
EPRS1	18.2855	25.48775	7.202246	1.68E-09
PSMB8-AS	1.548005	2.674176	1.12617	1.68E-09
TAP2	2.87893	4.629615	1.750685	1.69E-09
ATP5MD	72.65704	99.40195	26.74491	1.69E-09
RSRP1	3.321668	5.029403	1.707734	1.69E-09
NONO	22.7368	30.75098	8.014178	1.70E-09
MBD1	5.892802	7.551935	1.659133	1.73E-09
UBA1	35.30012	44.64422	9.344094	1.73E-09
HMMR	1.691539	3.121823	1.430284	1.73E-09
CD300A	1.516811	2.898959	1.382147	1.74E-09
EPOR	1.89443	3.327579	1.433149	1.74E-09
CCDC92	3.81792	5.183033	1.365113	1.74E-09
ST3GAL1	20.42974	12.24661	-8.18313	1.75E-09
UGT2B10	94.2741	46.63457	-47.6395	1.75E-09
TIMM8B	25.81871	34.96727	9.148558	1.76E-09
PRSS16	0.191448	1.565045	1.373597	1.76E-09
ZNF358	14.59347	24.05645	9.462977	1.80E-09
FAM234A	12.65302	17.08896	4.43594	1.82E-09
CALM3	29.20105	39.13022	9.929169	1.82E-09
RALGAPB	2.669633	3.695011	1.025378	1.82E-09
PCGF3	2.59734	3.926149	1.328809	1.83E-09
CRMP1	0.83812	2.531828	1.693708	1.83E-09
NCOA4	73.08875	54.51275	-18.576	1.83E-09
AC092868	0.699144	2.190428	1.491284	1.84E-09
ORMDL2	4.160324	5.307909	1.147586	1.84E-09
CNNM4	1.927369	3.772593	1.845224	1.84E-09
ITIH2	786.8993	480.3329	-306.566	1.87E-09
KCNJ11	0.798025	2.064166	1.266141	1.88E-09
SRD5A1	7.634404	4.541218	-3.09319	1.90E-09
HIBADH	54.42241	35.15636	-19.2661	1.90E-09
CARHSP1	9.382533	12.75536	3.372825	1.91E-09
STK26	1.799308	3.422046	1.622737	1.94E-09
HILPDA	1.549511	4.521374	2.971864	1.94E-09

MMGT1	4.043391	5.809356	1.765965	1.97E-09
B4GALT5	9.142637	16.44357	7.300931	1.98E-09
TRNP1	6.992231	20.23065	13.23842	1.99E-09
BAZ2A	4.402855	6.574337	2.171482	2.00E-09
EIF2AK1	30.2185	36.84959	6.631097	2.00E-09
AL390719.	0.133701	1.4581	1.324399	2.01E-09
SYK	1.197433	3.435062	2.237629	2.01E-09
DCAF13	3.503834	5.251111	1.747276	2.02E-09
COLCA2	0.860729	2.203107	1.342378	2.03E-09
TUSC3	1.740875	5.383147	3.642271	2.06E-09
SGPP2	0.635222	5.663978	5.028756	2.06E-09
BAIAP2L2	4.610714	14.65085	10.04013	2.07E-09
TEAD3	6.995873	10.13683	3.140958	2.11E-09
SLC16A3	1.26677	4.251552	2.984782	2.11E-09
GOLPH3L	7.091259	10.69664	3.605379	2.12E-09
AC040977	2.688774	4.708637	2.019863	2.13E-09
TSKU	70.00072	39.73077	-30.2699	2.14E-09
TRMT12	3.843884	5.147031	1.303147	2.14E-09
PSMD2	29.55084	38.76872	9.217888	2.14E-09
ASAP1	2.882619	5.038	2.15538	2.19E-09
CDC26	5.326412	6.77254	1.446128	2.22E-09
SIN3B	2.837491	3.932984	1.095494	2.23E-09
MKKS	4.500917	7.315346	2.814429	2.28E-09
LAMA5	4.301476	10.58839	6.286917	2.29E-09
SERPINA4	131.2235	80.15465	-51.0688	2.29E-09
ZC3HC1	5.310859	6.563072	1.252213	2.31E-09
GALNT10	1.641238	3.409537	1.768299	2.32E-09
APPL2	4.481146	7.126242	2.645096	2.33E-09
STAMBP	3.65801	4.708061	1.05005	2.34E-09
TREM2	3.499105	8.58071	5.081605	2.35E-09
CXorf56	5.59954	7.416163	1.816623	2.35E-09
AIP	26.87396	36.04203	9.16807	2.36E-09
SRPRB	18.30103	23.63485	5.333815	2.38E-09
THG1L	3.170402	4.289974	1.119572	2.39E-09
PNRC2	9.687883	12.68942	3.001534	2.40E-09
EIF2D	7.124215	9.227828	2.103613	2.40E-09
COTL1	4.69434	11.30855	6.614205	2.41E-09
ZFYVE19	6.030943	7.825027	1.794084	2.43E-09
THAP7	8.57696	10.90312	2.326162	2.43E-09
PPP4R1	4.692822	6.313783	1.620961	2.46E-09
DHX40	3.192505	4.884205	1.6917	2.46E-09
RAB9A	7.288767	10.43521	3.146444	2.47E-09
RAB22A	3.51266	4.623732	1.111071	2.48E-09
RPL13AP5	9.693378	16.54278	6.849398	2.49E-09
PRPF40A	5.82048	7.630804	1.810324	2.52E-09
ETNPPL	31.49026	11.78664	-19.7036	2.55E-09
SPINT1-A:	0.374742	1.545142	1.1704	2.55E-09
TAF7	26.04811	35.85669	9.808581	2.58E-09
CSTB	32.46421	47.6556	15.19139	2.61E-09
NOL6	5.964794	7.890393	1.9256	2.62E-09
PKN1	19.98135	39.57219	19.59084	2.62E-09
CERCAM	1.051344	4.000001	2.948657	2.63E-09
BICC1	2.520065	12.03549	9.51542	2.64E-09
C5orf15	25.8369	34.5668	8.7299	2.64E-09
PCYT1A	4.81052	5.973651	1.163131	2.67E-09
MED4	6.013621	7.492461	1.478839	2.67E-09
GTF2IRD1	2.052855	3.109218	1.056364	2.68E-09
UXS1	4.48934	6.247311	1.757971	2.69E-09

AP001781	1.987431	0.985167	-1.00226	2.70E-09
WDR76	1.390424	2.510802	1.120378	2.70E-09
TMEM203	21.25707	25.93533	4.678261	2.70E-09
COX7A2	49.74174	67.19592	17.45417	2.71E-09
ZDHHC1	1.068276	4.07071	3.002434	2.77E-09
NSF	5.995571	7.795816	1.800245	2.77E-09
SUCO	4.396569	6.958427	2.561857	2.80E-09
ATP5MPL	16.62297	22.42834	5.805369	2.80E-09
PLA2G12E	31.15566	21.14777	-10.0079	2.81E-09
SPOP	6.8063	8.266052	1.459753	2.82E-09
UBE2O	4.194504	5.459988	1.265484	2.84E-09
MHENCR	3.42355	6.236263	2.812713	2.84E-09
SLC39A6	7.212797	14.48052	7.267724	2.85E-09
ANAPC11	21.61983	32.91064	11.2908	2.86E-09
IFT20	3.953212	5.06193	1.108718	2.86E-09
ZBTB4	4.138028	7.39013	3.252101	2.87E-09
DSTYK	1.307546	2.435685	1.128139	2.87E-09
ZNF664	12.73197	18.7366	6.004632	2.91E-09
RGL2	7.891005	12.619	4.728	2.92E-09
TRAF5	0.632238	1.730392	1.098153	2.93E-09
COMMD5	9.858629	13.59486	3.736229	3.00E-09
CTPS2	3.090978	4.398181	1.307202	3.00E-09
STYXL1	11.56229	16.75849	5.196203	3.03E-09
AC093323	2.992662	4.083824	1.091162	3.05E-09
MAP2K7	5.950113	7.403949	1.453836	3.06E-09
POGK	5.332173	8.757658	3.425485	3.06E-09
MARVELD	2.099369	5.366268	3.266899	3.06E-09
MTMR14	6.900974	8.388008	1.487033	3.09E-09
CD59	16.58846	25.89	9.301535	3.13E-09
AC136601	2.274337	0.674147	-1.60019	3.15E-09
SAR1A	12.39929	14.89084	2.491551	3.15E-09
ABHD14A	2.611343	3.857284	1.245941	3.16E-09
MIA3	13.73927	10.10823	-3.63105	3.17E-09
DCAF15	5.636406	8.666082	3.029676	3.20E-09
HSPE1	19.11494	29.7479	10.63296	3.22E-09
LMCD1	1.574992	2.779199	1.204206	3.27E-09
MGAT5	2.305961	5.498071	3.19211	3.31E-09
MOCS1	10.88533	7.542253	-3.34308	3.33E-09
KLHL42	1.696362	3.055436	1.359074	3.33E-09
RPL39L	4.387905	10.39502	6.007116	3.33E-09
C2CD5	2.746006	4.049247	1.303241	3.34E-09
CS	12.04959	18.302	6.252409	3.39E-09
PDXK	6.869713	9.516442	2.646729	3.41E-09
P4HTM	2.392599	3.723878	1.33128	3.42E-09
UBLCP1	6.465836	8.387371	1.921535	3.43E-09
R3HDM2	4.938702	6.214224	1.275521	3.43E-09
SLC2A6	2.381774	4.796582	2.414808	3.44E-09
SUPT6H	10.33111	12.81408	2.482964	3.46E-09
INTS14	5.975784	7.451498	1.475714	3.48E-09
RNF144A	0.811987	1.841081	1.029094	3.48E-09
NOXA1	7.101761	12.26247	5.160705	3.48E-09
POLR2L	81.32041	116.2068	34.88635	3.50E-09
TMEM39A	3.328263	4.356192	1.027929	3.50E-09
SELENON	7.949329	12.15751	4.208186	3.51E-09
PDIA3	122.4327	154.0887	31.65604	3.52E-09
PTPN12	7.000345	10.28593	3.285587	3.54E-09
NDUFAF8	16.64546	27.41103	10.76557	3.56E-09
TNFRSF21	7.720861	21.1355	13.41464	3.58E-09

ANKRD27	2.076803	3.494062	1.417259	3.60E-09
JAGN1	24.28312	30.59727	6.314142	3.60E-09
TMEM219	40.03125	54.02996	13.99871	3.61E-09
COA6-AS:	2.310068	4.061556	1.751488	3.62E-09
ME3	1.294088	2.728174	1.434086	3.64E-09
NUBP2	10.90209	13.65853	2.756441	3.64E-09
GLIS2	1.962088	4.789444	2.827356	3.65E-09
TSR2	10.26977	12.6169	2.347133	3.65E-09
ANKRD10	9.323363	16.86577	7.542402	3.66E-09
KHK	45.43006	27.50827	-17.9218	3.68E-09
LGALS1	79.9656	201.0086	121.043	3.68E-09
TRPM4	2.599672	4.739253	2.13958	3.69E-09
AP001324	8.781985	15.77767	6.99568	3.71E-09
SNW1	13.09772	16.31775	3.220033	3.71E-09
C4orf48	2.013791	7.395627	5.381836	3.73E-09
UQCRB	13.90879	20.05021	6.141415	3.75E-09
NEDD4	5.789584	3.753943	-2.03564	3.75E-09
AGXT	335.7511	201.7014	-134.05	3.78E-09
PIP4K2C	8.74666	12.40108	3.654425	3.79E-09
PROZ	13.77779	7.327348	-6.45044	3.82E-09
STAT5A	2.378424	3.974793	1.596369	3.93E-09
HSD11B1	285.6326	96.9943	-188.638	3.98E-09
SH3BP5L	6.2828	8.409391	2.126592	4.08E-09
AC107956	2.131867	3.431604	1.299737	4.14E-09
HSDL2	44.81283	31.53739	-13.2754	4.15E-09
H2AW	1.931515	6.850729	4.919214	4.16E-09
LRRC47	9.069538	11.07196	2.002424	4.20E-09
SLC52A3	0.251212	1.425096	1.173884	4.41E-09
APEX1	52.23909	66.64389	14.4048	4.44E-09
PGAP4	1.252783	2.944476	1.691693	4.46E-09
KLHDC3	53.24004	70.16887	16.92883	4.46E-09
PHB	12.96679	16.65133	3.684545	4.47E-09
GALNT18	4.618113	9.360563	4.74245	4.48E-09
EHBP1	5.532739	4.033433	-1.49931	4.51E-09
ZIC2	1.314101	2.876186	1.562085	4.51E-09
MAP4	10.69453	14.63336	3.93883	4.53E-09
CTF1	0.451357	1.509993	1.058635	4.55E-09
WNT7B	0.103882	2.066547	1.962665	4.56E-09
SMC2	1.83057	2.957996	1.127426	4.57E-09
EXOSC10	3.728557	4.89501	1.166453	4.58E-09
C19orf25	4.970434	6.789708	1.819274	4.59E-09
RNH1	17.4292	22.07095	4.641753	4.59E-09
RPL21P16	5.107011	10.96921	5.862198	4.64E-09
AQP9	168.392	73.696	-94.696	4.66E-09
NOB1	8.222023	11.41779	3.195769	4.66E-09
TPMT	21.9097	16.4994	-5.4103	4.66E-09
ASL	20.31704	11.60727	-8.70977	4.67E-09
OTUD1	2.863356	4.04663	1.183273	4.69E-09
ZNF688	2.832628	3.86959	1.036963	4.79E-09
DAZAP2	32.91122	42.37283	9.461616	4.81E-09
EML2	2.380987	3.884916	1.503929	4.83E-09
SERPINB1	21.91053	30.97057	9.060044	4.86E-09
NMB	2.774001	8.829353	6.055352	4.86E-09
INTS1	11.76002	14.97459	3.214576	4.87E-09
CEACAM1	13.77414	9.607613	-4.16653	4.88E-09
TLE5	20.09849	28.0695	7.971013	4.89E-09
CDK9	16.00412	20.35472	4.3506	4.91E-09
PSME1	102.3736	130.5499	28.17633	4.92E-09

HAPLN3	0.685582	1.97051	1.284928	4.93E-09
SH3PXD2E	1.537977	3.282399	1.744422	4.94E-09
ELF3	7.585574	15.29047	7.704895	4.95E-09
TWF2	6.43363	9.547818	3.114188	4.98E-09
RSRC2	4.374256	5.662386	1.28813	5.00E-09
CFHR1	219.2277	129.733	-89.4947	5.01E-09
ESCO1	2.760988	3.770705	1.009717	5.11E-09
SLC17A4	13.89121	8.527638	-5.36357	5.14E-09
MTCL1	0.337768	2.01686	1.679092	5.15E-09
ST3GAL6	4.47721	2.826196	-1.65101	5.20E-09
ANXA4	15.88529	36.00854	20.12325	5.20E-09
IFFO2	1.945559	3.858409	1.91285	5.22E-09
DYRK1B	4.049763	7.133092	3.08333	5.28E-09
SERPINH1	12.90762	26.68505	13.77743	5.31E-09
FAM53B	2.056563	3.398292	1.341729	5.36E-09
GAL3ST1	3.882095	14.97827	11.09617	5.41E-09
BAIAP2L1	10.1187	15.15601	5.037316	5.50E-09
INPPL1	9.27742	12.71566	3.438237	5.50E-09
ZNF282	7.601834	9.866642	2.264808	5.50E-09
CUL7	4.906332	7.018835	2.112504	5.54E-09
CORO2A	1.548483	3.408277	1.859794	5.55E-09
ELP6	5.365093	6.634741	1.269648	5.56E-09
FLAD1	12.44827	16.70624	4.257969	5.59E-09
PALMD	5.785304	3.61755	-2.16775	5.59E-09
DESI2	6.12967	8.741905	2.612234	5.64E-09
PIP4K2A	3.631416	5.566217	1.934801	5.66E-09
GSPT1	10.00499	12.34733	2.342337	5.67E-09
CCNL2	10.43362	15.2882	4.854577	5.73E-09
TP53	5.264847	8.274813	3.009966	5.74E-09
SLC6A8	3.013224	10.49574	7.482513	5.81E-09
IFI16	3.9013	8.261409	4.360109	5.83E-09
SPAG5	3.825417	6.545754	2.720337	5.86E-09
GFM2	7.131272	5.467198	-1.66407	5.87E-09
PUS7	2.569552	3.819328	1.249776	5.87E-09
GPAM	26.19561	14.79957	-11.396	5.87E-09
VEGFA	8.905678	13.70669	4.801007	6.00E-09
ZNF672	10.73319	13.65568	2.922494	6.09E-09
SYDE1	1.7825	3.309424	1.526924	6.14E-09
HTATSF1	17.15263	22.69479	5.542165	6.14E-09
GRK2	9.586514	13.44485	3.858337	6.14E-09
SMIM15	14.22321	17.59544	3.372231	6.17E-09
DDX54	10.32807	12.81557	2.487496	6.22E-09
UGT1A4	30.84116	10.36876	-20.4724	6.22E-09
GNL3	16.58034	21.92961	5.349274	6.26E-09
REXO2	4.909829	6.406604	1.496775	6.27E-09
STK4	2.596736	3.625177	1.028441	6.29E-09
ZBTB2	3.114953	4.170033	1.055081	6.31E-09
FLVCR1	2.141278	3.297059	1.155781	6.33E-09
PHLDA3	5.346849	12.19822	6.851373	6.45E-09
SGSM2	1.979264	3.224765	1.245501	6.53E-09
SBNO2	6.183122	9.046743	2.863621	6.54E-09
SLC30A6	4.201085	5.536112	1.335027	6.66E-09
VSIG10	3.273814	5.158272	1.884458	6.69E-09
GNG5	4.052576	5.908227	1.855651	6.73E-09
EPS8L1	0.428527	3.921124	3.492598	6.77E-09
UBTD2	6.005019	7.853133	1.848114	6.83E-09
TPD52	5.343399	8.0789	2.7355	6.89E-09
FGFR1	0.812704	2.88027	2.067567	6.90E-09

TLCD1	7.165929	11.49702	4.331087	6.93E-09
NDUFB10	75.4296	104.1826	28.75301	6.93E-09
RPL36AL	187.8081	242.8653	55.05724	6.96E-09
LENG8	9.382825	15.49639	6.113568	7.01E-09
RPL22L1	16.34346	30.85723	14.51377	7.02E-09
KCTD17	3.414468	8.735458	5.32099	7.08E-09
EMC1	3.993603	5.306388	1.312785	7.09E-09
AL441992.	2.701345	4.186711	1.485367	7.15E-09
COA6	9.753709	13.74094	3.987227	7.22E-09
RNF187	45.89297	62.76233	16.86936	7.27E-09
UBASH3B	0.277248	1.850901	1.573653	7.38E-09
SPAG1	1.334427	2.339394	1.004967	7.41E-09
AL627309.	0.723736	1.811479	1.087743	7.43E-09
TBC1D20	5.65741	7.145122	1.487712	7.50E-09
PSMD4	76.13749	106.119	29.98155	7.53E-09
HNMT	25.76752	19.38194	-6.38558	7.55E-09
MRPL50	5.876753	7.785369	1.908617	7.62E-09
CCDC34	3.229767	5.412093	2.182326	7.67E-09
PARP6	1.671264	2.94494	1.273676	7.75E-09
NDUFA3	22.16655	34.23171	12.06516	7.82E-09
SCAND1	27.22859	37.7399	10.51131	7.86E-09
KHSRP	25.08586	32.48381	7.397958	7.90E-09
RNY3P16	0.844199	1.932494	1.088296	7.96E-09
ITGA3	1.105496	8.960751	7.855256	7.98E-09
SLC23A2	13.56075	7.898091	-5.66266	8.00E-09
HSF1	13.64635	18.90506	5.258711	8.01E-09
CTTNBP2N	1.142992	2.415613	1.272622	8.04E-09
AKR7A3	67.55503	35.41916	-32.1359	8.06E-09
ZNF638	5.223807	6.521174	1.297367	8.12E-09
ARHGEF1	6.613838	12.10192	5.488077	8.13E-09
PDE3B	3.061761	1.990326	-1.07144	8.18E-09
ARRDC2	9.448728	23.35424	13.90551	8.20E-09
PPP3CB	5.098505	6.620373	1.521867	8.23E-09
EHF	3.197667	9.610262	6.412596	8.34E-09
UFD1	4.481834	5.522914	1.041079	8.48E-09
GPD1L	1.545774	2.831691	1.285917	8.51E-09
PCAT6	1.68148	2.836383	1.154903	8.52E-09
USP11	7.533704	10.55488	3.021177	8.60E-09
DEPDC1B	0.893317	3.926112	3.032796	8.61E-09
CLDND1	4.600103	5.79065	1.190546	8.64E-09
PDCD10	5.61778	7.347415	1.729635	8.67E-09
ARHGAP1	2.192246	3.697455	1.505209	8.74E-09
ATXN7L3	7.978434	10.67723	2.698792	9.17E-09
HPCAL1	6.760242	8.596499	1.836257	9.27E-09
TXN	243.3231	361.2409	117.9178	9.27E-09
GLI4	2.773277	4.14001	1.366733	9.28E-09
BTD	5.752635	3.936673	-1.81596	9.40E-09
MIR4292	1.503433	2.934892	1.431459	9.47E-09
SEPTIN8	4.02505	6.034348	2.009298	9.67E-09
ELMO3	3.836738	10.61627	6.779534	9.67E-09
FXR1	6.866982	8.604906	1.737924	9.71E-09
ING4	7.890128	10.41586	2.525732	9.81E-09
DBR1	2.805999	3.819741	1.013743	9.85E-09
RBPMS	4.529028	8.323852	3.794824	9.96E-09
EIF3H	25.04051	34.5826	9.54209	9.98E-09
S100A6	45.56768	361.7864	316.2187	9.98E-09
NT5DC2	3.266617	8.538891	5.272274	9.99E-09
GADD45A	39.16159	25.77321	-13.3884	1.00E-08

RPL13AP2	5.346194	14.1746	8.828402	1.01E-08
ADH1C	347.0481	167.6722	-179.376	1.01E-08
HMGCS1	27.61888	15.61367	-12.0052	1.01E-08
ESRP1	1.141894	5.681923	4.540029	1.04E-08
THNSL1	4.504844	3.381568	-1.12328	1.04E-08
NUP210	6.317063	9.699322	3.38226	1.04E-08
TIMM10	37.61372	51.0452	13.43148	1.05E-08
AC021146	12.91016	5.861189	-7.04897	1.06E-08
TMEM99	3.252889	5.074251	1.821362	1.07E-08
ARMCX2	0.737518	2.838831	2.101312	1.08E-08
SYMPK	8.151688	12.03459	3.882901	1.09E-08
DDR1	3.832674	13.82062	9.987945	1.09E-08
CENPQ	2.305105	3.421654	1.116549	1.10E-08
GLYCTK	31.33948	20.56813	-10.7714	1.10E-08
MFAP3L	2.664259	1.329972	-1.33429	1.11E-08
GOT2	76.84991	52.76741	-24.0825	1.13E-08
H3-3A	5.234736	6.902975	1.668239	1.13E-08
ASPDH	30.64114	15.46344	-15.1777	1.13E-08
CTHRC1	3.0837	10.32893	7.245225	1.14E-08
RDH14	7.687134	9.130968	1.443834	1.14E-08
TUBB6	3.095782	9.011149	5.915367	1.14E-08
UNC119B	2.016599	3.22825	1.211651	1.15E-08
CNP	11.80411	14.96138	3.157269	1.15E-08
ITGB8	0.112463	1.198812	1.08635	1.16E-08
PPP1R13L	2.609826	7.196611	4.586785	1.17E-08
AC108673	2.049985	3.18741	1.137425	1.19E-08
CYP2J2	28.31807	18.16799	-10.1501	1.19E-08
SRD5A2	6.51485	1.976102	-4.53875	1.19E-08
SLC17A2	18.74793	9.562139	-9.18579	1.19E-08
EXOC4	4.637634	5.774066	1.136432	1.19E-08
CCDC9B	0.998528	3.437122	2.438594	1.20E-08
SEMA4B	5.23213	8.412097	3.179967	1.20E-08
VEZT	3.570467	5.458515	1.888048	1.20E-08
UQCR10	28.55841	43.52697	14.96856	1.21E-08
SLC35F2	0.421771	1.897023	1.475251	1.23E-08
PDSS1	2.657981	3.78968	1.1317	1.23E-08
PITHD1	13.45314	17.1342	3.681066	1.23E-08
PRRC2A	16.98765	23.45751	6.469865	1.25E-08
TIMM44	7.164879	9.016009	1.85113	1.26E-08
KIF12	6.847784	25.09425	18.24646	1.26E-08
PDP1	1.033629	3.070318	2.036689	1.27E-08
RIC8B	1.509588	2.860991	1.351402	1.29E-08
IMP3	7.243177	8.921513	1.678336	1.30E-08
CNOT8	7.224872	9.08841	1.863538	1.33E-08
RABGGTA	6.327391	7.621591	1.2942	1.34E-08
SLIRP	11.88054	16.43163	4.551093	1.34E-08
XYLB	7.13137	5.013916	-2.11745	1.35E-08
ZNF3	4.633615	5.722355	1.08874	1.36E-08
MRPL14	66.85122	94.13028	27.27906	1.36E-08
IL27RA	1.995901	4.007428	2.011527	1.36E-08
NNT	14.27334	9.423608	-4.84973	1.37E-08
PATJ	2.734871	4.3203	1.58543	1.38E-08
SMAD3	5.326282	8.394174	3.067892	1.39E-08
ARMC10	4.234959	5.693302	1.458344	1.40E-08
STRAP	32.85905	41.47307	8.614015	1.40E-08
CCDC86	3.201357	4.531291	1.329934	1.41E-08
AMDHD2	3.21039	4.391071	1.180681	1.43E-08
SPINT2	1.347462	6.571879	5.224418	1.44E-08

CABLES2	2.052123	3.228764	1.17664	1.45E-08
BCLAF1	5.360947	6.790018	1.429071	1.46E-08
STX2	2.782345	4.093491	1.311146	1.46E-08
KANSL3	4.711089	6.408125	1.697036	1.47E-08
NRARP	1.821464	4.829423	3.007959	1.49E-08
IQGAP3	2.328381	4.08763	1.759248	1.49E-08
GMFG	6.808891	13.24226	6.43337	1.49E-08
AZGP1	352.5194	208.0804	-144.439	1.52E-08
GGT7	5.265093	7.883255	2.618162	1.53E-08
KIFC3	4.694772	7.820998	3.126226	1.53E-08
RASA3	1.724926	3.485319	1.760393	1.54E-08
COG1	3.581283	4.666806	1.085523	1.55E-08
PRR12	2.553946	3.976509	1.422564	1.55E-08
PFKFB1	9.521807	5.13458	-4.38723	1.55E-08
TYMS	1.303648	2.310299	1.00665	1.55E-08
SEC13	18.15152	22.83268	4.681152	1.55E-08
DUT	9.505843	12.94753	3.441685	1.56E-08
TMPRSS6	61.31535	37.78939	-23.526	1.56E-08
SEC24A	7.956433	6.015562	-1.94087	1.56E-08
HSP90B1	196.9224	250.6656	53.74326	1.57E-08
PAK1	4.779766	7.245099	2.465333	1.58E-08
IFI35	21.14103	31.39836	10.25733	1.58E-08
TDO2	46.7434	24.08253	-22.6609	1.58E-08
B3GNT3	4.214683	16.20986	11.99518	1.59E-08
AC010719	1.573837	2.650327	1.07649	1.60E-08
SMARCD3	1.000833	2.753822	1.752989	1.61E-08
RPS2P5	21.44651	32.82169	11.37518	1.62E-08
DGCR2	17.59942	24.35319	6.753771	1.63E-08
ILDR1	0.556089	1.880227	1.324138	1.63E-08
UNG	14.78563	19.36673	4.581107	1.64E-08
APH1A	58.51101	73.74876	15.23775	1.66E-08
EPCAM	15.79894	54.83636	39.03742	1.67E-08
SNHG9	6.618776	12.84358	6.224809	1.67E-08
CUX2	6.937685	3.383738	-3.55395	1.68E-08
RAB20	15.63061	25.96745	10.33684	1.69E-08
SDHAF4	4.754933	6.108363	1.35343	1.74E-08
C1orf162	2.032947	3.843359	1.810412	1.74E-08
IFT43	4.463536	5.625903	1.162367	1.75E-08
HP	1612.707	851.6356	-761.072	1.75E-08
DFFA	4.087949	5.308779	1.220831	1.76E-08
YJU2	11.23432	14.34853	3.114212	1.78E-08
HLA-DRB1	129.1926	278.2135	149.0209	1.79E-08
TMEM54	4.20289	17.06048	12.85759	1.81E-08
AL645608.	0.237796	1.389545	1.151749	1.81E-08
HSCB	7.262102	9.733025	2.470923	1.83E-08
GTF3A	28.95825	38.25454	9.296295	1.84E-08
LAPTM5	10.1584	20.84013	10.68173	1.84E-08
AC103810	1.232972	2.353221	1.120248	1.85E-08
NAT2	6.987013	2.827865	-4.15915	1.86E-08
ETFRF1	11.87444	9.053877	-2.82056	1.87E-08
CFHR2	138.5471	87.85096	-50.6961	1.88E-08
GLIS3	0.685301	2.026538	1.341237	1.88E-08
PPM1H	1.396043	2.96299	1.566947	1.89E-08
PIAS3	2.097291	3.385541	1.28825	1.90E-08
DHRS7B	3.9738	5.472591	1.49879	1.92E-08
NUDT19	4.233058	5.87791	1.644852	1.92E-08
EEF1D	5.942689	8.11809	2.175401	1.94E-08
NEO1	3.417727	6.941733	3.524006	1.95E-08

RTL8B	1.2	2.968916	1.768916	1.95E-08
PAQR6	0.819058	1.944754	1.125696	1.96E-08
DHX15	6.854533	9.344279	2.489745	1.97E-08
RNF121	6.009531	7.790582	1.781051	1.97E-08
AC009779	2.983563	4.114767	1.131204	1.98E-08
UBA3	4.53099	5.647428	1.116438	1.98E-08
EPB41L2	2.556135	4.116227	1.560092	1.98E-08
SERPINA1	121.8695	59.36048	-62.509	2.03E-08
DERL2	5.575264	7.00147	1.426207	2.07E-08
RNF4	5.131553	6.809596	1.678042	2.07E-08
GYPC	3.540145	7.903168	4.363023	2.09E-08
TMEM14B	7.646387	10.15063	2.504248	2.09E-08
AHSA1	23.62182	29.91255	6.290725	2.10E-08
FAF2	8.247034	10.24463	1.9976	2.11E-08
NDN	2.759629	8.717307	5.957678	2.12E-08
IL2RG	1.377819	5.261763	3.883944	2.14E-08
MSL1	6.703684	8.724486	2.020802	2.16E-08
GSTP1	13.64281	79.14004	65.49723	2.18E-08
ATP6AP2	10.11281	12.66957	2.55676	2.18E-08
TMEM62	2.63794	3.698669	1.060729	2.21E-08
SLC30A5	4.799489	6.095858	1.296369	2.23E-08
RNF126	9.946351	12.467	2.520644	2.24E-08
CDKN2C	4.83288	8.47271	3.63983	2.26E-08
DEGS1	16.09921	22.60767	6.508454	2.28E-08
TRAF1	1.718937	3.220235	1.501298	2.29E-08
BROX	7.849418	10.6106	2.761178	2.29E-08
ARID5A	6.297353	9.271761	2.974408	2.30E-08
NUFIP2	5.85592	7.823896	1.967976	2.35E-08
BSPRY	1.268033	3.723554	2.455521	2.35E-08
AFTPH	7.056252	9.626334	2.570083	2.36E-08
UAP1L1	1.035342	3.360767	2.325425	2.36E-08
IAH1	4.998051	6.351272	1.353222	2.36E-08
DCK	3.068169	4.471719	1.40355	2.36E-08
MASP2	47.85691	29.85454	-18.0024	2.37E-08
SUMO3	23.41826	29.35904	5.94078	2.38E-08
TMEM176	612.5015	442.4634	-170.038	2.39E-08
SMARCC1	7.718067	10.28236	2.564297	2.41E-08
TMEM248	21.38945	25.91818	4.528735	2.44E-08
UGT1A1	19.91921	8.329341	-11.5899	2.44E-08
CDCA7	0.710131	2.347592	1.637462	2.45E-08
NRIP1	3.430934	2.310187	-1.12075	2.45E-08
ULK3	8.611024	11.50847	2.89745	2.45E-08
WDSUB1	3.224587	4.437575	1.212988	2.50E-08
DNASE1L3	6.842309	3.242077	-3.60023	2.50E-08
PRMT6	1.827651	2.880686	1.053035	2.51E-08
TNFRSF18	0.758768	2.701253	1.942485	2.51E-08
ZNF830	4.407064	5.763701	1.356637	2.52E-08
MED16	13.03827	16.49167	3.4534	2.52E-08
PLPP3	17.03396	11.84204	-5.19191	2.53E-08
MDM4	2.284815	3.520852	1.236037	2.53E-08
DYNC1H1	3.622687	5.122897	1.500209	2.54E-08
FUT4	0.659598	1.828127	1.16853	2.55E-08
NCBP1	4.328814	5.490673	1.16186	2.55E-08
CAPN1	17.2537	24.879	7.625297	2.61E-08
TAF13	13.49411	17.13154	3.637425	2.61E-08
CDCA7L	1.025958	2.2469	1.220943	2.62E-08
TBC1D8	5.209561	8.887506	3.677945	2.66E-08
TPM2	8.954208	27.98313	19.02892	2.67E-08

MPP6	1.807703	3.285928	1.478225	2.67E-08
STK40	7.988098	10.96964	2.981542	2.68E-08
ANAPC16	17.18766	20.80414	3.616474	2.68E-08
PRTFDC1	0.913568	2.094152	1.180583	2.70E-08
FAM89A	2.461402	4.43665	1.975248	2.70E-08
GUK1	40.14118	52.24829	12.10711	2.72E-08
COMMD1	8.004291	9.896705	1.892415	2.74E-08
ITGAV	7.563008	14.99533	7.43232	2.75E-08
PAQR5	1.252464	3.628367	2.375903	2.75E-08
RETREG3	16.93402	21.05563	4.121616	2.76E-08
GPRIN2	0.117211	2.148233	2.031022	2.78E-08
SLC43A2	1.663746	2.876495	1.212749	2.79E-08
PNISR	3.076777	4.479998	1.403221	2.79E-08
GAS2L1	4.710617	6.748094	2.037476	2.82E-08
DYNC1I2	5.501565	8.558667	3.057103	2.83E-08
COA4	27.53913	33.4812	5.942073	2.84E-08
RNU6-8	2.146466	5.053264	2.906799	2.86E-08
LEAP2	91.11427	54.55714	-36.5571	2.87E-08
AGXT2	11.57268	6.323202	-5.24948	2.88E-08
ISOC1	27.27118	21.32477	-5.94642	2.88E-08
CHCHD6	2.45528	4.019873	1.564593	2.89E-08
SNHG5	3.971925	7.041827	3.069902	2.90E-08
ZNF185	0.651952	2.346783	1.694831	2.91E-08
PRXL2A	31.95089	23.72666	-8.22423	2.93E-08
PLSCR1	5.893408	10.55497	4.661558	2.95E-08
RTL6	1.356021	2.750835	1.394814	2.95E-08
NSRP1	3.842274	5.064158	1.221884	2.98E-08
TMX2	29.30855	35.36761	6.059062	2.99E-08
ALDH3B1	1.838802	4.611905	2.773103	2.99E-08
SEPTIN7	3.864836	5.062737	1.197901	2.99E-08
MFGE8	3.579775	6.643217	3.063441	3.00E-08
AC243919	83.97375	174.6139	90.64013	3.00E-08
ATP2B2	9.222181	5.93773	-3.28445	3.02E-08
PSMD10	11.57065	15.19542	3.62477	3.03E-08
KIF5B	13.02878	18.07506	5.046282	3.04E-08
YIF1B	9.33876	12.47185	3.133093	3.05E-08
DARS1	10.30628	13.13812	2.831837	3.06E-08
ARMCX3	4.968733	8.792175	3.823442	3.07E-08
STAM	2.89155	3.9156	1.02405	3.08E-08
TBCC	10.63981	13.85748	3.217674	3.08E-08
CST3	75.4925	107.1519	31.6594	3.10E-08
CPEB4	6.458657	4.445155	-2.0135	3.11E-08
BCL2L1	24.3652	39.34619	14.98099	3.17E-08
CARD11	0.583911	2.977417	2.393506	3.19E-08
CD2AP	6.444081	9.257593	2.813511	3.21E-08
DECR1	46.3673	34.21447	-12.1528	3.22E-08
PBXIP1	31.59872	44.68771	13.089	3.24E-08
SLC35A1	3.34452	4.413962	1.069442	3.24E-08
AL121832	2.094436	3.650797	1.55636	3.25E-08
QSOX1	8.070918	28.40927	20.33836	3.25E-08
AC008608	3.091369	4.833923	1.742553	3.26E-08
NUGGC	5.043507	2.441585	-2.60192	3.30E-08
CYP7B1	5.774358	3.354644	-2.41971	3.33E-08
MICOS13	31.63708	42.96786	11.33078	3.37E-08
ARHGEF17	1.587045	2.944567	1.357522	3.38E-08
TAF9	12.71566	16.86732	4.151662	3.39E-08
AGFG1	5.005445	6.610834	1.605389	3.43E-08
PYGB	16.42931	31.23077	14.80147	3.46E-08

SLC22A25	3.686878	1.962091	-1.72479	3.46E-08
TMED4	20.34322	24.68619	4.342969	3.48E-08
GRIPAP1	6.152161	7.575834	1.423673	3.49E-08
ARGLU1	6.641087	10.37004	3.728957	3.50E-08
GSR	21.29028	32.17855	10.88827	3.51E-08
ABITRAM	3.942459	5.150842	1.208383	3.51E-08
KRTCAP3	3.806842	12.93089	9.124049	3.51E-08
MBNL1	6.451911	9.019132	2.567221	3.53E-08
NAPG	3.737773	5.056832	1.319059	3.54E-08
LSM14B	9.348752	13.02511	3.676358	3.57E-08
PHF23	9.415554	12.00447	2.588921	3.60E-08
THUMPD1	3.952183	5.223674	1.271491	3.62E-08
NDUFAB1	33.38431	42.3474	8.963092	3.67E-08
CUL4B	6.53108	8.574597	2.043517	3.68E-08
JOSD1	10.17557	13.81544	3.639869	3.72E-08
COPG1	41.92733	51.01031	9.082981	3.75E-08
CASC3	9.106293	11.61528	2.508987	3.77E-08
SLC7A2	28.76278	13.66068	-15.1021	3.78E-08
PMEPA1	2.483662	20.25831	17.77465	3.80E-08
RNASE6	5.524791	11.0584	5.533608	3.82E-08
GGNBP2	5.386049	6.581629	1.19558	3.86E-08
EOLA2	4.625456	5.821429	1.195973	3.88E-08
ADAP1	0.430321	1.679374	1.249053	3.92E-08
KIAA0319I	8.119816	10.76866	2.648843	3.93E-08
SFN	15.48538	42.73623	27.25085	3.96E-08
YTHDC1	5.538426	7.215025	1.676599	3.98E-08
APOA5	136.9958	73.83368	-63.1621	3.99E-08
ARL4C	4.159759	16.44537	12.28561	4.04E-08
FAM20A	14.61296	10.6051	-4.00786	4.07E-08
RCL1	13.10419	8.274673	-4.82951	4.12E-08
SHARPIN	26.82277	36.24884	9.426071	4.12E-08
MDFI	0.916687	4.39203	3.475342	4.13E-08
LBH	5.125582	10.80464	5.679054	4.13E-08
PELI1	3.832408	6.307945	2.475536	4.14E-08
TMEM156	1.498623	5.279212	3.780589	4.16E-08
HSD17B13	70.18661	20.58903	-49.5976	4.19E-08
AGRN	12.01869	25.72483	13.70614	4.20E-08
GPR35	0.78222	2.296345	1.514125	4.21E-08
BHLHE41	0.643093	2.704889	2.061796	4.22E-08
GCGR	14.40558	6.190204	-8.21538	4.22E-08
MFN1	3.557622	4.675633	1.118011	4.23E-08
EFNA5	0.17779	1.886867	1.709077	4.26E-08
PFKP	1.449017	6.562248	5.113232	4.29E-08
UBFD1	5.06003	6.754665	1.694635	4.29E-08
DANCR	1.605796	2.659154	1.053358	4.34E-08
SF3B1	15.78782	20.41591	4.628091	4.37E-08
PTGES3P1	4.701359	7.019732	2.318373	4.38E-08
BIN1	7.540723	11.39283	3.85211	4.40E-08
CASTOR2	1.47321	2.528779	1.055569	4.41E-08
PLEKHA4	4.857946	7.766872	2.908926	4.44E-08
PGLYRP2	57.77436	28.11461	-29.6597	4.45E-08
MRPL58	24.64861	31.14327	6.494668	4.48E-08
CHMP7	5.363774	7.521677	2.157903	4.49E-08
WWTR1	4.026844	9.477337	5.450494	4.55E-08
NLRP6	1.752377	0.673163	-1.07921	4.57E-08
CMTM4	1.512822	3.191515	1.678694	4.57E-08
AGPAT2	57.26631	99.37643	42.11012	4.60E-08
TUBA1A	6.493433	18.2363	11.74287	4.60E-08

CREB3L4	4.645867	6.525965	1.880098	4.60E-08
HLA-DQB	1.759729	6.106942	4.347213	4.60E-08
PLD3	32.5562	43.15069	10.59449	4.63E-08
SMARCA5	5.534128	7.542305	2.008177	4.63E-08
POGZ	5.496949	8.343021	2.846072	4.64E-08
GPS1	19.71216	25.17046	5.458301	4.69E-08
TCF19	4.454239	7.802149	3.34791	4.70E-08
MICALL1	3.347209	4.832998	1.485789	4.73E-08
TOMM40	15.02588	26.67599	11.65011	4.76E-08
ALOX5	1.470967	5.756233	4.285266	4.76E-08
ACP1	18.08388	22.20806	4.124183	4.77E-08
SH3RF1	2.145176	4.942252	2.797076	4.77E-08
MAD2L2	8.600455	12.2165	3.61605	4.77E-08
EIF3FP3	2.887265	4.064536	1.177271	4.78E-08
FGD6	1.2103	2.868352	1.658052	4.79E-08
NUDT15	5.38036	7.053195	1.672835	4.80E-08
PSMC3	56.4741	69.29324	12.81913	4.84E-08
CD86	1.114219	2.145517	1.031298	4.85E-08
SLC41A1	2.003906	3.897242	1.893336	4.87E-08
TTYH3	10.99691	19.67939	8.682482	4.90E-08
VAC14	4.783519	6.101583	1.318065	4.90E-08
PAK1IP1	9.434081	12.06307	2.628988	4.91E-08
WAC	6.770843	8.758323	1.98748	4.92E-08
RPS20P14	2.113585	4.160933	2.047348	4.94E-08
CCDC198	2.97361	5.299202	2.325592	5.02E-08
HSD17B8	39.48771	27.41078	-12.0769	5.03E-08
STXBP3	4.139406	5.838284	1.698878	5.07E-08
AL023284.	4.095352	6.497815	2.402463	5.07E-08
NCOA6	3.102151	4.131962	1.029811	5.16E-08
RPN1	91.64531	108.2642	16.61886	5.18E-08
AC022400	2.081586	3.748792	1.667205	5.19E-08
SPIN1	7.401084	10.95752	3.55644	5.22E-08
APOO	4.868404	6.534176	1.665772	5.23E-08
HP1BP3	8.264525	10.71622	2.451698	5.27E-08
GPC4	1.113029	3.838006	2.724978	5.30E-08
RHOB	248.7249	169.3532	-79.3718	5.32E-08
KCNK5	2.862209	10.32235	7.460143	5.34E-08
PLEKHB1	0.597082	5.852982	5.2559	5.35E-08
THRAP3	18.55086	22.78235	4.231494	5.36E-08
CORO1B	15.67147	20.07997	4.408504	5.37E-08
THOC2	3.011593	4.013949	1.002356	5.44E-08
MLF2	61.54109	73.24297	11.70188	5.50E-08
SLC9A3R1	98.62434	135.2495	36.6252	5.56E-08
OPTN	22.8192	29.31174	6.492536	5.58E-08
PPP6C	9.083833	10.86049	1.776659	5.68E-08
AC026803	9.046923	4.980056	-4.06687	5.73E-08
SMIM4	4.820933	7.502569	2.681636	5.74E-08
DLGAP1-/	2.609495	4.092315	1.48282	5.77E-08
AC021074	5.528004	2.418386	-3.10962	5.77E-08
PLBD1	1.748348	6.332188	4.583839	5.78E-08
PKP4	5.718395	8.150847	2.432452	5.89E-08
MYOF	1.660252	5.702589	4.042337	5.90E-08
KRT17P8	10.13209	5.115066	-5.01703	5.93E-08
ATP5MC1	36.478	52.55797	16.07997	5.95E-08
MAU2	2.886525	4.180009	1.293484	6.00E-08
C16orf70	5.529372	4.09305	-1.43632	6.00E-08
FAXDC2	13.8411	8.727766	-5.11334	6.02E-08
BHMT	105.8601	44.41721	-61.4429	6.04E-08

MIR4653	0.796398	1.909307	1.112909	6.04E-08
WNK2	0.696059	4.427368	3.731309	6.05E-08
DSG1	2.061777	0.811347	-1.25043	6.08E-08
NDUFB8	1.990247	3.511799	1.521552	6.22E-08
MIR3685	2.62936	6.118508	3.489148	6.32E-08
HLA-DMB	2.620581	5.520548	2.899967	6.38E-08
RBBP4	4.774162	6.660769	1.886608	6.42E-08
JAG1	3.466108	8.732732	5.266624	6.45E-08
RAB31	2.307214	4.53708	2.229866	6.46E-08
RAB36	0.193962	1.736645	1.542683	6.50E-08
ECHS1	440.5926	328.8412	-111.751	6.51E-08
CTTN	19.37048	28.15359	8.78311	6.52E-08
UPF3A	3.081813	4.221701	1.139888	6.57E-08
CBX4	11.67517	16.40897	4.733794	6.59E-08
ABCC2	24.82293	16.08016	-8.74277	6.59E-08
UNC13D	0.910298	6.70342	5.793122	6.61E-08
GTF2E2	6.662692	9.146962	2.48427	6.63E-08
TMEM267	2.591343	4.217213	1.62587	6.70E-08
FOXJ1	0.363415	4.351741	3.988326	6.76E-08
SFT2D2	4.846105	6.845406	1.9993	6.78E-08
WWC3	2.418216	3.605969	1.187753	6.82E-08
SPATA2L	5.927107	8.126827	2.19972	6.84E-08
WDR1	21.43649	28.22884	6.792358	6.86E-08
ZNF385B	2.325007	1.052194	-1.27281	6.87E-08
AC006978	3.873808	5.001903	1.128096	6.88E-08
ZNF76	4.333649	5.499917	1.166268	6.91E-08
IDUA	3.706377	5.477872	1.771495	6.94E-08
MEA1	15.67635	19.90295	4.226594	6.94E-08
RPE	5.237223	6.806158	1.568934	6.96E-08
DYSF	6.337846	4.507649	-1.8302	7.04E-08
CHST11	1.452718	2.984893	1.532175	7.05E-08
CP	108.5771	62.99278	-45.5844	7.06E-08
CSK	11.58435	15.73527	4.150922	7.09E-08
DCTN6	4.321825	5.757699	1.435874	7.09E-08
NSUN6	7.064607	5.345086	-1.71952	7.13E-08
CHML	1.610847	2.911393	1.300546	7.16E-08
ASH2L	4.049179	5.433698	1.384519	7.17E-08
GCNT3	0.566705	3.368067	2.801362	7.24E-08
PIGK	4.084817	5.325975	1.241158	7.39E-08
MOGS	19.61761	23.45539	3.837773	7.40E-08
BX322562	1.581707	3.634838	2.053132	7.40E-08
AC092718	6.163425	8.882112	2.718687	7.42E-08
AK2	10.78079	13.03408	2.25329	7.46E-08
GAPDH	380.25	531.1675	150.9175	7.48E-08
TMEM263	7.837983	10.18701	2.349023	7.51E-08
KLF9	24.12825	15.50411	-8.62414	7.53E-08
TNFAIP2	10.34056	22.02703	11.68647	7.63E-08
MCM2	2.498246	4.67765	2.179404	7.64E-08
NHP2	16.29296	20.75401	4.461048	7.64E-08
ENAH	4.703986	8.261176	3.55719	7.73E-08
STK35	3.553834	4.803993	1.250159	7.74E-08
DDX1	7.748031	9.501668	1.753637	7.84E-08
SPI1	4.76361	9.255073	4.491463	7.85E-08
SDCCAG8	2.195867	3.537426	1.341559	7.86E-08
RPL41P1	120.15	181.5838	61.43383	7.88E-08
SLC39A7	65.35556	85.0154	19.65984	7.93E-08
FRAS1	0.363545	2.006176	1.642631	7.97E-08
ITIH3	320.3396	208.8071	-111.533	8.02E-08

PDCD11	4.932403	6.539765	1.607362	8.02E-08
GLT1D1	7.587074	4.598495	-2.98858	8.05E-08
AC079922	3.076667	6.584051	3.507384	8.12E-08
ZBTB5	2.853586	3.898159	1.044573	8.15E-08
BAIAP2-D	4.435092	6.863949	2.428857	8.17E-08
ERO1A	7.910713	11.8304	3.919686	8.20E-08
ILF3-DT	5.760439	8.373082	2.612643	8.21E-08
RASSF8	0.91861	2.507993	1.589383	8.24E-08
COL9A2	0.437601	3.973798	3.536196	8.26E-08
PCTP	11.60308	8.698665	-2.90442	8.32E-08
SEL1L3	3.843105	13.74566	9.902551	8.34E-08
SSR1	14.67747	18.50012	3.822649	8.37E-08
TPRA1	6.861132	8.090652	1.22952	8.39E-08
RPL10P16	2.375547	3.709806	1.33426	8.42E-08
PPP1R15A	10.7634	21.11513	10.35173	8.42E-08
MITF	0.438013	1.782208	1.344195	8.47E-08
KIF3C	0.412728	2.188874	1.776146	8.51E-08
SMC5	3.186663	4.401629	1.214967	8.61E-08
RENBP	3.712895	11.63775	7.924857	8.61E-08
IL4I1	0.966893	3.307407	2.340514	8.63E-08
KIAA0100	5.919074	7.727632	1.808557	8.64E-08
SLCO3A1	1.074062	2.270555	1.196493	8.70E-08
TTLL4	2.55474	5.033683	2.478942	8.70E-08
TIMELESS	5.855702	8.159598	2.303896	8.75E-08
CKAP5	7.355363	9.694147	2.338784	8.79E-08
RNMT	3.239574	4.300687	1.061113	8.81E-08
HEXIM1	8.886013	12.39113	3.505116	8.83E-08
MPPED1	2.175633	0.962894	-1.21274	8.87E-08
INAVA	1.198476	3.702855	2.504379	8.88E-08
XPR1	2.810567	4.124149	1.313583	8.89E-08
CHCHD5	5.0417	6.60125	1.55955	8.89E-08
AC010503	13.87571	22.69438	8.818669	8.92E-08
VAV3	0.541749	2.069895	1.528146	9.01E-08
RAB6B	0.462355	1.633558	1.171203	9.03E-08
VEPH1	0.234327	1.751202	1.516875	9.14E-08
SMIM10	0.809839	1.866459	1.05662	9.38E-08
RPP25	2.454535	5.398061	2.943525	9.39E-08
GCDH	18.11054	12.3182	-5.79235	9.46E-08
XPO7	4.34546	7.098337	2.752877	9.53E-08
AL355102.	1.186201	4.39595	3.209749	9.55E-08
LINC0148!	11.64681	6.130381	-5.51643	9.59E-08
TTI1	5.162742	6.491659	1.328917	9.61E-08
AC073861	5.732001	9.878881	4.14688	9.62E-08
SLC2A1	0.931749	4.22734	3.295591	9.67E-08
PTPRA	9.483374	12.12683	2.643457	9.67E-08
BTBD3	2.614563	4.210845	1.596282	9.70E-08
AP1S1	28.42923	36.49339	8.064165	9.74E-08
ATG4D	3.715622	5.339885	1.624263	9.80E-08
WIPI2	8.079429	9.941596	1.862167	9.83E-08
RNF152	3.542835	2.398283	-1.14455	9.83E-08
MEIS2	1.217256	2.894182	1.676926	9.91E-08
DHX30	8.593023	10.20332	1.610294	9.99E-08
BAMBI	14.35462	27.19954	12.84493	1.00E-07
ATF7IP	2.83114	3.9707	1.13956	1.00E-07
CNPY3	37.02876	52.52196	15.49321	1.01E-07
TAMALIN	1.797743	2.849706	1.051963	1.01E-07
SKIL	3.600699	5.350943	1.750244	1.03E-07
SLC26A6	3.117979	4.714568	1.59659	1.04E-07

RNF216	4.679778	5.928814	1.249036	1.04E-07
ARF1	142.5047	175.1302	32.6255	1.05E-07
GPX7	3.464743	6.586924	3.122181	1.05E-07
PID1	14.57018	9.870385	-4.6998	1.05E-07
ELOVL7	1.513362	6.039905	4.526543	1.06E-07
AKR1C6P	5.955833	3.190772	-2.76506	1.06E-07
POLD2	27.42068	35.70582	8.285139	1.07E-07
MMP14	15.07803	46.24714	31.16911	1.09E-07
JAG2	1.743641	3.091532	1.347891	1.10E-07
MAP3K21	0.974271	2.611252	1.63698	1.10E-07
PPP2R1B	17.8404	12.08117	-5.75923	1.11E-07
BACE2	2.372637	10.45542	8.082783	1.12E-07
ENTPD4	2.458088	3.619509	1.161421	1.12E-07
CAND1	4.499125	5.796699	1.297574	1.12E-07
WDR5	11.68953	15.04125	3.351714	1.13E-07
MYO1C	17.05506	25.76792	8.712861	1.14E-07
MT-ND5	2036.221	1300.9	-735.321	1.14E-07
GGA2	6.794074	9.097615	2.303541	1.15E-07
MRPL55	29.44519	42.01094	12.56575	1.17E-07
LINC0134	5.41206	2.629719	-2.78234	1.17E-07
CDC42SE1	9.653907	14.73936	5.085454	1.18E-07
MED21	3.416585	4.492101	1.075516	1.18E-07
PTPN6	6.037245	9.834779	3.797534	1.18E-07
INF2	7.04553	10.0391	2.993573	1.19E-07
ACVR1	5.166609	8.111516	2.944907	1.19E-07
ZNF146	11.11036	15.04348	3.933121	1.19E-07
SLC16A13	16.6502	10.54643	-6.10378	1.20E-07
ZMAT2	41.7188	51.4333	9.714504	1.20E-07
ICA1	2.473328	4.239201	1.765873	1.21E-07
LGALSL	3.286189	4.453213	1.167024	1.22E-07
FGFR4	38.42847	56.63153	18.20307	1.22E-07
DUSP23	92.50405	134.8932	42.38911	1.23E-07
GPX8	0.921213	2.588474	1.667261	1.23E-07
TMEM164	2.271413	3.448876	1.177464	1.24E-07
LAMC2	0.459695	12.02884	11.56915	1.24E-07
POLR2J	26.19028	42.38264	16.19236	1.25E-07
ADORA2B	2.856741	1.105468	-1.75127	1.25E-07
N4BP2L1	4.081428	2.992207	-1.08922	1.26E-07
NDUFS8	18.35934	25.78456	7.425212	1.26E-07
DNASE2	21.37269	27.64138	6.268691	1.26E-07
FKBP3	11.21227	13.75452	2.542249	1.27E-07
LSM8	1.206612	2.2323	1.025688	1.28E-07
ANO10	4.481118	6.150101	1.668984	1.28E-07
FAIM	1.861938	3.167232	1.305293	1.28E-07
H6PD	26.53013	18.97811	-7.55202	1.29E-07
FZD1	0.954084	3.193503	2.239419	1.29E-07
MAOA	32.63623	22.62877	-10.0075	1.30E-07
ADAM8	1.047504	3.042086	1.994582	1.31E-07
SLC66A3	4.636698	7.351219	2.714521	1.32E-07
VASN	15.32137	22.52142	7.200053	1.33E-07
PTPN1	8.54257	10.85726	2.314693	1.33E-07
SINHCAF	1.534357	3.704367	2.170009	1.33E-07
SLC26A1	5.640352	3.506136	-2.13422	1.34E-07
C9orf16	17.05446	29.89616	12.8417	1.35E-07
ANO1	12.27862	5.503312	-6.77531	1.35E-07
PM20D2	2.333429	3.556972	1.223543	1.36E-07
SNHG8	4.783185	7.142335	2.359151	1.36E-07
TINAGL1	3.989281	9.337383	5.348102	1.36E-07

CCNO	0.757826	1.822424	1.064599	1.36E-07
ITPKA	3.111095	5.88306	2.771965	1.38E-07
RAB25	2.604134	18.83351	16.22938	1.38E-07
METAP2	13.44759	16.57029	3.122698	1.38E-07
POLR1E	6.377488	8.078896	1.701408	1.38E-07
MAP1LC3I	8.96114	12.1389	3.177759	1.39E-07
MOB3B	1.181043	2.495161	1.314118	1.40E-07
DCDC2	5.882735	20.82781	14.94508	1.42E-07
RELA	13.07844	15.45775	2.379312	1.42E-07
HLA-DMA	15.7998	32.81738	17.01758	1.46E-07
PEMT	22.60815	14.35751	-8.25064	1.46E-07
LINC0187	1.187785	2.604416	1.416632	1.47E-07
NPLOC4	12.82849	16.04983	3.221339	1.47E-07
MTFR1	10.08421	7.712343	-2.37187	1.48E-07
LIF	1.116249	6.155987	5.039738	1.48E-07
CRACR2B	1.231236	3.412543	2.181306	1.48E-07
FZD7	0.677751	2.259348	1.581597	1.49E-07
ITGA1	6.665907	5.256599	-1.40931	1.50E-07
BICDL2	0.209714	1.927137	1.717423	1.51E-07
MRPL30	6.309969	7.709789	1.39982	1.51E-07
ABL1	5.82381	8.086585	2.262775	1.52E-07
STX1A	0.364737	1.38661	1.021873	1.52E-07
MAEA	4.139314	5.417288	1.277973	1.54E-07
LRRC59	22.70038	29.6296	6.929227	1.54E-07
FKBP4	16.69835	22.40593	5.707583	1.54E-07
ETFA	40.37983	31.8782	-8.50163	1.55E-07
MTPN	19.79573	25.92252	6.126795	1.55E-07
YIPF5	9.10061	11.34404	2.24343	1.56E-07
C1orf116	0.580059	4.407988	3.827929	1.57E-07
RASEF	1.291033	2.846484	1.555451	1.58E-07
TPRG1-AS	4.97099	2.139619	-2.83137	1.60E-07
ATG3	5.313084	6.446264	1.133179	1.60E-07
TCN2	6.298359	10.51027	4.211915	1.60E-07
NPDC1	3.695891	7.32383	3.627939	1.61E-07
FGB	1732.683	1082.446	-650.236	1.61E-07
CNIH1	22.96138	18.83052	-4.13086	1.61E-07
GFRA1	4.693719	2.264043	-2.42968	1.62E-07
RPS6KA1	3.647024	5.482962	1.835937	1.62E-07
ENGASE	3.381337	4.560869	1.179532	1.63E-07
AC025181	2.706469	3.924252	1.217783	1.64E-07
UCK1	12.80103	16.54941	3.748383	1.64E-07
AL356356.	0.612201	1.749484	1.137283	1.65E-07
DOK2	2.388534	4.50611	2.117575	1.65E-07
SKIV2L	11.10879	13.85606	2.747271	1.69E-07
MTG2	7.79904	9.560207	1.761167	1.70E-07
CSF1	4.876933	10.09695	5.220021	1.70E-07
RPS23	20.62259	28.95599	8.333402	1.71E-07
NPC2	23.39776	34.48716	11.0894	1.71E-07
PDK2	6.543835	4.732211	-1.81162	1.71E-07
CEP20	10.56257	13.03287	2.470304	1.72E-07
FJX1	1.184894	3.629375	2.444481	1.72E-07
BLOC1S5	4.016831	5.226562	1.209731	1.74E-07
EPS8L2	18.85079	26.30276	7.451968	1.76E-07
SIRPA	12.81207	18.6397	5.82763	1.79E-07
PEX11A	7.641468	5.606018	-2.03545	1.79E-07
NIPA1	1.948522	3.617775	1.669254	1.81E-07
ABCF3	10.20632	11.78583	1.579512	1.82E-07
QRICH1	7.724825	9.248868	1.524043	1.82E-07

CAVIN1	20.98373	37.57633	16.5926	1.85E-07
LETMD1	7.216751	8.928896	1.712144	1.85E-07
SLC22A10	8.102252	3.545153	-4.5571	1.86E-07
FAAP100	7.590353	9.610764	2.020411	1.86E-07
SSH3	6.161792	9.759114	3.597322	1.86E-07
TM6SF2	4.608213	2.430477	-2.17774	1.87E-07
HSPA4	23.55218	29.02435	5.472178	1.89E-07
RGL3	3.630171	6.127554	2.497383	1.90E-07
FCGBP	0.49835	2.684182	2.185832	1.90E-07
ABI1	9.074705	12.001	2.926294	1.90E-07
ZSWIM7	4.381113	5.682193	1.301079	1.90E-07
TP53I11	3.790956	5.935097	2.144141	1.91E-07
LEPR	18.48209	8.004291	-10.4778	1.91E-07
ASPM	1.516196	2.7518	1.235604	1.91E-07
RPP25L	23.05981	29.45741	6.397598	1.92E-07
CA5A	8.947904	5.441288	-3.50662	1.93E-07
MYO10	0.865186	3.063863	2.198677	1.96E-07
PLLP	1.38268	3.166332	1.783652	1.97E-07
LBR	13.94644	19.14764	5.201195	1.98E-07
RAI1	1.420037	2.569053	1.149016	1.98E-07
VAT1	39.36776	59.85843	20.49067	1.99E-07
KLF5	2.157471	7.394082	5.236611	1.99E-07
NBPF13P	5.603684	2.623307	-2.98038	2.00E-07
FOLH1	2.827071	1.681214	-1.14586	2.00E-07
ARHGAP4	3.834266	8.323046	4.48878	2.02E-07
TRIM56	4.304601	5.849728	1.545127	2.03E-07
PIGZ	1.373358	2.462549	1.089191	2.04E-07
SLC41A2	10.93893	7.712679	-3.22625	2.04E-07
INCENP	1.994219	3.007672	1.013453	2.05E-07
C1QA	64.79176	137.8314	73.03965	2.07E-07
MYBBP1A	5.964208	8.02021	2.056002	2.07E-07
GNA11	6.172908	7.462178	1.289269	2.07E-07
NR1H4	20.23498	15.89581	-4.33917	2.08E-07
WDR18	15.58477	20.08066	4.495889	2.08E-07
UGT1A3	1.878117	0.580149	-1.29797	2.08E-07
GAPDHP1	5.950676	13.16301	7.212331	2.08E-07
SLC30A7	3.458358	4.54825	1.089892	2.09E-07
GLIPR2	2.098944	4.39073	2.291786	2.09E-07
UGT1A2P	4.497586	1.20362	-3.29397	2.09E-07
PGAM5	10.04336	12.60993	2.566571	2.11E-07
B4GALNT4	0.521193	4.079805	3.558613	2.11E-07
HOXB7	0.541969	2.895753	2.353783	2.12E-07
GATAD2A	7.215679	9.292214	2.076534	2.14E-07
PRKCSH	68.34004	81.429	13.08896	2.16E-07
AC144530	2.638851	3.825777	1.186927	2.17E-07
C19orf54	2.720263	4.473775	1.753513	2.18E-07
ITGB5	24.06356	34.89652	10.83296	2.18E-07
SLC30A1	21.87569	14.17016	-7.70553	2.19E-07
HSDL1	2.855498	3.978928	1.123431	2.19E-07
COX5A	75.96853	96.90702	20.93849	2.23E-07
HLF	18.22859	9.064938	-9.16365	2.23E-07
PJA1	4.010433	6.974175	2.963742	2.25E-07
SMG7	9.308992	12.06378	2.754786	2.25E-07
TMEM128	5.770635	7.48987	1.719235	2.26E-07
MAPK1IP1	7.779976	9.616343	1.836367	2.26E-07
HDAC6	8.805637	6.649815	-2.15582	2.27E-07
AC090498	98.58624	146.7953	48.20908	2.28E-07
PCED1B	1.383216	2.91778	1.534564	2.28E-07

NFIB	3.486781	4.973751	1.48697	2.28E-07
TAP1	8.655053	17.30078	8.645732	2.28E-07
SNORD6	1.414307	2.734178	1.31987	2.30E-07
TRIM21	8.513729	11.06601	2.552283	2.31E-07
TMEM109	35.76897	49.2348	13.46583	2.32E-07
LARP4	9.672577	7.958087	-1.71449	2.32E-07
TM2D2	5.998067	7.865405	1.867339	2.33E-07
ICE1	2.742588	3.847196	1.104608	2.35E-07
SETD7	6.875448	5.202815	-1.67263	2.37E-07
MIR570	3.227874	5.402997	2.175123	2.38E-07
PLOD3	22.45752	29.59122	7.133698	2.38E-07
ALDOB	1139.314	597.3651	-541.949	2.38E-07
SNRNP20(	14.53851	18.88016	4.341649	2.41E-07
PCCA	7.174199	5.174263	-1.99994	2.41E-07
BEX4	5.239107	9.938616	4.699508	2.42E-07
MANF	39.99118	54.93924	14.94805	2.43E-07
KLB	14.18977	9.130736	-5.05904	2.43E-07
C6orf136	9.702254	12.71218	3.009924	2.44E-07
LAMC1	16.99459	29.65365	12.65906	2.44E-07
SYNGR1	1.845087	3.697999	1.852912	2.47E-07
RPRD1A	4.122767	6.119163	1.996396	2.49E-07
AC009166	2.483235	1.291776	-1.19146	2.49E-07
INTS10	4.775096	6.248059	1.472963	2.52E-07
S100A14	16.98278	55.94076	38.95798	2.53E-07
CRAT	39.72443	31.70113	-8.0233	2.57E-07
SLU7	7.099035	8.578498	1.479463	2.57E-07
GJB3	0.172075	2.133089	1.961014	2.58E-07
USH1C	2.436144	9.664303	7.228159	2.59E-07
MALSU1	7.175734	8.921033	1.7453	2.59E-07
PKIB	1.336086	3.857565	2.521479	2.61E-07
NPIPB15	1.638671	2.94605	1.307379	2.62E-07
ETV7	1.248185	2.652368	1.404183	2.64E-07
PRR36	0.347869	1.555644	1.207775	2.65E-07
NOMO1	6.003938	7.472634	1.468696	2.65E-07
AP000254	2.241191	3.312192	1.071001	2.65E-07
CDC27	5.500964	6.881045	1.380081	2.65E-07
AL354836.	0.867232	2.19927	1.332039	2.67E-07
C1QTNF6	2.105019	3.649939	1.54492	2.71E-07
CNGA1	4.20994	2.158901	-2.05104	2.73E-07
IER5	4.628879	7.263375	2.634497	2.76E-07
FBXL12	4.478228	5.610577	1.132349	2.77E-07
PRKAR1B	3.520538	5.622639	2.102101	2.78E-07
PARP1	7.409596	9.547114	2.137518	2.78E-07
ACAA2	56.57308	37.20052	-19.3726	2.82E-07
SLC28A1	14.12528	7.464962	-6.66032	2.83E-07
ACBD4	15.73443	11.54885	-4.18558	2.86E-07
MRPL36	15.60209	19.36094	3.758849	2.87E-07
ETF1	16.86223	20.5594	3.697171	2.91E-07
PRSS22	0.472809	4.845321	4.372512	2.92E-07
MEX3A	0.820179	1.909515	1.089336	2.93E-07
PTPN23	8.193118	10.19639	2.003277	2.94E-07
SLC35B2	23.6569	31.78834	8.131437	2.96E-07
SERPINF2	522.451	362.4645	-159.987	2.97E-07
THEMIS2	1.874978	3.473954	1.598976	2.98E-07
CPSF1	13.28707	17.64065	4.353585	2.99E-07
MED12	3.778389	5.15589	1.377501	3.01E-07
DNALI1	2.550927	4.813553	2.262626	3.02E-07
RMND5A	14.22717	11.20528	-3.02189	3.03E-07

H2BC8	0.950075	2.604468	1.654394	3.03E-07
POLDIP3	14.62909	18.90093	4.271842	3.08E-07
ZFYVE27	7.268353	9.312619	2.044266	3.08E-07
HES6	3.203575	5.605448	2.401873	3.11E-07
YBEY	6.324084	8.634677	2.310593	3.11E-07
FAM174C	24.20177	35.25484	11.05307	3.11E-07
HLA-DRB(	8.178666	18.25508	10.07642	3.13E-07
CAV1	8.210281	14.53976	6.32948	3.18E-07
CSDE1	55.45271	69.93688	14.48416	3.22E-07
RCAN1	8.286732	5.289082	-2.99765	3.23E-07
LRP5	33.79606	23.57851	-10.2175	3.24E-07
FAM210B	31.30055	24.14882	-7.15173	3.25E-07
FGD3	0.703263	1.895774	1.192511	3.29E-07
Z97056.2	3.19702	1.491698	-1.70532	3.30E-07
MAPK15	0.095755	1.455012	1.359257	3.30E-07
ELP5	9.141752	11.81904	2.677289	3.31E-07
RAB27B	0.334119	1.444115	1.109996	3.32E-07
SPATA20	10.21069	16.54034	6.329646	3.35E-07
UNC5CL	7.208299	13.86621	6.657913	3.35E-07
SLC2A10	10.55754	6.875005	-3.68253	3.36E-07
ELOVL2	30.73172	17.75422	-12.9775	3.36E-07
PPP1R2	5.147385	6.647173	1.499788	3.38E-07
SELENBP1	83.0944	53.53956	-29.5548	3.41E-07
CCDC85B	12.02526	18.37778	6.352526	3.42E-07
SDC2	117.5363	86.28674	-31.2496	3.44E-07
C1RL	27.11952	20.95899	-6.16053	3.46E-07
LINC0183(	0.572877	2.260635	1.687758	3.51E-07
SLC35E1	4.881189	6.262459	1.381269	3.51E-07
NPAS2	1.040065	2.443514	1.403449	3.51E-07
GZMB	0.873136	2.377456	1.504319	3.56E-07
PKD2	2.242636	4.158857	1.916221	3.57E-07
IL4R	10.54164	14.41271	3.871073	3.59E-07
NLRC5	1.560953	2.730901	1.169949	3.60E-07
SH3D19	9.9378	7.261281	-2.67652	3.60E-07
WWP2	4.524404	5.632397	1.107993	3.61E-07
ATP5ME	11.34878	22.95985	11.61107	3.61E-07
C11orf54	10.60691	7.83685	-2.77006	3.72E-07
MSRB2	22.32662	30.00587	7.679252	3.74E-07
BOLA1	13.01372	16.80288	3.78916	3.77E-07
FTCD	93.67931	59.43921	-34.2401	3.77E-07
PROM1	0.244885	4.186595	3.94171	3.78E-07
CLEC2B	1.529641	3.010179	1.480538	3.84E-07
MRPL45	21.39347	27.09669	5.703224	3.85E-07
ZNF33B	2.229591	3.430007	1.200417	3.95E-07
LAG3	0.926129	2.659254	1.733125	3.99E-07
SLC6A13	2.275527	1.145113	-1.13041	3.99E-07
MAD2L1B	10.40261	12.88285	2.480245	4.00E-07
C8orf33	11.74727	15.71708	3.969807	4.03E-07
OSMR	3.447485	6.85533	3.407845	4.04E-07
ALDH3A2	23.97086	17.04497	-6.92589	4.04E-07
PARVA	3.005487	4.360912	1.355425	4.04E-07
RCOR3	2.69257	3.751986	1.059416	4.09E-07
SHB	3.047902	4.305634	1.257732	4.10E-07
SLC1A2	4.070821	1.759278	-2.31154	4.14E-07
TMC4	4.966385	19.95841	14.99203	4.19E-07
FBXL15	5.108645	6.976273	1.867628	4.22E-07
TESC	10.60936	39.70676	29.0974	4.23E-07
PKP3	0.383877	5.089829	4.705952	4.24E-07

DPP7	30.91669	47.10048	16.1838	4.29E-07
SIPA1	8.647085	11.98793	3.340847	4.30E-07
DPM1	21.59586	26.21348	4.617622	4.32E-07
RBM25	3.491364	4.540542	1.049179	4.35E-07
RINL	2.204134	3.280301	1.076167	4.37E-07
DHODH	8.314142	5.348223	-2.96592	4.40E-07
SNHG11	2.733975	4.328783	1.594808	4.41E-07
ZNF644	3.035535	4.064325	1.02879	4.41E-07
PHETA2	1.465851	3.274386	1.808534	4.42E-07
PNPLA3	6.189166	4.077215	-2.11195	4.42E-07
DNAJC4	8.118013	10.58261	2.464597	4.43E-07
ECPAS	9.463189	11.46711	2.003918	4.43E-07
VDAC3	19.77866	25.91772	6.139061	4.46E-07
KDM3B	5.809579	7.922537	2.112959	4.46E-07
COX17	20.15464	24.79044	4.635796	4.46E-07
CPNE2	2.188063	3.227866	1.039803	4.49E-07
EMB	0.841155	2.4831	1.641944	4.53E-07
CASP1	2.252057	3.557408	1.305352	4.53E-07
MYO6	3.545891	5.207795	1.661903	4.64E-07
KCNF1	0.386503	1.501954	1.115451	4.65E-07
NUDCD3	7.204798	8.730847	1.526049	4.65E-07
PUM3	5.776197	9.346941	3.570744	4.67E-07
IQANK1	0.260479	1.311164	1.050685	4.73E-07
OSTC	48.47784	58.7539	10.27607	4.77E-07
IRAK1	29.48753	40.33061	10.84308	4.77E-07
SRA1	12.71313	16.01024	3.297115	4.79E-07
SLC4A11	0.301021	1.39132	1.090299	4.82E-07
HENMT1	0.819004	2.059767	1.240763	4.87E-07
NDUFAF3	25.57332	33.05471	7.481391	4.88E-07
CHPF2	8.348981	10.861	2.512018	4.91E-07
PKHD1	0.452154	2.089422	1.637268	4.91E-07
SGF29	6.691541	8.5947	1.903158	4.96E-07
HPS6	5.430529	6.579633	1.149105	4.96E-07
EPAS1	30.94382	23.98097	-6.96285	5.00E-07
HCCS	8.359178	10.18065	1.821472	5.01E-07
MYADM	8.996089	14.16189	5.1658	5.02E-07
PLEKHH3	4.186919	5.806192	1.619272	5.03E-07
SLC9A7	1.332209	2.514446	1.182237	5.04E-07
CDCP1	0.402023	3.118809	2.716786	5.05E-07
BCAP31	90.85421	119.8267	28.97247	5.12E-07
ADSS2	7.606546	10.42291	2.816362	5.12E-07
LIMS1	4.026602	6.136796	2.110194	5.14E-07
AP1B1	14.18645	17.43498	3.248529	5.15E-07
TMEM141	13.3427	29.29621	15.9535	5.17E-07
ATP6V1G1	22.14069	27.0765	4.935805	5.20E-07
KRT80	0.668547	7.405122	6.736574	5.20E-07
VPS11	5.896279	7.234928	1.338649	5.21E-07
SH3YL1	0.973922	3.239752	2.26583	5.28E-07
VPS41	4.570404	6.435871	1.865467	5.28E-07
C19orf53	56.8237	91.50099	34.6773	5.30E-07
ST14	13.20282	27.42324	14.22042	5.33E-07
SUZ12	4.516361	5.902529	1.386168	5.33E-07
AC005332	5.276642	6.642012	1.36537	5.41E-07
PHB2	42.15465	51.24407	9.089425	5.43E-07
NDUFA1	276.8211	363.3759	86.55476	5.43E-07
TOP1MT	6.298098	8.7928	2.494702	5.44E-07
GPATCH8	2.572586	3.575684	1.003098	5.48E-07
CRY2	14.07507	10.58852	-3.48656	5.49E-07

MRPL4	7.485409	9.056756	1.571346	5.54E-07
DAB2	4.821417	8.923953	4.102536	5.55E-07
SIAE	5.997812	4.27651	-1.7213	5.60E-07
MANSC1	2.487981	4.20978	1.721799	5.60E-07
BNIP3L	7.684832	11.02207	3.337242	5.62E-07
VSIR	4.447985	6.958296	2.510311	5.65E-07
AZGP1P1	7.103667	4.302614	-2.80105	5.70E-07
FCGR2A	1.658914	3.138474	1.47956	5.70E-07
NCF4	1.616491	3.835529	2.219037	5.73E-07
CTNNA1	29.30255	37.56062	8.258067	5.77E-07
TRIP12	7.207776	9.062537	1.854761	5.78E-07
IL18BP	2.318832	3.842552	1.523721	5.81E-07
HLA-DOA	2.080645	5.066736	2.986091	5.81E-07
BCCIP	8.469934	10.10783	1.637893	5.83E-07
PCCA-DT	4.749696	6.989214	2.239518	5.84E-07
SLC25A30	8.204512	5.337151	-2.86736	5.87E-07
CAPRIN1	12.8268	16.27139	3.444597	5.93E-07
TAB1	6.739767	8.169043	1.429276	6.01E-07
NAB2	6.553217	9.059114	2.505897	6.02E-07
MYRF	5.380198	11.75726	6.377064	6.04E-07
URI1	7.927913	10.34734	2.419427	6.07E-07
CYTH4	1.180963	2.256474	1.075511	6.12E-07
TRAK2	3.949836	5.699915	1.750078	6.14E-07
SEMA3E	0.088415	1.382817	1.294402	6.17E-07
COX5B	97.8981	128.1206	30.22246	6.19E-07
VPS54	5.801344	8.07211	2.270766	6.19E-07
HIGD1A	25.00344	18.78533	-6.21811	6.22E-07
GPRC5B	2.285989	4.953218	2.66723	6.22E-07
VANGL2	0.317617	2.213725	1.896108	6.23E-07
SPP1	177.4957	706.8145	529.3188	6.34E-07
PGD	30.54938	47.27829	16.72891	6.40E-07
AARS2	4.368378	5.426088	1.057709	6.42E-07
MIR647	3.018225	4.878522	1.860298	6.42E-07
FAM102A	8.1369	15.92453	7.787629	6.43E-07
MST1R	0.44271	2.386392	1.943682	6.49E-07
ABI3	2.756964	4.708093	1.95113	6.51E-07
DIDO1	3.537625	4.571619	1.033994	6.54E-07
PARK7	70.4008	87.11331	16.71251	6.65E-07
ARG1	180.8639	107.6222	-73.2417	6.66E-07
ADK	11.29911	8.855569	-2.44354	6.67E-07
SNAPC4	2.991463	4.013466	1.022003	6.68E-07
FLII	14.18102	19.3803	5.199278	6.73E-07
USP20	4.4856	5.555418	1.069818	6.74E-07
AL590867.	7.547241	11.4897	3.942455	6.75E-07
PI4K2A	5.711071	7.185486	1.474415	6.75E-07
PARP12	7.58241	9.891493	2.309083	6.78E-07
ARHGDIB	24.4586	42.54128	18.08268	6.79E-07
PTP4A3	8.135823	15.35088	7.215057	6.83E-07
HIF1A	10.87558	20.79735	9.921765	6.84E-07
RUSC2	3.80061	7.12521	3.3246	6.87E-07
NKG7	5.333292	14.55473	9.221434	6.88E-07
VTA1	3.667639	4.699884	1.032245	6.88E-07
CHEK2	2.026929	3.124071	1.097142	6.91E-07
PLEKHG6	1.865369	3.420896	1.555527	6.92E-07
DDX18	6.169333	7.730334	1.561001	6.93E-07
MALAT1	6.842262	10.19972	3.357459	7.00E-07
GORASP2	17.93367	21.54694	3.613275	7.02E-07
LOXL2	2.170518	3.910091	1.739573	7.03E-07

MFAP2	0.4493	1.813	1.363699	7.08E-07
ZNF204P	0.568546	1.620118	1.051571	7.09E-07
FMNL1	1.060256	2.337016	1.276761	7.11E-07
GZMH	1.605494	3.434656	1.829162	7.12E-07
SUGCT	7.707702	5.640738	-2.06696	7.13E-07
HORMAD:	3.013433	1.35238	-1.66105	7.15E-07
RHOV	0.500878	6.361211	5.860333	7.16E-07
SLC6A6	1.075608	3.169514	2.093906	7.23E-07
PKN3	2.788256	4.324821	1.536565	7.23E-07
GLB1L	1.860943	2.985696	1.124752	7.26E-07
RHOQ	3.002039	4.733499	1.73146	7.26E-07
SMIM6	1.99434	5.904689	3.910348	7.28E-07
MISP	1.452657	10.73033	9.277669	7.33E-07
PRKDC	6.328753	8.787944	2.459191	7.37E-07
FCER1G	13.54962	34.25455	20.70493	7.38E-07
SH3BGRL	12.88974	21.42207	8.532333	7.43E-07
PIM2	4.724994	8.143905	3.418911	7.57E-07
CYP3A4	503.0637	134.0304	-369.033	7.60E-07
CES3	8.365491	4.798214	-3.56728	7.61E-07
LY86	2.003635	4.233445	2.22981	7.65E-07
CDC42EP4	12.40323	16.7505	4.347271	7.67E-07
RAB5B	19.37595	23.05424	3.678289	7.75E-07
HMBS	5.485843	6.823985	1.338141	7.75E-07
ADGRA3	6.750884	4.279313	-2.47157	7.76E-07
FGFR3	14.66757	29.63172	14.96414	7.85E-07
WBP11	10.49545	13.02383	2.52838	7.89E-07
TRIM37	2.867202	3.888374	1.021172	7.93E-07
PDGFC	2.293535	4.974579	2.681044	7.99E-07
GCKR	17.74861	11.93331	-5.8153	8.01E-07
CCL5	10.16119	24.82485	14.66366	8.04E-07
TRIP6	25.69269	34.86391	9.171216	8.11E-07
GOLGA7B	0.506552	1.815872	1.309321	8.11E-07
ZFAND6	11.60646	13.71435	2.107891	8.12E-07
KIFC2	2.338009	3.902317	1.564308	8.24E-07
GUCA2A	0.510451	3.472325	2.961874	8.27E-07
AP3B1	6.548628	8.168426	1.619798	8.27E-07
SWAP70	3.545059	5.13564	1.590581	8.36E-07
CST2	0.369979	1.477769	1.10779	8.41E-07
DCAF7	9.343709	12.13105	2.787341	8.50E-07
RNF10	21.41809	25.05329	3.635203	8.51E-07
BCL3	18.43184	25.74377	7.311933	8.52E-07
OPLAH	18.116	13.21566	-4.90035	8.55E-07
CD83	2.20257	3.770783	1.568213	8.66E-07
LRRC61	8.58869	12.51026	3.921574	8.72E-07
REX1BD	9.439943	13.00373	3.563787	8.73E-07
DMTN	5.885251	10.79092	4.905672	8.78E-07
HTRA2	5.015907	6.069059	1.053152	8.86E-07
C1orf198	10.21451	15.46752	5.253006	8.86E-07
BTBD6	12.26884	15.78819	3.51935	8.90E-07
SEC23A	8.580649	6.676454	-1.90419	8.92E-07
SDC1	256.4642	194.4873	-61.9768	8.94E-07
VAV1	0.8558	2.123299	1.2675	8.96E-07
EPB41L4B	7.083675	4.938633	-2.14504	9.02E-07
ALKBH3	4.26561	5.514836	1.249226	9.07E-07
CERS2	71.35947	52.59236	-18.7671	9.22E-07
TNNT1	0.234088	3.653678	3.41959	9.27E-07
SLC27A1	1.793764	3.131146	1.337382	9.31E-07
HSP90AB1	336.6512	428.4854	91.83422	9.31E-07

QSOX2	3.681526	4.844577	1.163052	9.35E-07
AGAP3	5.408752	6.582262	1.173509	9.37E-07
CMBL	39.11133	25.85044	-13.2609	9.37E-07
SNRPD2	49.12984	120.4101	71.28031	9.40E-07
PTK7	1.411559	3.670019	2.25846	9.45E-07
ACTR10	7.533026	9.14995	1.616924	9.50E-07
GRB10	5.973244	7.872023	1.898779	9.55E-07
SPTAN1	13.8979	17.17692	3.279019	9.58E-07
ADIPOR2	25.36361	19.41593	-5.94767	9.60E-07
GPR108	18.64618	22.83184	4.185658	9.73E-07
TBX2	1.819359	2.96354	1.144181	9.78E-07
SLC47A1	20.10663	11.69348	-8.41315	9.80E-07
UBE2J1	10.66915	13.11471	2.445559	9.84E-07
LIMCH1	1.066469	2.640896	1.574427	9.88E-07
S100A4	13.20599	47.30648	34.10049	9.94E-07
SLC22A1	112.4704	48.94896	-63.5214	1.01E-06
CDC34	37.65568	47.27738	9.621696	1.01E-06
FNDC3A	10.41543	7.640961	-2.77447	1.01E-06
PAPLN	1.13623	3.026975	1.890745	1.03E-06
PCID2	3.447517	4.571897	1.12438	1.04E-06
VIM	22.99622	55.80983	32.8136	1.06E-06
NDUFA2	25.12944	32.78755	7.658109	1.06E-06
GRHL2	0.178402	1.503763	1.325361	1.07E-06
TOB1	64.49709	43.8686	-20.6285	1.07E-06
NIPSNAP1	59.65032	47.24371	-12.4066	1.07E-06
TMEM132	1.113811	8.487949	7.374137	1.08E-06
GSS	8.897858	10.75579	1.857933	1.08E-06
ADAM10	4.402979	6.296457	1.893477	1.08E-06
ZNF704	1.530815	2.541124	1.010309	1.08E-06
NAA50	12.57695	15.29763	2.720674	1.10E-06
CPNE7	0.703934	3.563817	2.859883	1.10E-06
TIMP2	12.28884	30.18782	17.89898	1.10E-06
FMNL2	1.590829	4.28233	2.6915	1.11E-06
BCL7A	2.628989	3.82346	1.19447	1.11E-06
TFDP1	8.600084	13.40781	4.807728	1.13E-06
CAPS	1.967287	8.963524	6.996236	1.13E-06
C9orf78	9.037632	10.5232	1.48557	1.14E-06
SAP130	4.714625	5.869567	1.154942	1.15E-06
RAB3GAP1	4.875881	6.187554	1.311673	1.18E-06
TMEM125	0.62994	3.12327	2.49333	1.18E-06
RCC1L	14.70881	17.54688	2.838067	1.18E-06
ACOT13	10.63673	7.944666	-2.69207	1.18E-06
CDH5	6.566264	4.755853	-1.81041	1.18E-06
MST1L	1.8135	3.142522	1.329022	1.19E-06
BICDL1	2.171825	3.710386	1.538561	1.19E-06
SETD5	3.984026	5.131809	1.147783	1.19E-06
HPR	191.6445	107.4323	-84.2122	1.20E-06
PNPO	11.18117	8.554421	-2.62675	1.20E-06
ABT1	10.54749	12.88124	2.333746	1.20E-06
SELPLG	2.901985	5.615767	2.713781	1.20E-06
ID3	12.49349	20.93649	8.442999	1.21E-06
EPN1	10.38963	12.59041	2.200784	1.21E-06
NME4	22.67897	31.19839	8.519422	1.22E-06
ATP6V0D1	9.986403	12.27487	2.288465	1.22E-06
EBI3	1.44844	2.968508	1.520068	1.22E-06
APOF	44.12149	22.68063	-21.4409	1.23E-06
ADAMTS1	1.805309	3.296742	1.491433	1.23E-06
GUCY1B1	1.713889	3.263922	1.550033	1.24E-06

BAG6	39.68517	47.92222	8.237051	1.24E-06
PSORS1C3	0.528355	1.944583	1.416228	1.24E-06
S1PR1	5.069118	3.636689	-1.43243	1.25E-06
CRIP2	11.63735	18.35431	6.716962	1.27E-06
HAL	30.11722	16.24399	-13.8732	1.28E-06
TFEB	1.972562	3.382813	1.410251	1.29E-06
OLFM2	41.1814	24.18178	-16.9996	1.29E-06
BEX5	1.367821	3.012494	1.644672	1.29E-06
MARCHF7	5.769645	7.28953	1.519885	1.30E-06
EVPL	0.397569	1.954076	1.556506	1.30E-06
PPP1R3B	16.8324	10.75789	-6.07451	1.30E-06
PLK3	1.853014	3.260824	1.407809	1.30E-06
BEX3	59.14466	91.23303	32.08837	1.31E-06
DONSON	3.205825	4.423193	1.217368	1.31E-06
SLC25A3	40.46528	48.19306	7.727786	1.31E-06
TST	210.122	157.0146	-53.1074	1.32E-06
AC104794	2.814395	3.998518	1.184124	1.32E-06
S100A3	0.335076	1.62359	1.288514	1.32E-06
NUP54	4.334612	5.423634	1.089022	1.33E-06
GNA13	7.625549	9.829735	2.204186	1.34E-06
BOP1	17.11297	28.05019	10.93723	1.34E-06
NDUFAF4	6.019411	7.548751	1.529339	1.35E-06
LHFPL3-A	0.118525	1.748362	1.629837	1.35E-06
FOLH1B	1.542848	0.495055	-1.04779	1.35E-06
TPT1	142.1928	183.896	41.70319	1.35E-06
ALDH18A1	14.64639	19.7888	5.142415	1.36E-06
VPS28	40.53357	55.4501	14.91653	1.36E-06
EMC7	43.07436	50.59976	7.525395	1.36E-06
DPP4	8.032369	4.847639	-3.18473	1.37E-06
MMP24OS	5.080591	7.249574	2.168983	1.38E-06
LSM1	3.515281	4.577097	1.061816	1.39E-06
ARAP1	5.956392	7.55922	1.602828	1.39E-06
CD74	224.9747	394.0507	169.0759	1.39E-06
PPP1R26	3.326019	4.657393	1.331374	1.40E-06
RPL39P3	12.78172	21.87323	9.091508	1.40E-06
FOXN3	4.980456	3.77405	-1.20641	1.40E-06
CCL4L2	1.539067	3.029784	1.490717	1.40E-06
AC009283	4.310003	5.756661	1.446658	1.42E-06
BTG1	31.68698	47.45009	15.7631	1.42E-06
MFSD12	5.849524	7.494239	1.644716	1.43E-06
KIAA2013	21.43753	26.98671	5.549178	1.43E-06
VAMP2	8.693561	12.03552	3.341959	1.45E-06
ISG20	2.192836	3.851666	1.65883	1.47E-06
CARD10	6.695528	9.700247	3.004719	1.48E-06
NDRG2	26.93028	19.1539	-7.77638	1.48E-06
BCL9	3.137237	4.805201	1.667965	1.49E-06
TRAPPC6A	30.51074	42.01268	11.50193	1.49E-06
IYD	2.575174	1.365898	-1.20928	1.49E-06
HUWE1	7.972012	10.10392	2.131905	1.50E-06
BAG1	6.819952	8.879693	2.059741	1.50E-06
MYL12A	48.2783	60.37599	12.09769	1.50E-06
IRF7	11.841	18.0318	6.1908	1.51E-06
PSMA3	23.17824	27.26279	4.08455	1.54E-06
RASSF4	6.542743	9.927041	3.384298	1.54E-06
MAPKAPK	8.076829	10.74906	2.672234	1.56E-06
GTPBP1	4.302701	5.557466	1.254764	1.57E-06
FSCN1	6.986316	19.24621	12.25989	1.57E-06
MRPL12	10.46093	16.34382	5.882897	1.58E-06

MPZL3	3.145106	4.831002	1.685896	1.60E-06
MINK1	5.359299	7.910257	2.550959	1.60E-06
SYBU	8.880631	5.7781	-3.10253	1.61E-06
ITGA2	1.139655	3.367973	2.228318	1.62E-06
ACOT4	5.937875	4.419913	-1.51796	1.66E-06
AADAT	3.192826	1.795675	-1.39715	1.67E-06
DUSP16	12.78479	9.813687	-2.9711	1.69E-06
CXCL1	3.946546	20.1557	16.20916	1.69E-06
HLA-DPA1	16.06956	29.83684	13.76728	1.71E-06
CDR2	6.090386	8.720339	2.629953	1.71E-06
NUP133	5.54704	7.100581	1.553541	1.74E-06
GPT	37.3662	22.30287	-15.0633	1.74E-06
KLHL36	2.205441	3.247355	1.041914	1.76E-06
LTBR	21.16647	25.79777	4.631302	1.76E-06
ARID3C	2.909943	1.59609	-1.31385	1.76E-06
CTXN1	0.322537	3.004257	2.681719	1.76E-06
CDH6	0.558861	3.627592	3.068732	1.77E-06
HCP5	2.35985	3.755793	1.395943	1.77E-06
SNX12	13.49349	16.99335	3.499855	1.79E-06
TOR3A	15.12046	21.18568	6.065213	1.79E-06
ZG16B	0.377349	2.783068	2.405719	1.80E-06
H2AJ	17.99757	26.32936	8.331793	1.82E-06
PPARD	7.598668	10.19055	2.591878	1.82E-06
MED30	5.571745	7.335625	1.76388	1.83E-06
SERPINA5	133.2668	89.09217	-44.1747	1.83E-06
MIF4GD	4.755236	6.227309	1.472073	1.83E-06
NLGN2	1.165453	2.360838	1.195385	1.83E-06
ASPHD1	2.303339	6.568694	4.265355	1.85E-06
IPO9	5.639831	7.21225	1.572419	1.86E-06
DLST	23.71766	19.6521	-4.06556	1.87E-06
BAZ1B	9.514189	11.93121	2.417017	1.89E-06
MTRES1	5.936652	7.282206	1.345555	1.89E-06
CKAP2	2.425617	3.554251	1.128634	1.91E-06
PLRG1	7.486184	8.956788	1.470604	1.91E-06
RNPEP	16.82188	20.74213	3.92025	1.92E-06
NES	6.379602	11.71955	5.339943	1.93E-06
PPIB	89.20067	107.8672	18.66653	1.93E-06
ENY2	9.611925	12.2875	2.675576	1.94E-06
SNHG29	12.92361	20.00786	7.084252	1.94E-06
ELL2	19.33455	13.78659	-5.54796	1.95E-06
VCL	4.18949	7.18497	2.99548	1.96E-06
CYSTM1	39.1955	54.90635	15.71085	1.97E-06
IER3	8.134924	17.39221	9.257288	1.97E-06
PFN2	2.384522	6.583658	4.199136	1.98E-06
DPP3	8.078254	10.36441	2.286155	2.02E-06
COL16A1	0.98969	2.639699	1.650008	2.02E-06
DCAF11	20.85675	16.13907	-4.71767	2.04E-06
CREBZF	4.166891	5.490378	1.323488	2.04E-06
PKN2	3.657458	4.794992	1.137534	2.07E-06
API5	11.55459	13.49783	1.943239	2.08E-06
CAPN2	12.14663	18.80619	6.659562	2.08E-06
FBXO46	4.303931	7.504677	3.200746	2.11E-06
TRABD	9.320821	12.04152	2.720701	2.11E-06
NEURL3	1.49231	9.012934	7.520625	2.12E-06
GCLC	12.14606	8.562705	-3.58335	2.13E-06
SRSF8	6.101147	7.84691	1.745763	2.13E-06
ENDOD1	2.382472	6.717012	4.33454	2.15E-06
GTF2H3	4.217735	5.316971	1.099236	2.16E-06

WIPF1	1.743378	3.319713	1.576334	2.20E-06
PSMB10	6.915109	9.733733	2.818625	2.21E-06
NDUFB9	9.474233	13.54332	4.069089	2.22E-06
RGS2	6.461898	19.50214	13.04025	2.22E-06
ELAC2	7.700587	9.315079	1.614492	2.23E-06
MBOAT1	2.898624	5.953227	3.054602	2.23E-06
KRT19	3.891639	76.04596	72.15433	2.24E-06
RTN2	1.73061	3.007332	1.276722	2.24E-06
ADH5	44.54546	36.42846	-8.11701	2.24E-06
POGLUT3	3.229779	4.646395	1.416616	2.24E-06
FOSL2	6.560406	10.46434	3.903932	2.25E-06
TAF6	3.209216	4.329982	1.120766	2.25E-06
MAP4K3	3.909575	6.237258	2.327682	2.26E-06
SLC16A5	0.529377	1.63956	1.110183	2.26E-06
TALDO1	64.77835	89.35749	24.57914	2.26E-06
AC015912	0.7464	2.503141	1.756741	2.26E-06
HDAC5	7.551831	9.371539	1.819708	2.28E-06
PRR15L	4.328753	12.29572	7.966966	2.28E-06
ACADL	2.578403	1.503736	-1.07467	2.29E-06
UCP2	7.612224	16.37072	8.758497	2.30E-06
STT3A	20.54115	25.34366	4.802509	2.30E-06
USE1	10.33857	13.59289	3.254323	2.31E-06
SLC29A2	5.305284	7.630203	2.32492	2.31E-06
TMEM126	8.514177	10.15784	1.643666	2.31E-06
RPL30	61.00642	82.55226	21.54585	2.31E-06
ANXA13	5.759622	21.05091	15.29128	2.33E-06
EMP3	5.127514	13.31779	8.190275	2.34E-06
LAMTOR2	56.32038	73.33875	17.01837	2.34E-06
AC026403	6.538623	16.01884	9.480217	2.35E-06
HIPK2	5.513299	3.968936	-1.54436	2.36E-06
SEMA4C	4.439732	5.932724	1.492993	2.36E-06
DCAF12	8.361733	10.38395	2.022221	2.38E-06
RERG	0.538076	1.961559	1.423483	2.39E-06
ITGA5	13.612	22.21569	8.603689	2.39E-06
COMTD1	7.821837	12.24795	4.426116	2.39E-06
NPNT	2.022898	7.785444	5.762546	2.42E-06
PPIL4	4.122139	5.236086	1.113946	2.42E-06
GPT2	35.83913	21.71933	-14.1198	2.43E-06
NAT8	20.39038	12.42206	-7.96832	2.45E-06
HUNK	0.461583	1.778373	1.31679	2.45E-06
CCDC28B	1.198933	2.296849	1.097916	2.48E-06
EEF1AKNM	9.53649	11.81382	2.277333	2.49E-06
AL161787.	2.336132	3.972343	1.636212	2.52E-06
HLA-DQB	7.049841	13.77135	6.721507	2.52E-06
RXRA	18.7906	14.63896	-4.15164	2.52E-06
METAP1	6.410389	8.195263	1.784874	2.53E-06
SLTM	6.342612	7.731673	1.389061	2.54E-06
RTP3	44.75948	24.51832	-20.2412	2.54E-06
CD3D	3.512395	11.89473	8.38234	2.55E-06
TAOK2	5.722338	6.974608	1.25227	2.55E-06
AQP11	7.286356	5.140496	-2.14586	2.56E-06
AP001065	3.334491	2.04739	-1.2871	2.56E-06
GOLT1B	8.966042	10.91282	1.946777	2.57E-06
GPBP1L1	11.51526	13.88706	2.371803	2.58E-06
AL162413.	1.679336	7.282876	5.60354	2.59E-06
EDEM1	11.72218	9.437467	-2.28471	2.61E-06
LINC0236:	2.221952	1.043825	-1.17813	2.61E-06
ANAPC13	16.28616	18.77172	2.485564	2.62E-06

ERLIN1	20.89754	16.30445	-4.59309	2.62E-06
UGCG	8.793069	12.34082	3.547752	2.64E-06
RFC3	2.80273	4.032055	1.229325	2.65E-06
NR1I3	17.34175	10.1106	-7.23115	2.66E-06
HSPB1P1	5.240583	11.65195	6.411367	2.66E-06
RAD21	20.3809	26.18021	5.799311	2.67E-06
NOLC1	14.93699	18.68737	3.750379	2.67E-06
ITIH5	0.799251	11.68362	10.88437	2.69E-06
SCTR-AS1	0.417287	3.005721	2.588434	2.69E-06
PRPF4B	5.014786	6.381614	1.366828	2.70E-06
ATP5PB	36.33963	43.297	6.957374	2.78E-06
WDR43	6.83803	8.476821	1.638792	2.78E-06
FAM133A	0.54871	1.74228	1.193569	2.79E-06
IL18	1.355117	9.73082	8.375703	2.82E-06
OSTF1	17.80541	21.43259	3.62718	2.82E-06
PTPRF	32.27597	47.29477	15.0188	2.84E-06
GPANK1	8.124812	10.25954	2.134732	2.85E-06
MED18	6.828349	8.052788	1.224439	2.85E-06
LYPD6	0.124793	1.212622	1.08783	2.88E-06
TYK2	9.847636	12.02504	2.177401	2.91E-06
ORMDL3	70.77671	55.92442	-14.8523	2.91E-06
NISCH	6.192734	7.496036	1.303301	2.92E-06
ATP5IF1	44.45228	55.11823	10.66594	2.92E-06
ELOVL6	8.467602	5.402639	-3.06496	2.94E-06
WDR91	2.814863	5.59589	2.781028	2.95E-06
PGK1	48.04874	67.65228	19.60354	2.96E-06
AL355353.	1.63045	3.668887	2.038437	2.98E-06
ATP2C1	5.664433	7.389506	1.725074	2.99E-06
CSF1R	4.359867	8.221339	3.861472	3.00E-06
RRAGB	2.594218	3.595988	1.00177	3.02E-06
GNAS	39.23065	52.34907	13.11842	3.05E-06
TCP1	20.07072	25.8757	5.804979	3.06E-06
COPS7A	21.83002	25.52321	3.693197	3.07E-06
CHMP5	18.64892	22.91197	4.263053	3.07E-06
WAPL	5.600206	7.384276	1.78407	3.08E-06
SNHG25	2.140999	6.156411	4.015412	3.10E-06
TMEM208	31.56029	38.82829	7.268005	3.10E-06
PRRC2C	7.671727	10.59577	2.924044	3.10E-06
MEN1	7.53549	8.83877	1.303281	3.12E-06
ABCF1	20.42527	24.74557	4.320301	3.13E-06
KRT7	8.051708	32.71389	24.66218	3.15E-06
IP6K1	7.969799	9.653455	1.683657	3.16E-06
SLC7A5	3.719522	7.077398	3.357875	3.22E-06
SPAG7	12.81377	16.22837	3.414605	3.23E-06
FPGS	14.25105	17.95228	3.701231	3.23E-06
TNFAIP8L1	7.844093	6.072367	-1.77173	3.25E-06
PSIP1	4.230174	6.075672	1.845499	3.26E-06
APBB1IP	5.341934	9.974746	4.632812	3.27E-06
SMC4	1.946551	3.217049	1.270498	3.27E-06
COPB1	18.90485	23.18158	4.276721	3.27E-06
CST7	3.212499	6.813242	3.600743	3.28E-06
CHCHD3	9.908324	12.31902	2.410696	3.28E-06
AC138696	2.08619	3.225937	1.139747	3.30E-06
ATP1A1	14.48465	35.41031	20.92567	3.30E-06
MED20	4.4876	5.669083	1.181484	3.32E-06
SLC25A22	7.053416	8.946855	1.893439	3.33E-06
ATP6V1D	9.392647	11.11808	1.725428	3.37E-06
CYB561D2	9.33058	11.65203	2.321453	3.37E-06

TCEAL8	21.98906	32.85743	10.86837	3.37E-06
BOK	42.41617	29.98215	-12.434	3.39E-06
NSMCE4A	5.858763	6.954095	1.095333	3.39E-06
MTLN	6.79704	9.139495	2.342455	3.41E-06
LINC0233:	0.447275	1.891345	1.44407	3.43E-06
UBE2B	17.75279	20.86765	3.11486	3.43E-06
CLUH	35.83494	29.53329	-6.30165	3.44E-06
C5orf22	4.18905	5.271993	1.082943	3.44E-06
LYPD6B	0.114458	4.537295	4.422837	3.44E-06
ARRB2	8.642614	12.59962	3.95701	3.44E-06
C1QBP	24.05142	30.48758	6.436159	3.49E-06
HCK	2.31843	4.13094	1.81251	3.50E-06
CLDN7	14.50175	26.3349	11.83315	3.53E-06
SELENOK	19.08884	22.57885	3.490012	3.57E-06
PNP	4.355187	5.846824	1.491637	3.59E-06
TNRC18	7.372491	9.537557	2.165066	3.62E-06
XBP1	159.477	130.6092	-28.8678	3.62E-06
DDX5	36.03026	44.054	8.023746	3.63E-06
HACD3	26.5308	21.43482	-5.09597	3.64E-06
EBF4	2.377844	4.725068	2.347225	3.67E-06
ARPP19	11.50537	14.32326	2.817889	3.69E-06
IFI27L1	3.656348	4.953277	1.296929	3.72E-06
MYRIP	6.274996	3.101832	-3.17316	3.72E-06
GALE	16.72468	21.63407	4.909391	3.72E-06
FAM98A	6.170536	7.268365	1.097829	3.73E-06
DUSP4	0.63343	2.111806	1.478376	3.74E-06
DCTN1	5.810588	7.393506	1.582919	3.77E-06
FN3KRP	13.68825	17.91053	4.222283	3.78E-06
SH2B2	0.910341	2.015062	1.104721	3.78E-06
UBE2E2	3.95782	5.263754	1.305934	3.78E-06
MCRIP2	11.14013	15.77518	4.635054	3.80E-06
DHX38	5.632004	7.213956	1.581952	3.80E-06
GZMA	3.897076	8.133567	4.236491	3.84E-06
PRDX1	121.0936	158.5178	37.42422	3.85E-06
AP1M2	6.240812	14.8102	8.569392	3.90E-06
RAC2	4.964421	15.40984	10.44542	3.90E-06
HPD	586.0757	281.8164	-304.259	3.96E-06
FUZ	1.733075	2.807539	1.074465	3.97E-06
NAA60	10.09995	11.95006	1.850108	4.00E-06
FBLN1	4.542297	13.0697	8.527401	4.01E-06
TGFB1	10.08916	19.83206	9.7429	4.01E-06
CAPN6	0.538865	4.958037	4.419172	4.03E-06
RTP4	5.86119	9.232589	3.371399	4.06E-06
SULT1B1	3.733537	1.59749	-2.13605	4.09E-06
VARS1	18.66645	23.23918	4.572726	4.13E-06
PHF13	4.713536	5.863603	1.150067	4.16E-06
PGM1	40.58151	32.02952	-8.55199	4.19E-06
F2RL1	5.081055	10.07314	4.992084	4.20E-06
KIAA0040	3.951224	5.952687	2.001463	4.22E-06
ZMYM3	4.483447	5.711976	1.228529	4.27E-06
TMEM14C	69.50975	84.53852	15.02877	4.28E-06
CTSH	11.6112	19.48655	7.875352	4.29E-06
EGLN3	0.890677	2.897509	2.006833	4.32E-06
GDF15	36.86509	73.17246	36.30737	4.45E-06
MAOB	90.21922	71.3942	-18.825	4.45E-06
ZG16	4.413044	2.270366	-2.14268	4.45E-06
ABCG2	6.337616	3.206308	-3.13131	4.48E-06
KIRREL1	1.33248	2.54662	1.21414	4.49E-06

CPQ	30.56352	23.53569	-7.02783	4.52E-06
GDI2	52.51799	61.53271	9.01472	4.53E-06
HLA-DQA	5.528042	12.70282	7.174774	4.54E-06
C1R	192.7885	139.7437	-53.0448	4.56E-06
SPPL2A	11.99044	10.08079	-1.90965	4.57E-06
CLDN10	0.583512	8.437357	7.853845	4.57E-06
DDX17	26.42477	34.2002	7.775436	4.58E-06
PAQR7	2.710168	3.902976	1.192808	4.61E-06
FAM8A1	30.70327	20.44263	-10.2606	4.61E-06
RPL14P1	3.707829	5.472312	1.764483	4.63E-06
NBL1	2.632799	9.45422	6.821421	4.64E-06
ANKIB1	2.993031	4.464513	1.471483	4.68E-06
BPGM	5.603379	7.061383	1.458004	4.68E-06
CDK5RAP:	5.499538	7.202962	1.703424	4.69E-06
DHX32	3.227676	4.860769	1.633093	4.69E-06
LPAR6	2.309118	3.432115	1.122997	4.71E-06
RTF1	6.9921	8.271981	1.27988	4.75E-06
FGG	1772.319	1164.101	-608.218	4.75E-06
TTC39C	21.26944	14.40784	-6.86159	4.78E-06
LYSMD1	4.014943	5.255844	1.240901	4.81E-06
KLHL9	3.645271	4.777353	1.132082	4.82E-06
KCTD9	2.211246	3.412749	1.201503	4.86E-06
TMEM94	6.58484	8.019966	1.435126	4.86E-06
CTSO	22.69876	17.02871	-5.67005	4.89E-06
TMEM9B	12.44065	15.23302	2.792374	4.96E-06
MRPL24	70.60989	91.89776	21.28787	4.97E-06
CNFN	1.166917	2.748708	1.581791	5.00E-06
CTDSP1	31.50207	36.59643	5.094365	5.01E-06
STK11	5.540039	6.603533	1.063494	5.07E-06
TBC1D16	3.840623	5.238306	1.397683	5.16E-06
LAPTM4B	31.86261	55.53011	23.6675	5.20E-06
TMEM127	15.64244	19.32844	3.685998	5.20E-06
HLA-DQA	5.3975	10.84544	5.447938	5.22E-06
TOP2B	11.58763	14.50743	2.919801	5.27E-06
CCDC88B	1.019604	2.485581	1.465977	5.35E-06
PTGES	1.937657	5.405194	3.467537	5.36E-06
ADORA2A	5.909856	4.0732	-1.83666	5.44E-06
CES1	861.8929	522.2547	-339.638	5.46E-06
TACC1	5.267823	9.163657	3.895834	5.48E-06
CTSF	33.01118	24.19371	-8.81748	5.50E-06
MMP15	25.04035	36.16548	11.12513	5.53E-06
TMUB1	28.36486	35.15878	6.793923	5.56E-06
HHEX	19.49507	25.04041	5.545335	5.57E-06
TOX4	6.37102	7.436118	1.065098	5.65E-06
NDRG1	14.2973	29.10775	14.81045	5.66E-06
SERPINE2	2.29551	6.577714	4.282204	5.66E-06
SYT13	0.427174	8.749968	8.322794	5.71E-06
CADPS2	3.410735	4.876059	1.465323	5.72E-06
LINC0102:	2.95897	4.440474	1.481504	5.74E-06
HAVCR2	1.514307	3.805557	2.291249	5.76E-06
AC011445	1.411613	4.451394	3.039781	5.76E-06
CAV2	7.157711	11.65821	4.5005	5.80E-06
CLIC3	0.543713	3.270884	2.727171	5.81E-06
C1QB	55.35411	112.4088	57.05469	5.82E-06
ITGAX	1.59251	2.605205	1.012695	5.83E-06
TMEM44-	3.302965	4.521894	1.218929	5.85E-06
ZNF302	3.65706	4.706647	1.049588	5.87E-06
ADRB2	4.130808	2.494206	-1.6366	5.87E-06

C6orf141	0.35603	3.908566	3.552536	5.88E-06
FERMT2	7.058031	5.593048	-1.46498	5.89E-06
DDX39B	5.332284	7.5674	2.235116	5.98E-06
DUS1L	22.02051	27.08966	5.069149	6.00E-06
PRRG2	2.57424	4.246806	1.672566	6.01E-06
B4GALT4	2.254019	3.746754	1.492734	6.03E-06
PPP1R14C	0.074256	2.36654	2.292285	6.03E-06
RAB10	26.82142	34.89178	8.07036	6.05E-06
OSER1	13.1775	15.59469	2.417189	6.05E-06
FERMT3	3.707628	6.824479	3.116851	6.11E-06
PIP5K1A	7.05288	9.395245	2.342365	6.12E-06
NPR2	5.609142	3.365812	-2.24333	6.13E-06
HLA-DRA	203.4742	380.5685	177.0943	6.13E-06
NOTCH2	3.43938	5.553986	2.114606	6.14E-06
GOT1	137.052	94.70274	-42.3493	6.15E-06
ATP5F1D	43.61626	62.91797	19.30171	6.22E-06
EIF2A	11.41779	13.69629	2.278501	6.26E-06
UGT2B27F	2.974496	1.497824	-1.47667	6.29E-06
SMIM22	0.049376	2.099142	2.049766	6.30E-06
CYP2E1	330.383	157.0865	-173.297	6.30E-06
UBC	123.4027	145.9444	22.54177	6.30E-06
EML3	5.76537	7.17949	1.414119	6.34E-06
ECD	6.500312	7.79005	1.289738	6.36E-06
NDUFB5	11.78955	13.92736	2.137811	6.39E-06
UGT2B7	144.1662	82.2366	-61.9296	6.44E-06
TGFB2	0.484795	2.331003	1.846209	6.46E-06
DNAJB11	7.636091	9.562834	1.926744	6.48E-06
THRSP	59.73848	21.33215	-38.4063	6.51E-06
SRRM2	21.20194	28.1354	6.933458	6.51E-06
OCRL	5.177464	6.786326	1.608863	6.51E-06
RASSF5	1.96703	3.067894	1.100864	6.57E-06
BRD4	5.524906	6.855695	1.330788	6.64E-06
CYP4F11	30.01056	20.62344	-9.38712	6.75E-06
MXD4	8.269321	10.88727	2.617952	6.76E-06
GALNT3	0.269067	2.664621	2.395554	6.79E-06
LPIN2	28.8361	20.67133	-8.16477	6.80E-06
HGFAC	36.31543	17.65233	-18.6631	6.86E-06
TMEM59	28.85947	33.71424	4.854771	6.88E-06
ACMSD	21.37116	14.35148	-7.01967	6.93E-06
AC107385	28.10343	16.03444	-12.069	6.95E-06
SLC39A8	5.48641	3.912179	-1.57423	6.99E-06
USP10	8.716214	10.48224	1.766029	7.01E-06
NSMCE2	6.912696	8.943866	2.03117	7.02E-06
AKR1C3	82.82598	117.1981	34.3721	7.10E-06
RETREG2	22.1888	25.94991	3.761115	7.16E-06
FCHSD2	3.279815	4.293043	1.013228	7.17E-06
TKFC	22.29625	12.61653	-9.67972	7.20E-06
DIAPH1	30.23967	25.14654	-5.09313	7.24E-06
KLHL12	9.790255	12.18416	2.393904	7.25E-06
PICK1	5.14695	6.632139	1.485189	7.27E-06
AL359893.	2.284364	1.20886	-1.0755	7.32E-06
TNFAIP6	0.213128	3.12835	2.915223	7.33E-06
EIF5	23.25561	19.86765	-3.38796	7.36E-06
FOLR1	0.330521	3.750727	3.420206	7.37E-06
CYP2D6	115.433	63.36973	-52.0632	7.54E-06
HPS1	10.45403	13.51691	3.062885	7.56E-06
EGFL7	16.02804	25.90841	9.880371	7.64E-06
NOA1	6.330944	8.083906	1.752962	7.64E-06

FBXO7	15.096	12.60219	-2.49381	7.68E-06
TMEM256	10.94053	18.8208	7.880268	7.69E-06
FCGR3A	8.052391	16.79708	8.744694	7.73E-06
CUTA	92.65725	119.6117	26.9545	7.86E-06
COG3	6.652772	5.265942	-1.38683	7.88E-06
AC026979	1.4308	3.081439	1.650638	7.88E-06
LAIR1	1.133621	2.160475	1.026854	7.88E-06
COQ3	5.533184	6.891694	1.358509	7.91E-06
SCUBE1	2.35567	0.907364	-1.44831	7.93E-06
IGFBP3	29.80838	53.32302	23.51464	7.99E-06
MRM1	4.649174	5.961426	1.312251	8.00E-06
WDR13	13.50359	18.70122	5.197636	8.00E-06
TBC1D1	4.302631	5.667162	1.364531	8.01E-06
STX12	6.981878	8.315874	1.333997	8.01E-06
CCDC71	7.785763	9.298359	1.512596	8.02E-06
JAK3	0.839271	2.338661	1.49939	8.09E-06
HKDC1	8.114968	15.9577	7.84273	8.13E-06
ADCK2	7.374081	9.268378	1.894297	8.17E-06
VAMP3	17.61642	21.97755	4.361129	8.21E-06
ADAMTSL	5.337233	3.502523	-1.83471	8.22E-06
EPS15	5.759561	6.9042	1.144639	8.29E-06
MYORG	17.68927	12.79981	-4.88946	8.37E-06
AC107983	2.323914	5.759367	3.435453	8.40E-06
SDS	225.5806	94.64733	-130.933	8.42E-06
SFXN4	15.11162	18.42547	3.31385	8.51E-06
ERMP1	4.69996	6.906914	2.206955	8.53E-06
HAGH	31.20595	23.4478	-7.75815	8.58E-06
MTFR1L	5.096611	6.102699	1.006088	8.61E-06
TMC5	0.709433	3.640221	2.930788	8.64E-06
TUFM	95.84182	110.9726	15.13079	8.66E-06
PRSS8	4.375927	11.17753	6.801606	8.72E-06
GMDS	7.018348	10.0823	3.063947	8.73E-06
AC092535	5.346336	9.692712	4.346376	8.74E-06
HAAO	54.0203	37.26487	-16.7554	8.77E-06
DNM2	7.626904	9.545748	1.918843	8.79E-06
IPO5	9.571275	12.88496	3.313682	8.80E-06
CLPP	13.28257	15.77877	2.496193	8.83E-06
POLR2K	28.66229	35.3561	6.693814	8.88E-06
SPRY4	3.10823	4.837189	1.72896	8.89E-06
OXA1L	27.61627	31.49877	3.882499	8.89E-06
SEC14L6	0.3876	1.600056	1.212456	9.07E-06
PUM1	7.557972	9.202182	1.64421	9.14E-06
GM2A	16.33818	20.80919	4.471007	9.15E-06
ERCC2	3.6508	4.762403	1.111603	9.15E-06
CIRBP	23.43336	29.75972	6.326364	9.24E-06
CNN2	13.18055	18.86953	5.688981	9.25E-06
MTND4P2	15.08997	7.545061	-7.54491	9.29E-06
COPB2	6.335193	7.556351	1.221159	9.30E-06
PAK2	12.35107	14.83999	2.488916	9.34E-06
MRFAP1	87.70172	101.105	13.40333	9.46E-06
WASF1	2.561864	3.885279	1.323415	9.54E-06
ODC1	32.08521	48.81871	16.7335	9.54E-06
ARRDC4	7.376764	4.450103	-2.92666	9.60E-06
PLEC	14.75003	20.20833	5.4583	9.67E-06
STK38L	1.874688	3.099626	1.224938	9.74E-06
PAIP1	11.88133	14.73234	2.851003	9.75E-06
POSTN	3.317418	8.695168	5.37775	9.81E-06
IL6R	23.25119	15.93391	-7.31727	9.83E-06

ASF1A	5.118924	6.502669	1.383746	9.87E-06
LAMB3	6.177915	19.58068	13.40276	9.89E-06
MEGF9	9.017723	6.816638	-2.20108	9.90E-06
ECHDC3	40.18367	29.42644	-10.7572	9.98E-06
ELOVL5	33.82994	25.96281	-7.86713	9.98E-06
NR2F2	6.774472	9.587339	2.812867	9.98E-06
TNKS1BP1	15.58629	19.99389	4.407598	9.99E-06
LINC0155	22.91775	8.25029	-14.6675	1.00E-05
ASGR1	148.1922	110.5905	-37.6018	1.00E-05
AXL	1.990025	3.656514	1.666489	1.00E-05
PIM3	24.12795	33.51766	9.389707	1.01E-05
SSR3	27.21404	32.42633	5.212287	1.01E-05
FAM99A	7.430155	3.430059	-4.0001	1.02E-05
GADD45B	66.33651	44.70148	-21.635	1.03E-05
H4C8	1.620545	3.241438	1.620894	1.04E-05
ACACB	6.700073	4.808413	-1.89166	1.04E-05
GSTZ1	5.291139	3.51923	-1.77191	1.05E-05
S1PR2	3.57283	4.956196	1.383367	1.05E-05
CYP2A6	395.6326	174.741	-220.892	1.05E-05
KCNJ16	0.155945	1.981942	1.825997	1.05E-05
CUTC	8.38078	10.23051	1.849728	1.05E-05
CCL4	1.897253	3.583396	1.686143	1.07E-05
C1orf210	4.773145	8.471376	3.698231	1.07E-05
ENPP1	7.211446	5.300716	-1.91073	1.08E-05
NXN	1.832466	4.073554	2.241088	1.09E-05
SLC7A9	6.938992	4.945225	-1.99377	1.10E-05
DPYSL2	5.261211	8.483599	3.222388	1.12E-05
A2M	436.7973	261.6604	-175.137	1.12E-05
ARF6	22.50387	27.22572	4.721849	1.13E-05
HSD11B2	2.863777	5.057619	2.193842	1.13E-05
AC242842	2.170752	3.186039	1.015288	1.13E-05
CASC9	0.845799	1.900285	1.054487	1.14E-05
ATRN	27.66969	20.51717	-7.15252	1.15E-05
HSPH1	6.700246	9.061752	2.361506	1.15E-05
PAM	2.541057	4.619032	2.077975	1.15E-05
PROM2	0.243629	2.451707	2.208078	1.15E-05
MZB1	1.10194	5.184602	4.082662	1.15E-05
UBR7	6.029615	7.337467	1.307851	1.15E-05
BLOC1S2	9.030804	10.63782	1.60702	1.16E-05
SLC22A7	90.31456	51.51912	-38.7954	1.17E-05
IL27	5.090684	3.313596	-1.77709	1.18E-05
ACBD3	11.74328	14.73957	2.996293	1.18E-05
PLAAT4	28.33897	45.68598	17.34701	1.19E-05
ITGB1-DT	0.330279	1.480318	1.150038	1.20E-05
CDK2AP2	37.46523	46.72347	9.258236	1.20E-05
MMP11	4.210848	13.20144	8.99059	1.20E-05
PEX11G	5.998487	4.448959	-1.54953	1.21E-05
PRNP	14.975	22.17292	7.19792	1.21E-05
TEX261	16.49943	19.20017	2.700749	1.21E-05
DAP	54.34783	67.55947	13.21165	1.22E-05
CYP3A43	1.927899	0.847556	-1.08034	1.22E-05
SLFN11	1.055861	2.069325	1.013464	1.23E-05
SLC35B1	12.1102	14.45453	2.344327	1.24E-05
AC136628	1.058877	2.156908	1.098032	1.24E-05
CRB3	8.96736	12.12049	3.153133	1.24E-05
PREPL	2.888774	3.967159	1.078385	1.25E-05
CDIPT	31.95835	38.5367	6.578354	1.25E-05
MFNG	2.423149	4.024101	1.600952	1.26E-05

DNAJB12	8.702838	9.947742	1.244904	1.26E-05
GMCL1	4.093396	6.055059	1.961663	1.26E-05
MRPL13	10.56691	13.46874	2.90183	1.26E-05
ACBD5	5.975485	4.751826	-1.22366	1.27E-05
EEF1A1P6	3.385076	5.08611	1.701034	1.28E-05
AL365181.	3.23241	5.990006	2.757596	1.28E-05
DUSP2	1.40674	3.559667	2.152927	1.29E-05
DUSP5	6.431062	11.53261	5.101544	1.29E-05
GPR183	2.000267	3.864935	1.864668	1.29E-05
CPSF7	9.532625	11.74724	2.214618	1.29E-05
SIX2	0.234526	1.508483	1.273957	1.30E-05
NCF2	3.477269	6.345524	2.868254	1.30E-05
ZFPM2-AS	0.699485	1.771044	1.071559	1.31E-05
ZBTB41	2.266381	3.332067	1.065686	1.32E-05
KLHL2	5.374648	4.161541	-1.21311	1.32E-05
CPVL	5.383834	9.727258	4.343424	1.33E-05
PSMD3	25.39362	29.98144	4.587818	1.33E-05
SH3KBP1	4.767847	6.48149	1.713642	1.33E-05
DOK7	0.639559	1.770393	1.130834	1.36E-05
RNF19A	8.868269	13.33959	4.471323	1.36E-05
WDR6	11.30801	14.36029	3.05228	1.36E-05
AC110285	1.175474	2.464998	1.289524	1.37E-05
RPS27L	8.630739	10.69873	2.067986	1.37E-05
TIMM10B	7.267575	8.54284	1.275265	1.37E-05
GBA2	9.575711	11.85527	2.279555	1.38E-05
MOCS2	10.42235	8.469353	-1.953	1.38E-05
ACTN4	47.01687	61.0892	14.07233	1.38E-05
MPHOSPH	6.900777	8.097693	1.196915	1.39E-05
SMDT1	22.91946	27.7927	4.873235	1.40E-05
PHF10	5.610044	7.078228	1.468184	1.43E-05
MRPS17	4.119999	5.22909	1.109092	1.43E-05
RNF122	1.933433	3.566679	1.633247	1.43E-05
USP14	8.926022	10.78914	1.863117	1.43E-05
A4GALT	1.859565	3.300444	1.440879	1.43E-05
ZNF22	9.02067	11.09869	2.078018	1.44E-05
EEF1A1	248.674	319.9031	71.22901	1.45E-05
TOP1	14.05378	16.99407	2.940289	1.46E-05
ABCC4	1.47317	2.730316	1.257146	1.46E-05
NDUFV3	7.374725	8.678271	1.303546	1.49E-05
TRPV2	1.987204	3.922206	1.935001	1.49E-05
HPGD	19.54974	9.370626	-10.1791	1.51E-05
RNF20	7.110776	8.758704	1.647929	1.52E-05
CAPZA2	8.738764	11.36933	2.630562	1.52E-05
ARL8B	16.3087	19.37241	3.063717	1.54E-05
SCTR	1.121679	13.82369	12.70201	1.54E-05
RPS28P4	20.39727	44.66057	24.2633	1.54E-05
CXCL6	3.0168	14.73024	11.71344	1.56E-05
PPFIBP1	1.905007	3.115244	1.210237	1.56E-05
SAYSD1	5.810691	7.421791	1.6111	1.57E-05
STK17B	1.314488	2.650794	1.336305	1.57E-05
VAPA	8.942968	10.45795	1.51498	1.57E-05
SULF1	1.869011	4.457646	2.588635	1.57E-05
EIF3J	15.8926	18.51672	2.624113	1.58E-05
WRNIP1	11.06508	13.33134	2.26626	1.58E-05
LIPC	25.70951	16.96037	-8.74914	1.59E-05
UTP3	10.55879	12.33967	1.780884	1.59E-05
PATZ1	6.420898	8.230492	1.809594	1.60E-05
RBM47	7.289292	9.300087	2.010796	1.60E-05

LAMTOR4	26.50436	51.5122	25.00784	1.61E-05
NXPH4	1.609609	6.319223	4.709614	1.62E-05
MRPS25	6.320627	7.661675	1.341048	1.64E-05
TUSC8	2.735548	1.50697	-1.22858	1.64E-05
SHISA5	34.35818	40.86887	6.51069	1.66E-05
LCP1	11.94927	20.17123	8.221967	1.66E-05
LRP6	5.478879	4.120474	-1.3584	1.67E-05
LIMK2	4.994044	7.217355	2.223311	1.68E-05
SEMA3C	0.334279	1.526678	1.192399	1.71E-05
UGT2B4	409.837	268.0147	-141.822	1.71E-05
PAWR	3.480544	4.656595	1.176051	1.75E-05
ARF4	55.6303	68.47534	12.84503	1.77E-05
BLZF1	4.875459	6.287794	1.412335	1.78E-05
PPP1R14D	0.327823	2.514066	2.186242	1.79E-05
ABCA3	0.957118	2.896538	1.93942	1.79E-05
CALD1	28.91115	23.89758	-5.01357	1.80E-05
DAP3	25.61504	30.23188	4.61684	1.80E-05
PPT2	2.996295	4.07209	1.075795	1.80E-05
AL590550.	1.295179	3.689461	2.394282	1.81E-05
MORF4L2	62.4177	74.82789	12.41019	1.82E-05
ANKRD10	2.073511	4.357014	2.283503	1.82E-05
EIF4B	33.99258	43.61573	9.623156	1.83E-05
ADM	6.686807	11.11081	4.424003	1.85E-05
VKORC1	7.791937	10.90004	3.108101	1.85E-05
AC027307	5.288625	6.664078	1.375453	1.87E-05
EPHB6	0.961433	3.55752	2.596087	1.88E-05
TYSND1	5.754961	7.409388	1.654428	1.89E-05
EXOSC4	16.83045	28.9993	12.16885	1.90E-05
ZNF385A	3.390884	6.804098	3.413214	1.91E-05
SLC44A1	5.804044	7.513359	1.709315	1.92E-05
TRIM44	4.534549	5.551431	1.016882	1.92E-05
RNU6-118	3.084472	1.153007	-1.93147	1.92E-05
LINC0143(	0.478972	4.153573	3.6746	1.92E-05
TM7SF2	59.85948	40.87483	-18.9846	1.92E-05
METRN	4.906114	7.5567	2.650587	1.93E-05
SEZ6L2	8.619584	15.6396	7.020017	1.94E-05
AC026740	0.847785	2.467942	1.620157	1.95E-05
UBB	153.756	127.4889	-26.2672	1.95E-05
IL17RE	2.244081	3.899605	1.655524	1.97E-05
LIPH	0.430298	2.339788	1.90949	1.97E-05
NOTCH1	2.641157	3.820724	1.179567	1.98E-05
ARFGAP2	14.67631	16.78095	2.104641	1.98E-05
PEX16	13.18043	15.74562	2.56519	2.00E-05
DEK	11.10689	14.39507	3.28818	2.00E-05
SEC23B	12.46413	15.44494	2.980811	2.02E-05
TMEM86A	1.219068	2.24094	1.021872	2.03E-05
ATP6V0E2	12.91116	8.482019	-4.42914	2.03E-05
MYO15B	3.875693	5.947276	2.071583	2.03E-05
CSGALNA	1.259661	3.995105	2.735445	2.03E-05
MMP7	4.086345	38.46615	34.3798	2.05E-05
OSBP	18.4069	15.90865	-2.49824	2.05E-05
SLC50A1	32.47316	42.55958	10.08642	2.05E-05
C5AR1	2.457062	3.656501	1.199439	2.05E-05
AL451165.	3.596325	5.245619	1.649294	2.06E-05
SLC66A2	29.55141	23.90902	-5.64239	2.07E-05
PBX2	9.714325	12.66498	2.950654	2.07E-05
C3P1	20.19515	11.86581	-8.32934	2.07E-05
CCDC51	5.558634	6.579862	1.021228	2.08E-05

NUAK2	3.370896	7.652175	4.281279	2.10E-05
MAPKAP1	7.459256	8.595805	1.136549	2.11E-05
BCS1L	6.569477	8.106638	1.537161	2.11E-05
GATAD1	5.209547	6.734606	1.525059	2.11E-05
TIGD2	5.775989	4.182501	-1.59349	2.11E-05
CYS1	0.431258	6.047578	5.616321	2.11E-05
SLC22A9	23.61147	14.72399	-8.88749	2.12E-05
THRA	2.566855	3.900105	1.33325	2.13E-05
FZR1	7.138562	8.580961	1.442399	2.13E-05
BIRC3	6.973551	15.26226	8.288705	2.18E-05
SMAD7	2.386826	3.451418	1.064593	2.21E-05
ARL6IP5	22.22603	28.22855	6.002517	2.22E-05
ORAI3	6.116379	7.910349	1.79397	2.22E-05
CCNI	27.93634	36.60496	8.668617	2.22E-05
TDP2	16.10687	19.56823	3.461359	2.24E-05
TM9SF4	16.45906	19.39556	2.9365	2.24E-05
BRD1	3.611579	4.653767	1.042188	2.25E-05
SCAMP5	3.034182	6.36907	3.334888	2.25E-05
VCP	31.86808	36.42045	4.552368	2.25E-05
GHDC	9.292284	11.30219	2.009907	2.25E-05
SNX6	6.894673	8.668318	1.773644	2.26E-05
SRGN	23.31503	37.59803	14.283	2.27E-05
HES1	18.44077	24.90301	6.462239	2.27E-05
PITRM1	3.909724	5.240909	1.331185	2.29E-05
SNN	5.246433	7.18903	1.942597	2.31E-05
SF3A1	13.18755	15.81059	2.623035	2.32E-05
ARVCF	2.568847	3.571827	1.002979	2.32E-05
TGIF1	4.841883	6.444016	1.602133	2.33E-05
HMGCL	27.74127	21.90541	-5.83585	2.34E-05
CIC	6.392426	8.072778	1.680353	2.36E-05
CHPF	29.99781	40.42879	10.43098	2.40E-05
PBX3	4.058984	6.704418	2.645434	2.44E-05
FA2H	0.398919	2.240214	1.841295	2.45E-05
INTS7	5.217362	6.472315	1.254953	2.45E-05
TRIM27	9.342953	11.05402	1.711069	2.46E-05
ABHD3	8.825746	12.27052	3.444772	2.46E-05
OGFOD3	5.083396	6.2642	1.180804	2.48E-05
FUT3	0.226114	2.018115	1.792001	2.50E-05
CACFD1	20.95092	26.27469	5.323769	2.50E-05
C1GALT1	2.719703	3.740468	1.020766	2.55E-05
FBH1	4.636353	5.927599	1.291246	2.56E-05
RAB8B	3.313456	4.686728	1.373272	2.57E-05
GNAZ	2.323141	3.931157	1.608016	2.58E-05
PKP2	4.292619	3.174616	-1.118	2.60E-05
IGFBP1	481.7153	287.3247	-194.391	2.60E-05
ADRA2C	2.267284	5.562297	3.295013	2.60E-05
PYGL	30.54468	22.28032	-8.26436	2.60E-05
APOC3	4743.592	3172.662	-1570.93	2.63E-05
RAP1A	8.944082	10.49283	1.548748	2.64E-05
SNHG19	17.78406	38.20597	20.42191	2.64E-05
RRAGD	5.327365	7.706887	2.379522	2.65E-05
KPNA6	5.029874	6.166059	1.136185	2.67E-05
TMPRSS3	1.529013	4.600001	3.070988	2.68E-05
RMDN3	10.63086	9.336481	-1.29438	2.69E-05
PLAUR	1.065309	3.93992	2.874611	2.70E-05
TOMM20	61.34714	75.81492	14.46778	2.70E-05
GPR137B	6.169383	9.123545	2.954163	2.73E-05
FAH	25.81374	20.63857	-5.17517	2.74E-05

CHST3	0.732739	2.48416	1.75142	2.75E-05
FAM171A1	9.771896	19.06529	9.293392	2.78E-05
IQSEC1	12.25782	10.09948	-2.15834	2.81E-05
SLC39A14	52.16543	37.29945	-14.866	2.82E-05
TSPYL2	3.477673	5.325521	1.847848	2.82E-05
KLK11	0.122667	3.214211	3.091544	2.82E-05
URB1-AS1	3.877319	5.02934	1.152021	2.84E-05
RORC	22.29852	15.79511	-6.50342	2.86E-05
PPID	13.2581	11.47252	-1.78558	2.86E-05
MCL1	50.34729	64.10241	13.75512	2.86E-05
NDUFS1	9.471535	8.0313	-1.44023	2.86E-05
YEATS4	5.644385	7.014482	1.370097	2.87E-05
ZMPSTE24	15.20326	18.1355	2.932239	2.87E-05
EIF3E	6.201116	8.128143	1.927027	2.87E-05
KLC4	13.70831	10.6802	-3.02811	2.88E-05
TRIAP1	22.78461	26.05684	3.272234	2.89E-05
UBXN6	20.76968	24.2412	3.471517	2.91E-05
DPP9	8.952554	10.69122	1.738669	2.92E-05
NQO1	35.55275	81.74793	46.19518	2.92E-05
COL5A3	10.36302	6.452174	-3.91084	2.93E-05
ENTR1	11.96109	14.79702	2.835932	2.93E-05
ABHD15	7.344834	5.627222	-1.71761	2.93E-05
AC245041	0.298736	1.601486	1.30275	2.94E-05
ZPR1	5.570645	6.699136	1.128491	2.96E-05
IGSF6	2.176903	3.396068	1.219165	2.97E-05
PIP4P1	6.503996	7.681402	1.177406	2.97E-05
GOLGB1	7.348287	8.92822	1.579933	3.04E-05
AURKA	5.727201	8.51635	2.789149	3.06E-05
PYCR3	6.785382	9.07554	2.290158	3.10E-05
PRAME	1.442477	5.615882	4.173405	3.12E-05
IMMT	21.51954	25.20044	3.680902	3.14E-05
APCS	977.8116	665.89	-311.922	3.15E-05
CX3CL1	5.889646	10.1482	4.258557	3.16E-05
TWSG1	2.346884	3.376984	1.0301	3.17E-05
SMO	15.06106	11.05474	-4.00631	3.17E-05
ISCU	15.31084	17.95367	2.642825	3.18E-05
ABCC3	17.88746	26.28082	8.39336	3.21E-05
P3H3	0.78023	2.244173	1.463943	3.21E-05
CCDC50	8.704311	10.46853	1.764216	3.22E-05
GNAI2	27.6096	32.60754	4.997945	3.23E-05
AP3D1	17.06498	20.59213	3.527151	3.23E-05
MT-CYB	7163.321	5616.166	-1547.16	3.24E-05
AC097639	4.500966	3.152778	-1.34819	3.25E-05
CD40	6.847917	9.967498	3.11958	3.27E-05
ITGB2	5.371867	9.454629	4.082763	3.27E-05
FETUB	43.657	24.99713	-18.6599	3.28E-05
RAF1	5.046016	3.881718	-1.1643	3.28E-05
SORT1	5.790209	8.048933	2.258724	3.29E-05
HK1	3.195277	6.008028	2.812751	3.31E-05
HYAL2	8.991164	10.79521	1.804047	3.33E-05
VTCN1	1.204703	14.14172	12.93702	3.37E-05
SLC35F6	17.81743	22.0978	4.280365	3.38E-05
KANK1	8.986868	6.817354	-2.16951	3.39E-05
MPG	9.2824	11.04218	1.759779	3.40E-05
CXCL8	6.648694	20.89463	14.24594	3.41E-05
AKIRIN1	13.46119	16.56405	3.102859	3.44E-05
PLIN1	3.064676	1.565648	-1.49903	3.44E-05
SHH	3.85412	2.737885	-1.11623	3.57E-05

OR2I1P	27.7802	58.93829	31.15809	3.58E-05
TREH	1.897244	0.851297	-1.04595	3.60E-05
UNC50	10.5715	12.18493	1.613425	3.61E-05
C5orf46	0.343267	1.802969	1.459702	3.64E-05
SCCPDH	109.9534	86.51658	-23.4368	3.65E-05
UBTD1	7.732806	9.622048	1.889241	3.67E-05
TMEM134	5.010158	6.041979	1.031822	3.70E-05
GFPT1	8.128052	10.30497	2.176915	3.72E-05
TGFBR1	4.3537	5.944763	1.591063	3.76E-05
LIMD2	2.267536	5.871613	3.604077	3.79E-05
C4BPB	96.46275	71.58434	-24.8784	3.80E-05
TTC36	4.516673	1.961411	-2.55526	3.81E-05
NMRAL1	12.18763	16.07276	3.885128	3.84E-05
SLC37A4	21.05948	16.58323	-4.47625	3.85E-05
EPO	0.919369	3.639112	2.719742	3.86E-05
DMWD	2.092022	3.761512	1.66949	3.94E-05
PFKM	1.995987	3.712565	1.716578	4.03E-05
PEPD	40.55273	31.21087	-9.34186	4.07E-05
FSTL3	9.309854	18.98861	9.678753	4.08E-05
CTSW	1.653766	3.363221	1.709456	4.08E-05
DAB2IP	4.692305	7.032066	2.33976	4.13E-05
COX6C	57.94386	80.01064	22.06678	4.15E-05
RSU1	10.73027	13.60576	2.875489	4.16E-05
ERAP2	4.307406	6.904167	2.596761	4.18E-05
XRN2	17.01682	20.78809	3.771268	4.18E-05
PDSS2	6.787814	5.676897	-1.11092	4.19E-05
HSD3B7	20.01705	26.36488	6.347825	4.19E-05
WSB2	11.7612	14.56774	2.806547	4.26E-05
JSRP1	0.153093	1.243614	1.090521	4.26E-05
ZC3H15	16.20203	18.86194	2.659907	4.27E-05
CNBP	63.7352	71.75354	8.018346	4.27E-05
ENPP7	7.986473	4.522407	-3.46407	4.28E-05
GSDMB	3.101956	4.805526	1.70357	4.29E-05
IGHG1	165.6836	784.3609	618.6773	4.30E-05
TRIB3	24.37836	36.33631	11.95795	4.30E-05
TKT	27.44143	40.71903	13.2776	4.35E-05
AMIGO2	1.099955	2.256757	1.156801	4.35E-05
RFX5	6.480725	8.641261	2.160536	4.36E-05
AL079338.	0.092301	1.265592	1.173291	4.38E-05
SMAD5	5.783736	7.84727	2.063533	4.42E-05
C1QC	51.89148	97.45971	45.56823	4.44E-05
SPP2	83.42779	44.31547	-39.1123	4.46E-05
NUP50	4.984618	6.030488	1.04587	4.47E-05
CISD1	9.82359	11.92056	2.096971	4.48E-05
TPCN1	6.161982	8.073808	1.911827	4.52E-05
LMF2	32.47146	38.23252	5.761055	4.56E-05
CCL16	57.9372	37.42599	-20.5112	4.57E-05
GPSM3	4.910015	10.43446	5.524447	4.60E-05
SMURF1	4.667331	6.034462	1.367131	4.68E-05
IGFBPL1	0.726145	2.725152	1.999006	4.70E-05
SELENOS	15.6709	19.47104	3.80014	4.73E-05
GFUS	24.5914	32.33473	7.743328	4.73E-05
FTH1P20	4.419799	6.369204	1.949405	4.74E-05
AHNAK2	0.169147	1.442349	1.273202	4.74E-05
TMEM181	6.023051	7.764895	1.741844	4.78E-05
SECTM1	5.159073	8.541312	3.382238	4.81E-05
NEU1	23.83934	31.88345	8.044109	4.82E-05
NECTIN1	2.105951	3.852512	1.746561	4.84E-05

APMAP	138.9172	111.8679	-27.0493	4.85E-05
NCKAP1	3.7322	4.768434	1.036234	4.86E-05
XPOT	9.213641	11.96365	2.75001	4.87E-05
TACSTD2	1.827206	25.35256	23.52535	4.89E-05
GOLGA2	8.482494	10.08056	1.598071	4.90E-05
EPS8	5.242007	7.438129	2.196122	4.91E-05
GNA12	6.381291	8.089376	1.708085	4.98E-05
ATF6B	17.3263	21.21485	3.888552	4.99E-05
CRHBP	2.323108	0.639466	-1.68364	5.01E-05
EVC	1.396136	2.962967	1.56683	5.03E-05
TP53I13	6.627893	8.721345	2.093453	5.05E-05
ULK1	8.280391	10.35753	2.077138	5.06E-05
HGS	4.742677	5.936388	1.193711	5.13E-05
ISYNA1	7.085918	18.57576	11.48984	5.14E-05
SLC48A1	5.393725	6.967808	1.574083	5.20E-05
ABCD1	7.495209	10.11975	2.624538	5.22E-05
UGT1A10	0.140299	1.307614	1.167315	5.25E-05
MTSS1	13.24843	10.52464	-2.72379	5.26E-05
TWF1	8.90834	10.86086	1.952521	5.29E-05
LAMTOR3	5.26596	6.278236	1.012277	5.30E-05
PKDCC	4.129401	10.67495	6.545549	5.31E-05
SH3BGRL2	16.26665	12.11143	-4.15522	5.34E-05
EHD2	9.276852	14.45631	5.179456	5.37E-05
FBXO8	10.43463	8.42113	-2.0135	5.41E-05
CD8A	1.556155	3.702528	2.146373	5.41E-05
TJP3	3.992875	6.010879	2.018004	5.42E-05
CLDN15	19.80119	12.70295	-7.09823	5.44E-05
ABCC6P1	6.106316	4.558701	-1.54761	5.52E-05
CHP1	57.23227	48.17509	-9.05718	5.55E-05
LINC0085	1.890599	2.902397	1.011798	5.60E-05
RAB12	8.131016	9.359479	1.228464	5.60E-05
NSMF	6.859921	9.089178	2.229257	5.62E-05
MGP	8.834938	17.61951	8.784574	5.65E-05
AL008729	1.63016	3.201172	1.571012	5.73E-05
TMEM50B	5.269621	6.271863	1.002242	5.76E-05
BAIAP2	5.428594	4.184606	-1.24399	5.81E-05
TMEM184	3.264769	4.667423	1.402654	5.83E-05
COX7B	42.24963	52.24308	9.993448	5.85E-05
OGA	11.62387	13.87708	2.253214	5.86E-05
AL161668	2.618924	1.360473	-1.25845	5.92E-05
B4GALNT	0.184664	1.638243	1.453579	5.93E-05
CDH1	28.43737	42.87865	14.44127	5.94E-05
PLXND1	8.0909	11.19733	3.106433	5.96E-05
CD44	4.538054	9.715553	5.177499	6.00E-05
RARS2	8.119251	9.758542	1.639292	6.05E-05
DPM3	55.38598	80.45802	25.07204	6.12E-05
PANK3	8.465235	6.873865	-1.59137	6.14E-05
PUF60	38.96938	48.69632	9.726935	6.15E-05
COMMD9	4.743181	7.184055	2.440873	6.21E-05
FH	114.6847	91.89253	-22.7921	6.21E-05
HTRA3	2.333783	5.674267	3.340484	6.21E-05
JUND	84.17082	112.2415	28.07068	6.24E-05
IL34	1.868191	3.43966	1.571468	6.25E-05
CYBB	3.209903	6.188152	2.978249	6.26E-05
SERPINA7	100.3365	61.57362	-38.7629	6.30E-05
NPC1	3.230985	4.380377	1.149392	6.35E-05
MISP3	3.661998	5.938583	2.276585	6.39E-05
DDX6	6.104868	7.31819	1.213322	6.41E-05

HAGLR	0.834633	2.12191	1.287277	6.46E-05
NINJ1	29.28094	35.48336	6.20242	6.46E-05
DEF6	1.850966	4.869792	3.018826	6.50E-05
RDX	10.6331	8.640472	-1.99263	6.56E-05
ACTR2	31.56455	38.37623	6.811675	6.56E-05
HK2	1.307208	3.449289	2.142081	6.66E-05
CRIM1-DT	2.599842	5.219901	2.620059	6.67E-05
KIF13B	2.384131	3.985651	1.601521	6.73E-05
CLDND2	1.143625	2.480535	1.33691	6.74E-05
MSRA	7.185639	5.603442	-1.5822	6.80E-05
RPS28P7	243.4602	406.9323	163.4721	6.85E-05
FAM174B	2.023107	3.360014	1.336907	6.92E-05
FLJ20021	3.457663	4.681355	1.223692	6.94E-05
PRKACB	3.230622	4.393559	1.162937	6.96E-05
YIPF3	45.83716	54.32754	8.490379	6.99E-05
RASSF9	0.354748	1.389662	1.034914	7.05E-05
ETNK2	21.784	14.01374	-7.77026	7.10E-05
TARBP1	4.083784	5.421788	1.338004	7.10E-05
C6orf223	0.769939	2.839308	2.069368	7.15E-05
RPLP0P6	3.692393	5.837732	2.145338	7.16E-05
GSPT2	2.566981	4.105167	1.538186	7.19E-05
AC005336	5.459032	3.341506	-2.11753	7.21E-05
AMACR	2.401028	1.290429	-1.1106	7.27E-05
SFXN5	5.665812	4.480291	-1.18552	7.28E-05
CLPTM1L	20.05977	24.03331	3.973541	7.32E-05
HCLS1	4.205842	7.322157	3.116315	7.34E-05
DZIP1	0.275461	1.427621	1.15216	7.35E-05
PSMF1	14.44321	16.44626	2.003056	7.40E-05
NCOA2	8.209489	6.481252	-1.72824	7.48E-05
PIK3CD	0.919117	1.982846	1.063729	7.54E-05
LEPROT	16.06819	20.44446	4.376273	7.56E-05
NDUFB1	23.81912	30.41109	6.591968	7.58E-05
NFKBIZ	2.91296	4.275062	1.362101	7.59E-05
ZDHHC5	15.47095	18.06969	2.598736	7.79E-05
PDCD1	0.778068	2.32379	1.545722	7.81E-05
IGKC	470.5689	2322.756	1852.187	7.83E-05
ID1	18.33098	33.89027	15.55929	7.84E-05
NAGS	15.5001	9.636565	-5.86354	7.87E-05
AKAP7	2.864662	5.411469	2.546807	7.87E-05
CLDN3	25.40084	45.76771	20.36688	7.89E-05
WAS	2.711248	5.141879	2.430631	7.95E-05
NDST1	15.42803	12.55556	-2.87247	7.96E-05
SNAP25	1.390459	4.451997	3.061538	8.09E-05
DOLK	10.26144	11.87636	1.614914	8.09E-05
ZDHHC20	4.465659	5.766293	1.300633	8.10E-05
TUBGCP6	4.200594	5.361067	1.160474	8.13E-05
RNF213	6.390646	8.041457	1.650811	8.16E-05
ZMIZ1	4.481802	6.619477	2.137675	8.16E-05
GNPAT	22.32903	27.01143	4.682393	8.22E-05
RHBDD2	36.06552	44.53896	8.473442	8.41E-05
NUPR1	2.585939	3.775614	1.189675	8.43E-05
CHRNA4	1.80514	0.756293	-1.04885	8.53E-05
TCF12	3.571793	4.649116	1.077324	8.55E-05
SUN1	5.830264	7.147853	1.317589	8.55E-05
ADD3	4.166384	6.267601	2.101218	8.63E-05
AC027117	10.54209	6.128473	-4.41361	8.64E-05
NSUN2	9.103737	10.59992	1.496183	8.65E-05
ZC3H3	9.402928	11.47837	2.075437	8.67E-05

GORAB	2.612121	3.862837	1.250716	8.71E-05
EIF4A2	34.28257	42.57091	8.28834	8.71E-05
SLC15A3	2.746965	3.892632	1.145667	8.79E-05
POFUT1	15.19518	12.70019	-2.49499	8.85E-05
CUL2	5.389686	6.401072	1.011386	8.91E-05
PDGFA	6.0104	9.489987	3.479587	8.95E-05
SMARCD2	22.31709	26.49466	4.177568	9.06E-05
FNDC3B	5.776477	7.541566	1.765089	9.07E-05
LIPG	3.535635	2.386163	-1.14947	9.09E-05
GART	5.314653	6.375183	1.06053	9.14E-05
CASP7	5.102559	6.317383	1.214824	9.15E-05
SNORD3A	4.427883	9.944075	5.516192	9.16E-05
TNIP1	31.94482	39.38627	7.441459	9.17E-05
SPAG4	2.214339	3.527305	1.312966	9.29E-05
SERTAD1	6.713277	8.785426	2.072149	9.30E-05
NDUFA6	56.59462	66.85099	10.25637	9.30E-05
AMPD2	4.883916	5.914774	1.030858	9.35E-05
NEAT1	9.713557	13.67739	3.96383	9.40E-05
LXN	2.144259	4.444084	2.299825	9.43E-05
SLC15A4	4.541187	5.564969	1.023782	9.45E-05
TCF25	7.69293	8.925443	1.232513	9.49E-05
SLAMF7	1.117778	2.470285	1.352507	9.49E-05
NDUFB6	32.14801	36.77842	4.630403	9.59E-05
SLC12A2	2.087534	7.967853	5.880319	9.62E-05
PDGFB	3.905708	5.899558	1.993849	9.69E-05
NORAD	36.47477	43.24169	6.76692	9.71E-05
CD72	0.781925	1.837049	1.055125	9.72E-05
PROX1	18.68109	14.14334	-4.53775	9.76E-05
PALM	2.538904	4.805525	2.26662	9.88E-05
HLA-DOB	0.428486	1.571093	1.142607	9.89E-05
MIR4664	7.577326	4.995206	-2.58212	9.95E-05
AC016735	1.504356	6.945197	5.440841	0.0001
LPIN1	3.855464	2.817945	-1.03752	0.000101
KRT23	8.575421	24.11889	15.54347	0.000102
CDS1	1.298664	2.47525	1.176586	0.000102
CA11	0.938701	2.355003	1.416301	0.000102
FKBP11	7.237846	9.681013	2.443167	0.000102
ZHX1	9.653993	7.703341	-1.95065	0.000102
LGALS3	26.44434	45.02286	18.57852	0.000104
SELENOO	26.20059	21.66687	-4.53372	0.000104
MT-ND4L	2938.002	2100.101	-837.901	0.000105
NDUFA4L2	7.017816	15.79331	8.775497	0.000105
UTRN	3.201786	4.323664	1.121878	0.000105
PRSS21	0.133438	1.341139	1.207701	0.000105
POGLUT2	3.805002	5.502165	1.697163	0.000106
EIF4G2	51.34256	62.70385	11.36129	0.000106
RPS6KB2	5.502309	6.521078	1.018769	0.000107
KDELR3	11.87752	17.31146	5.433943	0.000108
LIVAR	3.339589	2.005625	-1.33396	0.000108
ANKRD1	1.537415	7.900729	6.363314	0.000109
CBLC	10.071	14.22602	4.155022	0.000109
WNT10A	0.096066	1.10757	1.011504	0.000109
GABARAP	26.05901	19.36274	-6.69627	0.000109
ID4	1.055188	2.497329	1.44214	0.000109
MLPH	3.60406	6.255431	2.651371	0.000109
TM9SF3	20.78852	24.20511	3.416591	0.00011
GJB1	87.84243	71.16978	-16.6726	0.00011
APOL6	11.77203	9.244781	-2.52725	0.00011

AL365181.	0.659171	1.86111	1.201939	0.000111
EML4	11.78449	14.4054	2.620907	0.000112
NR2F6	29.93413	36.86876	6.934625	0.000112
PPA1	21.75941	26.25428	4.494867	0.000112
ATP9A	6.263113	8.535357	2.272244	0.000112
THY1	5.129567	7.156601	2.027034	0.000113
LPCAT3	4.041012	2.898231	-1.14278	0.000113
CADM4	4.662399	6.9324	2.270001	0.000114
CMTM8	23.70767	19.17999	-4.52768	0.000115
GPI	28.73242	36.61032	7.877907	0.000116
PLXNB3	0.340707	1.562374	1.221667	0.000116
ABRACL	8.930029	12.46995	3.539925	0.000117
CYP7A1	36.85042	19.30383	-17.5466	0.000117
TMEM98	8.586844	11.68654	3.099692	0.000117
POLR3D	1.416822	2.838218	1.421397	0.000117
M6PR	7.782399	10.21337	2.430971	0.000117
CISH	7.403333	5.232918	-2.17042	0.000117
LRIG3	2.349036	6.344653	3.995617	0.000118
MRPL41	107.4584	144.9684	37.50998	0.000118
ANAPC2	7.911748	9.15409	1.242342	0.000118
GCH1	14.50026	10.18761	-4.31265	0.000119
TNS1	5.882359	8.008779	2.12642	0.000119
TICAM1	10.99585	13.8925	2.896646	0.00012
ALDH1A1	457.568	329.0197	-128.548	0.00012
C9	115.121	56.68727	-58.4337	0.000121
IDH1	71.2339	58.06893	-13.165	0.000121
ACLY	17.23061	21.70537	4.474762	0.000121
IGF2BP1	1.319214	2.738547	1.419333	0.000122
BBOX1	7.687332	3.968184	-3.71915	0.000122
BCL2L11	3.554627	4.929346	1.37472	0.000122
MCAM	7.082613	12.85465	5.772037	0.000122
AHSG	1562.879	1049.69	-513.189	0.000123
KLF2	3.628548	5.755047	2.126499	0.000124
PTGFR	1.275533	2.873994	1.598462	0.000124
AFAP1-AS	0.393011	1.533798	1.140787	0.000125
LIX1L	6.454172	8.316844	1.862671	0.000125
BMF	2.326929	3.758404	1.431475	0.000126
GPX4	178.7951	215.7487	36.95361	0.000126
PPP6R1	9.77095	11.79692	2.025971	0.000128
CCND2	0.904251	2.894297	1.990046	0.000128
ARSA	18.06503	14.73107	-3.33396	0.000129
CSAD	6.021896	4.597367	-1.42453	0.000129
GPR137	8.11521	9.770734	1.655525	0.00013
SLC38A2	37.20096	28.69616	-8.5048	0.000131
ANKRD13.	4.21676	5.277479	1.06072	0.000131
COL1A2	23.26045	46.93088	23.67043	0.000132
PLEKHF2	5.59816	7.037836	1.439676	0.000133
TCEAL4	24.88213	29.66561	4.783479	0.000133
AL445205.	0.651483	2.147382	1.495899	0.000134
ELK4	4.453848	6.046533	1.592685	0.000136
TRIOBP	5.27259	6.89233	1.61974	0.000137
PODXL2	3.23653	8.26572	5.02919	0.000137
OLFML3	2.829276	6.401776	3.5725	0.00014
HSH2D	1.018504	2.620161	1.601657	0.000141
SYNE4	3.525809	5.274428	1.748619	0.000141
TRIM16	2.083061	3.969761	1.8867	0.000141
MAN1A1	31.65087	24.43526	-7.21561	0.000142
MARK4	6.661108	8.214621	1.553512	0.000142

AL450998.	1.44839	2.76846	1.320069	0.000142
PITPNM1	3.391953	4.732795	1.340841	0.000142
LINGO1	1.30107	2.912602	1.611532	0.000142
HPN	128.8062	105.8284	-22.9778	0.000143
ACSL5	30.0266	19.17523	-10.8514	0.000143
DGAT2	18.00971	12.23754	-5.77217	0.000143
KLF11	6.628196	5.159099	-1.4691	0.000144
OSTM1	3.968545	5.534746	1.5662	0.000144
LGMN	22.84462	28.96978	6.125158	0.000144
RHBDD3	8.692468	10.4215	1.729036	0.000144
REEP6	158.1113	116.3993	-41.712	0.000145
AL354872.	11.37551	6.024642	-5.35087	0.000145
STING1	2.68207	4.448042	1.765971	0.000146
MSN	31.098	42.3003	11.2023	0.000147
CAMKK2	6.996732	8.371521	1.374789	0.000148
FOXS1	1.468606	3.25959	1.790983	0.000148
UBXN2B	7.554949	6.098722	-1.45623	0.000148
PHGDH	6.944499	10.68132	3.736823	0.000149
C15orf48	0.994777	2.50713	1.512352	0.000149
BCL2A1	1.37722	3.049495	1.672275	0.000149
LPXN	3.420289	5.150314	1.730025	0.00015
TRBC2	8.930547	25.47632	16.54577	0.00015
BCL9L	2.142871	3.299385	1.156514	0.000151
SLC12A8	2.603538	3.638635	1.035097	0.000151
GSTM3	2.701587	4.409876	1.708289	0.000151
APP	68.31084	100.7332	32.42238	0.000152
RTCB	25.9043	30.00117	4.096873	0.000153
MPC2	74.48102	88.9299	14.44888	0.000153
LARGE2	2.303978	4.03184	1.727862	0.000154
CDHR5	71.53608	49.70415	-21.8319	0.000154
NUCKS1	49.48293	58.66968	9.186753	0.000155
TUT1	5.119097	6.308702	1.189605	0.000156
PGF	1.100282	2.502821	1.402539	0.000156
MDFIC	3.393059	5.059113	1.666054	0.000156
SGCB	3.405317	5.074517	1.6692	0.000157
POMGNT2	5.006605	6.059923	1.053318	0.000159
LDLR	14.76796	10.59818	-4.16978	0.000159
PLPP1	27.27051	20.38994	-6.88057	0.000159
UAP1	31.73317	25.67054	-6.06263	0.00016
TPR	6.51676	8.362492	1.845732	0.000161
MYO18A	7.091676	5.67189	-1.41979	0.000161
IGHGP	15.93553	62.27324	46.3377	0.000161
S100A2	0.272834	1.824385	1.551551	0.000162
ADCK5	5.169157	6.458643	1.289486	0.000162
MAPK11	3.150888	4.722116	1.571228	0.000162
IRS2	13.61023	10.1171	-3.49313	0.000163
MCMBP	6.228637	7.370357	1.14172	0.000164
SCRIB	14.78899	18.58324	3.79425	0.000165
BRI3	21.84396	28.39253	6.548562	0.000165
LSR	68.18609	88.37657	20.19048	0.000166
MT-ND6	2691.352	1977.63	-713.722	0.000166
EIF1B	16.91737	19.3039	2.386533	0.000166
NECTIN2	46.80688	60.09533	13.28845	0.000166
STMN3	1.926616	4.099891	2.173275	0.000166
RPL10P9	5.566278	17.18857	11.62229	0.000166
WDR82	10.28285	12.11658	1.833736	0.000167
PPP3CA	3.773727	4.993765	1.220037	0.000167
CD2	3.326233	7.088696	3.762463	0.00017

NECTIN4	0.274569	3.789094	3.514525	0.000171
DIO1	52.27628	35.49344	-16.7828	0.000173
SNORD14	1.41029	3.098907	1.688617	0.000173
IRF2BP2	28.96988	35.2061	6.236212	0.000173
SKAP2	3.634811	4.864287	1.229476	0.000173
ANTKMT	6.529103	8.01604	1.486937	0.000174
FNDC10	1.043615	2.532665	1.489049	0.000175
EPDR1	7.547362	10.376	2.828639	0.000175
ZNF706	8.98013	10.82551	1.845385	0.000177
AC120498	3.816435	5.569907	1.753472	0.000177
TCIRG1	17.02469	23.52912	6.504432	0.000177
UBE2G2	8.195849	9.379005	1.183156	0.000178
TTC3	5.771192	7.035691	1.264499	0.000181
DDX58	3.726113	4.923402	1.197289	0.000182
DDB1	13.78402	15.8152	2.031185	0.000183
CPN1	31.50243	21.89908	-9.60335	0.000184
CFD	3.346631	8.08916	4.742529	0.000184
LASP1	29.52787	36.08216	6.554291	0.000187
ARHGAP1	6.074546	7.74145	1.666904	0.000187
MRPL1	10.27851	11.90807	1.629557	0.000187
POLR2F	7.358351	9.33188	1.973529	0.000187
TUBB2B	0.993941	2.719725	1.725783	0.000188
TNPO3	9.89611	11.53093	1.634823	0.000188
AGMAT	25.37476	19.84467	-5.53009	0.000188
AQP1	21.83824	101.5554	79.71712	0.000188
MS4A6A	3.004778	4.761572	1.756794	0.000188
ATP6V1B2	6.25192	7.970744	1.718824	0.000189
CDC37L1	7.346352	6.037465	-1.30889	0.00019
GTSF1	1.397359	3.975742	2.578383	0.00019
IGLC2	42.46876	181.9782	139.5094	0.000193
CHST4	1.182134	8.524495	7.342361	0.000194
FBXO44	6.865535	8.584007	1.718471	0.000194
CD248	4.557903	8.482018	3.924115	0.000194
SLC5A1	0.229128	3.303843	3.074715	0.000195
RPRD2	5.364716	6.730766	1.36605	0.000196
ATP11C	3.984511	2.963637	-1.02087	0.000198
AC008549	8.2658	4.309902	-3.9559	0.000198
CYP26B1	0.399115	1.428814	1.029699	0.000198
EFEMP2	2.330106	3.733959	1.403852	0.000198
AP005233	0.10114	1.726494	1.625354	0.000199
SLC17A1	9.222345	5.608506	-3.61384	0.0002
DUSP1	127.4603	92.26005	-35.2003	0.000204
WARS1	7.423495	13.32097	5.897471	0.000204
VIL1	8.287187	13.3992	5.112009	0.000204
DVL1	14.73293	17.71334	2.980416	0.000204
NFYA	5.389367	6.867131	1.477764	0.000205
WFDC2	3.952981	13.48253	9.529549	0.000206
PLEK	3.099747	5.496703	2.396956	0.000206
SESN2	7.745876	9.752371	2.006495	0.000207
GNS	27.35071	33.79199	6.441274	0.000208
UBE2L6	34.58542	43.99043	9.405008	0.000208
PSPH	7.907407	11.0668	3.159394	0.000209
TTR	1079.291	691.4991	-387.792	0.000209
HSPA1B	18.65517	36.7248	18.06963	0.000209
ACO1	22.50823	17.60217	-4.90607	0.000209
SIGIRR	12.86732	17.90197	5.034652	0.00021
PABPC1	86.16655	112.3428	26.17622	0.000211
CRELD2	9.85407	12.57704	2.72297	0.000211

TC2N	1.543364	3.333225	1.789861	0.000212
PGAP2	8.59323	10.06032	1.467091	0.000212
DBH	2.134411	1.098354	-1.03606	0.000213
AC010547	0.792309	6.490375	5.698067	0.000214
MROH1	3.730147	4.939248	1.209101	0.000214
GNLY	0.786294	2.082594	1.2963	0.000215
CCNL1	5.724854	7.025267	1.300413	0.000218
MTMR11	2.836098	5.386807	2.55071	0.000218
ALDH1L1	41.32276	25.37717	-15.9456	0.000219
PICALM	18.31133	21.65435	3.34302	0.000219
CAB39	9.979796	11.65489	1.67509	0.00022
RRAS2	6.499541	8.282057	1.782516	0.000223
CSTF2T	6.138474	7.325412	1.186938	0.000226
RHEB	15.65903	18.0912	2.432166	0.000227
MRPS14	10.56092	12.11688	1.555961	0.000228
SND1	38.16971	43.75119	5.581486	0.000228
MAP3K14	2.999768	4.584596	1.584827	0.000228
FZD4	5.277628	4.155627	-1.122	0.00023
FBXO21	4.13041	5.412199	1.281789	0.00023
PARP9	9.134526	7.448206	-1.68632	0.00023
ADRA2A	0.636223	1.955837	1.319614	0.000231
DHRS1	14.57561	10.695	-3.88061	0.000234
PNMA8A	0.356716	1.4672	1.110484	0.000235
LSP1	2.057498	4.277183	2.219685	0.000236
TRPV4	1.846701	4.231908	2.385206	0.000237
HOOK1	4.841219	6.200819	1.3596	0.000237
CAMK2N1	32.61902	39.73044	7.111424	0.000238
SIK1B	3.843295	2.579767	-1.26353	0.000239
PF4V1	0.414852	1.776882	1.36203	0.000239
MRPL57	21.56692	25.39525	3.828335	0.00024
ZC3H12A	4.585542	6.478646	1.893103	0.000241
MRPL54	39.18518	48.65588	9.4707	0.000242
MSC	3.836794	11.98617	8.149376	0.000243
BTN3A2	4.779659	6.144442	1.364784	0.000245
CHAD	7.335755	4.894539	-2.44122	0.000247
SRRM3	0.348596	1.497701	1.149106	0.000248
RHPN2	6.94632	9.143412	2.197092	0.000248
CYCS	19.24358	24.04095	4.797376	0.000248
WBP2	31.33983	36.85785	5.518022	0.00025
TMEM167	13.73468	15.86305	2.128375	0.00025
LDHA	80.01324	97.44134	17.42809	0.000252
TIMM13	36.36247	57.20041	20.83794	0.000253
PMVK	89.0107	107.9457	18.93499	0.000254
IGKV3-20	25.19058	119.4535	94.26287	0.000254
HADHB	51.50367	43.39471	-8.10896	0.000254
PUS3	9.878855	8.735843	-1.14301	0.000254
MGST3	10.55755	12.72841	2.170863	0.000257
NUDT16	10.20419	8.689866	-1.51432	0.000257
AL359704.	2.457564	1.014363	-1.4432	0.000258
C1orf43	105.5284	120.179	14.65057	0.000259
CLK1	8.324874	11.405	3.080128	0.000259
TRIM15	5.317347	7.881492	2.564145	0.000264
LRCOL1	3.941611	1.821457	-2.12015	0.000264
BEX2	5.655033	10.51583	4.860796	0.000264
MRPS35	35.01946	41.7983	6.77884	0.000265
SRP14P4	7.883132	5.3732	-2.50993	0.000265
ODF3B	8.333848	11.25404	2.920195	0.000266
OXTR	0.259812	2.12703	1.867217	0.000266

LACTB	8.932398	7.780986	-1.15141	0.000266
TPM1	7.190111	9.990614	2.800503	0.000268
UCHL1	1.764873	13.94929	12.18442	0.000268
CTH	42.80853	15.44995	-27.3586	0.000268
ORC3	4.953304	6.101909	1.148604	0.000269
KLHDC8B	8.67711	11.68277	3.005665	0.000269
ARCN1	26.92563	31.21268	4.287053	0.00027
TIMP3	37.63547	29.41554	-8.21993	0.000274
AC016885	0.605452	1.722168	1.116716	0.000274
KIFAP3	5.569419	6.995558	1.426139	0.000274
CDC42EP5	1.883903	2.947592	1.063689	0.000275
MMRN2	5.156668	4.0225	-1.13417	0.000275
LCAL1	0.40176	2.291355	1.889595	0.000276
EFR3A	14.68359	11.84937	-2.83421	0.000278
SLC39A4	2.927192	6.61409	3.686898	0.000279
AL513165.	3.791759	4.886572	1.094813	0.00028
TNC	1.683159	7.100486	5.417327	0.00028
PDK4	45.17655	21.49514	-23.6814	0.00028
MAB21L2	0.261377	1.405921	1.144544	0.000281
RUSF1	10.92238	8.958952	-1.96343	0.000283
ALDH1B1	48.03457	34.71108	-13.3235	0.000285
TSPAN4	8.881532	11.26663	2.385102	0.000288
MRS2	4.803971	6.690227	1.886255	0.000293
IGLC3	26.91686	103.0581	76.14127	0.000293
CCM2	8.042738	9.353773	1.311034	0.000293
FOLR2	7.933395	13.65945	5.726053	0.000294
IGHG3	15.24108	53.0446	37.80352	0.000294
BORCS7	18.36615	15.06386	-3.30229	0.000295
PSMD5	5.288653	6.292287	1.003634	0.000296
PLEKHA6	6.475557	8.538867	2.063309	0.000297
OSGIN2	5.249938	6.522433	1.272496	0.0003
TMSB4XP8	5.034351	12.07859	7.044235	0.0003
REC8	0.720789	2.631486	1.910698	0.000302
MEAF6	7.578203	9.197032	1.618829	0.000304
MPV17L2	7.586511	9.061694	1.475183	0.000306
AL360181.	3.37604	2.053361	-1.32268	0.000306
MAP4K4	9.345475	11.87661	2.531134	0.000311
UCA1	0.232296	4.022383	3.790087	0.000311
RPL10P6	3.709856	12.77642	9.066563	0.000314
MRFAP1L1	23.7496	26.92712	3.177526	0.000319
YIPF2	11.30359	13.40543	2.101842	0.00032
CAVIN3	1.055214	2.608885	1.553671	0.000321
P3H2	0.501862	1.513163	1.011301	0.000321
ADD1	10.84832	12.932	2.083676	0.000323
MXRA7	2.59439	3.654863	1.060473	0.000324
MIR4482	3.57245	2.04431	-1.52814	0.00033
BX571818	0.838068	2.790117	1.952049	0.000331
NDUFA4	61.72541	72.43824	10.71283	0.000331
SUN2	16.32746	11.37183	-4.95563	0.000332
ALCAM	14.61941	19.91885	5.299439	0.000335
PXDN	2.260503	3.874246	1.613743	0.000336
SERINC5	12.89407	10.2373	-2.65676	0.000339
DPYSL3	2.126267	4.387912	2.261645	0.000339
JAK1	9.398073	11.63048	2.232403	0.000343
SLC3A2	20.91116	25.59869	4.687534	0.000343
EPHB3	0.327011	2.479812	2.1528	0.000348
SLC20A2	9.942987	7.622959	-2.32003	0.00035
HINT1	45.20178	52.24859	7.046804	0.000351

NDUFS4	39.09872	46.68685	7.588132	0.000352
ARL14	0.644516	5.538554	4.894038	0.000352
ETS2	36.08207	28.81793	-7.26414	0.000355
AC020978	2.913436	1.433934	-1.4795	0.000361
IGLV1-44	7.513593	31.20355	23.68995	0.000365
SYTL1	0.447868	1.493983	1.046115	0.000366
MUC1	0.365951	8.638859	8.272907	0.000368
COMP	1.020747	3.738983	2.718236	0.000369
HEPACAM	3.516149	0.859513	-2.65664	0.000369
LRIG1	5.391506	4.221764	-1.16974	0.00037
RAI2	1.554133	2.879171	1.325039	0.000371
FGL1	378.8435	242.9011	-135.942	0.000372
CYP2B6	37.36974	18.58713	-18.7826	0.000372
ZNF395	4.115903	5.220938	1.105035	0.000376
MPRIP	3.527891	4.584285	1.056394	0.000377
PLGRKT	8.307357	10.05726	1.749901	0.000377
CRABP2	0.792076	4.384313	3.592237	0.000379
YY1AP1	5.737972	7.112311	1.374339	0.00038
MEGF8	3.563255	4.688846	1.125592	0.000384
FBLN5	6.607862	4.145192	-2.46267	0.000384
PPARGC1/	6.401353	4.10691	-2.29444	0.000384
SULF2	7.421971	12.27715	4.855177	0.000385
UNC5B	2.238674	3.634112	1.395437	0.000387
CPT1A	32.30609	21.76671	-10.5394	0.000388
CTSD	134.7548	170.6515	35.89674	0.000389
ACTN1	12.66271	16.69302	4.030313	0.00039
NUCB2	7.107331	9.029654	1.922323	0.00039
TRAC	9.430215	21.58117	12.15096	0.00039
BATF	4.001656	7.980736	3.979081	0.000391
WDR72	2.527728	4.965769	2.438041	0.0004
CYB5B	7.439407	9.6472	2.207792	0.000412
SLC25A11	25.34498	29.59212	4.247142	0.000413
AMBP	3076.568	2438.756	-637.811	0.000413
SRPX2	0.507008	1.953136	1.446128	0.000414
TMEM41B	10.53533	12.0724	1.537071	0.000414
SLC30A10	14.62831	11.22244	-3.40587	0.000415
EEPD1	5.783086	7.427387	1.644301	0.000415
SLC39A9	13.59587	11.94397	-1.6519	0.000416
SNX3	62.0152	71.43771	9.422506	0.000417
SDCBP2	4.554206	8.452081	3.897875	0.00043
MINCR	2.471324	3.978492	1.507168	0.000434
NPTX2	1.919225	9.933303	8.014078	0.000444
COL4A2	12.61916	17.95603	5.336875	0.000447
PLEKHH2	0.161367	1.167092	1.005725	0.00045
LINC0155!	0.038911	1.252435	1.213524	0.00045
SUSD4	3.272833	5.476438	2.203605	0.000453
EIF4EBP2	32.30677	27.07829	-5.22848	0.000453
ZBTB33	8.189761	6.576722	-1.61304	0.000456
FURIN	118.7593	89.49157	-29.2677	0.000463
RAPGEF1	8.1952	9.761931	1.56673	0.000463
RAB11FIP1	1.608104	2.931399	1.323295	0.000466
ARHGAP4	3.509721	6.040648	2.530928	0.000466
IFITM2	106.8469	137.2998	30.45293	0.000466
KCNQ1	1.329557	4.584251	3.254695	0.000467
GSTK1	70.93077	60.79145	-10.1393	0.00047
CD7	1.718493	7.575309	5.856816	0.000471
MROH6	2.161027	3.513648	1.352621	0.000474
STEAP3	26.33127	18.16165	-8.16962	0.000475

TBX3	11.89363	7.091888	-4.80174	0.000477
CDV3	28.00821	32.07965	4.071438	0.000479
COL1A1	48.9162	90.36108	41.44488	0.000481
SCD	422.0716	274.0068	-148.065	0.000485
AKR1B1	6.524593	12.39594	5.87135	0.000485
PRF1	2.209721	3.658247	1.448527	0.000488
DNAJB1	13.44075	18.60769	5.166937	0.000488
PRSS23	2.610498	4.497432	1.886934	0.000489
CRCP	9.212438	10.60457	1.392136	0.000489
PMAIP1	0.298179	1.641177	1.342998	0.00049
AARS1	16.135	18.86714	2.732134	0.00049
KCNS3	1.607786	2.943647	1.335861	0.000492
PALM3	7.158534	11.16303	4.004494	0.000492
SLC20A1	7.829245	9.8539	2.024655	0.000493
ABCG5	12.58833	9.400949	-3.18738	0.000494
AASS	9.367285	5.426372	-3.94091	0.000497
TCTN3	10.86581	12.41123	1.54542	0.000498
RAB11B	41.47989	47.30401	5.82412	0.0005
SLC1A7	0.937242	3.516936	2.579694	0.000501
KIF3B	10.69378	12.75053	2.056751	0.000503
SFRP5	3.946227	28.37777	24.43154	0.000505
AC006329	3.538392	2.218502	-1.31989	0.00051
SON	8.248194	9.857911	1.609718	0.00051
B3GNT7	0.725516	3.708776	2.983261	0.000511
MUC20	1.221288	2.997741	1.776453	0.000513
ECE1	14.90234	18.47815	3.575806	0.000524
GBE1	15.68244	12.27272	-3.40972	0.000524
Z97056.1	2.92996	1.523209	-1.40675	0.000527
MRM3	6.228343	7.762853	1.53451	0.000527
SMARCC2	4.346715	5.500212	1.153497	0.000527
POLRMT	14.04783	16.18117	2.133338	0.000529
SLC7A8	0.814017	1.820964	1.006947	0.000537
FNBP1	5.012147	6.233081	1.220934	0.000538
CLU	691.8914	553.6168	-138.275	0.00054
CCL2	6.751566	14.72045	7.968885	0.000543
KMT2E-AS	5.187281	7.331205	2.143924	0.000544
EDN1	1.340107	2.630658	1.29055	0.000551
ANXA1	5.240833	10.64076	5.399928	0.000552
DNAJB4	3.403304	4.674936	1.271632	0.000553
CHCHD4	8.404078	9.535876	1.131798	0.000555
IGFBP6	1.992476	5.763483	3.771007	0.000556
LMAN2	153.8532	176.7535	22.90035	0.000557
TMPO	8.951828	10.87279	1.920957	0.000559
AVPI1	19.02932	15.10611	-3.92321	0.000574
MXI1	7.191227	6.091964	-1.09926	0.000575
MBTPS1	7.074543	8.346788	1.272244	0.000575
SRP72	11.1053	12.74337	1.638066	0.000576
CYC1	96.64935	120.0829	23.43357	0.000576
IGKV3-11	10.21896	52.02724	41.80828	0.000577
YY1	9.570597	10.67115	1.100553	0.000579
TNFRSF11	2.388121	7.908261	5.52014	0.00058
MXRA8	3.351733	8.744732	5.392999	0.000581
LZTS3	3.699788	5.331311	1.631523	0.000582
TXN2	56.82727	64.07885	7.251577	0.000588
HSPB8	4.777876	8.987726	4.20985	0.000594
AIFM2	8.705658	10.67862	1.972957	0.000599
GABRE	1.924799	3.006779	1.081981	0.000602
CCND2P1	10.15603	6.000946	-4.15509	0.000607

SLC4A3	0.328458	3.316068	2.987611	0.000607
ADAR	29.47719	35.31339	5.836197	0.000607
STAT1	21.38034	32.77319	11.39285	0.000617
C2CD4A	1.116932	5.035489	3.918557	0.000619
LRRC75B	6.387015	9.086186	2.699171	0.00062
ANKS4B	5.864909	4.699391	-1.16552	0.000625
ST3GAL4	3.555511	4.845759	1.290248	0.000625
FBXO31	10.68341	7.74104	-2.94237	0.000627
ARHGAP3	1.973638	3.45328	1.479642	0.000628
GSDMD	29.80188	35.97241	6.170522	0.000632
SOX13	6.425872	7.972942	1.54707	0.000633
CD53	6.303823	11.82949	5.525671	0.000634
FAM83H	16.48788	20.95138	4.463502	0.00064
AGL	4.699932	3.581955	-1.11798	0.000641
PEBP4	1.622398	0.218402	-1.404	0.000645
CYFIP1	7.854702	9.167453	1.312751	0.000645
LAP3	35.7237	28.69189	-7.03182	0.00065
AP006222	0.825217	2.72922	1.904003	0.000654
CFHR3	36.66286	20.84099	-15.8219	0.000665
SORD2P	5.696523	2.994592	-2.70193	0.000666
HDLBP	44.40002	50.00177	5.601756	0.000668
ARSF	1.692489	0.565513	-1.12698	0.000675
NPTXR	1.056287	2.121879	1.065592	0.00068
GAS7	0.563997	2.422127	1.85813	0.000682
BATF2	1.715022	3.15324	1.438218	0.000684
HLA-B	432.1819	587.5636	155.3818	0.000689
ABCA1	6.788966	5.378412	-1.41055	0.000692
GGT6	0.469642	2.609116	2.139474	0.000692
AP1G2	0.970012	2.584985	1.614972	0.000694
PHYHD1	5.889962	3.830806	-2.05916	0.000697
GZMM	1.266777	2.434962	1.168185	0.000707
PAQR8	1.278314	2.516463	1.238149	0.000709
MAGEA6	2.92163	6.60641	3.684781	0.000711
RNF144B	3.928735	2.918977	-1.00976	0.000713
CXCL5	0.96155	6.281495	5.319944	0.000719
TBC1D17	10.13621	11.59963	1.463422	0.00072
NR1D2	6.4266	5.398245	-1.02836	0.000721
DCAF8	7.273095	8.360664	1.087568	0.000722
SELENOT	14.67791	16.45698	1.779071	0.000724
LRPPRC	12.83106	14.75171	1.920649	0.000726
MRPS27	8.826065	10.06898	1.242912	0.000726
JCHAIN	6.712698	19.20458	12.49189	0.000728
IGKV3D-1	0.320142	1.479617	1.159475	0.000734
MVD	12.37633	17.83829	5.461962	0.000735
MROH2A	1.917256	0.506059	-1.4112	0.000735
SLCO1B3	5.785655	2.847277	-2.93838	0.000736
WFDC21P	0.4603	1.821742	1.361442	0.000739
AC018638	6.141275	7.867907	1.726632	0.000743
CGGBP1	7.954557	9.108302	1.153745	0.000743
GON7	7.96029	9.15627	1.19598	0.000744
IGLV3-21	8.808989	37.10996	28.30097	0.000749
SRPRA	50.80171	44.61853	-6.18318	0.000751
TMEM63A	6.145454	8.389186	2.243732	0.000751
FASN	95.07232	66.64211	-28.4302	0.000752
HMGB3	14.19722	18.83379	4.63657	0.000752
GHITM	90.2813	80.08449	-10.1968	0.000753
LOXL4	7.867765	13.63929	5.771523	0.000754
DOK4	9.192277	11.84186	2.649585	0.000759

DBI	73.10913	86.33492	13.22579	0.000759
HLA-F	17.72758	25.04892	7.321333	0.000766
CALM1	20.54157	23.41743	2.875856	0.000773
UGT3A1	8.042769	5.114919	-2.92785	0.000774
FOXO1	4.573724	3.45421	-1.11951	0.000775
ALS2CL	3.339546	5.009415	1.669869	0.000778
SIVA1	10.85666	12.99949	2.142826	0.000778
HGH1	16.38143	20.39637	4.014944	0.000784
GPR37	4.132315	2.805815	-1.3265	0.000787
MRPS10	20.27411	23.11295	2.838835	0.000799
ANTXR1	2.347374	4.085162	1.737787	0.000807
ADORA1	0.539969	2.222626	1.682657	0.000808
STK19B	3.262584	6.335988	3.073404	0.000811
TFRC	10.55845	13.51547	2.957012	0.000813
COL5A2	6.691298	10.56095	3.869655	0.000816
ATP6V1C1	12.1875	14.81783	2.630322	0.000829
ARSI	0.256268	1.61091	1.354641	0.000832
PHRF1	8.344033	9.528941	1.184908	0.000839
PTPRK	7.527436	9.268945	1.741509	0.000841
FUT6	2.776284	4.877711	2.101427	0.000842
WDR26	8.782658	10.39068	1.608022	0.000844
TBL1XR1	5.584978	8.382143	2.797165	0.000847
CDKN1C	2.088046	5.863736	3.77569	0.000852
ALDH16A1	6.308076	8.076358	1.768281	0.000853
MED11	6.114041	7.217893	1.103852	0.000862
IL13RA1	35.92148	30.23484	-5.68665	0.000864
GINM1	10.71997	12.20481	1.484847	0.000865
POLR2A	10.56358	13.06835	2.504771	0.000866
NEK6	14.51017	17.13957	2.629394	0.000871
MYO1D	6.755795	9.171657	2.415863	0.000876
PRAMEF1(	2.127533	0.662852	-1.46468	0.000878
IGF2BP2	3.094496	4.922558	1.828062	0.000887
PLXNB2	46.61913	60.34164	13.7225	0.000888
ZFR	8.523325	9.990244	1.46692	0.000892
NAMPT	20.09036	14.99461	-5.09575	0.000894
MAPK8IP2	1.566375	2.59357	1.027196	0.000899
AC009407	9.43873	13.13165	3.692919	0.000902
SNTB1	22.43463	18.30359	-4.13104	0.000903
TSTD1	21.73175	26.72941	4.997664	0.000903
EFNA1	134.6725	170.1346	35.46206	0.000904
FGFRL1	15.80752	12.91309	-2.89444	0.000904
YWHAG	39.60839	45.96656	6.358177	0.000907
PRODH2	31.81565	23.46548	-8.35017	0.000908
CTR9	11.0948	9.507917	-1.58688	0.000913
ZFAND2A	14.9864	19.31666	4.330258	0.000916
GSTM1	16.21407	6.586711	-9.62736	0.000924
VPS4B	7.16078	8.35477	1.19399	0.000928
FAM168B	15.69038	18.54362	2.853238	0.000933
HECTD1	8.470097	7.241726	-1.22837	0.000937
PUM2	13.26571	15.20931	1.9436	0.000938
IGLV9-49	0.629905	2.747645	2.117739	0.000939
ABLIM1	6.791159	9.941959	3.1508	0.000947
BIRC2	9.234051	10.73222	1.498167	0.000948
RAB7A	26.78602	29.81879	3.032772	0.000952
CLTRN	1.705597	4.894338	3.188742	0.000956
GSN	16.00865	20.51565	4.507001	0.000962
GLUD1	142.7153	104.2592	-38.4561	0.000971
RGS14	14.31378	17.85607	3.542292	0.000971

TRIM31	1.975883	3.826172	1.850289	0.000972
CAMSAP3	8.438314	10.0641	1.625788	0.000973
SLC2A3	1.994412	3.478567	1.484154	0.000973
RTN4RL1	1.9967	3.173165	1.176465	0.000976
CTAG2	2.871553	9.098024	6.22647	0.000977
MAMDC4	7.207293	3.883452	-3.32384	0.000986
AC079466	2.071473	6.728266	4.656792	0.000986
IL1R2	3.08069	4.428828	1.348138	0.000993
F3	1.051441	3.325432	2.273991	0.000997
SLC16A11	13.65754	7.164006	-6.49353	0.000998
TOM1L1	5.663678	4.603895	-1.05978	0.000998
SCPEP1	10.77051	16.1646	5.394085	0.001002
COL5A1	5.263092	8.996949	3.733857	0.001008
MIR3189	1.352375	4.1374	2.785025	0.00101
NMRAL2P	1.724314	2.873538	1.149225	0.001012
NIPSNAP3	8.659437	7.321406	-1.33803	0.001012
SLC12A7	23.107	28.11981	5.01281	0.001015
CSRP1	11.35261	14.2877	2.935095	0.001019
ADM2	3.802012	5.329715	1.527703	0.001023
ECM1	2.455768	4.007743	1.551974	0.001026
ICAM1	13.46576	19.47208	6.006324	0.001026
GPSM1	2.45956	3.608804	1.149245	0.00103
DKK1	3.308404	15.92829	12.61989	0.001034
PDZD8	3.942961	5.452179	1.509218	0.001038
HOXB9	0.031779	1.042967	1.011188	0.00104
LIN7A	4.971041	3.928517	-1.04252	0.001049
TMCO3	4.078879	5.45304	1.374161	0.00105
SSX1	5.333667	11.47542	6.141755	0.001056
TRMT10C	11.06643	12.52226	1.455828	0.001056
PLOD1	41.36212	51.45869	10.09657	0.001061
TECRP1	2.279739	3.79223	1.512491	0.001065
DNAJC1	18.71305	21.74665	3.033595	0.001072
DCBLD2	1.858832	3.20007	1.341238	0.001074
DEGS2	0.33961	1.348323	1.008713	0.001079
PDS5A	6.135879	7.20375	1.067871	0.001079
BPNT2	18.12556	14.29079	-3.83476	0.001087
PDCD4	8.141902	10.85816	2.716254	0.001105
TIMP1	171.9838	373.783	201.7993	0.001108
MT-CO1	11854.47	9753.937	-2100.53	0.001111
GOLGA4	8.198595	6.950111	-1.24848	0.001113
RNASE1	40.04571	74.30452	34.25881	0.001118
PLTP	8.660966	21.02102	12.36005	0.001128
FHL2	1.741226	2.940107	1.198882	0.001132
CD207	0.15843	1.390004	1.231574	0.001133
RBL2	5.673388	4.568184	-1.1052	0.00114
PI3	2.58576	24.95502	22.36926	0.001142
ZNF513	6.991477	8.041476	1.049999	0.001152
TRBC1	5.122547	13.934	8.811451	0.001153
PPP1R1B	0.136388	1.997075	1.860687	0.001154
HBEGF	1.994307	3.318849	1.324542	0.001161
RNF167	34.18067	38.41679	4.236113	0.001162
CLIC2	1.941908	3.05046	1.108552	0.001165
CYB5D2	11.22385	9.373933	-1.84991	0.001166
BIK	1.755112	3.104578	1.349466	0.001175
CYP1A2	41.02297	12.65324	-28.3697	0.001177
RGS4	0.692953	5.458503	4.76555	0.001179
MBD6	6.460449	8.173478	1.713029	0.001184
EVI2B	2.052548	3.903939	1.851391	0.001186

SHC2	18.0426	22.63324	4.590642	0.001189
ASPG	5.426936	3.401534	-2.0254	0.001195
GBP4	3.912211	5.784307	1.872097	0.001204
PEX19	23.47025	20.6137	-2.85656	0.001208
TLN1	23.28088	27.68979	4.408913	0.001212
ARHGEF26	4.944439	3.740367	-1.20407	0.001217
TMEM176	227.7797	185.5504	-42.2293	0.001222
TBX15	4.372272	2.772974	-1.5993	0.001225
PRPS2	8.641628	10.68208	2.040448	0.001234
UPP2	5.21047	1.794574	-3.4159	0.001237
ARHGAP3	10.21963	8.943044	-1.27659	0.001242
PRDX4	62.39226	74.3032	11.91093	0.001255
CD99L2	17.0188	14.11444	-2.90436	0.001261
NDUFB7	169.4661	265.3532	95.88709	0.001283
SHROOM3	2.125768	3.212408	1.08664	0.001287
FBXO6	10.19066	12.72604	2.535383	0.001305
HS3ST1	0.212507	1.276858	1.06435	0.001308
GREM2	5.635135	2.952365	-2.68277	0.001321
PRKAB2	16.2115	13.18612	-3.02538	0.001337
LYPLAL1	9.847238	11.64707	1.799827	0.001337
CHI3L1	211.7379	108.4616	-103.276	0.00135
ISLR	4.417048	10.96457	6.54752	0.001352
FMOD	4.325743	10.02838	5.70264	0.001356
ELK1	8.574558	9.772258	1.1977	0.001362
UBAP1	18.16431	20.82715	2.66284	0.001366
C2orf72	46.55148	38.57444	-7.97704	0.001367
CAP2	11.26212	8.45265	-2.80947	0.001375
KISS1	2.533685	4.100533	1.566848	0.001382
CA9	5.879495	35.79102	29.91153	0.001385
MAGEA3	3.883634	8.048777	4.165143	0.001388
TOR1A	8.797586	9.848989	1.051402	0.00139
ERG28	38.60062	32.19754	-6.40308	0.001406
ABHD2	23.79609	18.66349	-5.1326	0.001407
DCXR	362.5791	246.2114	-116.368	0.00141
SBF1	5.545928	6.737608	1.19168	0.001417
IPO7	12.3622	14.6617	2.299503	0.001421
ACVR1B	8.648289	10.49865	1.850364	0.001423
FCN2	2.938712	1.15054	-1.78817	0.001428
IGKV2-30	1.336682	5.777746	4.441064	0.001438
CELF1	9.286402	10.28827	1.00187	0.001441
C2CD4B	0.367532	1.606181	1.238649	0.001455
COL6A3	4.020069	7.64761	3.627541	0.001457
PARP3	6.406729	7.745809	1.339081	0.001468
KDELR2	81.99472	91.29328	9.298558	0.001474
SLC9A3-A	1.833991	3.548629	1.714637	0.001474
CYTIP	1.079046	2.265704	1.186658	0.001479
MOSPD1	3.639425	4.716128	1.076702	0.00148
TSPYL5	0.508351	1.653369	1.145019	0.001486
RRAD	1.451151	3.906296	2.455145	0.001488
IGLV7-43	1.096997	5.348197	4.2512	0.001494
LARP1	19.57795	22.48034	2.902395	0.001494
DDRGK1	50.66741	58.161	7.493585	0.001495
RNF208	7.454994	9.173059	1.718066	0.001508
MBD4	8.688976	9.953753	1.264777	0.001522
AMFR	27.45075	23.94379	-3.50696	0.001527
HNRNPH2	28.74437	32.23349	3.489122	0.00153
HSPA13	4.919128	5.992151	1.073023	0.001533
COL27A1	4.009533	5.463401	1.453868	0.001533

AHCYL1	21.12183	24.50625	3.384412	0.00154
UQCRQ	74.6205	94.5352	19.9147	0.00155
DNAJC25	7.483256	6.246524	-1.23673	0.001556
TRAF7	13.8875	16.05043	2.162929	0.001561
MAP2K1	10.28351	11.88914	1.605629	0.00157
RDH11	20.00891	16.93099	-3.07792	0.001574
ENC1	2.913198	4.495642	1.582444	0.001578
HLA-E	206.0688	252.6928	46.62403	0.001581
H3P6	5.099345	6.264363	1.165018	0.001584
INSR	15.84884	13.60285	-2.24599	0.001585
CHSY1	2.695477	4.055995	1.360518	0.001589
TBCA	30.34062	35.44008	5.099461	0.001593
ABHD8	6.830912	7.97331	1.142398	0.001594
TMCO1	31.41589	35.68213	4.266245	0.001601
TF	263.0396	201.7101	-61.3294	0.001607
SLC19A2	8.620902	6.496698	-2.1242	0.001608
DUSP9	6.399148	15.44959	9.05044	0.001608
JUNB	44.40815	59.749	15.34085	0.001618
BMP2	3.704858	5.749099	2.044241	0.00163
MIR6772	1.059362	2.083881	1.024519	0.001637
FUOM	67.45815	53.61802	-13.8401	0.001639
PSMD12	10.63692	11.81251	1.17559	0.001643
VWF	9.485796	7.05592	-2.42988	0.00165
MMP9	3.744628	10.59987	6.855239	0.001657
ASCC2	13.77211	15.21204	1.439932	0.001662
AFDN	6.441934	7.90835	1.466416	0.001662
IGHV3-23	11.20805	39.60377	28.39573	0.001669
TMEM150	6.383886	4.521557	-1.86233	0.001669
CRYAB	3.537167	15.01488	11.47772	0.001689
EEF1A1P5	9.38194	14.65994	5.277997	0.001699
CLTC	27.42988	31.53058	4.100693	0.001725
MCFD2	26.76179	23.0757	-3.68609	0.001728
TMEM171	2.514502	4.352217	1.837715	0.001729
GPN3	7.621513	8.715916	1.094403	0.001731
OBSL1	5.90922	9.094836	3.185616	0.001757
PLXNB1	12.66062	15.0403	2.379679	0.001765
FAM99B	2.341048	1.172057	-1.16899	0.00177
CTDSP2	22.45438	26.75549	4.301111	0.00177
TDGF1	3.870234	2.116047	-1.75419	0.001793
SDHC	57.46427	50.45943	-7.00484	0.001831
MOCOS	6.027083	5.012977	-1.01411	0.001835
CXCL9	9.119133	19.23282	10.11369	0.001842
PCSK1N	1.643672	11.72936	10.08569	0.001842
LITAF	29.72867	35.52225	5.793577	0.001845
GOLIM4	16.74897	13.63147	-3.1175	0.001846
CMPK1	44.27845	50.07041	5.791957	0.001848
ALKAL2	0.613397	2.591434	1.978038	0.001862
TGFBR2	14.2883	11.80847	-2.47983	0.001866
PLAAT3	14.14072	18.2137	4.072977	0.001877
SKAP1	2.66351	3.898901	1.235391	0.001878
CORO1A	3.638885	9.237718	5.598833	0.001882
LYN	4.929972	5.967388	1.037416	0.001883
INPP5D	1.2836	2.32923	1.045629	0.001901
ARHGDIA	37.60355	43.84535	6.2418	0.001906
TUBA3C	0.691157	4.697923	4.006766	0.001922
PLBD2	15.3674	17.87365	2.506255	0.001926
RAD23A	29.49519	32.94376	3.448572	0.001947
LTBP1	3.042464	5.137185	2.094721	0.001947

RPL12P14	2.915068	1.83313	-1.08194	0.001961
TPPP3	1.689807	8.723724	7.033917	0.001976
HSPA7	1.111517	3.651074	2.539557	0.001978
SLC25A44	10.53843	9.084882	-1.45355	0.001979
SERPINI1	2.362246	5.347742	2.985497	0.002012
CSRNP1	10.36579	8.204778	-2.16102	0.002023
S100A9	14.60994	85.71086	71.10091	0.002029
CSAG1	2.696718	6.6059	3.909182	0.002035
PLOD2	10.36322	13.33414	2.970916	0.002052
CFTR	0.841867	6.774187	5.93232	0.002063
KEAP1	22.09027	24.50779	2.417513	0.002069
EBP	48.54819	63.83075	15.28256	0.002078
ANXA6	45.17201	35.87134	-9.30067	0.002083
GLUL	337.8657	181.0194	-156.846	0.00209
NUMA1	5.461994	6.488593	1.0266	0.002092
CTSA	23.43618	27.82198	4.3858	0.002095
AC003093	0.095254	2.434115	2.338861	0.002097
NRBP2	19.28131	14.65332	-4.628	0.002098
PEX11B	18.06436	20.45215	2.387785	0.002102
TMBIM1	37.30572	43.13406	5.828346	0.002121
ARID3A	2.176668	3.724782	1.548114	0.002129
CROT	8.004413	6.297763	-1.70665	0.002135
COX7B2	4.9763	9.31226	4.33596	0.002152
DCTD	14.4451	16.81239	2.367291	0.002159
HIP1	4.734018	5.821171	1.087153	0.002167
ETFB	13.85785	19.45747	5.599627	0.002177
BMERB1	4.984635	3.774301	-1.21033	0.002195
LAMP1	79.61568	69.24151	-10.3742	0.002208
CDK18	4.124905	5.706825	1.581919	0.002211
TAF9B	6.143752	7.398188	1.254436	0.002249
SMIM32	2.026957	6.009889	3.982932	0.002262
ACHE	1.320277	2.504755	1.184477	0.002279
GJA1	4.489516	8.549866	4.060349	0.002284
SMIM3	2.704675	4.602593	1.897917	0.002288
SHOC2	6.628456	7.640187	1.011731	0.002288
HNRNPF	56.51355	63.55588	7.042337	0.002307
IGHV3-21	7.3356	25.10709	17.77149	0.002308
STAB1	4.048709	5.728336	1.679627	0.002354
FCAMR	3.254989	1.600016	-1.65497	0.002357
TPSP2	2.350873	5.248675	2.897802	0.002402
LIMA1	4.275299	5.594349	1.319051	0.002404
APOL2	20.92542	24.90336	3.977934	0.002445
UBXN10	2.88746	1.824191	-1.06327	0.002449
FRG1FP	0.852039	1.941607	1.089568	0.002462
SDF4	32.78763	36.81173	4.024102	0.002473
ANXA9	29.338	42.47175	13.13375	0.002479
ARAF	21.19394	23.8203	2.626365	0.002481
LTBP3	9.321556	11.93941	2.617854	0.002484
AP006621	4.056797	5.168841	1.112044	0.002485
IGKV2-29	0.547652	3.236277	2.688625	0.002491
CLDN12	9.086435	7.723413	-1.36302	0.002502
CXCR4	10.63901	23.58285	12.94384	0.002509
SPARCL1	62.30456	30.08325	-32.2213	0.002513
TAGLN	7.243338	10.26831	3.024975	0.002517
C7	16.76355	9.308698	-7.45485	0.002531
IFIT1	12.25046	7.539418	-4.71105	0.002534
CEP170B	5.174494	6.210068	1.035574	0.002534
ZDHHC6	7.747655	8.802214	1.054559	0.002538

GOLGA5	17.41316	15.68109	-1.73207	0.002539
LHPP	11.96181	9.784898	-2.17691	0.002544
C1orf115	54.95485	46.8347	-8.12015	0.002617
GPER1	7.569713	5.600656	-1.96906	0.002619
OXCT1	1.077107	2.378027	1.30092	0.00263
SMC3	6.812066	8.185357	1.373291	0.002645
DHCR7	57.38888	44.39258	-12.9963	0.002672
METTL27	2.695922	3.756273	1.060352	0.002678
TGFB3	1.872321	3.234913	1.362592	0.002697
CENPV	6.869256	8.493977	1.624721	0.002703
RRS1	11.66269	14.18666	2.523968	0.002729
IGHV1-18	8.148947	26.78909	18.64015	0.002746
SLC25A28	17.02372	19.16064	2.136914	0.002749
HLA-DRB5	50.12621	79.03717	28.91097	0.002782
RFNG	16.74846	19.45649	2.708035	0.00279
ZNF687	6.322162	7.645021	1.322859	0.002808
SGK1	7.413296	5.554193	-1.8591	0.002812
TOLLIP	25.09641	22.57068	-2.52573	0.002842
CTNNAL1	7.761539	9.970694	2.209155	0.002868
SLC5A6	21.08946	14.39515	-6.69431	0.002891
VNN2	4.498698	8.224482	3.725784	0.002904
NOTCH3	5.64546	8.2372	2.59174	0.002911
P4HA1	25.32163	19.62817	-5.69346	0.002915
KCNH3	0.126929	1.321534	1.194605	0.002923
DDB2	5.645702	6.946446	1.300744	0.002947
PON2	39.64154	47.97886	8.337321	0.002948
BCAT2	6.678669	8.472267	1.793598	0.002951
SURF1	32.51204	36.65323	4.141189	0.002976
PPP1R3G	8.015526	4.576383	-3.43914	0.002994
CAPN8	0.164396	1.338878	1.174482	0.003013
ZFP91	8.077705	9.295726	1.218021	0.003015
PAPOLA	8.676548	9.709984	1.033435	0.00305
APOA1	4732.788	3500.857	-1231.93	0.003083
TSPAN7	3.081893	4.253084	1.171191	0.003111
ACTR1B	20.50089	23.04403	2.543143	0.003134
TSPAN1	1.607371	5.385064	3.777693	0.003134
RETREG1	4.12235	2.630486	-1.49186	0.003148
RHOBTB3	6.900783	5.589926	-1.31086	0.00318
GIMAP4	7.826136	10.56501	2.738874	0.003186
LCK	1.227767	2.620563	1.392796	0.003226
IGLV2-18	0.6214	2.339617	1.718217	0.003245
ASS1	307.6596	202.9848	-104.675	0.003257
NDFIP1	33.38729	30.44408	-2.94321	0.003261
ATP1B1	102.1044	145.7874	43.68299	0.003263
SOCS1	2.256276	3.820913	1.564636	0.003267
CHCHD2P	5.84598	7.002612	1.156633	0.003268
FNDC5	12.44813	6.491974	-5.95615	0.003276
DPAGT1	8.500887	9.531537	1.03065	0.003354
EIF1AX	12.22478	13.78341	1.558629	0.003359
IDI1	34.57953	28.07174	-6.50779	0.003377
NFKBIA	50.28602	60.8641	10.57808	0.003432
CBR4	6.573625	5.520063	-1.05356	0.003433
NIT1	14.3795	12.73195	-1.64755	0.003451
POLR3GL	10.76076	12.4444	1.683634	0.003475
SHANK3	5.094572	4.000698	-1.09387	0.003484
CLIP3	0.970911	2.221792	1.250881	0.003495
KCTD12	3.647239	5.088146	1.440907	0.003501
CYP2A7	36.64683	12.61657	-24.0303	0.003532

RIOK3	12.71286	14.51095	1.798094	0.003542
KDM2A	7.965906	9.204444	1.238538	0.003555
PACSIN2	19.87977	24.03013	4.150352	0.003557
KCNN4	0.325132	1.577212	1.25208	0.00357
SPARC	92.09731	120.1379	28.04063	0.003584
TTYH1	0.385319	2.455019	2.0697	0.003585
PSPHP1	20.88398	29.05024	8.166259	0.003609
SLC13A3	14.68116	7.513361	-7.1678	0.003609
ASAH1	4.768151	7.440917	2.672766	0.00361
BTN3A1	5.55196	7.003305	1.451345	0.003611
VNN1	40.83185	27.39149	-13.4404	0.003668
ATP11A	3.269352	4.406571	1.137219	0.00368
RBBP9	8.679804	7.4511	-1.2287	0.003685
TMEM100	1.935079	0.778159	-1.15692	0.003715
HS1BP3-I	6.692427	4.240103	-2.45232	0.003736
MAIP1	14.28545	13.04049	-1.24496	0.003771
TCN1	0.25335	2.195567	1.942217	0.003786
SERPINA1	4230.265	3455.404	-774.861	0.003793
MFSD2A	13.2309	7.534846	-5.69605	0.003797
FBXO17	6.414248	7.802556	1.388308	0.003816
HEYL	3.884395	5.426437	1.542042	0.003845
PANX1	7.455949	6.119415	-1.33653	0.00385
RAB6A	15.35687	17.38235	2.025482	0.003856
TNFRSF19	2.27584	4.142593	1.866753	0.003904
LTB	5.872461	17.69948	11.82702	0.003921
LINC0112	4.234638	2.514753	-1.71988	0.003924
FBXW5	45.60946	51.51858	5.909129	0.003952
SH3BP4	12.27993	9.983336	-2.29659	0.003984
PSAP	401.6345	458.2234	56.5889	0.003986
ACADS	36.81988	30.27212	-6.54775	0.004053
CLDN14	9.905936	7.452986	-2.45295	0.004073
APCDD1	2.044042	5.785265	3.741223	0.004092
TINF2	11.66788	12.91842	1.25054	0.004138
DENND1C	3.703176	5.005155	1.301978	0.004151
SLC5A9	3.920156	5.304504	1.384348	0.004183
MKNK2	22.05636	25.54409	3.487728	0.004202
B3GALT2	0.161309	1.297972	1.136663	0.004203
POLR2E	33.0327	36.45736	3.424667	0.004255
CD3E	2.752771	5.752851	3.00008	0.00426
CC2D1A	8.474009	10.29088	1.816875	0.004266
AL391427.	1.202827	2.29449	1.091663	0.004267
LYPLA1	15.49243	13.4938	-1.99863	0.004284
LINC0094	0.966068	4.237667	3.271599	0.00429
CD164	28.69208	32.755	4.062918	0.004295
CASP3	8.432389	9.749779	1.31739	0.004303
SEC24B	7.973904	6.674444	-1.29946	0.00431
CKB	21.87323	37.11711	15.24388	0.004315
SS18L1	6.735255	5.634434	-1.10082	0.004329
ACY3	16.35841	12.6015	-3.75691	0.004344
ABHD14B	41.7946	36.03389	-5.76071	0.00435
MXRA5	0.805326	2.012383	1.207057	0.004375
ATP2A3	1.58165	3.414389	1.832739	0.004393
DUSP10	13.96514	11.48196	-2.48318	0.004435
CENPX	35.45361	44.62691	9.173303	0.00444
HNRNPCF	7.342928	8.494328	1.1514	0.004447
KCNJ4	2.059365	0.966533	-1.09283	0.00445
ZNF189	6.166033	7.588426	1.422393	0.004471
PPARG	3.933756	5.25301	1.319255	0.004488

PNPLA2	16.46747	18.9468	2.479333	0.00452
BNIP3	24.82075	21.47456	-3.34619	0.004535
AC108751	2.068276	3.205793	1.137517	0.004556
EFHD1	9.102504	5.933872	-3.16863	0.004558
MTERF3	10.17961	11.72164	1.542036	0.004578
IGKV1-5	14.43672	60.76264	46.32592	0.004585
AKR7A2	27.0693	30.94433	3.875021	0.004594
SPCS2	14.33205	17.33119	2.999139	0.004601
ZFAND5	32.05398	22.72935	-9.32463	0.004605
RAMP2	10.02973	12.82015	2.790413	0.004639
KMT2B	4.495702	5.839478	1.343776	0.004649
CCL20	20.80434	34.98133	14.17699	0.004659
RPL7L1	11.74965	13.17064	1.420992	0.004694
IGKJ1	3.41127	10.00559	6.594318	0.004711
RHOBTB2	1.08954	2.154815	1.065275	0.004747
CLDN9	1.422116	4.683363	3.261247	0.004774
DHX16	10.94469	12.28904	1.344349	0.004808
TEF	9.631593	7.996573	-1.63502	0.004811
LSS	16.97925	13.71455	-3.2647	0.004812
PDHA1	17.16614	19.44114	2.275	0.004813
XPNPEP2	23.0146	11.93807	-11.0765	0.004832
ANKRD65	2.520085	4.152957	1.632871	0.004853
HRCT1	7.11352	12.17341	5.059887	0.004872
LCAT	17.43504	12.83868	-4.59636	0.004872
MLXIPL	65.9371	54.44036	-11.4967	0.004878
RASAL3	1.206574	2.390187	1.183613	0.004887
RNF128	31.04864	25.94073	-5.10791	0.004945
FAM3A	12.44254	14.24095	1.798417	0.004959
MEPCE	18.38464	20.30428	1.919645	0.00499
SHROOM:	20.33093	17.29343	-3.0375	0.004991
HSPA1A	53.16891	73.69179	20.52288	0.005008
MPC1	50.30193	41.952	-8.34993	0.00502
SULT1C4	0.93691	8.024316	7.087406	0.005021
SNX10	8.414685	10.88036	2.465672	0.005042
CALB2	0.035903	1.826448	1.790544	0.00508
COL10A1	0.42246	1.892588	1.470129	0.005104
TMED2	71.46135	78.88051	7.419159	0.005131
TNFRSF1B	12.20594	15.00042	2.794478	0.005132
AGPAT1	9.465593	10.92463	1.459033	0.005137
FCN3	5.889814	2.710599	-3.17922	0.005177
MT-ND4	10010.68	8505.424	-1505.26	0.005182
DNER	0.333301	3.041699	2.708397	0.005217
GUCD1	45.07867	39.66759	-5.41108	0.00523
LPA	4.294048	2.664147	-1.6299	0.005287
CLDN23	5.196092	7.494875	2.298784	0.005425
LGR4	14.74467	12.18443	-2.56024	0.005447
CXCL14	1.352299	5.703359	4.35106	0.00545
RAB1A	41.95686	46.30808	4.351221	0.00548
VWA1	21.25118	25.96416	4.712974	0.005491
PIK3IP1	6.103667	8.207019	2.103352	0.005514
PCBD1	123.2828	107.8624	-15.4204	0.005544
IGHV3-64	0.353909	1.635692	1.281783	0.005569
AC093675	2.958754	1.669794	-1.28896	0.005625
SPIB	0.135982	2.159398	2.023416	0.005637
LY96	11.90179	18.85553	6.953738	0.00566
PLA2G7	2.904155	5.188868	2.284713	0.005675
OSBPL9	8.170595	9.779546	1.608951	0.005682
SOD1	314.7196	265.9681	-48.7515	0.005875

GLDC	9.514273	7.488888	-2.02538	0.005906
ESD	27.52063	24.93602	-2.58461	0.005915
CYP1A1	28.69938	8.069425	-20.63	0.005935
MT-ND2	5507.713	4601.354	-906.359	0.005949
NRP1	7.050944	9.161486	2.110542	0.005949
FBXL6	5.989216	7.215947	1.226731	0.005975
BCL2L10	8.168862	4.778643	-3.39022	0.00598
TMEM151	0.622869	2.319106	1.696237	0.005986
VCAN	2.15428	4.520955	2.366675	0.005989
VSIG4	3.003445	5.25457	2.251125	0.006014
SNHG32	5.260768	6.656384	1.395616	0.006058
PPP6R2	27.52929	24.43445	-3.09484	0.006075
ATXN10	17.22729	19.11328	1.885987	0.006085
EI24	47.76457	42.95755	-4.80701	0.006135
NRTN	5.329078	6.811943	1.482865	0.006149
TMEM82	12.54659	8.253987	-4.2926	0.006189
PXDC1	47.25598	40.67258	-6.5834	0.0062
BCAM	36.05086	50.75807	14.70721	0.00622
TUT7	6.102388	5.051757	-1.05063	0.006239
CEBPA-DT	4.138654	5.371804	1.23315	0.006239
COL3A1	49.4506	85.35559	35.90499	0.006277
REEP3	6.674313	7.711298	1.036984	0.006281
MMP10	0.358104	2.014561	1.656456	0.006281
KLHDC10	10.25029	8.946559	-1.30373	0.006292
TCF7L1	4.404015	5.49719	1.093175	0.006318
SULT1C2	1.986822	3.111672	1.124851	0.006364
UBQLN4	11.96941	14.00784	2.038435	0.006386
CTSL	22.43562	26.02051	3.584882	0.006413
ORM1	2678.578	2048.458	-630.12	0.006415
SLC22A3	11.19397	8.561504	-2.63247	0.006453
SASH3	1.758946	3.545732	1.786785	0.00648
PCP4L1	2.919964	1.775113	-1.14485	0.006482
ABHD4	10.3881	12.33658	1.948481	0.006495
DCAF4L2	2.324578	4.361159	2.036581	0.006504
C11orf58	9.962922	11.03798	1.075056	0.006526
IGKV3-15	9.807112	28.0264	18.21928	0.00658
LGALS2	2.305006	8.979049	6.674042	0.006615
CD27	0.541505	1.656621	1.115116	0.006619
HLA-H	29.71333	36.91853	7.205198	0.00663
WWP1	21.21812	18.36125	-2.85686	0.006672
HSPA8	149.3644	171.9756	22.6112	0.006712
RCAN2	1.954523	3.106069	1.151546	0.006755
ITGB6	0.078539	1.307323	1.228784	0.006759
SEMA4G	12.84842	10.51185	-2.33657	0.006778
SAP18	27.82295	30.61098	2.788028	0.006828
TOM1	18.28426	20.55338	2.269119	0.006837
ERGIC1	18.65807	16.50553	-2.15253	0.006875
PSAT1	41.46384	32.21621	-9.24763	0.006998
PMPCA	11.65849	13.29378	1.635292	0.007074
PTK6	2.361058	3.737809	1.376752	0.007079
HS3ST3B1	6.994671	4.603721	-2.39095	0.007087
CLNS1A	14.58021	16.14309	1.562888	0.007103
ASGR2	239.6895	199.5206	-40.1689	0.007127
TNFRSF10	10.30508	12.35378	2.048696	0.007186
NCOA7	4.922317	6.454025	1.531708	0.007225
PACSIN3	12.33169	15.20434	2.872659	0.007237
IGHV4-28	0.830163	2.861306	2.031144	0.007271
AL031668.	0.053609	2.176756	2.123146	0.007347

ARFGAP3	14.32423	16.19036	1.866126	0.007348
IGHV1-2	5.61056	22.76618	17.15562	0.007355
LAPTM4A	165.545	183.7784	18.23336	0.007374
IGHV3-48	3.9897	12.50439	8.514691	0.007385
AP003119	3.529122	2.281579	-1.24754	0.007401
EXT1	13.5195	16.14807	2.628576	0.007404
TNS3	13.08829	11.08027	-2.00802	0.007447
HSPA5	227.8778	257.1857	29.30788	0.007461
DDX21	10.83068	12.86424	2.033566	0.007543
FAM3C	5.789557	6.919468	1.129911	0.007561
SIAH2	27.05167	22.58977	-4.4619	0.007591
AVPR1A	4.789887	2.216371	-2.57352	0.007612
COPA	32.71293	37.4686	4.755666	0.007662
ARL8A	25.02585	28.36391	3.338068	0.007665
AIG1	15.97977	14.05129	-1.92848	0.007674
PPIAP29	3.052767	5.023581	1.970813	0.007719
IGLV2-8	1.975694	7.944757	5.969063	0.007737
RING1	30.90364	34.72217	3.818531	0.007753
SDF2L1	47.94684	57.06047	9.113629	0.007757
MRC2	2.045503	4.86267	2.817167	0.007923
LRRC20	14.46169	12.27282	-2.18887	0.008047
ATP13A3	11.92824	10.34744	-1.5808	0.008056
CYP2A13	3.526222	0.837623	-2.6886	0.008073
PPP4R3B	8.908776	10.05234	1.143563	0.008149
MTCH2	52.52903	48.30053	-4.22851	0.008188
IGLV6-57	4.727667	15.23543	10.50776	0.008229
TBC1D10(	1.207015	2.669578	1.462563	0.008233
DDX3X	10.19492	11.47446	1.279534	0.008248
CHCHD10	102.716	125.0501	22.33409	0.008293
SPINK1	191.982	313.6135	121.6314	0.008484
AC025423	4.142141	2.989903	-1.15224	0.008513
ANXA10	10.45309	6.366473	-4.08662	0.008558
ESM1	3.82015	5.813896	1.993746	0.008588
CTSE	0.533338	6.599257	6.065919	0.008597
SNCG	10.59029	34.11169	23.5214	0.008687
IGHV3-74	2.9618	8.048217	5.086417	0.008695
COL6A2	26.63123	41.58943	14.9582	0.008708
PTHLH	0.330175	2.561343	2.231168	0.008716
ASCL1	1.678887	0.498765	-1.18012	0.008724
AGPAT3	12.17334	10.91456	-1.25878	0.008769
MLEC	32.75484	36.53044	3.775596	0.008824
SCGN	8.184991	14.99023	6.80524	0.008847
SPOCK2	2.342197	3.954951	1.612754	0.008882
AREG	0.906831	2.351851	1.44502	0.008965
DERA	17.0091	15.10745	-1.90166	0.008979
RFLNA	0.218908	1.98534	1.766432	0.008989
GNMT	84.53151	51.88057	-32.6509	0.009089
TEX264	13.572	14.9273	1.355293	0.009147
UPK3B	0.232005	1.273178	1.041172	0.009176
IGHV3-15	6.133588	18.77009	12.6365	0.009222
SPON2	31.27549	38.93901	7.66352	0.009265
DPYD	6.98267	5.747782	-1.23489	0.009277
H2BC21	6.931467	9.284081	2.352613	0.009287
SCGB2A1	0.359614	1.496609	1.136995	0.009376
AL450405.	8.775857	16.53378	7.757928	0.009408
UGT3A2	0.591597	1.638726	1.04713	0.009413
BTG2	12.84266	17.4601	4.617445	0.009414
NFE2L1	50.80232	43.95057	-6.85175	0.009455

IGLV8-61	2.342726	19.27655	16.93383	0.009486
ITPRID2	9.367437	8.005533	-1.3619	0.00949
DUOXA2	0.60276	5.094342	4.491583	0.009495
PRSS3	3.343163	7.499342	4.15618	0.00951
LINC0184:	2.661866	4.577784	1.915918	0.009517
SRP9	67.14799	74.50308	7.355082	0.009595
ZHX2	10.21621	11.93722	1.721006	0.009642
KEL	0.265372	2.221889	1.956517	0.00966
HACL1	11.88656	10.18072	-1.70584	0.0097
C17orf58	6.322368	7.378503	1.056135	0.009783
PCSK9	9.264777	11.7527	2.487919	0.009811
SHC1	24.67122	30.45038	5.779156	0.009971
ALKBH7	54.81549	64.16939	9.353893	0.00999
SHMT2	39.57478	44.81627	5.241488	0.010082
CYB5R1	17.24253	19.59353	2.351006	0.010195
STRADB	14.29135	12.76459	-1.52676	0.01028
ACSS2	22.97253	18.1166	-4.85593	0.010342
IGKV2-24	1.294155	5.437371	4.143217	0.010468
PEX5	11.34298	10.20925	-1.13372	0.010487
GSTM4	5.468245	6.849149	1.380904	0.010539
IL17RC	8.591965	7.431611	-1.16035	0.010609
STARD10	91.71006	78.65119	-13.0589	0.01063
IFITM3	901.0573	1037.322	136.2644	0.010661
GPX2	230.082	318.3418	88.25975	0.01084
PROCR	2.086686	6.166141	4.079455	0.010892
TAX1BP1	12.75606	14.11966	1.3636	0.010922
ATPAF1	10.05528	11.06908	1.013801	0.010984
HMGCR	12.44903	10.41636	-2.03267	0.011096
FAM174A	11.26875	12.31486	1.046109	0.011159
PJA2	18.70553	16.72176	-1.98377	0.011167
AKIRIN2	15.55791	17.88933	2.331425	0.011244
GABRP	0.217505	4.465782	4.248277	0.011246
IRF2BP1	13.76228	18.78216	5.019881	0.011329
SCD5	1.617915	5.717037	4.099123	0.011354
MAFF	4.133481	5.215809	1.082328	0.011395
MAGEA12	1.910922	3.855108	1.944186	0.011489
LACTB2	14.28468	11.99595	-2.28873	0.01149
MRPL21	12.49001	20.29959	7.809574	0.011546
REPIN1	27.83783	31.05883	3.220994	0.011671
SERINC1	55.92357	49.86386	-6.05971	0.011793
FAM43A	3.976296	2.861499	-1.1148	0.011802
SLC6A2	2.673397	1.000547	-1.67285	0.011835
GPAA1	68.14366	80.04617	11.90252	0.011848
TRBD1	1.978837	4.433167	2.454331	0.011898
SNX4	16.79524	15.29443	-1.50081	0.011903
TRAM1	65.06382	75.46614	10.40232	0.011929
MTMR4	9.54006	8.075806	-1.46425	0.011977
GOLM2	8.379515	9.664724	1.285208	0.01214
ZNF467	4.145325	5.43139	1.286065	0.012149
IGKV1D-8	0.271838	1.478794	1.206956	0.012201
FAM83D	4.896033	6.052498	1.156465	0.012239
RER1	18.04934	19.82523	1.775898	0.012284
MSX1	0.76708	1.813911	1.046831	0.01233
ACSF2	12.74483	10.84227	-1.90256	0.012392
KRT17	0.743973	8.28412	7.540146	0.012621
CHMP1B	11.07796	12.73092	1.65296	0.012665
BPNT1	11.28985	12.58117	1.291319	0.012675
NCSTN	32.06865	36.12466	4.056009	0.012686

MARCHF2	21.46357	19.39475	-2.06882	0.012689
DDX24	10.63296	11.66036	1.027399	0.01269
IGHV1-69	1.477482	5.656744	4.179262	0.012747
RNF11	17.83329	19.90139	2.068102	0.012759
FSTL1	6.788295	10.17236	3.384069	0.012784
NFIL3	23.45627	19.87595	-3.58031	0.012796
MID1IP1	16.24078	19.51228	3.271498	0.012809
CHDH	12.00799	10.56338	-1.44461	0.012818
TCTA	18.56551	16.57278	-1.99273	0.012819
RHEX	0.47912	2.983881	2.504762	0.012823
RND1	16.25291	12.3584	-3.89451	0.012881
AIFM1	9.620304	8.478867	-1.14144	0.012896
IGHV1-46	2.98345	10.1554	7.171954	0.012915
LRRC45	10.14702	11.96074	1.813722	0.012997
S100P	87.90273	206.5341	118.6314	0.013209
MINPP1	10.30577	9.02423	-1.28154	0.013229
SHBG	17.05625	10.98829	-6.06796	0.013272
TNNI2	1.081098	5.172428	4.09133	0.013364
POLR2C	14.04039	15.82821	1.787817	0.013392
IGKV1-8	0.799255	3.121024	2.321769	0.013489
LRPAP1	12.47895	13.92653	1.447571	0.013554
CCNG1	37.00517	32.93471	-4.07046	0.01359
KLF4	2.937204	3.983307	1.046103	0.013619
IGHG4	9.981598	29.98606	20.00446	0.013622
EPHA1	8.594796	6.691518	-1.90328	0.013713
TSPAN8	35.3787	50.59355	15.21485	0.013752
PRODH	2.294909	1.146355	-1.14855	0.013802
VAMP5	66.85196	78.46185	11.6099	0.013824
RAP2A	6.256555	7.624831	1.368275	0.013891
NET1	10.4034	12.71993	2.316521	0.014011
GJB5	0.136456	1.179998	1.043542	0.014049
LFNG	1.405638	3.91296	2.507322	0.014073
MRPL40	24.378	26.94934	2.571344	0.014123
HHLA3	6.697176	7.989937	1.292762	0.014158
IGKV1-12	2.029894	9.253212	7.223319	0.014191
GPRC5A	0.165536	2.16105	1.995514	0.014271
PRKAA1	8.45715	9.6184	1.161249	0.014315
IRF6	22.63363	18.88627	-3.74736	0.014415
NAXD	21.67876	19.2445	-2.43425	0.014454
CD52	6.694833	16.07765	9.382818	0.014484
OLFML2B	2.306875	5.427639	3.120765	0.014626
SLC7A10	0.295003	1.587707	1.292704	0.014653
ZNHIT1	29.02541	34.72636	5.700951	0.014695
SLC16A1	19.6895	15.31631	-4.3732	0.01477
SLC51A	12.82378	9.307448	-3.51633	0.014821
TFF3	4.497895	17.53834	13.04045	0.014938
SCYL1	20.38453	21.8108	1.426266	0.015062
ERF	17.22777	19.79077	2.563001	0.015076
HSPD1	67.003	75.26876	8.26576	0.015263
PPA2	9.528307	10.66956	1.141252	0.015314
F13A1	2.092649	3.742828	1.650179	0.015315
RITA1	10.81977	12.02724	1.207465	0.015326
MRPS36	14.37042	15.77217	1.401751	0.015345
AL031058.	3.997183	6.185518	2.188335	0.015494
PRUNE1	9.407594	10.82243	1.41484	0.015537
CERS4	20.05135	17.07299	-2.97837	0.01554
MMP2	6.860924	14.52217	7.661248	0.01563
QPCT	0.994375	2.331414	1.337039	0.015678

GATA4	6.986159	8.459081	1.472922	0.015724
ADA2	3.536866	4.567913	1.031048	0.015741
LAMP5	0.44232	5.222187	4.779867	0.015743
ECHDC2	15.72665	13.47469	-2.25195	0.015857
LRATD2	5.182129	6.466065	1.283936	0.015886
IGLV1-47	10.40919	29.9378	19.52861	0.015951
YME1L1	14.55025	16.09174	1.541489	0.015976
CST1	3.830686	31.58584	27.75515	0.01605
IGHV4-59	7.443617	25.2491	17.80548	0.016224
AL035446.	0.599267	1.649961	1.050693	0.016281
PPP2CB	11.32507	12.85897	1.533896	0.016299
CREB3L3	100.7216	79.22976	-21.4919	0.016377
SLC44A4	0.379158	2.740897	2.361738	0.016396
IGSF23	13.82613	9.018012	-4.80811	0.016438
ACAT2	18.00515	15.00181	-3.00334	0.016467
PLAU	1.963172	5.917346	3.954173	0.016471
TINAG	1.696381	3.266241	1.569861	0.016487
BID	10.54595	11.9622	1.416257	0.016507
C6orf47	7.723058	8.861882	1.138824	0.016527
NRBF2	12.38965	11.33501	-1.05464	0.016568
TFPI	24.06252	19.14298	-4.91955	0.016583
GALNT2	15.35508	13.33614	-2.01895	0.016619
FTL	11013.42	13512.61	2499.193	0.016754
TMEM238	7.270238	8.8885	1.618262	0.016856
UGT1A9	15.51726	10.88721	-4.63005	0.016859
PLEKHF1	6.735076	8.15729	1.422213	0.01687
IGHV5-10	2.309072	7.8794	5.570328	0.016906
AP001783	10.94991	6.048868	-4.90104	0.017111
BGN	101.2925	134.7607	33.46822	0.017252
PPP4R2	11.02602	9.860252	-1.16577	0.017314
CANX	120.265	134.376	14.11095	0.017356
MYC	9.3297	11.70385	2.374151	0.017357
DCAKD	9.347156	10.46061	1.113456	0.017361
ELFN1-AS	0.395399	1.498978	1.10358	0.017363
NAGA	17.28622	15.04588	-2.24033	0.017468
PPP1R14A	3.2128	4.320453	1.107654	0.017485
BRD2	17.81057	19.77045	1.959882	0.017558
IGHV4-39	10.8018	41.00428	30.20248	0.017581
RAB1B	21.65113	23.96787	2.316735	0.017607
MUL1	12.77789	13.92326	1.145369	0.017615
LAMA5-A	3.462317	2.412726	-1.04959	0.01768
IGKV1D-1	0.582228	2.385156	1.802928	0.017769
GSTA1	494.4993	380.4825	-114.017	0.017772
MAN2B1	10.57291	11.95147	1.378555	0.017814
EFNB1	6.559539	13.78449	7.224956	0.017843
AC016739	4.9122	7.916918	3.004717	0.017856
IGKV1-6	2.432008	12.83235	10.40035	0.017869
IGHV3-30	7.090506	17.44867	10.35816	0.017964
SEC62	17.20437	15.84966	-1.3547	0.018025
F10	95.11126	77.51039	-17.6009	0.01807
GPC1	8.428051	13.88477	5.456716	0.018131
NAPSB	1.677287	4.326257	2.64897	0.018135
CHKA	6.725876	8.332208	1.606333	0.018163
EEF1AKM1	4.142136	5.550691	1.408554	0.018236
TTLL12	11.85054	13.44051	1.589975	0.018268
GBP5	0.847239	2.681902	1.834664	0.018441
MEG3	1.013443	2.519521	1.506078	0.018522
CACNG4	1.012322	2.589649	1.577328	0.018571

ZNF703	4.710208	6.590138	1.87993	0.018611
TFB2M	18.21574	16.38103	-1.83471	0.018644
ERP44	17.90532	19.30594	1.400612	0.018713
MAP2K3	14.82996	16.72537	1.895409	0.018713
HSP90AA1	146.5642	164.889	18.32475	0.018788
ALG1L	4.388721	6.076401	1.687681	0.018956
SIT1	0.790858	2.003921	1.213063	0.019003
IGHV4-55	0.415787	1.534399	1.118612	0.019227
YIF1A	46.27986	50.9341	4.654242	0.019325
IGHV1-24	3.228492	18.30392	15.07543	0.019352
IGKV3D-1	0.365505	2.043419	1.677914	0.019412
NPTN	16.55337	18.4661	1.912724	0.0195
RTN1	0.414634	1.422383	1.007749	0.019606
SSBP3	11.70388	13.35505	1.651174	0.019615
NECAB3	9.712388	11.51285	1.800463	0.019659
ARV1	10.27552	11.45987	1.184345	0.019704
APLNR	7.038412	5.639213	-1.3992	0.019944
MERTK	5.432253	3.758447	-1.67381	0.019964
RNF5	76.76433	86.08522	9.32089	0.020032
UQCRFS1	24.44923	27.75578	3.306544	0.020089
DUOX2	0.85825	10.31654	9.458289	0.020139
SERP1	29.37287	31.78315	2.410284	0.0202
ACSM1	17.62804	11.95992	-5.66812	0.020229
SLC6A19	0.630393	2.586542	1.956149	0.020266
PRADC1	21.87052	24.81362	2.943107	0.02033
ADIPOR1	47.40067	51.70888	4.308205	0.020396
TSPAN9	12.58367	11.03979	-1.54388	0.020536
GPAT4	7.378179	8.763067	1.384888	0.020619
IGLV2-14	16.51672	40.12528	23.60855	0.02064
RNF26	9.491191	10.67529	1.184096	0.020704
MAT2A	16.00702	18.83371	2.826685	0.020723
CLDN1	89.75006	106.8143	17.0642	0.020783
SPSB1	13.07157	15.91256	2.840987	0.020894
OASL	7.746795	5.76353	-1.98327	0.021052
DMBT1	0.041316	1.785965	1.744649	0.021118
ARL4D	23.81579	18.37306	-5.44273	0.021248
FOXP4	11.51093	13.12549	1.614558	0.021262
RAP2C	11.46479	10.06629	-1.39851	0.021292
EIF4H	25.87394	28.06375	2.189818	0.02134
FAM20B	10.30709	11.49891	1.191817	0.021361
AL391095.	10.83636	8.28181	-2.55455	0.021369
H1-2	182.5542	237.7889	55.23469	0.021404
NTHL1	12.3307	14.33166	2.000956	0.021518
IGLV3-19	18.07205	97.04164	78.96959	0.021525
SIPA1L2	3.713016	4.937458	1.224442	0.021565
TADA1	8.791337	7.770854	-1.02048	0.021568
TRIM16L	4.971274	7.354249	2.382975	0.021675
POLR2B	6.967301	8.054969	1.087668	0.021969
UBE2K	14.57516	16.01073	1.435568	0.022144
NDRG4	0.164771	2.193286	2.028514	0.022185
IGLV7-46	1.139831	7.598679	6.458848	0.022355
GALT	9.582566	10.9202	1.337637	0.022449
MYOM1	7.356156	5.866793	-1.48936	0.022525
ECI2	23.00833	20.17732	-2.83101	0.022921
CEACAM6	0.287905	17.92241	17.6345	0.023145
USP22	10.05295	11.7823	1.72935	0.023289
UPF1	12.5154	13.87155	1.356149	0.023478
MYD88	12.50053	11.33324	-1.16729	0.023494

DDIT4	12.74304	16.75734	4.014298	0.023496
GPX1	257.3748	294.3811	37.00632	0.023508
CFI	14.46284	12.31593	-2.14691	0.02359
IGLV4-60	0.497337	2.560284	2.062947	0.023594
PRPF8	13.75403	15.26918	1.515152	0.023619
MGST2	34.87867	31.05667	-3.822	0.02376
SPR	51.80484	56.90786	5.10302	0.023773
QPCTL	5.659731	9.599797	3.940067	0.02382
CYP2S1	1.068218	2.762016	1.693798	0.023855
IGKV1-9	4.120986	28.56124	24.44025	0.023895
ERP27	0.787706	2.64299	1.855284	0.023905
SELL	1.511583	4.017496	2.505913	0.023925
NAALADL	1.024039	2.62628	1.60224	0.02396
LOXL1	1.25758	3.931023	2.673443	0.024069
EEF2	417.0855	463.9607	46.87522	0.024076
LINC0141	3.975419	13.38788	9.412465	0.024227
SYT7	14.03189	9.14064	-4.89125	0.02431
IGHM	20.69072	80.73616	60.04545	0.024425
CRIM1	6.963101	9.672555	2.709453	0.0246
EPHA2	5.941113	8.041496	2.100383	0.024686
IGLV3-25	10.93272	26.42857	15.49585	0.024818
FAM83A-/	4.893019	2.289421	-2.6036	0.024839
KLF10	14.97362	12.56692	-2.4067	0.02484
ZKSCAN1	12.46406	11.08619	-1.37787	0.025052
SLC35C1	30.10675	25.29918	-4.80757	0.025111
MAGEH1	6.839413	13.24974	6.410325	0.025289
IBSP	0.138439	1.658887	1.520448	0.025293
EID1	45.99034	51.19544	5.205098	0.025319
EXOC3L4	8.932485	7.065005	-1.86748	0.025335
TRIM26	23.15796	21.11714	-2.04081	0.025359
PTDSS1	13.21835	14.85508	1.636731	0.02537
CIDEC	1.535612	3.177389	1.641776	0.025395
GDA	7.046551	5.472747	-1.5738	0.025579
TP53INP2	17.86567	15.24776	-2.61791	0.025742
IGHV5-51	10.29107	22.29041	11.99934	0.026049
UBQLN2	8.653134	9.676198	1.023064	0.026079
HAO2	29.6015	20.09312	-9.50838	0.026126
TMC8	1.155815	2.723392	1.567576	0.02613
MT-ATP8	1315.575	1081.04	-234.534	0.026175
MT-ND1	5313.4	4629.083	-684.317	0.026331
CD9	8.865223	10.92139	2.056172	0.026668
ATXN7L3E	9.891481	11.23634	1.344855	0.026699
CTSS	23.99342	29.62235	5.628933	0.02692
FBXL5	18.05089	15.9281	-2.1228	0.026954
CAMK2N2	3.09274	4.319084	1.226344	0.026963
MYCL	4.39351	3.302925	-1.09059	0.027013
GAA	35.22066	39.56418	4.343523	0.027085
TMEM158	0.36822	2.41451	2.04629	0.02721
ATP5PF	39.78917	44.10644	4.31727	0.027211
PFN1P11	6.77405	2.559951	-4.2141	0.027235
SYT8	0.747125	6.196665	5.449539	0.02753
TRIM55	13.74592	10.48712	-3.2588	0.027583
DNPH1	56.83964	64.1252	7.285554	0.027648
OAS3	4.065303	5.148003	1.0827	0.027716
PIR	11.4412	13.7649	2.323692	0.027782
SOD3	6.129622	16.00244	9.872819	0.027897
IL1R1	13.21618	11.31051	-1.90567	0.028011
HGSNAT	7.442903	8.628049	1.185146	0.028046

AFMID	26.25744	23.47991	-2.77753	0.028108
IGF2	14.44006	6.73054	-7.70952	0.02814
SLC25A4	11.19136	9.806427	-1.38493	0.028175
ELF1	6.570037	7.725656	1.155619	0.028199
CD79B	0.643484	2.101249	1.457765	0.028244
IGHA1	55.60634	178.5189	122.9126	0.028258
ALG5	16.03698	17.34869	1.3117	0.028388
KDM8	4.72976	3.438815	-1.29094	0.028552
MPST	43.36845	49.60777	6.239319	0.028554
ERLEC1	22.5347	24.62333	2.088627	0.028664
C6orf62	48.80678	54.2148	5.408023	0.028694
IDNK	8.33224	7.202748	-1.12949	0.028743
OLFM4	0.110743	10.65118	10.54044	0.028774
RRAGA	38.06552	41.51589	3.450375	0.028848
SLC2A4RG	43.6937	49.95506	6.261354	0.028895
DDC	13.4792	11.09791	-2.38129	0.029208
H2BC5	30.63997	37.11317	6.473202	0.029233
GBP3	5.235496	6.437025	1.201529	0.029296
H2AC8	4.811146	6.486064	1.674918	0.029381
IGKV1OR2	0.561397	2.184845	1.623448	0.029588
IGLV3-10	3.070025	26.11164	23.04162	0.029671
ANKRD22	0.784088	1.920693	1.136605	0.030276
IGHV6-1	2.246291	6.45711	4.21082	0.030486
PMM1	7.197293	8.253383	1.05609	0.030532
ANGPTL4	59.95062	47.23998	-12.7106	0.030608
PTMS	324.3351	366.6854	42.3503	0.030635
UBL3	11.76651	10.12369	-1.64282	0.030856
TSC22D1	18.64674	15.02784	-3.6189	0.030971
CREB3L1	1.59397	3.47697	1.883	0.030985
ATP5MC3	56.48285	62.57593	6.093084	0.031034
CGNL1	12.25759	8.92965	-3.32794	0.031079
HSPA9	45.98086	42.21867	-3.76219	0.031129
IGKV4-1	23.01093	98.8787	75.86777	0.031148
GBP7	8.427217	6.396641	-2.03058	0.031216
PPP6R3	7.55338	8.793194	1.239813	0.031242
CRTAP	17.6158	19.98924	2.37344	0.031318
FTLP2	3.921109	5.288665	1.367556	0.031428
CLIC4	14.36449	16.85394	2.489451	0.031558
LTBP4	5.10301	6.560795	1.457784	0.031703
AL138826.	2.857798	4.741869	1.884072	0.031856
SIL1	26.07869	29.38868	3.309992	0.031911
FGFR2	9.207822	12.25492	3.047094	0.032086
CXCL11	1.574019	2.718935	1.144916	0.03209
TMEM179	13.67895	14.7148	1.035856	0.0323
CST6	0.059854	1.090856	1.031002	0.032509
SNTA1	11.9169	18.06887	6.151979	0.032517
FDCSP	5.162106	61.91048	56.74838	0.032566
RAMP1	94.00029	76.1292	-17.8711	0.032689
SYAP1	12.28874	13.48474	1.196006	0.032757
DUSP6	15.30074	18.05684	2.756099	0.032886
APLP1	1.096988	3.932782	2.835794	0.032944
RXRB	12.06405	13.2999	1.235844	0.032954
SMPDL3A	17.76192	14.70137	-3.06055	0.032998
CXADR	10.36646	12.15813	1.791669	0.033479
NFIC	18.72287	16.67203	-2.05084	0.033725
MAP3K11	13.49929	14.56527	1.065978	0.033746
AGTR1	11.11211	9.212058	-1.90005	0.033786
MBNL3	15.63832	12.32545	-3.31287	0.033833

COL6A1	26.39197	37.59358	11.20161	0.033986
AC136475	7.020739	4.385457	-2.63528	0.034028
CD37	1.519071	3.959731	2.44066	0.034067
OSGIN1	43.45798	35.1473	-8.31069	0.034107
PPP1R16A	16.798	19.94382	3.145822	0.034128
NEK7	7.970731	9.106659	1.135929	0.034159
SIGMAR1	91.54537	82.23666	-9.30872	0.034194
IGLV3-27	1.384141	8.100983	6.716842	0.03421
TECR	17.87619	23.12522	5.249035	0.034293
IGFBP2	118.1952	86.92061	-31.2746	0.034606
IGHV3-66	0.881391	4.67289	3.791499	0.034649
RAP2B	2.523522	5.125727	2.602205	0.034803
FCGRT	67.62216	60.63414	-6.98802	0.034829
S100A8	2.189846	10.66826	8.478412	0.035477
CPPED1	12.61874	11.03368	-1.58506	0.035675
STIM1	13.92161	12.72767	-1.19394	0.035869
IGLV3-1	19.2579	48.40594	29.14804	0.036171
AMN	9.321296	7.045405	-2.27589	0.036478
RPL35P5	0.935395	2.885496	1.9501	0.036886
STC2	1.606841	2.636569	1.029728	0.037227
SMIM24	9.244191	24.99704	15.75285	0.037248
TRDC	0.859433	2.50825	1.648816	0.037497
CLGN	4.394969	6.045206	1.650237	0.03766
PLEK2	5.903073	8.260536	2.357464	0.037749
CILK1	5.791103	4.583464	-1.20764	0.037937
CLDN2	29.52556	19.99062	-9.53493	0.037945
AQP8	4.50338	2.016498	-2.48688	0.038077
APOE	3341.428	2845.55	-495.878	0.038317
FOXA3	24.31902	31.84112	7.522098	0.038525
MSLN	0.086863	4.896092	4.80923	0.038783
DPEP1	0.492477	1.567861	1.075384	0.039101
SLC23A1	8.533821	7.348584	-1.18524	0.039227
AFP	118.6144	294.9048	176.2904	0.039369
SERINC2	76.57623	89.11038	12.53414	0.039457
FAT1	9.978593	8.342319	-1.63627	0.039558
MTATP6P1	919.1605	808.1474	-111.013	0.039612
IGKV3D-2	0.93024	7.924378	6.994138	0.039679
CSTA	10.11987	12.7558	2.635934	0.039812
FLNC	2.075423	3.757264	1.681841	0.039994
MEF2D	9.449386	10.49032	1.040933	0.040065
HMOX1	22.97678	36.59716	13.62038	0.040065
CD22	0.248329	1.498993	1.250664	0.040097
REEP5	38.91484	41.90921	2.994373	0.040349
BANF1P2	6.103084	4.678313	-1.42477	0.040404
AL360169	1.806607	0.329783	-1.47682	0.040484
GPAT3	4.643463	3.490525	-1.15294	0.040505
PGRMC2	22.9471	21.476	-1.4711	0.040521
CYP2C18	15.93013	13.03832	-2.89181	0.040574
IL6ST	21.67227	19.62149	-2.05077	0.040591
SEC11C	26.89344	30.33529	3.441853	0.040639
CD163	3.815951	5.110949	1.294998	0.040705
OCIAD2	22.80236	26.07916	3.276803	0.041813
SLC39A11	10.49807	9.469217	-1.02885	0.041914
MEST	5.58994	10.38099	4.791052	0.042037
TMEM70	13.99377	12.42758	-1.56619	0.042142
SLC25A18	8.919182	7.322402	-1.59678	0.042143
BPIFB2	5.194782	14.27992	9.085139	0.04291
MMP1	0.366614	3.374773	3.008159	0.04295

SORBS2	6.008427	4.98779	-1.02064	0.042981
AGFG2	19.20783	21.61408	2.406257	0.043234
HAVCR1	0.54889	1.590366	1.041476	0.043345
LBX2-AS1	13.12923	11.64394	-1.48529	0.043353
LUM	17.97393	26.39239	8.418463	0.043383
IGHV1-69	5.714224	13.39247	7.678249	0.043827
SERPINE1	44.39085	61.41278	17.02193	0.044303
IGHV3-7	6.52584	13.70492	7.179079	0.04434
IGHV4-34	3.797019	11.57218	7.775156	0.044414
MGAT1	17.98181	19.34571	1.363899	0.044487
TMEM130	0.100818	1.13517	1.034352	0.044699
DSP	25.93762	30.84884	4.911218	0.044813
SFRP2	0.42248	4.708587	4.286107	0.044991
IGHA2	6.378392	17.40759	11.02919	0.045145
AEBP1	16.40311	30.69864	14.29553	0.04517
LINC0159!	6.862757	3.967567	-2.89519	0.045309
ABCB1	11.01719	8.959951	-2.05724	0.04547
MAP1LC3/	21.42818	26.01669	4.588508	0.045508
ETHE1	9.209647	10.24782	1.038175	0.045856
IGHV3-73	1.262085	9.046876	7.784791	0.046102
IRF8	3.246126	4.309537	1.063411	0.046144
ECSIT	22.33078	24.2092	1.878422	0.046418
PGAP6	17.3875	19.13832	1.750823	0.046475
AL136372.	3.685338	6.415592	2.730254	0.04648
CLINT1	13.80799	15.40701	1.599021	0.04669
RBP5	34.34743	27.67003	-6.67741	0.046735
GK	5.886768	4.777701	-1.10907	0.046995
CYP4F12	7.084434	5.814724	-1.26971	0.047091
FGF21	36.71837	25.93944	-10.7789	0.047422
LECT2	21.35956	16.28491	-5.07465	0.047592
AC010255	0.141077	1.817402	1.676325	0.047597
STAP2	26.057	29.53069	3.473689	0.047707
AJUBA	10.06441	8.680573	-1.38384	0.047787
VSIG2	2.374133	4.60054	2.226407	0.048014
USO1	16.69582	15.19732	-1.4985	0.048399
FN3K	23.15045	20.22657	-2.92388	0.048463
AKR1C4	87.06677	69.53226	-17.5345	0.049109
CBR1	88.29009	73.83102	-14.4591	0.049334
CACNA1H	3.784194	5.357688	1.573494	0.049516
TMEM123	80.21717	71.11533	-9.10184	0.049942
